# The global, regional, and national burden attributable to low bone mineral density, 1990–2020: an analysis of a modifiable risk factor from the Global Burden of Disease Study 2021

**DOI:** 10.1016/S2665-9913(25)00105-5

**Published:** 2025-09-16

**Authors:** GBD 2021 Low Bone Mineral Density Collaborators, Evelyn Hsieh, Dana Bryazka, Kanyin Liane Ong, Phoebe-Anne Rhinehart, Ewerton Cousin, Hailey Hagins, Cyrus Cooper, Marita Cross, Garland T Culbreth, Karsten E Dreinhoefer, Philippe Halbout, Jacek A Kopec, Sneha Ingle Nicholson, Daniel Prieto Alhambra, Anthony D Woolf, Theo Vos, Yohannes Habtegiorgis Abate, Sherief Abd-Elsalam, Meriem Abdoun, Mohamed Abouzid, Eman Abu-Gharbieh, Salahdein Aburuz, Abiola Victor Adepoju, Qorinah Estiningtyas Sakilah Adnani, Aqeel Ahmad, Haroon Ahmed, Luai A Ahmed, Syed Mahfuz Al Hasan, Tariq A Alalwan, Rasmieh Mustafa Al-amer, Hediyeh Alemi, Abid Ali, Yaser Mohammed Al-Worafi, Reza Amani, Abhishek Anil, Jalal Arabloo, Aleksandr Y Aravkin, Demelash Areda, Brhane Berhe Aregawi, Mohammad Asghari-Jafarabadi, Seyyed Shamsadin Athari, Sina Azadnajafabad, Ahmed Y Azzam, Ashish D Badiye, Nasser Bagheri, Sara Bagherieh, Saliu A Balogun, Maciej Banach, Shirin Barati, Pankaj Bhardwaj, Sonu Bhaskar, Gurjit Kaur Bhatti, Yasser Bustanji, Daniela Calina, Vijay Kumar Chattu, Endeshaw Chekol Abebe, Dinh-Toi Chu, Michael H Criqui, Natalia Cruz-Martins, Omid Dadras, Xiaochen Dai, Zhaoli Dai, Reza Darvishi Cheshmeh Soltani, Mohsen Dashti, Tadesse Asmamaw Dejenie, Cristian Del Bo', Edgar Denova-Gutiérrez, Vinoth Gnana Chellaiyan Devanbu, Syed Masudur Rahman Dewan, Vishal R Dhulipala, Michael Ekholuenetale, Mohamed A Elmonem, Farshid Etaee, Adeniyi Francis Fagbamigbe, Ildar Ravisovich Fakhradiyev, Ali Fatehizadeh, Alireza Feizkhah, Ginenus Fekadu, Bikila Regassa Feyisa, Florian Fischer, Abduzhappar Gaipov, Lucia Galluzzo, Mesfin Gebrehiwot, Fataneh Ghadirian, Tiffany K Gill, Kimiya Gohari, Ali Golchin, Bhawna Gupta, Sapna Gupta, Najah R Hadi, Arvin Haj-Mirzaian, Asif Hanif, Netanja I Harlianto, Ikramul Hasan, Md Saquib Hasnain, Amr Hassan, Simon I Hay, Jiawei He, Golnaz Heidari, Kamal Hezam, Yuta Hiraike, Praveen Hoogar, Chengxi Hu, Segun Emmanuel Ibitoye, Arad Iranmehr, Nahlah Elkudssiah Ismail, Masao Iwagami, Ali Jafari-Khounigh, Mihajlo Jakovljevic, Elham Jamshidi, Sathish Kumar Jayapal, Shubha Jayaram, Digisie Mequanint Jemere, Gwang Hun Jeong, Nitin Joseph, Charity Ehimwenma Joshua, Mikk Jürisson, Vidya Kadashetti, Sanjay Kalra, Morteza Abdullatif Khafaie, Himanshu Khajuria, Moien AB Khan, Javad Khanali, Shaghayegh Khanmohammadi, Moawiah Mohammad Khatatbeh, Sorour Khateri, Min Seo Kim, Oleksii Korzh, Kewal Krishan, Mukhtar Kulimbet, Vishnutheertha Kulkarni, Maria Dyah Kurniasari, Chandrakant Lahariya, Tri Laksono, Iván Landires, Kamaluddin Latief, Thao Thi Thu Le, Munjae Lee, Wei-Chen Lee, Erand Llanaj, Kashish Malhotra, Ahmad Azam Malik, Miquel Martorell, Andrea Maugeri, Hadush Negash Meles, Mohsen Merati, Tuomo J Meretoja, Tomislav Mestrovic, Alireza Mirahmadi, Nouh Saad Mohamed, Abdollah Mohammadian-Hafshejani, Ali H Mokdad, Lorenzo Monasta, Yousef Moradi, Negar Morovatdar, Shane Douglas Morrison, Ebrahim Mostafavi, Parsa Mousavi, Sumaira Mubarik, Christopher J L Murray, Sathish Muthu, Mohsen Naghavi, Pirouz Naghavi, Zuhair S Natto, Biswa Prakash Nayak, Mohammad Hadi Nematollahi, Duc Hoang Nguyen, Hien Quang Nguyen, Van Thanh Nguyen, Robina Khan Niazi, Efaq Ali Noman, Dieta Nurrika, Osaretin Christabel Okonji, Michal Ordak, Wael M S Osman, Yasamin Ostadi, Alicia Padron-Monedero, Shahina Pardhan, Pragyan Paramita Parija, Romil R Parikh, Jay Patel, Fanny Emily Petermann-Rocha, Hoang Tran Pham, Elton Junio Sady Prates, Ibrahim Qattea, Mehran Rahimi, Vafa Rahimi-Movaghar, Mosiur Rahman, Masoud Rahmati, Ivano Raimondo, Shakthi Kumaran Ramasamy, Sina Rashedi, Mohammad-Mahdi Rashidi, Salman Rawaf, Elrashdy M Redwan, Nazila Rezaei, Aly M A Saad, Umar Saeed, Amene Saghazadeh, Fatemeh Saheb Sharif-Askari, Amirhossein Sahebkar, Morteza Saki, Joseph W Sakshaug, Mohamed A Saleh, Yoseph Leonardo Samodra, Abdallah M Samy, Francesco Sanmarchi, Muhammad Arif Nadeem Saqib, Art Schuermans, Yashendra Sethi, Allen Seylani, Moyad Jamal Shahwan, Sunder Sham, Mohammed Shannawaz, Sadaf Sharfaei, Manoj Sharma, Seyed Afshin Shorofi, Emmanuel Edwar Siddig, Luís Manuel Lopes Rodrigues Silva, Jasvinder A Singh, Paramdeep Singh, Hamidreza Soleimani, Chandan Kumar Swain, Shima Tabatabai, Jacques Lukenze Tamuzi, Razieh Tavakoli Oliaee, Seyed Mohammad Tavangar, Masayuki Teramoto, Dufera Rikitu Terefa, Jansje Henny Vera Ticoalu, Asokan Govindaraj Vaithinathan, Tommi Juhani Vasankari, Siavash Vaziri, Fang Wang, Shu Wang, Juan Xia, Naohiro Yonemoto, Chuanhua Yu, Mazyar Zahir, Hanqing Zhao, Magdalena Zielińska, Osama A Zitoun, Lyn M March, Lidia Sanchez-Riera

**Affiliations:** a Section of Rheumatology, Allergy and Immunology, Yale University, New Haven, CT, USA; b Rheumatology Department, VA Connecticut Healthcare System, West Haven, CT, USA; c Institute for Health Metrics and Evaluation, University of Washington, Seattle, WA, USA; d Department of Health Metrics Sciences, School of Medicine, University of Washington, Seattle, WA, USA; e MRC Lifecourse Epidemiology Unit, University of Southampton, Southampton, UK; f Institute of Musculoskeletal Sciences, University of Oxford, Oxford, UK; g Faculty of Medicine and Health, University of Sydney, Sydney, NSW, Australia; h Global Alliance for Musculoskeletal Health, Sydney, NSW, Australia; i Center of Musculoskeletal Surgery, Charité Universitätsmedizin Berlin (Charité University Medical Center Berlin), Berlin, Germany; j IOF International Osteoporosis Foundation, Nyon, Switzerland; k School of Population and Public Health, University of British Columbia, Vancouver, BC, Canada; l Arthritis Research Canada, Richmond, BC, Canada; m Nuffield Department of Orthopaedics, Rheumatology, and Musculoskeletal Sciences, Oxford University, Oxford, UK; n Department of Medical Informatics, Erasmus University Medical Center, Rotterdam, Netherlands; o Osteoporosis Research Group, Lund University, Malmo, Sweden; p Global Alliance for Musculoskeletal Health, Truro, UK; q Department of Clinical Governance and Quality Improvement, Aleta Wondo General Hospital, Aleta Wondo, Ethiopia; r Department of Tropical Medicine and Infectious Diseases, Tanta University, Tanta, Egypt; s Department of Medicine, University of Setif Algeria, Sétif, Algeria; t Department of Health, Sétif, Algeria; u Department of Physical Pharmacy and Pharmacokinetics, Poznan University of Medical Sciences, Poznan, Poland; v Department of Clinical Sciences, University of Sharjah, Sharjah, United Arab Emirates; w Department of Biopharmaceutics and Clinical Pharmacy, University of Jordan, Amman, Jordan; x Department of Pharmacology and Therapeutics, United Arab Emirates University, Al Ain, United Arab Emirates; y College of Pharmacy, University of Jordan, Amman, Jordan; z Department of HIV and Infectious Diseases, Jhpiego, Abuja, Nigeria; aa Department of Adolescent Research and Care, Adolescent Friendly Research Initiative and Care, Ado Ekiti, Nigeria; bb Department of Public Health, Universitas Padjadjaran (Padjadjaran University), Bandung, Indonesia; cc College of Medicine, Shaqra University, Shaqra, Saudi Arabia; dd Department of Biosciences, COMSATS Institute of Information Technology, Islamabad, Pakistan; ee Institute of Public Health, United Arab Emirates University, Al Ain, United Arab Emirates; ff Division of Public Health Sciences, Department of Surgery, Washington University in St. Louis, St. Louis, MO, USA; gg Department of Biology, University of Bahrain, Zallaq, Bahrain; hh School of Nursing, Yarmouk University, Irbid, Jordan; ii School of Nursing and Midwifery, Western Sydney University, Sydney, NSW, Australia; jj Hematology, Oncology and Stem Cell Transplantation Research Center, Tehran University of Medical Sciences, Tehran, Iran; kk Department of Zoology, Abdul Wali Khan University Mardan, Mardan, Pakistan; ll Department of Medical Sciences, Azal University for Human Development, Sana'a, Yemen; mm Department of Clinical Sciences, University of Science and Technology of Fujairah, Fujairah, United Arab Emirates; nn Interdisciplinary Graduate Program in Human Toxicology, University of Iowa, Iowa City, IA, USA; oo Holden Comprehensive Cancer Center, University of Iowa Hospitals and Clinics, Iowa City, IA, USA; pp Department of Pharmacology, All India Institute of Medical Sciences, Jodhpur, India; qq All India Institute of Medical Sciences, Bhubaneswar, India; rr Health Management and Economics Research Center, Iran University of Medical Sciences, Tehran, Iran; ss Department of Applied Mathematics, University of Washington, Seattle, WA, USA; tt College of Art and Science, Ottawa University, Surprise, AZ, USA; uu School of Life Sciences, Arizona State University, Tempe, AZ, USA; vv College of Medicine and Health Sciences, Adigrat University, Adigrat, Ethiopia; ww Cabrini Research, Cabrini Health, Malvern, VIC, Australia; xx School of Public Health and Preventive Medicine, Monash University, Melbourne, VIC, Australia; yy Department of Immunology, Zanjan University of Medical Sciences, Zanjan, Iran; zz Department of Surgery, Washington University in St. Louis, St. Louis, MO, USA; aaa Leeds Institute of Rheumatic and Musculoskeletal Medicine, University of Leeds, Leeds, UK; bbb ASIDE Healthcare, Lewes, DE, USA; ccc Faculty of Medicine, October 6 University, 6th of October City, Egypt; ddd Department of Forensic Science, Government Institute of Forensic Science Nagpur, Nagpur, India; eee Rashtrasant Tukadoji Maharaj Nagpur University, Nagpur, India; fff Health Research Institute, University of Canberra, Canberra, ACT, Australia; ggg School of Medicine, Isfahan University of Medical Sciences, Isfahan, Iran; hhh Goldfields University Department of Rural Health, Curtin University, Kalgoorlie, WA, Australia; iii Department of Hypertension, Medical University of Lodz, Lodz, Poland; jjj Polish Mothers' Memorial Hospital Research Institute, Lodz, Poland; kkk Department of Anatomy, Saveh University of Medical Sciences, Saveh, Iran; lll Department of Community Medicine and Family Medicine, All India Institute of Medical Sciences, Jodhpur, India; mmm School of Public Health, All India Institute of Medical Sciences, Jodhpur, India; nnn Global Health Neurology Lab, NSW Brain Clot Bank, Sydney, NSW, Australia; ooo Division of Cerebrovascular Medicine and Neurology, National Cerebral and Cardiovascular Center, Suita, Japan; ppp Department of Medical Lab Technology, Chandigarh University, Mohali, India; qqq School of Pharmacy, The University of Jordan, Amman, Jordan; rrr Department of Basic Biomedical Sciences, University of Sharjah, Sharjah, United Arab Emirates; sss Department of Clinical Pharmacy, University of Medicine and Pharmacy of Craiova, Romania, Craiova, Romania; ttt Temerty Faculty of Medicine, University of Toronto, Toronto, ON, Canada; uuu Department of Community Medicine, Datta Meghe Institute of Medical Sciences, Sawangi, India; vvv Department of Medical Biochemistry, Debre Tabor University, Debre Tabor, Ethiopia; www The Interdisciplinary Research Group on Biomedicine and Health, VNU International School (VNUIS), Hanoi, Viet Nam; xxx Faculty of Applied Sciences, VNU International School (VNUIS), Hanoi, Viet Nam; yyy Department of Family Medicine and Public Health, University of California San Diego, La Jolla, CA, USA; zzz Life and Health Sciences Research Institute (ICVS), University of Minho, Braga, Portugal; aaaa Institute for Research and Innovation in Health (i3S), University of Porto, Porto, Portugal; bbbb Research Center for Child Psychiatry, University of Turku, Turku, Finland; cccc Iranian Research Center for HIV/AIDS (IRCHA), Tehran University of Medical Sciences, Tehran, Iran; dddd School of Population Health, University of New South Wales, Sydney, NSW, Australia; eeee School of Pharmacy and Charles Perkins Centre, University of Sydney, Sydney, NSW, Australia; ffff Department of Environmental Health, Arak University of Medical Sciences, Arak, Iran; gggg Immunology Research Center, Tabriz University of Medical Sciences, Tabriz, Iran; hhhh Department of Medical Biochemistry, University of Gondar, Gondar, Ethiopia; iiii Department of Food, Environmental and Nutritional Sciences, Università degli Studi di Milano (University of Milan), Milan, Italy; jjjj Center for Nutrition and Health Research, National Institute of Public Health, Cuernavaca, Mexico; kkkk Chettinad Hospital & Research Institute, Chettinad Academy of Research and Education, Chennai, India; llll Department of Pharmacy, United International University, Dhaka, Bangladesh; mmmm Pharmacology Division, Center for Life Sciences Research Bangladesh, Dhaka, Bangladesh; nnnn University of South Carolina, Columbia, SC, USA; oooo Faculty of Science and Health, University of Portsmouth, Hampshire, UK; pppp Department of Clinical and Chemical Pathology, Cairo University, Cairo, Egypt; qqqq Department of Internal Medicine, Yale University, New Haven, CT, USA; rrrr Department of Epidemiology and Medical Statistics, University of Ibadan, Ibadan, Nigeria; ssss Research Centre for Healthcare and Community, Coventry University, Coventry, UK; tttt Director of the Scientific and Technological Park, Kazakh National Medical University, Almaty, Kazakhstan; uuuu Department of Medicine, Korea University, Seoul, South Korea; vvvv School of Engineering, Edith Cowan University, Joondalup, WA, Australia; wwww Department of Social Medicine and Epidemiology, Guilan University of Medical Sciences, Rasht, Iran; xxxx Department of Infectious Diseases and Public Health, City University of Hong Kong, Hong Kong, China; yyyy Department of Pharmacy, Wollega University, Nekemte, Ethiopia; zzzz Institute of Health Sciences, Wollega University, Nekemte, Ethiopia; aaaaa Jimma University, Jimma, Ethiopia; bbbbb Institute of Public Health, Charité Universitätsmedizin Berlin (Charité Medical University Berlin), Berlin, Germany; ccccc Department of Medicine, Nazarbayev University, Astana, Kazakhstan; ddddd Department of Cardiovascular, Endocrine-metabolic Diseases, and Aging, ISS - Italian National Institute of Health, Rome, Italy; eeeee Department of Environmental Health, Wollo University, Dessie, Ethiopia; fffff School of Nursing and Midwifery, Shahid Beheshti University of Medical Sciences, Tehran, Iran; ggggg Adelaide Medical School, University of Adelaide, Adelaide, SA, Australia; hhhhh Department of Biostatistics, Tarbiat Modares University, Tehran, Iran; iiiii Quantitative Department, Non-Communicable Diseases Research Center (NCDRC), Tehran, Iran; jjjjj Department of Applied Cell Sciences, Urmia University of Medical Sciences, Urmia, Iran; kkkkk Cellular and Molecular Medicine Institute, Urmia University of Medical Sciences, Urmia, Iran; lllll Department of Public Health, Torrens University Australia, Melbourne, VIC, Australia; mmmmm Department of Toxicology, Shriram Institute for Industrial Research, Delhi, India; nnnnn Department of Clinical Pharmacology and Medicine, University of Kufa, Najaf, Iraq; ooooo Department of Radiology, Massachusetts General Hospital, Boston, MA, USA; ppppp Obesity Research Center, Shahid Beheshti University of Medical Sciences, Tehran, Iran; qqqqq Sakarya University, Turkey, Sakarya, Türkiye; rrrrr Faculty of Medicine, Utrecht University, Utrecht, Netherlands; sssss Department of Radiology, University Medical Center Utrecht, Utrecht, Netherlands; ttttt Department of Pharmaceutical Technology, University of Dhaka, Dhaka, Bangladesh; uuuuu Department of Pharmacy, Marwadi University, Rajkot, India; vvvvv Department of Neurology, Cairo University, Cairo, Egypt; wwwww Independent Consultant, Santa Clara, CA, USA; xxxxx Department of Microbiology, Taiz University, Taiz, Yemen; yyyyy School of Medicine, Nankai University, Tianjin, China; zzzzz Graduate School of Medicine, University of Tokyo, Tokyo, Japan; aaaaaa School of Social Sciences, The Apollo University, Chittoor, India; bbbbbb Department of Psychology, Tsinghua University, Beijing, China; cccccc Department of Health Promotion and Education, University of Ibadan, Ibadan, Nigeria; dddddd Department of Neurosurgery, Tehran University of Medical Sciences, Tehran, Iran; eeeeee Department of Clinical Pharmacy & Pharmacy Practice, Asian Institute of Medicine, Science and Technology, Bedong, Malaysia; ffffff Malaysian Academy of Pharmacy, Puchong, Malaysia; gggggg Department of Health Services Research, University of Tsukuba, Tsukuba, Japan; hhhhhh Department of Non-Communicable Disease Epidemiology, London School of Hygiene & Tropical Medicine, London, UK; iiiiii Road Traffic Injury Research Center, Tabriz University of Medical Sciences, Tabriz, Iran; jjjjjj The World Academy of Sciences UNESCO, Trieste, Italy; kkkkkk Shaanxi University of Technology, Hanzhong, China; llllll Johns Hopkins University, Baltimore, MD, USA; mmmmmm Centre of Studies and Research, Ministry of Health, Muscat, Oman; nnnnnn Department of Biochemistry, Government Medical College, Mysuru, India; oooooo Caring Futures Institute, Flinders University, Adelaide, SA, Australia; pppppp Geumsan Public Health Center, Geumsan-gun, South Korea; qqqqqq College of Medicine, Gyeongsang National University, Jinju, South Korea; rrrrrr Department of Community Medicine, Manipal Academy of Higher Education, Mangalore, India; ssssss Department of Economics, National Open University, Benin City, Nigeria; tttttt Institute of Family Medicine and Public Health, University of Tartu, Tartu, Estonia; uuuuuu Department of Oral and Maxillofacial Pathology, Krishna Vishwa Vidyapeeth (Deemed to be University), Karad, India; vvvvvv Department of Endocrinology, Bharti Hospital Karnal, Karnal, India; wwwwww University Centre for Research and Development, Chandigarh University, Mohali, India; xxxxxx Department of Public Health, Ahvaz Jundishapur University of Medical Sciences, Ahvaz, Iran; yyyyyy Environmental Technologies Research Center, Medical Basic Sciences Research Institute, Ahvaz Jundishapur University of Medical Sciences, Ahvaz, Iran; zzzzzz Amity Institute of Forensic Sciences, Amity University, Noida, India; aaaaaaa Family Medicine Department, United Arab Emirates University, Al Ain, United Arab Emirates; bbbbbbb Primary Care Department, NHS North West London, London, UK; ccccccc Social Determinants of Health Research Center, Shahid Beheshti University of Medical Sciences, Tehran, Iran; ddddddd Non-communicable Diseases Research Center, Tehran University of Medical Sciences, Tehran, Iran; eeeeeee Department of Epidemiology, Non-Communicable Diseases Research Center (NCDRC), Tehran, Iran; fffffff School of Medicine, Tehran University of Medical Sciences, Tehran, Iran; ggggggg Department of Basic Medical Sciences, Yarmouk University, Irbid, Jordan; hhhhhhh School of Medicine, Kurdistan University of Medical Sciences, Sanandaj, Iran; iiiiiii Broad Institute of MIT and Harvard, Cambridge, MA, USA; jjjjjjj Massachusetts General Hospital, Boston, MA, USA; kkkkkkk Department of General Practice and Family Medicine, Kharkiv National Medical University, Kharkiv, Ukraine; lllllll Department of Anthropology, Panjab University, Chandigarh, India; mmmmmmm Research and Publication Activity Division, Kazakh National Medical University, Almaty, Kazakhstan; nnnnnnn Center of Medicine and Public Health, Asfendiyarov Kazakh National Medical University, Almaty, Kazakhstan; ooooooo Department of Medicine, Queensland Health, Brisbane, QLD, Australia; ppppppp Faculty of Medicine and Health Science, Universitas Kristen Satya Wacana, Salatiga, Indonesia; qqqqqqq School of Nursing, Taipei Medical University, Taipei, Taiwan; rrrrrrr Division of Evidence Synthesis, Foundation for People-centric Health Systems, New Delhi, India; sssssss Division of Lifestyle Medicine, Centre for Health: The Specialty Practice, New Delhi, India; ttttttt Department of Physiotherapy, Universitas Aisyiyah Yogyakarta, Yogyakarta, Indonesia; uuuuuuu Institute of Allied Health Sciences, National Cheng Kung University, Tainan, Taiwan; vvvvvvv Unidad de Genética y Salud Pública, Instituto de Ciencias Médicas, Las Tablas, Panama; wwwwwww Ministry of Health, Hospital Joaquín Pablo Franco Sayas, Las Tablas, Panama; xxxxxxx Centre for Family Welfare, University of Indonesia, Depok, Indonesia; yyyyyyy Department of Global Health and Health Security, Taipei Medical University, Taipei, Taiwan; zzzzzzz University of Medicine and Pharmacy at Ho Chi Minh City, Ho Chi Minh City, Viet Nam; aaaaaaaa Department of Medical Science, Ajou University School of Medicine, Suwon, South Korea; bbbbbbbb Department of Family Medicine, University of Texas Medical Branch, Galveston, TX, USA; cccccccc Department of Molecular Epidemiology, German Institute of Human Nutrition Potsdam-Rehbrücke, Potsdam, Germany; dddddddd German Center for Diabetes Research (DZD), München-Neuherberg, Germany; eeeeeeee Rama Medical College Hospital and Research Centre, Uttar Pradesh, India; ffffffff Institute of Applied Health Research, University of Birmingham, Birmingham, UK; gggggggg Rabigh Faculty of Medicine, King Abdulaziz University, Jeddah, Saudi Arabia; hhhhhhhh Department of Nutrition and Dietetics, University of Concepción, Concepción, Chile; iiiiiiii Centre for Healthy Living, University of Concepción, Concepción, Chile; jjjjjjjj Department of Medical and Surgical Sciences and Advanced Technologies ""GF Ingrassia"", University of Catania, Catania, Italy; kkkkkkkk Department of Medical Laboratory Sciences, Adigrat University, Adigrat, Ethiopia; llllllll Comprehensive Cancer Center, Helsinki University Hospital, Helsinki, Finland; mmmmmmmm University of Helsinki, Helsinki, Finland; nnnnnnnn University Centre Varazdin, University North, Varazdin, Croatia; oooooooo Department of Orthopedics, Shahid Beheshti University of Medical Sciences, Tehran, Iran; pppppppp Molecular Biology Unit, Sirius Training and Research Centre, Khartoum, Sudan; qqqqqqqq Bio-Statistical and Molecular Biology Department, Sirius Training and Research Centre, Khartoum, Sudan; rrrrrrrr Modeling in Health Research Center, Shahrekord University of Medical Sciences, Shahrekord, Iran; ssssssss Clinical Epidemiology and Public Health Research Unit, Burlo Garofolo Institute for Maternal and Child Health, Trieste, Italy; tttttttt Department of Epidemiology and Biostatistics, Kurdistan University of Medical Sciences, Sanandaj, Iran; uuuuuuuu Clinical Research Development Unit, Mashhad University of Medical Sciences, Mashhad, Iran; vvvvvvvv Division of Plastic and Reconstructive Surgery, University of Washington Medical Center, Seattle, WA, USA; wwwwwwww Department of Medicine, Stanford University, Palo Alto, CA, USA; xxxxxxxx Stanford Cardiovascular Institute, Stanford University, Palo Alto, CA, USA; yyyyyyyy Unit of Pharmacotherapy, Epidemiology and Economics, Rijksuniversiteit Groningen (University of Groningen), Groningen, Netherlands; zzzzzzzz Department of Epidemiology and Biostatistics, Wuhan University, Wuhan, China; aaaaaaaaa Department of Research Methods, Orthopaedic Research Group, Coimbatore, India; bbbbbbbbb Department of Biotechnology, Karpagam Academy of Higher Education (Deemed to be University), Coimbatore, India; ccccccccc Department of Computer Science, University of Illinois, Urbana, IL, USA; ddddddddd Department of Dental Public Health, King Abdulaziz University, Jeddah, Saudi Arabia; eeeeeeeee Department of Health Policy and Oral Epidemiology, Harvard University, Boston, MA, USA; fffffffff Applied Cellular and Molecular Research Center, Kerman University of Medical Sciences, Kerman, Iran; ggggggggg Cardiovascular Laboratory, Methodist Hospital, Merrillville, IN, USA; hhhhhhhhh Department of Allergy, Immunology and Dermatology, Hanoi Medical University, Hanoi, Viet Nam; iiiiiiiii Cardiovascular Research Department, Methodist Hospital, Merrillville, IN, USA; jjjjjjjjj Tuberculosis Group, Oxford University Clinical Research Unit, Vietnam, Ho Chi Minh City, Viet Nam; kkkkkkkkk Department of General Medicine, University of Medicine and Pharmacy at Ho Chi Minh City, Ho Chi Minh City, Viet Nam; lllllllll International Islamic University Islamabad, Islamabad, Pakistan; mmmmmmmmm School of Chemical & Biomolecular Engineering, University of Sydney, Sydney, NSW, Australia; nnnnnnnnn Faculty of Applied Sciences, Taiz University, Taiz, Yemen; ooooooooo Department of Public Health, Banten School of Health Science, South Tangerang, Indonesia; ppppppppp Ministry of Research, Technology and Higher Education, Higher Education Service Institutions (LL-DIKTI) Region IV, Bandung, Indonesia; qqqqqqqqq School of Pharmacy, University of the Western Cape, Cape Town, South Africa; rrrrrrrrr Department of Pharmacotherapy and Pharmaceutical Care, Medical University of Warsaw, Warsaw, Poland; sssssssss Department of Biology, Khalifa University, Abu Dhabi, United Arab Emirates; ttttttttt School of Medicine, Shahid Beheshti University of Medical Sciences, Tehran, Iran; uuuuuuuuu National School of Public Health, Institute of Health Carlos III, Madrid, Spain; vvvvvvvvv Vision and Eye Research Institute, Anglia Ruskin University, Cambridge, UK; wwwwwwwww Department of Community Medicine, All India Institute of Medical Sciences, Jammu, India; xxxxxxxxx Division of Health Policy and Management, University of Minnesota, Minneapolis, MN, USA; yyyyyyyyy Global Health Governance Programme, University of Edinburgh, Edinburgh, UK; zzzzzzzzz School of Dentistry, University of Leeds, Leeds, UK; aaaaaaaaaa Facultad de Medicina (Faculty of Medicine), Universidad Diego Portales (Diego Portales University), Santiago, Chile; bbbbbbbbbb School of Cardiovascular and Metabolic Health, University of Glasgow, Glasgow, UK; cccccccccc Department of Internal Medicine, Weiss Memorial Hospital, Chicago, IL, USA; dddddddddd Department of Maternal-Child Nursing and Public Health, Federal University of Minas Gerais, Belo Horizonte, Brazil; eeeeeeeeee Department of Neonatology, Case Western Reserve University, Akron, OH, USA; ffffffffff Cardiovascular Research Center, Tabriz University of Medical Sciences, Tabriz, Iran; gggggggggg Sina Trauma and Surgery Research Center, Tehran University of Medical Sciences, Tehran, Iran; hhhhhhhhhh Department of Population Science and Human Resource Development, University of Rajshahi, Rajshahi, Bangladesh; iiiiiiiiii Health Service Research and Quality of Life Center (CEReSS), Aix-Marseille University, Marseille, France; jjjjjjjjjj Department of Medical, Surgical and Experimental Sciences, University of Sassari, Sassari, Italy; kkkkkkkkkk Gynecology and Breast Care Center, Mater Olbia Hospital, Olbia, Italy; llllllllll Department of Radiology, Stanford University, Stanford, CA, USA; mmmmmmmmmm Brigham and Women's Hospital, Harvard Medical School, Boston, MA, USA; nnnnnnnnnn Department of Primary Care and Public Health, Imperial College London, London, UK; oooooooooo Academic Public Health England, Public Health England, London, UK; pppppppppp Department of Biological Sciences, King Abdulaziz University, Jeddah, Egypt; qqqqqqqqqq Department of Protein Research, Research and Academic Institution, Alexandria, Egypt; rrrrrrrrrr Cardiovascular Department, Zagazig University, Zagazig, Egypt; ssssssssss Operational Research Center in Healthcare, Near East University (NEU), Nicosia Cyprus, Türkiye; tttttttttt International Center of Medical Sciences Research (ICMSR), Islamabad, Pakistan; uuuuuuuuuu Research Center for Immunodeficiencies, Tehran University of Medical Sciences, Tehran, Iran; vvvvvvvvvv Sharjah Institute of Medical Sciences, University of Sharjah, Sharjah, United Arab Emirates; wwwwwwwwww Center for Global Health Research, Saveetha University, Chennai, India; xxxxxxxxxx Biotechnology Research Center, Mashhad University of Medical Sciences, Mashhad, Iran; yyyyyyyyyy Department of Microbiology, Ahvaz Jundishapur University of Medical Sciences, Ahvaz, Iran; zzzzzzzzzz LMU-Munich, Munich, Germany; aaaaaaaaaaa Institute for Employment Research, Nuremberg, Germany; bbbbbbbbbbb College of Medicine, University of Sharjah, Sharjah, United Arab Emirates; ccccccccccc Faculty of Pharmacy, Mansoura University, Mansoura, Egypt; ddddddddddd Institute of Epidemiology and Preventive Medicine, National Taiwan University, Taipei, Taiwan; eeeeeeeeeee Benang Merah Research Center (BMRC), Minahasa Utara, Indonesia; fffffffffff Department of Entomology, Ain Shams University, Cairo, Egypt; ggggggggggg Medical Ain Shams Research Institute (MASRI), Ain Shams University, Cairo, Egypt; hhhhhhhhhhh Department of Biomedical and Neuromotor Sciences, University of Bologna, Bologna, Italy; iiiiiiiiiii Primary Healthcare Department, Azienda USL di Bologna, Bologna, Italy; jjjjjjjjjjj Research Development Coordination Section, Pakistan Health Research Council, Islamabad, Pakistan; kkkkkkkkkkk School of Sciences, University of Management and Technology, Lahore, Pakistan; lllllllllll Faculty of Medicine, Katholieke Universiteit Leuven, Leuven, Belgium; mmmmmmmmmmm Department of Cardiovascular Sciences, Katholieke Universiteit Leuven, Leuven, Belgium; nnnnnnnnnnn Department of Medicine, Swami Vivekanand Subharti University, Meerut, India; ooooooooooo National Heart, Lung, and Blood Institute, National Institutes of Health, Rockville, MD, USA; ppppppppppp Center for Medical and Bio-Allied Health Sciences Research, Ajman University, Ajman, United Arab Emirates; qqqqqqqqqqq Department of Pathology and Laboratory Medicine, Northwell Health, New York, NY, USA; rrrrrrrrrrr Amity Institute of Public Health, Amity University, Noida, India; sssssssssss Department of Safety Services, Baim Institute for Clinical Research, Boston, MA, USA; ttttttttttt Beth Israel Deaconess Medical Center, Harvard University, Boston, MA, USA; uuuuuuuuuuu Department of Social and Behavioral Health, University of Nevada Las Vegas, Las Vegas, NV, USA; vvvvvvvvvvv Department of Medical-Surgical Nursing, Mazandaran University of Medical Sciences, Sari, Iran; wwwwwwwwwww Department of Nursing and Health Sciences, Flinders University, Adelaide, SA, Australia; xxxxxxxxxxx Unit of Basic Medical Sciences, University of Khartoum, Khartoum, Sudan; yyyyyyyyyyy Department of Medical Microbiology and Infectious Diseases, Erasmus University, Rotterdam, Netherlands; zzzzzzzzzzz Sport Physical Activity and Health Research & Innovation Center (SPRINT), Polytechnic Institute of Guarda, Guarda, Portugal; aaaaaaaaaaaa CICS-UBI Health Sciences Research Center, University of Beira Interior, Covilhã, Portugal; bbbbbbbbbbbb School of Medicine, Baylor College of Medicine, Houston, TX, USA; cccccccccccc Department of Medicine Service, US Department of Veterans Affairs (VA), Houston, TX, USA; dddddddddddd Department of Radiodiagnosis, All India Institute of Medical Sciences, Bathinda, India; eeeeeeeeeeee Department of Analytical and Applied Economics, Utkal University, Bhubaneswar, India; ffffffffffff Department of Medical Education, Shahid Beheshti University of Medical Sciences, Tehran, Iran; gggggggggggg Department of Epidemiology, Stellenbosch University, Cape Town, South Africa; hhhhhhhhhhhh Department of Medicine, Northlands Medical Group, Omuthiya, Namibia; iiiiiiiiiiii Basic Sciences in Infectious Diseases Research Center, Shiraz University of Medical Sciences, Shiraz, Iran; jjjjjjjjjjjj Department of Pathology, Tehran University of Medical Sciences, Tehran, Iran; kkkkkkkkkkkk Department of Preventive Medicine, Northwestern University, Chicago, IL, USA; llllllllllll Outpatient Department, Wollega University, Bedele Town, Ethiopia; mmmmmmmmmmmm Department of Public Health, Wollega University, Nekemte, Ethiopia; nnnnnnnnnnnn Faculty of Public Health, Universitas Sam Ratulangi (Sam Ratulangi University), Manado, Indonesia; oooooooooooo College of Health and Sport Sciences, University of Bahrain, Zallaq, Bahrain; pppppppppppp UKK Institute, Tampere, Finland; qqqqqqqqqqqq Faculty of Medicine and Health Technology, Tampere University, Tampere, Finland; rrrrrrrrrrrr Department of Infectious Disease, Kermanshah University of Medical Sciences, Kermanshah, Iran; ssssssssssss School of Public Health, Xuzhou Medical University, Xuzhou, China; tttttttttttt Department of Neurosurgery, Capital Medical University, Beijing, China; uuuuuuuuuuuu Department of Neurosurgery, Beijing Tiantan Hospital, Beijing, China; vvvvvvvvvvvv School of Public Health, Capital Medical University, Beijing, China; wwwwwwwwwwww Department of Biostatistics, University of Toyama, Toyama, Japan; xxxxxxxxxxxx Department of Public Health, Juntendo University, Tokyo, Japan; yyyyyyyyyyyy Norris Comprehensive Cancer Center, Keck School of Medicine, University of Southern California, Los Angeles, CA, USA; zzzzzzzzzzzz College of Traditional Chinese Medicine, Hebei University, Baoding, China; aaaaaaaaaaaaa Department of Biochemistry and Pharmacogenomics, Medical University of Warsaw, Warsaw, Poland; bbbbbbbbbbbbb School of Public Health Sciences, University of Waterloo, Waterloo, ON, Canada; ccccccccccccc College of Medicine, Sulaiman Alrajhi University, Al Bukairiyah, Saudi Arabia; ddddddddddddd Department of Rheumatology, Royal North Shore Hospital, St Leonards, NSW, Australia; eeeeeeeeeeeee Institute of Bone and Joint Research, University of Sydney, Sydney, NSW, Australia

## Abstract

**Background:**

Fractures related to osteoporosis and low bone mineral density lead to substantial morbidity, mortality, and cost to individuals and health systems. Here we present the most up-to-date global, regional, and national estimates of the contribution of low bone mineral density to the burden of fractures from falls and additional categories of injuries from the Global Burden of Diseases, Injuries, and Risk Factors Study (GBD) 2021.

**Methods:**

The burden of low bone mineral density was estimated from 1990 to 2020 in terms of years lived with disability (YLDs), disability-adjusted life years (DALYs), and deaths, for individuals aged 40 years and older, using data from population-based studies from 48 countries or territories (169 unique sources). Mean standardised femoral neck bone mineral density values were estimated by GBD location, age, and sex by meta-regression. Based on a separate meta-analysis of population-based studies from nine countries (12 unique sources), we also estimated the pooled relative risk of fractures per unit decrease in bone mineral density (g/cm^2^). The population-attributable fraction for low bone mineral density was calculated by comparing the observed distributions of standardised femoral neck bone mineral density to an age-specific and sex-specific counterfactual distribution, defined as the 99th percentile of five rounds of the National Health and Nutrition Examination Survey in the USA, by 5-year age group and sex. Hospital and emergency department data were used to derive the incidence of fractures for six categories of injury (road injuries, other transport injuries, falls, non-venomous animal contact, exposure to mechanical forces, and physical interpersonal violence) using ICD codes. Deaths due to fractures were estimated as the proportion of in-hospital deaths due to the specified injury causes for which a fracture (nature of injury code) was more severe than the cause of injury code. YLDs and DALYs attributable to low bone mineral density by cause of injury were also determined according to previous GBD methods.

**Findings:**

In 2020, 8·32 million (95% UI 5·58–10·84) YLDs, 17·2 million (14·1–20·2) DALYs, and 477 000 (411 000–536 000) deaths were attributable to low bone mineral density globally in individuals aged 40 years and older. Between 1990 and 2020, global YLDs, DALYs, and deaths attributable to low bone mineral density increased by 91·8% (88·5–95·1), 89·8% (81·5–99·0), and 127·1% (108·5–144·5), respectively. Over this period, the age-standardised global rates of YLDs, DALYs, and deaths attributable to low bone mineral density showed modest decreases. In 2020, falls accounted for 76·2% (95% UI 74·2–78·3) of YLDs, 65·2% (62·9–67·6) of DALYs, and 71·0% (67·4–72·8) of deaths attributable to low bone mineral density, and road injuries largely accounted for the remaining amount: 12·4% (11·1–13·6) of YLDs, 24·6% (22·5–27·1) of DALYs, and 23·1% (21·6–26·2) of deaths. As a proportion of all fall-related burden, low bone mineral density accounted for 26·6% (23·2–28·7) of YLDs, 25·6% (22·1–27·4) of DALYs, and 40·6% (35·4–44·0) of deaths in 2020. Of all road injury-related burden, 12·6% (10·8–13·5) of YLDs, 6·3% (5·4–6·9) of DALYs, and 8·9% (7·6–9·6) of deaths were attributable to low bone mineral density. In men, road injuries accounted for the largest proportion of DALYs attributable to low bone mineral density in those aged 40–59 years and the largest proportion of deaths in those aged 40–64 years. In women, road injuries were the leading cause of DALYs attributable to low bone mineral density in those aged 40–44 years and the leading cause of deaths attributable to low bone mineral density in those aged 40–54 years. In older age groups among both men and women, falls were the leading cause of the burden attributable to low bone mineral density.

**Interpretation:**

Low bone mineral density is a crucial modifiable risk factor for fractures, which are an important cause of morbidity and mortality particularly in ageing populations. This analysis highlights low bone mineral density as a cause of health loss not just from falls, but also from road injuries.

**Funding:**

Gates Foundation.

**Research in context**

**Evidence before this study**

Low bone mineral density is a reliably measurable and modifiable risk factor for fractures at the hip, spine, and other skeletal sites. Low bone mineral density is acknowledged as a risk factor for fractures related to falls and minor trauma, but few studies to date have explored the relationship between bone mineral density and major non-fall-related injuries. The Global Burden of Diseases, Injuries, and Risk Factors Study (GBD) analyses low bone mineral density as a risk factor that directly contributes to quantifiable disease burden from fractures. For GBD 2015 we searched PubMed for population-based studies reporting femoral neck bone mineral density measured by dual-energy x-ray absorptiometry from Jan 1, 1980, to Dec 31, 2015, using the search terms (osteoporosis OR osteopenia OR osteopaenia OR bone mineral density OR radiolucency) AND (prevalen* OR inciden* OR cross-sectional OR cross sectional OR epidemi* OR survey OR population-based OR population based OR population study OR population sample OR cohort OR follow-up OR follow up OR longitudinal OR regist* OR data collection). Additional studies encountered opportunistically during data review were added for GBD 2016, 2017, 2019, and 2021. In parallel, a systematic search of PubMed done for GBD 2019 and updated for GBD 2021 identified studies published from Jan 1, 2010, to Dec 31, 2020, reporting the relative risk of hip or non-hip fracture per change in bone mineral density, using the search terms (bone mineral density[title/abstract] OR bone mineral densities[title/abstract]) OR bone density[Mesh]) AND (mean[title/abstract] OR average[title/abstract]) AND risk[Mesh]) AND fracture[title/abstract]).

**Added value of this study**

This synthesis provides updated and comprehensive estimates of the global burden from fractures attributable to low bone mineral density from 1990 to 2020, and quantifies the contribution of six categories of injury (falls, road injuries, other transport injuries, exposure to mechanical forces, animal contact, and interpersonal violence) in men and women aged 40 years and older. An increase in burden related to low bone mineral density was observed, with a nearly two-times increase in years lived with disability and disability-adjusted life-years and a greater than two-times increase in deaths attributable to low bone mineral density. Among the types of injury, road injuries were the largest contributor to burden attributable to low bone mineral density among middle-aged men, and a main contributor among middle-aged women. In older individuals, falls represented the predominant cause of burden attributable to low bone mineral density.

**Implications of all the available evidence**

Low bone mineral density is an important predictor of fracture that is modifiable by existing prevention and treatment interventions. Strengthened policy and implementation strategies are needed to improve the uptake of evidence-based injury and fracture prevention approaches and to promote lifestyle strategies, to support bone health across the life-course.

## Introduction

Fractures related to osteoporosis represent a substantial burden and economic cost for societies across the world, and a steady increase in incidence is forecast over the coming decades.^1^, ^2^ In addition to the pain, functional impairment, and mortality risks associated with a fracture in the short term, the lasting consequences of bone fractures include chronic pain, permanent disability, and long-term institutionalisation.^3^, ^4^ A study of the Danish National Hospital Discharge Registry showed that 1 year after a hip fracture event, cumulative mortality was 37·1% in men and 26·4% in women, compared with 9·9% in men and 9·3% in women in the general population.^5^ In the USA, approximately half of individuals who have osteoporotic fractures (also known as fragility fractures) never regain their pre-fracture level of physical function, and many lose their independence, requiring long-term care.^6^ Furthermore, estimates show that fragility fractures lead to higher costs for individuals and health-care systems than other common disabling disorders such as myocardial infarction, stroke, Parkinson's disease, and rheumatoid arthritis.^7^, ^8^

Cost-effective strategies exist for the identification and treatment of individuals with low bone mineral density who are at high risk of fracture,^9^, ^10^ yet there is little awareness regarding these strategies among health professionals, public health institutions, and the general population, as highlighted in a report of EU countries.^7^ Low bone mineral density has been consistently shown to predict fragility fractures at the hip and other sites.^11^ Standard methods to measure bone mineral density exist using dual-energy x-ray absorptiometry (DXA), allowing for the comparison of values between different populations, and the establishment of diagnostic and treatment thresholds.^12^, ^13^

We previously estimated the burden of falls attributable to low bone mineral density in persons aged 50 years and older as part of the Global Burden of Diseases, Injuries, and Risk Factors Study (GBD) 2010.^14^ In this updated and expanded analysis of the GBD 2021 dataset, which encompasses data from 1990 to 2020, we estimate the burden—including years lived with disability (YLDs), disability-adjusted life years (DALYs), and mortality—attributable to low bone mineral density in people aged 40 years and older in the setting of falls, road injuries and other transport injuries, exposure to mechanical forces, animal contact, and interpersonal violence.

## Methods

### Overview

This Article was produced as part of the GBD Collaborator Network and in accordance with the GBD Protocol. This study adheres to the GATHER statement^15^ and follows the comparative risk assessment methodology used in the GBD, as previously described.^14^, ^16^ Briefly, by this methodology, the burden due to a risk factor is compared to a hypothetical counterfactual exposure level that would result in the lowest attributable burden, referred to as the theoretical minimum risk exposure level (TMREL; appendix 1 p 1).^14^, ^16^ Full details of the methods are included in appendix 1 (pp 14–31).

### Case definition and input data

The case definition for bone mineral density refers to bone mineral density measured at the femoral neck by DXA in g/cm^2^. A systematic review was done for GBD 2010 to identify all population-based studies published from Jan 1, 1980, to Dec 31, 2010, reporting femoral neck bone mineral density (mean [SD]) measured by DXA.^14^ This search was last updated during GBD 2015, but with each successive round of the GBD up to GBD 2021, new sources suggested by collaborators within the GBD Network or identified in the Global Health Data Exchange database by GBD librarians (keywords: bone mineral density or osteoporosis) have been included. All sources up to GBD 2021 are available online in the GBD 2021 Sources Tool. Details of the search strategy, databases, inclusion and exclusion criteria, risk of bias assessment, and data extraction have been described previously.^14^, ^16^ All mean (SD) values for femoral neck bone mineral density from eligible studies, measured using DXA systems from different manufacturers, were extracted and standardised by international conversion formulas to mean standardised femoral neck bone mineral density and standardised SD.^17^ Mean standardised femoral neck bone mineral density values were estimated as a continuous parameter for each GBD location, age, and sex, from 1990 to 2020 using DisMod-MR 2.1 (Disease Modelling Meta-Regression; version 2.1), a Bayesian meta-regression tool developed specifically for the GBD.^18^ DisMod-MR 2.1 was chosen due to its ability to leverage information across locations, years, and heterogeneous age groups to generate estimates when data are sparse or missing. Estimates were generated for 204 countries and territories and for the GBD regions and super-regions. Age was modelled in 5-year intervals starting from 40–44 years, with the final category covering ages 95 years and older. Estimates were modelled up to 2020 to align with other papers in a recent musculoskeletal series by GBD collaborators.^19^

### Defining the TMREL for low bone mineral density

To select counterfactual exposure levels of bone mineral density, defined as the exposure level with no or very low exposure to low bone mineral density, the TMREL was taken as the 99th percentile of the femoral neck bone mineral density values (mean [SD]), by 5-year age groups (from age 40–44 years up to ≥95 years) and sex, from five different cycles (years 1988–94, 2005–06, 2007–08, 2009–10, and 2013–14) of the US National Health and Nutrition Examination Survey,^20^, ^21^, ^22^, ^23^, ^24^ as the most broadly accepted standard international reference. Low bone mineral density exposure was defined as the difference between the mean bone mineral density of a population and the TMREL at the same age and sex. This meant that low bone mineral density exposure in a population was quantified as the amount by which the population's mean bone mineral density fell below the TMREL for the same age and sex. If the mean bone mineral density was higher than the TMREL, the exposure was considered zero.

### Risk estimation

To establish the relationship between low bone mineral density and fracture risk we conducted a meta-analysis. A systematic review originally conducted for GBD 2019 and updated for GBD 2021 identified six studies that reported fracture risk per SD of bone mineral density.^25^, ^26^, ^27^, ^28^, ^29^, ^30^ Specifically, studies were eligible for inclusion if they: included representative, population-based longitudinal data; reported femoral neck bone mineral density measured by DXA as the exposure variable; reported relative risk of fractures per change in bone mineral density (most often captured as the per 1 SD decrease in bone mineral density); and included fractures (including type of fractures) as an outcome of interest. These studies were supplemented with six additional studies^31^, ^32^, ^33^, ^34^, ^35^, ^36^ meeting criteria for inclusion (from a larger meta-analysis of 12 studies that previously estimated the relative risk of fragility fracture associated with low bone mineral density^11^). The relationship between low bone mineral density and fracture risk was subsequently modelled using a meta-regression–Bayesian, regularised, trimmed meta-analysis.^37^ Relative risks were estimated separately for hip and non-hip fractures (appendix 1 p 2).

### Estimate projections

The attributable burden of fractures due to low bone mineral density was calculated by comparing the observed distribution of standardised femoral neck bone mineral density to the counterfactual distribution for each age group, sex, location, year, and cause according to the following formula, as described previously:^14^

$${\text{PAF}}_{oasgt}=\frac{\int_{x=l}^{u}{\text{RR}}_{o}(x){\text{P}}_{asgt}(x)dx-{\text{RR}}_{o}(TMREL_{as})}{\int_{x=l}^{\text{u}}{\text{RR}}_{o}(x){\text{P}}_{asgt}(x)dx}$$

where PAF_oasgt_ represents the population attributable fraction for outcome *o* (ie, hip or non-hip fracture), age group *a*, sex *s*, location *g*, and year *t*; RR_o_ (*x*) is the relative risk at exposure level *x* for outcome *o* with the lowest observed exposure as *l* and the highest as *u*; P_asgt_(*x*) is the exposure at level *x* for age group *a*, sex *s*, location *g*, and year *t*; TMREL_as_ is the TMREL for age group *a* and sex *s*.

### Risk estimation for injuries

In view of the evidence showing the similar role of bone mineral density in both low-energy and high-energy trauma,^38^ the estimation of burden attributable to low bone mineral density included fractures from unintentional injuries (falls, non-venomous animal contact, and exposure to mechanical forces [an aggregate of two causes: unintentional firearms and other exposure to mechanical forces]), from transportation injuries, including road injuries (pedestrian road injuries, cyclist road injuries, motorcyclist road injuries, motor vehicle road injuries) and other transport injuries (eg, trains, ferries, and aeroplanes), and interpersonal violence (physical violence by other means than firearms). The six distinct categories evaluated in the present analyses were road injuries, other transport injuries, falls, animal contact, exposure to mechanical forces, and interpersonal violence.

Methods to estimate the incidence of and deaths due to each injury have been described in detail previously,^39^ with references for datasets available on the GBD 2021 Sources Tool. In summary, we utilised data from vital registration records, verbal autopsy, and police records for deaths, and from hospital and emergency or outpatient departments, insurance claims, and surveys that reported on injuries that warranted care for incidence. Death estimates were modelled with the Cause of Death Ensemble model tool,^40^ which selects an ensemble of mixed-effects or spatiotemporal Gaussian regression models of mortality rates or cause fractions. The hierarchical Bayesian meta-regression modelling tool, DisMod-MR 2.1, was used to estimate the incidence of each injury.^18^ Years of life lost (YLLs) were the product of the number of deaths and standard life expectancy at each age of death,^41^ and YLDs were the product of the disability weight corresponding to each ICD nature-of-injury code (N-code) and their probabilities of long-term and short-term impacts. DALYs were calculated as the sum of YLLs and YLDs.

There were two steps to estimate the fraction of fractures that were due to low bone mineral density for each injury. First, we estimated the fraction of each injury that resulted in a fracture. We calculated this across the six causes of injuries separately (by ICD external cause codes, E-codes) and then as a total. Briefly, we applied the ratio of fracture versus non-fracture injuries within each E-code. To derive these ratios, we used hospital and emergency department datasets with detailed diagnostic codes (ICD-9 and ICD-10) that were dual-coded with both the cause of injury (E-code; eg, falls or road injury) and nature of injury (N-code; eg, fracture or traumatic brain injury). We calculated the ratio of injuries attributable to fracture versus non-fracture injuries. These dual-coded data were available from 35 countries: Argentina, Brazil, Bulgaria, China, Chile, Colombia, Cyprus, Czech Republic, Denmark, Egypt, England, Estonia, Georgia, Hungary, Iceland, Iran, Italy, India, Kyrgyzstan, Latvia, Macedonia, Malta, Mauritius, Mexico, Mozambique, Netherlands, New Zealand, Norway, the Philippines, Portugal, Slovenia, Spain, Sweden, Uganda, the USA, and Zambia.^39^ Second, we applied this ratio to the population attributable fractions for fractures by E-code to quantify the magnitude of each burden measurement that resulted from low bone mineral density. We then aggregated the low bone mineral density-specific and total number of YLDs, DALYs, and deaths across E-codes to calculate the proportion of burden of each E-code that was due to low bone mineral density.

We observed that each cause of injury could result in multiple N-codes within each dataset, so we applied a series of logical and pragmatic decisions. First, we assumed that only hip fractures and some non-hip fractures, including vertebral, pelvic, and humeral fractures, were assumed to be potentially fatal. Second, we assigned the death to the fracture in the absence of a more severe injury code (N-code) that could better explain the death (ie, moderate to severe head trauma, spinal cord lesion, and intra-abdominal or thoracic organ damage).

For all measures, uncertainty was calculated by taking the final 100 draws from the posterior distribution. We report the 95% uncertainty intervals (UIs) as the 2·5th and 97·5th percentile values. Age-standardised rates and values were calculated with use of the GBD standard population.^42^ We provide detailed data on DALYs but not always on YLDs because, for low bone mineral density, YLDs are nearly equivalent to DALYs. This is due to the age structure of low bone mineral density, which primarily affects older adults who contribute few YLLs.

### Decomposition analysis

A Das Gupta decomposition analysis was done to determine the relative contributions of four factors to the change in absolute DALYs and deaths attributable to low bone mineral density between 1990 and 2020. These factors were population growth, population ageing, changes in exposure to low bone mineral density, and changes in risk-deleted injury (ie, injury-related DALY or death rate after removing the effect of low bone mineral density on overall rate).^43^

### Role of the funding source

The funder of the study had no role in study design, data collection, data analysis, data interpretation, or writing of the report.

## Results

The current analysis is based on 169 unique sources (including some sources reporting bone mineral density in multiple countries) representing 48 countries or territories and all seven GBD super-regions for mean bone mineral density, and on 12 sources representing nine countries and three super-regions for the meta-analysis establishing the relationship between low bone mineral density and risk of fracture (appendix 1 p 3).

**Figure 1:**
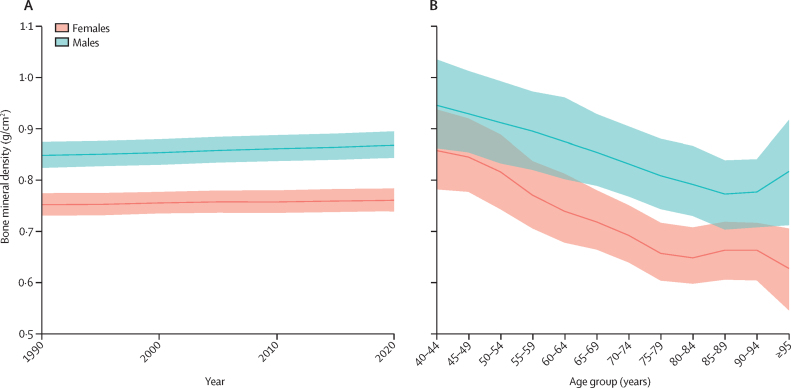
Bone mineral density values for the global population by sex (A) Age-standardised mean bone mineral density values from 1990 to 2020. (B) Mean bone mineral density values in 2020 by 5-year age-strata. Shaded regions represent 95% UIs.

In 2020, 8·32 million (95% UI 5·58–10·84) YLDs, 17·2 million (14·1–20·2) DALYs, and 477 000 (411 000–536 000) deaths were attributable to low bone mineral density. From 1990 to 2020, age-standardised mean bone mineral density values in the global population remained stable in men (0·85 g/cm^2^ [0·82–0·87] in 1990 vs 0·87 g/cm^2^ [0·84–0·89] in 2020) and women (0·75 g/cm^2^ [0·73–0·77] in 1990 vs 0·76 g/cm^2^ [0·74–0·78]) in 2020 (figure 1A). In 2020, mean bone mineral density was lower in women than in men across all age strata. In women, mean bone mineral density decreased from 0·86 g/cm^2^ (0·78–0·94) at age 40–44 years to 0·65 g/m^2^ (0·60–0·70) at age 80–84 years (figure 1B). The lowest mean bone mineral density was observed in women aged 95 years and older, although the 95% UIs widened due to the smaller size of this age group. Similarly, in men, mean bone mineral density decreased from 0·95 g/cm^2^ (0·86–1·04) at age 40–44 years to 0·79 g/cm^2^ (0·73–0·87) at age 85–89 years. Global, region, and country or territory level mean bone mineral density data in 2020, including change from 1990, by sex for all ages combined, are shown in appendix 1 (pp 4–7).

Between 1990 and 2020, global YLDs attributable to low bone mineral density increased by 91·8% (95% UI 88·5–95·1), DALYs increased by 89·8% (81·5–99·0), and deaths increased by 127·1% (108·5–144·5). After standardisation by age, global rates of YLDs, DALYs and deaths attributable to low bone mineral density showed modest decreases between 1990 and 2020 (–12·9% [–14·2 to –11·6] for YLDs, –12·7% [–16·5 to –8·6] for DALYs, and –4·0% [–10·9 to 3·5] for deaths; table).

**Table:** Absolute numbers and age-standardised rates of DALYs and deaths attributable to low bone mineral density in 2020, and percentage change in age-standardised rates from 1990 to 2020, globally and by super-region, region, and country or territory

		**Number of DALYs in 2020**	**Age-standardised rate of DALYs per 100** **000 in 2020**	**Percentage change in age-standardised rate of DALYs per 100** **000 from 1990 to 2020**	**Number of deaths in 2020**	**Age-standardised rate of deaths per 100** **000 in 2020**	**Percentage change in age-standardised rate of deaths per 100** **000 from 1990 to 2020**
**Global**		**17 200 000 (14 100 000–20 200 000)**	**207·6 (170·0–243·3)**	**−12·7 (−16·5 to −8·6)**	**477 000 (411 000–536 000)**	**6·0 (5·1–6·7)**	**−4·0 (−10·9 to 3·5)**
**Central Europe, eastern Europe, and central Asia**		**1 170 000 (878 000–1 460 000)**	**190·6 (142·3–236·0)**	**−29·9 (−32·8 to −27·6)**	**19 100 (16 200–21 200)**	**3·1 (2·6–3·4)**	**−37·9 (−40·7 to −34·9)**
Central Asia		97 300 (73 900–117 000)	123·7 (94·3–148·3)	−31·5 (−34·9 to −28·6)	1510 (1280–1690)	2·2 (1·9–2·5)	−32·6 (−37·5 to −26·7)
	Armenia	4100 (3270–4960)	102·2 (82·0–123·5)	−44·8 (−47·8 to −42·4)	78·8 (66·8–89·9)	2·0 (1·7–2·2)	−40·8 (−46·7 to −34·9)
	Azerbaijan	8160 (5920–10 000)	81·1 (59·3–98·7)	−41·7 (−46·8 to −37·2)	98·3 (77·4–119)	1·2 (0·9–1·5)	−53·6 (−62·8 to −43·4)
	Georgia	12 200 (9430–14 500)	212·7 (164·5–255·0)	8·9 (2·1 to 14·5)	287 (237–324)	4·5 (3·7–5·1)	31·6 (17·8 to 43·3)
	Kazakhstan	27 400 (21 400–33 200)	149·2 (116·0–180·3)	−23·8 (−30·2 to −18·2)	385 (321–449)	2·3 (1·9–2·6)	−34·2 (−43·3 to −24·7)
	Kyrgyzstan	6200 (5070–7430)	120·5 (97·2–143·7)	−38·7 (−43·6 to −33·4)	104 (86·7–123)	2·2 (1·8–2·5)	−39·6 (−47·7 to −29·9)
	Mongolia	5310 (4100–6510)	183·1 (141·5–222·7)	9·8 (−1·5 to 20·6)	79·2 (63·2–100)	3·1 (2·5–3·8)	−6·2 (−27·4 to 16·1)
	Tajikistan	5560 (4250–6850)	96·9 (74·2–118·1)	−41·1 (−46·5 to −35·2)	76·8 (59·8–93·3)	1·7 (1·3–2·1)	−38·8 (−50·7 to −23·6)
	Turkmenistan	3550 (2590–4450)	82·0 (59·8–102·2)	−37·2 (−44·1 to −31·6)	45·2 (35·9–55·3)	1·1 (0·9–1·3)	−49·0 (−57·6 to −37·9)
	Uzbekistan	24 800 (18 800–30 300)	104·0 (79·1–126·5)	−39·2 (−43·5 to −35·4)	357 (298–417)	2·0 (1·7–2·2)	−45·3 (−51·4 to −38·9)
Central Europe		431 000 (319 000–530 000)	204·8 (151·3–252·6)	−38·7 (−42·4 to −35·7)	8380 (7050–9410)	3·8 (3·2–4·2)	−53·5 (−56·9 to −50·7)
	Albania	6210 (4350–7930)	150·6 (105·2–192·8)	−22·3 (−27·4 to −17·9)	72·8 (58·5–90·2)	1·8 (1·4–2·2)	−26·6 (−39·1 to −9·3)
	Bosnia and Herzegovina	7760 (5380–9900)	135·7 (94·2–173·4)	−31·1 (−34·7 to −26·5)	83·2 (58·6–104)	1·5 (1·1–1·9)	−41·7 (−52·0 to −27·5)
	Bulgaria	24 400 (17 300–30 900)	182·4 (129·5–230·2)	−24·1 (−29·0 to −19·6)	333 (283–386)	2·4 (2·1–2·8)	−39·9 (−45·8 to −32·1)
	Croatia	24 300 (18 500–29 400)	267·1 (201·9–324·1)	−24·8 (−29·5 to −19·6)	627 (517–730)	6·5 (5·3–7·6)	−34·7 (−42·3 to −24·9)
	Czechia	47 500 (35 400–57 800)	224·5 (165·6–274·6)	−50·4 (−54·8 to −46·6)	1090 (921–1250)	4·8 (4·1–5·5)	−65·5 (−69·7 to −60·9)
	Hungary	44 400 (33 200–54 400)	227·1 (169·0–277·1)	−56·0 (−60·4 to −52·5)	997 (804–1170)	4·7 (3·8–5·6)	−72·3 (−76·0 to −68·9)
	Montenegro	1710 (1240–2120)	180·3 (130·4–224·7)	−12·3 (−19·1 to −5·2)	27·3 (21·6–33·2)	3·1 (2·5–3·8)	−4·9 (−22·8 to 25·4)
	North Macedonia	4800 (3410–6030)	161·4 (116·4–203·8)	14·5 (4·9 to 22·4)	75·4 (59·5–90·2)	3·3 (2·5–4·0)	72·1 (37·6 to 103·2)
	Poland	142 000 (107 000–177 000)	206·2 (154·6–256·2)	−35·8 (−39·6 to −32·2)	2900 (2340–3360)	3·9 (3·1–4·5)	−46·7 (−52·1 to −40·5)
	Romania	68 400 (48 900–87 100)	193·5 (138·2–246·3)	−34·2 (−38·9 to −30·5)	1030 (851–1190)	2·7 (2·3–3·2)	−41·2 (−48·4 to −34·9)
	Serbia	24 100 (17 900–30 200)	162·2 (121·7–203·6)	−25·5 (−31·0 to −20·0)	393 (315–473)	2·9 (2·3–3·5)	−35·5 (−45·7 to −22·4)
	Slovakia	20 900 (15 200–26 400)	229·9 (165·2–290·0)	−33·3 (−41·0 to −27·3)	361 (300–444)	4·0 (3·3–4·9)	−44·7 (−54·7 to −31·3)
	Slovenia	13 800 (10 300–16 700)	292·4 (215·9–355·5)	−29·4 (−34·1 to −25·3)	391 (303–453)	6·9 (5·4–8·0)	−20·2 (−30·8 to −10·8)
Eastern Europe		646 000 (480 000–812 000)	195·0 (144·8–244·4)	−23·3 (−26·3 to −20·8)	9170 (7580–10 300)	2·7 (2·3–3·1)	−24·3 (−29·4 to −19·4)
	Belarus	31 300 (23 000–39 300)	202·9 (148·9–254·4)	−9·8 (−14·7 to −4·5)	448 (374–520)	2·8 (2·4–3·3)	−8·1 (−17·0 to 3·1)
	Estonia	3860 (2770–4760)	155·1 (111·2–191·5)	−48·7 (−51·7 to −45·0)	68·9 (56·2–82·8)	2·4 (2·0–2·9)	−50·4 (−56·6 to −42·9)
	Latvia	6940 (5120–8430)	187·3 (137·0–226·8)	−50·4 (−53·7 to −47·3)	139 (116–165)	3·3 (2·8–4·0)	−50·9 (−56·2 to −44·3)
	Lithuania	11 500 (8440–14 000)	208·2 (152·1–254·1)	−33·5 (−37·9 to −30·1)	225 (184–263)	3·6 (3·0–4·2)	−26·6 (−34·9 to −18·4)
	Moldova	8250 (6220–10 300)	145·1 (109·2–181·0)	−46·7 (−50·1 to −43·6)	128 (104–149)	2·2 (1·8–2·6)	−50·3 (−56·0 to −45·0)
	Russia	455 000 (339 000–572 000)	200·4 (149·1–251·3)	−20·2 (−24·0 to −16·9)	6760 (5680–7660)	2·9 (2·5–3·4)	−20·6 (−26·6 to −14·0)
	Ukraine	130 000 (95 500–164 000)	180·5 (133·6–226·9)	−29·4 (−34·8 to −23·9)	1400 (1090–1720)	1·9 (1·5–2·4)	−37·4 (−47·9 to −25·3)
**High income**		**4 260 000 (3 210 000–5 270 000)**	**191·6 (143·1–236·7)**	**−15·7 (−17·8 to −14·1)**	**107 000 (86 600–121 000)**	**4·0 (3·3–4·5)**	**−12·8 (−17·6 to −9·9)**
Australasia		126 000 (93 400–158 000)	239·3 (175·3–301·6)	−3·1 (−5·6 to −0·7)	3040 (2470–3480)	5·0 (4·1–5·7)	23·7 (14·9 to 35·8)
	Australia	106 000 (78 900–133 000)	238·4 (175·3–300·5)	0·9 (−2·0 to 4·1)	2620 (2120–3010)	5·1 (4·1–5·8)	30·4 (19·7 to 44·4)
	New Zealand	20 000 (14 500–25 200)	242·7 (174·4–304·6)	−18·8 (−20·8 to −16·4)	424 (343–497)	4·5 (3·7–5·2)	−3·2 (−9·8 to 4·3)
High-income Asia Pacific		622 000 (454 000–788 000)	133·2 (96·1–167·4)	−32·7 (−36·0 to −30·7)	13 100 (10 300–15 300)	2·2 (1·8–2·5)	−32·3 (−39·8 to −27·3)
	Brunei	632 (477–756)	209·0 (158·6–246·0)	−30·1 (−36·9 to −23·8)	9·38 (8·01–10·6)	4·8 (4·1–5·6)	−26·0 (−38·0 to −8·7)
	Japan	426 000 (312 000–540 000)	113·8 (81·9–143·7)	−31·9 (−34·1 to −30·7)	9700 (7530–11 300)	1·8 (1·5–2·1)	−27·8 (−35·4 to −23·4)
	Singapore	9280 (6440–11 700)	114·3 (79·7–144·7)	−30·3 (−36·8 to −26·0)	104 (90–115)	1·4 (1·2–1·5)	−50·8 (−54·7 to −47·3)
	South Korea	186 000 (137 000–233 000)	203·9 (149·5–255·1)	−44·7 (−51·4 to −40·4)	3290 (2600–3890)	3·8 (3·0–4·5)	−50·3 (−65·2 to −40·1)
High-income North America		1 410 000 (1 070 000–1 710 000)	219·8 (167·4–267·5)	11·1 (7·6 to 14·1)	35 000 (29 000–39 400)	5·0 (4·2–5·6)	50·1 (42·4 to 55·2)
	Canada	152 000 (115 000–187 000)	204·2 (154·2–252·9)	−0·6 (−3·3 to 3·1)	4530 (3700–5280)	5·4 (4·5–6·3)	18·0 (9·8 to 26·5)
	Greenland	227 (181–278)	367·4 (295·7–442·5)	−16·7 (−25·8 to −8·5)	6·11 (4·32–7·61)	12·0 (8·2–15·1)	−10·2 (−28·8 to 10·8)
	USA	1 250 000 (956 000–1 520 000)	221·2 (168·7–268·6)	12·1 (8·4 to 15·6)	30 500 (25 300–34 200)	5·0 (4·2–5·5)	53·3 (45·2 to 58·3)
Southern Latin America		142 000 (108 000–173 000)	169·2 (128·4–205·9)	−16·3 (−19·7 to −13·7)	2710 (2280–2960)	3·1 (2·6–3·4)	−24·4 (−27·9 to −21·7)
	Argentina	84 200 (64 100–102 000)	155·6 (118·4–188·9)	−19·7 (−22·7 to −17·1)	1510 (1280–1660)	2·7 (2·3–3·0)	−30·9 (−34·8 to −27·8)
	Chile	47 500 (35 800–58 100)	194·4 (146·4–237·4)	−12·8 (−18·1 to −8·5)	942 (785–1060)	3·9 (3·2–4·3)	−19·6 (−24·5 to −13·9)
	Uruguay	10 500 (8090–12 900)	193·0 (146·9–236·8)	−11·5 (−14·0 to −9·0)	250 (209–280)	3·9 (3·3–4·3)	−3·7 (−8·5 to 2·8)
Western Europe		1 970 000 (1 470 000–2 430 000)	200·0 (148·2–247·9)	−21·1 (−23·9 to −19·4)	52 700 (42 300–60 500)	4·4 (3·5–5·0)	−24·8 (−29·1 to −21·8)
	Andorra	460 (332–577)	301·6 (218·0–375·7)	4·4 (−8·8 to 13·4)	7·64 (5·81–9·75)	4·5 (3·5–5·8)	−19·7 (−39·2 to 4·8)
	Austria	43 000 (32 400–52 800)	225·3 (167·4–277·5)	−26·9 (−29·8 to −24·7)	1140 (930–1310)	5·0 (4·1–5·7)	−28·8 (−34·0 to −24·3)
	Belgium	68 600 (50 700–84 800)	279·2 (203·6–346·8)	1·2 (−1·8 to 4·5)	1640 (1350–1890)	5·4 (4·5–6·2)	−2·7 (−8·8 to 4·9)
	Cyprus	4290 (3200–5260)	233·3 (174·9–285·4)	−45·3 (−52·1 to −39·8)	97·9 (76·3–120)	6·6 (4·7–8·0)	−64·1 (−73·4 to −55·6)
	Denmark	21 100 (15 600–25 900)	176·7 (130·7–216·7)	−43·0 (−46·3 to −40·2)	540 (431–615)	4·0 (3·2–4·5)	−55·3 (−58·8 to −52·3)
	Finland	33 900 (24 900–41 900)	263·8 (191·8–324·8)	−22·3 (−26·4 to −19·2)	789 (656–913)	5·1 (4·3–5·9)	−30·0 (−35·1 to −25·0)
	France	371 000 (273 000–463 000)	242·7 (177·9–303·9)	−25·8 (−30·3 to −22·6)	10 400 (8100–12 200)	5·1 (4·0–5·9)	−41·1 (−45·9 to −37·6)
	Germany	427 000 (323 000–523 000)	206·7 (154·1–253·9)	−15·6 (−18·7 to −13·2)	12 400 (9910–14 600)	5·1 (4·1–6·0)	−13·1 (−20·8 to −7·6)
	Greece	36 800 (27 700–45 200)	156·7 (116·1–192·5)	−30·2 (−32·1 to −28·7)	909 (757–1020)	2·9 (2·4–3·2)	−19·1 (−24·7 to −14·7)
	Iceland	1120 (844–1370)	188·4 (140·1–230·2)	−14·1 (−17·6 to −10·6)	31·4 (24·7–37·3)	4·4 (3·5–5·2)	5·6 (−5·7 to 17·0)
	Ireland	12 400 (8980–15 600)	162·1 (116·9–203·0)	−24·4 (−28·9 to −21·0)	217 (176–252)	2·7 (2·2–3·1)	−45·0 (−49·6 to −40·9)
	Israel	15 900 (11 600–19 700)	132·3 (96·3–163·3)	−21·9 (−26·3 to −18·9)	322 (262–371)	2·4 (1·9–2·7)	−35·4 (−39·8 to −31·5)
	Italy	302 000 (227 000–373 000)	194·6 (143·7–240·7)	−34·5 (−36·6 to −32·8)	7960 (6440–9170)	4·0 (3·2–4·5)	−41·8 (−44·6 to −40·0)
	Luxembourg	2450 (1830–3030)	224·9 (167·1–277·5)	−21·9 (−25·2 to −18·4)	63·1 (52·1–71·8)	5·0 (4·2–5·7)	−17·2 (−24·3 to −9·2)
	Malta	1690 (1230–2110)	178·6 (128·2–222·0)	−21·5 (−24·7 to −18·6)	37 (29·8–42·4)	3·4 (2·8–3·9)	−32·5 (−37·3 to −26·8)
	Monaco	135 (96·1–171)	141·2 (98·8–175·5)	−8·4 (−14·2 to −2·3)	2·57 (1·94–3·22)	2·1 (1·6–2·6)	−14·4 (−30·2 to 17·0)
	Netherlands	87 600 (67 800–106 000)	236·9 (181·4–288·6)	24·2 (19·6 to 28·9)	3170 (2520–3690)	7·9 (6·3–9·1)	52·3 (42·0 to 65·1)
	Norway	24 200 (18 300–29 500)	227·0 (169·8–278·3)	−28·8 (−31·7 to −26·8)	786 (623–910)	6·3 (5·1–7·3)	−19·5 (−25·9 to −15·5)
	Portugal	37 800 (28 500–46 400)	153·1 (114·9–187·6)	−40·7 (−43·3 to −38·0)	981 (794–1120)	3·3 (2·7–3·7)	−36·8 (−42·2 to −32·5)
	San Marino	107 (76·2–134)	160·4 (113·1–201·0)	−10·6 (−17·8 to −3·6)	2·04 (1·52–2·61)	2·4 (1·8–3·1)	−14·7 (−31·1 to 8·8)
	Spain	158 000 (114 000–196 000)	158·1 (113·2–196·4)	−17·3 (−22·8 to −13·3)	2890 (2350–3340)	2·3 (1·9–2·6)	−26·8 (−32·0 to −21·7)
	Sweden	44 100 (33 200–54 100)	191·8 (142·2–237·2)	−17·4 (−19·5 to −15·2)	1230 (1030–1420)	4·5 (3·7–5·1)	−8·0 (−13·2 to −3·2)
	Switzerland	48 100 (35 700–60 100)	247·8 (181·9–308·7)	−32·9 (−35·7 to −30·9)	1270 (1000–1500)	5·3 (4·2–6·2)	−41·7 (−47·3 to −37·0)
	UK	224 000 (167 000–278 000)	169·8 (125·5–211·3)	−1·6 (−3·1 to 0·2)	5800 (4720–6540)	3·8 (3·1–4·3)	25·9 (20·7 to 29·5)
**Latin America and Caribbean**		**1 070 000 (861 000–1 230 000)**	**177·7 (142·6–204·1)**	**−26·3 (−28·6 to −23·9)**	**27 600 (23 100–30 700)**	**4·7 (3·9–5·3)**	**−23·6 (−27·2 to −20·1)**
Andean Latin America		93 300 (75 300–110 000)	159·2 (128·6–187·8)	−10·8 (−19·4 to −0·7)	2640 (2170–3170)	4·7 (3·9–5·6)	−6·0 (−19·9 to 8·6)
	Bolivia	16 300 (12 500–20 400)	180·3 (139·6–225·7)	−22·2 (−34·4 to −7·0)	448 (346–577)	5·6 (4·4–7·2)	−22·7 (−37·8 to −3·3)
	Ecuador	28 700 (22 800–34 200)	187·1 (148·5–221·8)	−10·6 (−20·3 to 1·1)	824 (637–1000)	6·1 (4·7–7·3)	0·5 (−15·1 to 19·1)
	Peru	48 300 (38 700–57 800)	142·4 (114·0–170·7)	−6·2 (−17·4 to 9·6)	1370 (1080–1690)	4·0 (3·2–5·0)	−0·6 (−19·5 to 22·9)
Caribbean		93 800 (75 200–109 000)	176·9 (142·1–206·2)	−4·9 (−11·1 to 0·5)	3320 (2720–3810)	6·2 (5·1–7·1)	−5·9 (−13·1 to 0·8)
	Antigua and Barbuda	100 (77–122)	98·5 (76·8–120·3)	−3·2 (−8·9 to 3·6)	2·34 (1·98–2·66)	2·7 (2·3–3·0)	14·9 (5·1 to 26·9)
	The Bahamas	506 (410–616)	126·1 (102·5–152·4)	−13·3 (−23·5 to 1·0)	13·9 (11·5–16·8)	3·9 (3·2–4·8)	−3·3 (−19·2 to 17·0)
	Barbados	452 (357–551)	93·5 (73·6–113·8)	−2·0 (−10·6 to 8·5)	14·5 (11·6–17·7)	3·0 (2·4–3·6)	5·8 (−7·6 to 20·5)
	Belize	414 (330–485)	136·5 (108·8–160·5)	10·5 (3·1 to 18·1)	10·0 (8·45–11·5)	3·7 (3·1–4·3)	14·1 (1·3 to 24·7)
	Bermuda	115 (88·4–138)	88·8 (68·6–106·7)	−35·4 (−42·2 to −29·3)	3·55 (2·99–4·27)	2·5 (2·0–3·0)	−41·6 (−48·0 to −33·1)
	Cuba	48 200 (39 200–56 100)	236·9 (192·7–275·0)	0·3 (−6·3 to 7·8)	2070 (1710–2400)	9·5 (7·9–11·0)	2·6 (−6·9 to 13·5)
	Dominica	98·7 (78·2–117)	111·2 (88·2–131·7)	−4·0 (−15·7 to 7·2)	3·13 (2·49–3·81)	3·5 (2·8–4·2)	1·6 (−19·9 to 20·7)
	Dominican Republic	13 000 (9770–15 200)	133·7 (100·8–157·7)	−6·3 (−21·0 to 7·6)	326 (240–402)	3·6 (2·7–4·5)	−22·1 (−42·3 to −5·1)
	Grenada	153 (126–180)	144·1 (118·3–168·8)	13·4 (5·6 to 20·6)	4·93 (4·28–5·62)	5·5 (4·7–6·3)	39·2 (25·5 to 52·9)
	Guyana	1200 (942–1420)	196·6 (155·3–229·5)	1·1 (−10·0 to 16·6)	35·0 (28·1–42·7)	7·2 (5·8–8·5)	5·0 (−12·5 to 24·7)
	Haiti	12 400 (9150–15 900)	170·2 (127·8–215·2)	−16·8 (−32·6 to 1·4)	334 (241–428)	6·0 (4·5–7·7)	−11·5 (−33·4 to 12·0)
	Jamaica	2500 (1910–3070)	79·9 (61·1–98·4)	13·8 (4·8 to 24·8)	69·8 (55·3–84·9)	2·0 (1·6–2·5)	37·9 (15·8 to 67·2)
	Puerto Rico	8090 (6170–9690)	113·1 (86·1–135·8)	−17·2 (−24·4 to −11·6)	221 (178–254)	2·6 (2·1–3·0)	−37·2 (−44·6 to −29·1)
	Saint Kitts and Nevis	78·4 (61·5–95·1)	124·0 (98·9–149·6)	−14·9 (−21·7 to −8·9)	1·97 (1·64–2·26)	3·9 (3·3–4·5)	−13·4 (−21·5 to −3·9)
	Saint Lucia	228 (180–272)	103·3 (81·6–122·9)	−29·2 (−35·9 to −22·0)	5·92 (4·85–6·89)	2·9 (2·3–3·3)	−37·7 (−46·5 to −27·8)
	Saint Vincent and the Grenadines	162 (130–191)	122·8 (99·5–144·4)	5·4 (−0·7 to 13·3)	5·09 (4·41–5·73)	4·4 (3·8–4·9)	19·3 (10·0 to 31·3)
	Suriname	861 (685–998)	139·9 (111·0–161·7)	−8·2 (−16·6 to 1·5)	25·1 (21·1–29·7)	4·4 (3·6–5·2)	−9·5 (−22·4 to 5·3)
	Trinidad and Tobago	1890 (1490–2270)	103·0 (81·4–123·7)	−23·6 (−31·3 to −15·1)	53·7 (42·7–63·9)	3·0 (2·4–3·6)	−35·1 (−44·3 to −23·6)
	Virgin Islands	211 (171–251)	125·5 (100·7–150·4)	−11·5 (−21·3 to −0·6)	5·97 (4·99–7·17)	3·8 (3·1–4·6)	−25·9 (−38·9 to −11·7)
Central Latin America		385 000 (298 000–450 000)	156·7 (121·5–183·1)	−39·9 (−43·1 to −37·1)	8510 (7390–9550)	3·6 (3·1–4·0)	−46·4 (−49·9 to −42·4)
	Colombia	66 200 (51 000–78 900)	120·8 (93·0–144·1)	−40·5 (−44·8 to −36·5)	1530 (1260–1810)	2·7 (2·2–3·2)	−44·7 (−51·1 to −38·4)
	Costa Rica	8870 (6870–10 500)	167·3 (129·9–198·3)	−25·4 (−30·9 to −20·0)	259 (209–297)	4·8 (3·9–5·5)	−30·5 (−39·0 to −21·9)
	El Salvador	11 600 (9290–13 600)	188·1 (151·2–221·5)	−19·8 (−27·4 to −13·3)	320 (264–376)	5·0 (4·1–5·7)	−23·2 (−34·3 to −11·4)
	Guatemala	21 000 (16 600–24 700)	183·1 (145·1–215·1)	−19·2 (−25·2 to −13·5)	436 (375–493)	4·4 (3·7–4·9)	−41·4 (−47·9 to −36·3)
	Honduras	11 600 (9190–13 900)	182·2 (145·3–214·3)	−3·8 (−14·8 to 10·8)	301 (248–362)	5·4 (4·6–6·4)	6·2 (−11·3 to 27·0)
	Mexico	203 000 (158 000–239 000)	167·7 (130·8–197·5)	−47·0 (−50·1 to −44·5)	4250 (3530–4830)	3·8 (3·1–4·3)	−54·8 (−58·1 to −50·6)
	Nicaragua	6310 (4970–7360)	145·1 (114·3–169·5)	−18·0 (−24·2 to −10·4)	149 (121–171)	4·1 (3·4–4·9)	−16·2 (−28·8 to −1·4)
	Panama	4570 (3500–5480)	105·0 (80·5–126·1)	−38·4 (−43·9 to −33·3)	96·7 (79·2–111)	2·2 (1·8–2·5)	−48·4 (−54·4 to −41·5)
	Venezuela	52 000 (39 800–63 700)	170·2 (130·3–208·1)	−21·2 (−30·2 to −9·6)	1160 (910–1440)	4·0 (3·1–4·9)	−26·3 (−39·1 to −9·5)
Tropical Latin America		503 000 (411 000–582 000)	202·2 (165·8–234·3)	−19·8 (−22·5 to −17·9)	13 100 (10 800–14 700)	5·5 (4·6–6·2)	−6·4 (−12·4 to −2·5)
	Brazil	493 000 (403 000–572 000)	202·9 (166·2–235·6)	−20·4 (−23·0 to −18·4)	12 900 (10 600–14 400)	5·6 (4·6–6·2)	−7·3 (−13·1 to −3·3)
	Paraguay	9810 (7750–11 900)	169·4 (134·4–205·4)	15·0 (0·1 to 35·3)	255 (189–323)	4·6 (3·4–5·8)	43·4 (5·5 to 86·3)
**North Africa and Middle East**		**792 000 (652 000–913 000)**	**168·6 (139·1–193·8)**	**−24·2 (−28·3 to −20·3)**	**18 100 (15 800–20 400)**	**4·6 (4·1–5·2)**	**−26·1 (−31·5 to −19·3)**
	Afghanistan	39 900 (31 800–50 200)	266·7 (212·5–327·1)	3·0 (−14·6 to 33·6)	1110 (837–1370)	10·7 (7·5–13·4)	4·1 (−13·8 to 33·4)
	Algeria	57 600 (46 200–69 800)	161·7 (129·8–195·0)	−31·1 (−38·8 to −21·3)	1290 (1060–1580)	4·4 (3·6–5·3)	−25·2 (−37·7 to −8·1)
	Bahrain	1240 (964–1470)	102·8 (82·0–121·3)	−36·8 (−44·2 to −30·2)	20·3 (16·4–23·5)	3·4 (2·8–4·0)	−40·8 (−50·3 to −31·5)
	Egypt	74 600 (56 200–87 000)	110·7 (85·1–130·6)	−22·6 (−28·6 to −16·5)	1270 (1020–1570)	2·4 (1·9–3·1)	−24·9 (−38·2 to −13·2)
	Iran	131 000 (109 000–152 000)	165·5 (136·4–192·5)	−42·2 (−45·8 to −38·6)	3050 (2670–3500)	4·3 (3·7–5·0)	−41·8 (−46·7 to −36·6)
	Iraq	43 100 (33 600–52 500)	158·3 (124·5–188·8)	−24·3 (−36·1 to −10·8)	869 (683–1110)	3·8 (3·0–4·6)	−16·4 (−36·1 to 6·6)
	Jordan	7650 (6170–9070)	103·1 (82·8–122·2)	−40·9 (−49·4 to −32·9)	154 (124–186)	2·8 (2·2–3·4)	−46·7 (−57·2 to −35·4)
	Kuwait	4050 (3100–4830)	123·4 (95·9–145·9)	−33·5 (−39·6 to −27·7)	88·7 (71·7–104)	3·8 (3·0–4·4)	−23·1 (−32·4 to −12·9)
	Lebanon	5950 (4800–7020)	113·1 (91·3–133·3)	−45·5 (−51·9 to −37·6)	174 (146–206)	3·4 (2·9–4·0)	−57·6 (−66·2 to −39·0)
	Libya	14 600 (11 700–18 100)	234·8 (185·7–296·9)	4·3 (−17·3 to 39·4)	361 (268–494)	6·6 (4·9–9·4)	12·2 (−22·0 to 77·4)
	Morocco	55 900 (41 500–68 900)	173·8 (130·2–213·3)	−22·3 (−32·1 to −9·5)	1250 (919–1580)	4·5 (3·3–5·7)	−17·8 (−33·7 to 5·3)
	Oman	5600 (4560–6530)	269·5 (220·5–312·0)	−47·3 (−57·3 to −34·5)	123 (99·8–147)	9·3 (7·5–10·9)	−40·6 (−52·9 to −20·5)
	Palestine	2700 (2150–3170)	113·9 (92·0–133·2)	−25·0 (−35·6 to −16·2)	55·7 (47·9–64·4)	3·2 (2·7–3·6)	−36·3 (−48·2 to −24·7)
	Qatar	2820 (2130–3530)	193·8 (148·6–237·5)	−37·1 (−49·7 to −22·9)	51·2 (38·7–66·5)	6·8 (5·1–8·5)	−50·8 (−62·4 to −36·3)
	Saudi Arabia	99 800 (78 500–120 000)	385·4 (304·4–459·4)	−13·2 (−26·3 to 2·1)	1680 (1300–2100)	7·8 (6·2–9·3)	−23·9 (−40·0 to −1·5)
	Sudan	37 300 (29 400–45 100)	184·7 (147·3–221·8)	−28·5 (−38·7 to −14·1)	972 (765–1220)	5·7 (4·5–7·1)	−29·0 (−42·0 to −8·9)
	Syria	15 400 (11 900–19 200)	120·2 (94·6–147·7)	−15·0 (−27·8 to 1·1)	314 (239–396)	3·2 (2·5–3·8)	−15·2 (−34·7 to 9·0)
	Tunisia	20 300 (16 400–25 800)	155·4 (126·4–197·7)	−23·3 (−34·9 to −6·1)	476 (346–638)	4·0 (2·9–5·4)	−28·6 (−47·0 to −3·9)
	Türkiye	118 000 (95 800–140 000)	133·4 (108·1–159·0)	−17·9 (−27·4 to −5·5)	3630 (2860–4440)	4·4 (3·4–5·4)	−24·3 (−39·3 to −0·1)
	United Arab Emirates	12 300 (9290–15 200)	184·3 (143·1–224·4)	−41·1 (−52·5 to −27·9)	159 (122–209)	5·3 (4·2–6·6)	−47·3 (−59·6 to −30·5)
	Yemen	40 800 (31 300–51 700)	261·1 (204·3–324·4)	−26·2 (−43·7 to −6·5)	1010 (762–1320)	7·8 (6·0–10·0)	−23·5 (−44·3 to 2·2)
**South Asia**		**4 120 000 (3 510 000–4 620 000)**	**312·3 (266·5–350·2)**	**1·9 (−6·5 to 9·7)**	**140 000 (119 000–157 000)**	**12·5 (10·6–14·1)**	**6·3 (−8·6 to 19·9)**
	Bangladesh	112 000 (90 400–142 000)	83·9 (67·2–106·6)	−5·8 (−21·3 to 8·0)	3110 (2280–4330)	2·7 (2·0–3·8)	−6·0 (−25·2 to 17·8)
	Bhutan	1430 (1060–1830)	274·2 (202·6–351·1)	−13·2 (−33·1 to 9·9)	54·6 (35·1–76·2)	12·1 (7·6–17·0)	−18·1 (−39·3 to 15·3)
	India	3 780 000 (3 220 000–4 260 000)	354·6 (302·5–399·4)	1·0 (−7·3 to 9·5)	129 000 (110 000–146 000)	14·2 (12·0–16·2)	5·8 (−9·2 to 19·9)
	Nepal	46 500 (34 700–61 300)	229·6 (172·0–305·9)	2·0 (−12·8 to 18·4)	1330 (959–2080)	8·6 (6·2–13·1)	4·7 (−19·3 to 36·3)
	Pakistan	178 000 (142 000–213 000)	162·3 (127·6–195·2)	−8·9 (−23·6 to 7·5)	5810 (4500–7130)	7·1 (5·3–8·8)	−18·5 (−36·5 to 3·3)
**Southeast Asia, east Asia, and Oceania**		**4 760 000 (3 960 000–5 700 000)**	**178·2 (146·8–214·4)**	**−8·3 (−21·8 to 4·8)**	**131 000 (99 600–160 000)**	**5·5 (4·1–6·8)**	**−2·6 (−28·3 to 19·2)**
East Asia		3 650 000 (3 020 000–4 440 000)	177·2 (146·0–216·4)	−3·2 (−22·8 to 15·4)	98 000 (71 700–123 000)	5·3 (3·8–6·7)	9·6 (−31·5 to 44·3)
	China	3 540 000 (2 920 000–4 320 000)	178·8 (146·8–219·1)	−2·0 (−22·4 to 17·4)	94 900 (68 600–120 000)	5·3 (3·8–6·8)	11·3 (−31·5 to 48·0)
	North Korea	55 500 (43 800–69 200)	168·2 (134·3–209·0)	6·7 (−13·7 to 28·1)	1590 (1280–2090)	5·3 (4·3–6·9)	20·0 (−8·8 to 55·6)
	Taiwan (province of China)	45 900 (36 500–52 500)	113·3 (90·1–129·4)	−53·3 (−57·5 to −49·5)	1520 (1210–1730)	3·6 (2·9–4·1)	−45·6 (−52·5 to −39·3)
Oceania		13 100 (10 800–15 800)	187·2 (152·5–225·0)	−0·8 (−13·1 to 13·2)	291 (223–376)	5·3 (4·0–7·0)	−9·7 (−27·8 to 16·6)
	American Samoa	54·7 (43·8–62·9)	120·5 (96·8–137·9)	−8·3 (−19·0 to 3·9)	1·56 (1·28–1·88)	4·1 (3·3–5·0)	−5·5 (−25·5 to 20·4)
	Cook Islands	29·4 (23·5–34·6)	120·6 (96·3–142·1)	−28·8 (−39·0 to −16·9)	0·778 (0·619–0·966)	3·3 (2·6–4·1)	−38·1 (−50·3 to −22·0)
	Federated States of Micronesia	114 (88·9–141)	176·5 (137·2–214·3)	3·0 (−14·2 to 29·8)	3·02 (2·29–3·79)	6·5 (5·0–8·0)	11·7 (−11·8 to 49·4)
	Fiji	672 (531–804)	97·9 (78·5–117·1)	−9·9 (−21·3 to 6·0)	17·9 (13·9–20·8)	3·5 (2·8–4·2)	−0·9 (−21·6 to 24·7)
	Guam	189 (151–221)	98·7 (79·0–115·0)	−23·6 (−31·8 to −16·8)	5·76 (4·79–6·72)	3·0 (2·5–3·5)	−33·2 (−47·0 to −19·1)
	Kiribati	57·1 (44·4–69·1)	84·0 (67·4–99·8)	5·8 (−9·5 to 24·0)	1·19 (0·91–1·49)	2·5 (1·9–3·1)	28·4 (−0·4 to 63·2)
	Marshall Islands	53·9 (40·9–65·7)	161·7 (125·4–193·2)	1·9 (−12·4 to 16·6)	1·33 (1·03–1·67)	5·9 (4·5–7·2)	7·1 (−12·5 to 31·2)
	Nauru	10·5 (7·72–13·4)	208·6 (158·2–260·2)	−17·7 (−31·9 to 2·0)	0·220 (0·157–0·294)	6·4 (4·7–8·9)	−36·3 (−50·0 to −14·9)
	Niue	2·69 (2·09–3·35)	126·0 (97·8–157·5)	−4·9 (−18·7 to 11·2)	0·0875 (0·0685–0·112)	4·2 (3·3–5·3)	−1·9 (−27·2 to 28·4)
	Northern Mariana Islands	77·4 (60·2–93·7)	161·1 (126·6–192·7)	−12·2 (−25·8 to 2·2)	1·74 (1·46–2·16)	5·0 (4·0–6·0)	−14·2 (−31·8 to 7·9)
	Palau	44·5 (36·2–53·3)	242·4 (204·5–282·6)	−6·3 (−19·3 to 8·7)	1·21 (0·99–1·48)	9·7 (8·0–12·0)	−0·3 (−21·2 to 25·5)
	Papua New Guinea	9910 (7970–12 200)	211·9 (170·1–260·1)	−3·8 (−20·1 to 15·1)	214 (154–287)	5·8 (4·0–8·2)	−13·5 (−37·9 to 21·8)
	Samoa	211 (165–250)	146·7 (116·2–172·9)	4·5 (−10·4 to 23·0)	6·19 (4·99–7·53)	4·9 (3·9–6·0)	4·2 (−20·4 to 31·3)
	Solomon Islands	758 (589–956)	242·1 (191·5–298·2)	−4·7 (−20·5 to 16·5)	13·8 (10·3–18·1)	5·9 (4·3–7·9)	−15·2 (−35·4 to 15·8)
	Tokelau	1·62 (1·25–2·03)	123·8 (96·7–153·5)	−24·8 (−35·2 to −9·9)	0·0498 (0·0374–0·0667)	4·2 (3·2–5·5)	−37·0 (−50·8 to −16·7)
	Tonga	79·9 (63·0–95·5)	99·8 (79·1–119·3)	−12·0 (−25·1 to 5·1)	2·64 (2·06–3·34)	3·4 (2·7–4·3)	−8·6 (−31·4 to 19·7)
	Tuvalu	15·6 (12·8–18·5)	162·5 (132·7–192·8)	−13·5 (−23·2 to −0·2)	0·451 (0·343–0·556)	5·6 (4·1–6·9)	−16·6 (−32·9 to 4·6)
	Vanuatu	252 (195–301)	145·8 (113·6–174·2)	−2·9 (−19·0 to 17·5)	6·72 (5·06–8·15)	5·0 (3·7–6·0)	1·2 (−19·8 to 30·9)
Southeast Asia		1 100 000 (922 000–1 250 000)	183·8 (151·4–208·5)	−21·0 (−26·9 to −15·1)	32 800 (26 800–37 800)	6·4 (5·0–7·5)	−21·2 (−31·7 to −11·0)
	Cambodia	31 900 (25 700–38 200)	288·8 (234·3–343·1)	11·1 (−4·9 to 27·8)	1060 (810–1290)	11·8 (8·8–14·5)	16·9 (−6·9 to 41·9)
	Indonesia	351 000 (289 000–411 000)	173·1 (141·3–204·5)	−25·6 (−35·8 to −17·0)	10 300 (8270–12 200)	6·5 (4·9–7·8)	−14·6 (−32·1 to 1·4)
	Laos	7130 (5790–8730)	161·7 (131·6–196·0)	−19·6 (−34·2 to 2·1)	202 (161–251)	5·7 (4·6–7·0)	−15·1 (−32·6 to 16·4)
	Malaysia	48 100 (39 400–56 300)	176·6 (145·2–206·8)	−10·1 (−20·4 to 3·0)	1370 (1120–1630)	5·6 (4·6–6·7)	−8·0 (−22·9 to 8·7)
	Maldives	302 (237–365)	101·1 (79·7–122·4)	−25·5 (−36·5 to −12·0)	7·35 (6·05–9·21)	3·0 (2·3–3·7)	−27·0 (−43·0 to 1·8)
	Mauritius	1750 (1410–2040)	98·4 (79·1–114·2)	−8·3 (−17·4 to 0·3)	41·8 (34·6–48·3)	2·4 (2·0–2·8)	−7·0 (−19·0 to 5·2)
	Myanmar	87 000 (72 500–107 000)	194·8 (160·3–233·0)	−23·3 (−35·0 to −8·6)	2630 (2170–3230)	7·0 (5·6–8·5)	−24·5 (−41·1 to −3·5)
	Philippines	99 700 (82 200–116 000)	126·7 (103·8–146·0)	−4·4 (−14·3 to 4·3)	2580 (2150–2970)	4·0 (3·2–4·6)	7·0 (−12·3 to 26·0)
	Seychelles	129 (103–148)	109·1 (87·1–125·0)	−15·5 (−23·4 to −0·7)	3·03 (2·51–3·65)	2·8 (2·4–3·4)	−18·2 (−30·2 to 8·4)
	Sri Lanka	40 600 (32 400–51 200)	163·8 (130·5–205·6)	−20·6 (−33·7 to −7·1)	983 (717–1240)	4·4 (3·3–5·4)	−42·5 (−58·4 to −25·5)
	Thailand	171 000 (140 000–200 000)	161·4 (131·7–189·4)	−30·4 (−40·1 to −20·1)	4240 (3290–5300)	4·1 (3·1–5·1)	−41·6 (−53·5 to −26·5)
	Timor-Leste	1160 (953–1430)	148·0 (122·5–181·2)	3·1 (−16·0 to 27·1)	34·9 (27·2–44·2)	5·3 (4·1–6·6)	9·2 (−18·0 to 43·6)
	Viet Nam	264 000 (210 000–318 000)	292·0 (225·8–351·8)	−2·1 (−17·7 to 14·6)	9340 (5950–12 300)	11·8 (7·0–16·1)	−1·8 (−24·4 to 27·5)
**Sub-Saharan Africa**		**1 040 000 (907 000–1 160 000)**	**224·8 (197·9–250·5)**	**−9·5 (−18·2 to −1·6)**	**35 300 (31 300–39 500)**	**9·8 (8·6–10·9)**	**−2·2 (−12·8 to 8·2)**
Central sub-Saharan Africa		151 000 (125 000–181 000)	260·1 (215·7–311·0)	−3·0 (−19·4 to 15·9)	4460 (3460–5440)	10·1 (7·9–12·8)	3·2 (−17·4 to 24·1)
	Angola	29 400 (23 200–36 400)	242·7 (199·2–291·9)	−18·3 (−34·9 to 3·7)	847 (667–1040)	9·7 (8·0–11·8)	−12·7 (−30·0 to 10·1)
	Central African Republic	8730 (6780–11 300)	338·6 (266·3–425·1)	−7·3 (−24·6 to 15·0)	238 (182–309)	12·5 (9·8–15·7)	−5·1 (−23·1 to 14·2)
	Congo (Brazzaville)	7210 (5410–9050)	241·2 (187·7–293·6)	−25·6 (−41·7 to −6·6)	209 (157–264)	9·4 (7·1–11·9)	−21·8 (−38·2 to −3·1)
	DR Congo	102 000 (80 300–127 000)	263·7 (208·9–324·8)	6·0 (−16·9 to 29·0)	3050 (2230–3830)	10·1 (7·5–13·5)	13·1 (−14·5 to 40·4)
	Equatorial Guinea	1080 (791–1430)	209·0 (155·0–266·9)	−33·1 (−50·9 to −9·0)	33·1 (23·3–44·2)	8·2 (5·9–11·0)	−28·4 (−50·0 to 0·7)
	Gabon	2650 (2080–3280)	240·1 (191·2–293·7)	−12·5 (−28·2 to 10·9)	83·4 (64·6–105)	9·3 (7·2–11·9)	−4·5 (−22·8 to 23·1)
Eastern sub-Saharan Africa		366 000 (318 000–409 000)	237·7 (207·1–265·6)	−14·6 (−23·5 to −6·1)	13 600 (12 000–15 200)	11·4 (10·0–12·7)	−8·4 (−18·8 to 3·3)
	Burundi	10 900 (8860–13 400)	250·9 (202·4–305·5)	−22·5 (−39·4 to −1·5)	379 (296–473)	11·7 (9·2–14·7)	−20·7 (−39·3 to 1·0)
	Comoros	1170 (931–1410)	243·4 (193·5–295·5)	−23·5 (−37·7 to −4·6)	47·8 (36·7–57·6)	11·3 (8·8–14·0)	−24·6 (−40·1 to −4·8)
	Djibouti	1310 (1030–1680)	226·4 (182·2–282·8)	4·2 (−20·1 to 30·1)	42·8 (33·6–55·7)	10·7 (8·4–13·7)	13·1 (−15·2 to 46·3)
	Eritrea	7640 (5890–9580)	287·8 (225·1–348·2)	3·2 (−13·0 to 23·0)	243 (182–302)	13·0 (10·0–15·5)	2·9 (−16·0 to 21·3)
	Ethiopia	88 900 (76 200–100 000)	231·1 (200·0–258·9)	−39·2 (−50·1 to −29·2)	3590 (3120–3970)	11·7 (9·9–13·1)	−32·1 (−45·0 to −17·6)
	Kenya	48 700 (41 000–55 900)	243·3 (208·1–281·1)	11·6 (−5·4 to 33·2)	1820 (1540–2160)	12·1 (10·2–14·2)	15·8 (−6·8 to 45·2)
	Madagascar	18 800 (15 300–23 200)	177·6 (144·7–215·0)	−7·7 (−23·8 to 14·2)	602 (488–752)	8·0 (6·4–9·8)	−3·5 (−23·8 to 22·4)
	Malawi	17 700 (14 800–20 600)	257·6 (216·2–298·6)	9·0 (−9·2 to 26·2)	685 (577–790)	12·6 (10·2–14·6)	18·3 (−3·2 to 40·9)
	Mozambique	31 400 (24 000–40 400)	294·7 (226·0–375·8)	16·2 (−6·2 to 44·0)	1110 (837–1480)	13·4 (9·9–18·1)	22·0 (−6·4 to 60·4)
	Rwanda	14 200 (11 400–17 300)	253·3 (206·6–309·6)	−33·3 (−48·0 to −16·7)	526 (419–657)	12·2 (9·5–15·7)	−28·9 (−45·4 to −8·7)
	Somalia	21 700 (16 300–27 700)	318·3 (246·6–389·3)	21·9 (−2·5 to 48·2)	687 (505–862)	14·3 (11·1–18·0)	36·9 (7·2 to 64·6)
	South Sudan	9760 (7610–12 300)	251·6 (198·6–312·5)	14·1 (−11·8 to 40·7)	352 (273–442)	11·7 (9·1–14·8)	18·5 (−8·1 to 47·4)
	Tanzania	46 900 (37 900–57 100)	201·0 (163·7–242·6)	−15·3 (−26·0 to 1·2)	1770 (1440–2180)	9·5 (7·7–11·8)	−14·2 (−26·9 to 4·5)
	Uganda	30 100 (24 000–36 900)	224·6 (179·5–274·5)	−3·9 (−25·5 to 20·8)	1100 (876–1380)	10·5 (8·3–13·0)	−2·4 (−24·9 to 31·5)
	Zambia	17 000 (13 600–21 000)	262·0 (212·0–319·0)	−5·6 (−23·7 to 14·0)	597 (467–732)	12·4 (9·7–15·1)	−4·3 (−21·3 to 16·0)
Southern sub-Saharan Africa		113 000 (94 200–125 000)	177·0 (147·9–197·1)	−18·4 (−24·4 to −10·6)	2790 (2440–3060)	4·9 (4·3–5·4)	−8·7 (−19·2 to 6·1)
	Botswana	1900 (1590–2330)	123·3 (104·1–149·3)	−28·4 (−42·8 to −11·1)	41·9 (34·8–52·9)	3·4 (2·8–4·1)	−40·6 (−55·7 to −20·8)
	Eswatini	1710 (1280–2220)	246·9 (191·0–314·9)	14·9 (−8·9 to 56·7)	42·4 (31·6–56·4)	7·2 (5·6–9·5)	16·1 (−10·4 to 57·3)
	Lesotho	4360 (3450–5550)	302·4 (243·1–382·0)	58·1 (24·0 to 100·0)	112 (88·3–142)	8·9 (7·1–11·1)	58·6 (18·8 to 106·9)
	Namibia	2970 (2250–3820)	185·9 (142·7–234·4)	−7·3 (−26·3 to 11·8)	78·9 (59·7–105)	5·6 (4·4–7·2)	−6·6 (−27·2 to 14·9)
	South Africa	87 300 (74 100–97 900)	174·4 (147·8–195·2)	−24·4 (−30·3 to −17·5)	2110 (1830–2310)	4·6 (4·0–5·1)	−13·7 (−23·5 to 1·4)
	Zimbabwe	14 300 (10 900–17 500)	191·5 (147·6–231·9)	19·0 (−1·9 to 42·8)	407 (310–515)	6·9 (5·2–8·6)	35·3 (6·2 to 69·8)
Western sub-Saharan Africa		406 000 (354 000–459 000)	223·5 (194·7–249·5)	−4·7 (−14·5 to 4·9)	14 500 (12 700–16 300)	10·1 (8·8–11·4)	−0·4 (−10·8 to 12·5)
	Benin	10 800 (8650–13 200)	223·0 (183·0–268·6)	−11·4 (−24·0 to 3·3)	377 (309–450)	9·7 (8·1–11·6)	−12·8 (−25·4 to 5·8)
	Burkina Faso	27 300 (23 300–33 700)	306·0 (259·7–364·6)	−9·8 (−23·8 to 9·0)	983 (824–1210)	14·0 (11·6–17·0)	−11·4 (−24·8 to 10·8)
	Cabo Verde	545 (429–652)	123·3 (97·9–146·9)	12·1 (−5·6 to 34·1)	18·4 (14·7–24·5)	4·3 (3·4–5·7)	17·2 (−11·9 to 56·4)
	Cameroon	31 700 (25 000–39 100)	264·9 (212·7–324·7)	−10·3 (−26·3 to 15·6)	1070 (857–1350)	11·4 (9·3–14·4)	−12·0 (−30·0 to 15·9)
	Chad	13 300 (10 900–15 900)	241·7 (202·5–288·4)	15·8 (−3·3 to 48·0)	475 (391–578)	10·8 (8·9–12·9)	19·0 (−5·9 to 57·6)
	Côte d'Ivoire	24 400 (19 300–30 100)	233·3 (189·0–281·8)	−8·2 (−22·2 to 13·8)	808 (677–1000)	10·2 (8·6–12·4)	−7·8 (−24·8 to 14·4)
	The Gambia	2340 (1880–2850)	256·1 (207·6–311·3)	4·7 (−14·8 to 27·7)	94·2 (74·6–116)	12·3 (9·7–15·0)	6·7 (−15·1 to 33·3)
	Ghana	34 500 (27 300–41 700)	223·8 (179·2–267·4)	2·8 (−17·5 to 26·5)	1210 (940–1490)	10·2 (7·8–12·5)	8·6 (−16·2 to 40·4)
	Guinea	11 800 (9700–14 200)	221·6 (183·7–264·7)	7·5 (−15·5 to 36·4)	444 (363–537)	9·7 (8·2–11·8)	11·0 (−19·9 to 47·8)
	Guinea-Bissau	1990 (1550–2450)	270·6 (218·2–327·1)	−7·9 (−26·3 to 12·9)	61·5 (46·9–74·2)	11·2 (8·7–13·6)	1·8 (−18·3 to 24·8)
	Liberia	4310 (3460–5370)	208·5 (170·5–256·0)	6·2 (−16·4 to 31·9)	152 (122–194)	9·4 (7·6–11·8)	7·0 (−16·5 to 33·2)
	Mali	20 400 (16 500–25 000)	252·4 (203·7–306·0)	−8·9 (−23·5 to 7·2)	756 (595–912)	11·6 (9·0–13·8)	−0·7 (−20·1 to 20·1)
	Mauritania	4680 (3840–5740)	224·0 (184·3–272·0)	−23·5 (−36·9 to −2·1)	169 (134–206)	9·4 (7·4–11·5)	−17·7 (−32·6 to 8·0)
	Niger	16 200 (12 800–20 000)	226·4 (177·9–281·7)	−2·9 (−20·7 to 20·4)	543 (414–713)	10·0 (7·4–13·4)	−0·5 (−24·3 to 31·8)
	Nigeria	169 000 (143 000–201 000)	205·1 (175·1–239·7)	−7·0 (−20·9 to 9·9)	6200 (5190–7180)	9·5 (7·9–11·0)	−0·7 (−15·9 to 18·2)
	São Tomé and Príncipe	213 (181–256)	208·0 (176·8–244·9)	14·1 (0·5 to 31·3)	6·74 (5·54–8·17)	8·1 (6·5–10·2)	18·4 (−1·4 to 38·5)
	Senegal	15 800 (12 900–19 500)	220·9 (180·9–270·9)	−2·6 (−20·7 to 18·0)	601 (481–747)	10·0 (8·0–12·5)	−0·2 (−22·2 to 22·4)
	Sierra Leone	7410 (5920–9330)	210·2 (171·5–257·3)	2·9 (−14·7 to 25·4)	265 (209–321)	9·2 (7·4–11·0)	2·6 (−15·5 to 24·0)
	Togo	8630 (6930–10 800)	229·7 (188·8–278·8)	4·7 (−14·9 to 34·8)	266 (205–334)	9·6 (7·4–11·5)	7·9 (−16·7 to 42·3)

Numbers in parentheses are 95% uncertainty intervals. Rates are provided per 100 000 of the population. Values are rounded to three significant figures (absolute numbers) or one decimal place (rates and percentage changes). DALYs=disability-adjusted life-years.

**Figure 2:**
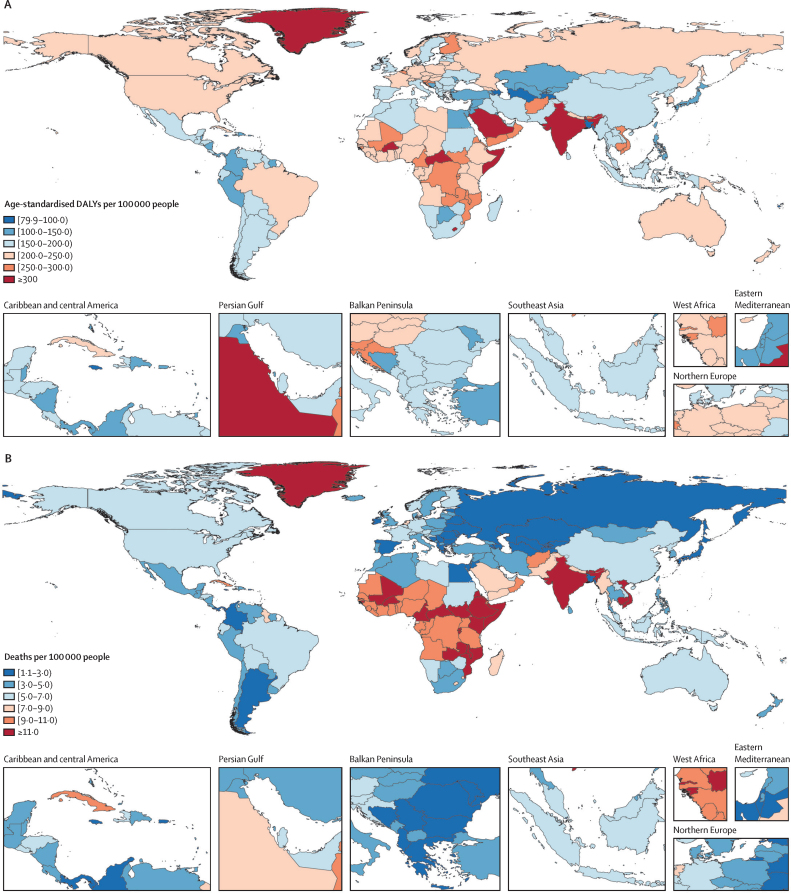
Map of age-standardised DALY rates (A) and death rates (B) for male and female sexes combined in 2020 Values are rounded to one decimal place. Rates are provided per 100 000 of the population. Intervals start at the lowest estimated rates (Jamaica for DALY rate and Turkmenistan for death rate; table). Areas with several small countries or details are magnified. DALYs=disability-adjusted life-years.

Figure 2 illustrates the geographical differences in the age-standardised rates of DALYs and deaths attributable to low bone mineral density in 2020. The absolute and age-standardised burden by super-region, region, and country or territory in 2020, and percentage change from 1990 to 2020, are shown in the table. At the region level, for DALYs, south Asia had the highest age-standardised rate in 2020 (312·3 [266·5–350·2] per 100 000 of the population) and central Asia had the lowest rate (123·7 [94·3–148·3] per 100 000). The region with the highest age-standardised mortality in 2020 attributed to low bone mineral density was south Asia (12·5 [10·6–14·1] deaths per 100 000), and the regions with the lowest rate were high-income Asia Pacific (2·2 [1·8–2·5]) per 100 000) and Central Asia (2·2 [1·9–2·5] per 100 000). With respect to YLDs, Australasia had the highest age-standardised rate of YLDs in 2020 (173·8 [121·2–240·6] per 100 000) and southern sub-Saharan Africa had the lowest rate (43·7 [31·7–58·4] per 100 000).

**Figure 3:**
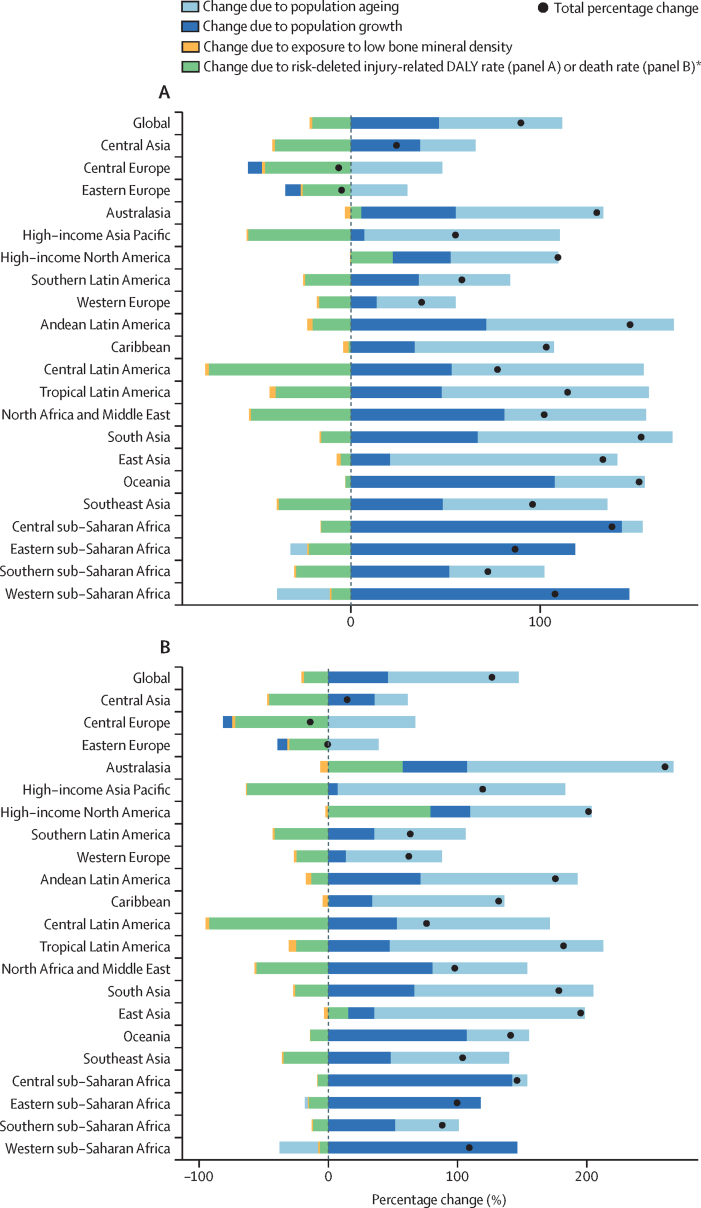
Decomposition of change in the number of DALYs (A) and deaths (B) attributable to low bone mineral density from 1990 to 2020 for male and female sexes combined, globally and by region Note that scales on x-axes differ between plots. DALYs=disability-adjusted life-years. *Injury-related DALY or death rate after removing the effect of low bone mineral density on the rate.

A decomposition analysis by region showed the relative contribution of population growth, population ageing, changes in exposure to low bone mineral density, and changes in risk-deleted injury-related DALY or death rates to the changes in absolute DALYs and deaths attributable to low bone mineral density from 1990 to 2020 (figure 3). Apart from population growth, which was the main driver of increases in DALYs and deaths in central Asia, north Africa and the Middle East, Oceania, and sub-Saharan Africa, population ageing was the main driver of increases in estimates in all other regions that showed an increase in estimates in 2020. In most regions that showed increases in DALYs and deaths, the increases were partially countered by reductions in injury-related DALY and death rates. In high-income North America and Australasia, increased injury-related DALY and deaths rates contributed to increases in absolute DALYs and deaths attributable to low bone mineral density. DALYs and deaths attributable to low bone mineral density showed a slight decrease or remained stable in eastern Europe and central Europe, primarily due to declines in injury-related DALY and death rates.

**Figure 4:**
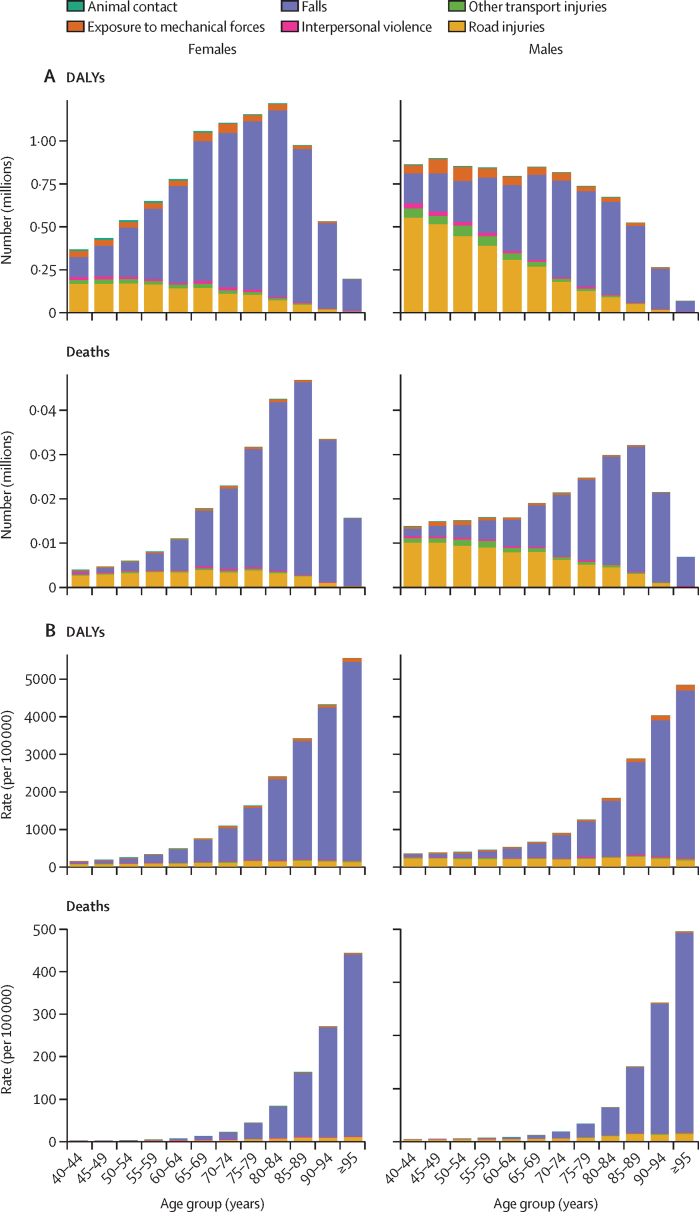
DALYs and deaths attributable to low bone mineral density in 2020 by sex, cause of injury, and 5-year age strata (A) Absolute number by cause of injury. (B) Rate per 100 000 of the population in each age group by cause of injury. Note that scales on y-axes differ between plots. DALYs=disability-adjusted life-years.

In 2020, falls and road injuries represented the first and second largest contributors, respectively, to the burden from fractures that was attributable to low bone mineral density (figure 4). The proportion of global burden attributable to low bone mineral density resulting from falls in 2020 was 76·2% (95% UI 74·2–78·3) for YLDs, 65·2% (62·9–67·6) for DALYs, and 71·0% (67·4–72·8) for deaths. In comparison, the proportion of global burden attributable to low bone mineral density resulting from road injuries in 2020 was 12·4% (11·1–13·6) for YLDs, 24·6% (22·5–27·1) for DALYs, and 23·1% (21·6–26·2) for deaths. appendix 1 (pp 8–13) presents the proportion of total fall-related burden and road injury-related burden attributable to low bone mineral density in 1990 and 2020, globally and by super-region, region, and country or territory. Globally, as a proportion of all fall-related burden, low bone mineral density accounted for 26·6% (23·2–28·7) of YLDs, 25·6% (22·1–27·4) of DALYs, and 40·6% (35·4–44·0) of deaths in 2020. Of all road injury-related burden, 12·6% (10·8–13·5) of YLDs, 6·3% (5·4–6·9) of DALYs, and 8·9% (7·6–9·6) of deaths were attributable to low bone mineral density. Compared with falls and road injuries, the relative burden from other causes was low and did not vary substantially over time (data not shown).

Counts of DALYs attributable to low bone mineral density due to falls were highest between age 70 and 84 years for men and women, and counts of fall-related deaths attributable to low bone mineral density were highest between age 80 and 94 years. In men, DALYs and deaths attributable to low bone mineral density due to road injuries were greatest at age 40–49 years and steadily decreased thereafter. In women, road injury-related DALYs were greatest at age 40–59 years and decreased thereafter, whereas road injury-related deaths were greatest at age 65–69 years and 75–79 years (figure 4). In men, road injuries accounted for the largest proportion of DALYs up to age 55–59 years, and deaths until age 60–64 years, after which falls became the primary driver of these outcomes. In women, road injuries were the leading cause of DALYs attributable to low bone mineral density in those aged 40–44 years, with the burden from falls predominating in older age groups. Road injuries were the primary driver of deaths attributable to low bone mineral density in women up to age 50–54 years, after which the burden from falls became the leading cause of attributable deaths.

## Discussion

This analysis updates and expands the evidence regarding the global burden attributable to low bone mineral density, an important modifiable risk factor for fractures related to injury. These data show an increase in burden related to low bone mineral density over time, with a nearly two-times increase in DALYs and YLDs and greater than two-times increase in deaths from 1990 to 2020. However, age-standardised rates showed slight decreases over this period, indicating that the numbers will continue to be driven by both population growth and population ageing. To our knowledge, this study highlights for the first time, the part that low bone mineral density plays in the global burden of fractures related to categories of injury other than falls, most notably road injuries.

We found that road injury-related deaths and disabilities constituted a substantial portion of burden associated with low bone mineral density, particularly in men. The data indicated that road injuries in women aged 40–54 years and men aged 40–64 years accounted for the largest proportion of deaths attributable to low bone mineral density, and also accounted for the largest proportion of DALYs attributable to low bone mineral density among women aged 40–44 years and men aged 40–59 years. Taken together, the data underscore the fact that the risk of death and disability attributable to low bone mineral density is not just an isolated problem among older women but also affects younger age groups and men.

South Asia was the region with the greatest burden attributable to low bone mineral density, with high overall rates of morbidity (DALYs) and mortality attributable to low bone mineral density (table). Although age-standardised rates of DALYs and deaths showed little change from 1990 to 2020 in this region, the rising absolute numbers of DALYs and deaths from fractures (figure 3) mean increasing costs to individuals and health systems.^44^, ^45^, ^46^, ^47^, ^48^ The Asian Federation of Osteoporosis Societies has projected that direct medical costs for hip fractures alone will escalate from US$9·5 billion in 2018 to $15 billion by 2050.^49^ Furthermore, infrastructure and capacity to adequately screen, diagnose, and manage low bone mineral density remain underdeveloped in many parts of Asia, with less than one DXA machine per million of the population in several countries or territories, and substantial gaps in health professional training and practice patterns.^50^ To meet this growing burden, strong collaborative efforts by regional osteoporosis societies have spurred the generation of locally relevant evidence and guidelines, multidisciplinary health professional education and capacity building programmes, including secondary prevention fracture-liaison programmes, and public awareness and advocacy initiatives.^48^

Sub-Saharan Africa also had increases in absolute DALYs and deaths attributable to low bone mineral density in the context of substantial population growth, including growth in the absolute number of older individuals in some regions. There is a notable lack of access to diagnostics, care, and treatments for osteoporosis across sub-Saharan Africa, and low prioritisation by health-care systems compared with communicable diseases or other non-communicable disease priorities.^51^, ^52^ Importantly, the WHO Essential Medicines List does not include any oral formulations of bisphosphonates,^53^ which are generally considered first-line agents for osteoporosis drug therapy, meaning in many low-income and middle-income countries (LMICs), including those in sub-Saharan Africa, availability might be limited. Intravenous zoledronic acid is on the Essential Medicines List, for the dosage recommended for adjuvant cancer treatment, but not for the formulation recommended for osteoporosis treatment.^53^ These gaps present an opportunity for advocacy and awareness. In sub-Saharan Africa, where long-standing priorities such as HIV, tuberculosis, and malnutrition continue to dominate the health agenda and international funding, health systems have difficulty managing the concurrent rise of non-communicable diseases and their risk factors.^54^ In this setting, there remains a paucity of high-quality regional epidemiological and health services data related to osteoporosis to inform evidence-based interventions.^52^

Although bone mineral density is a well established risk factor for fractures of all types, studies have consistently shown that the proportion of fractures attributable to osteoporosis alone, as defined by bone mineral density, is modest.^55^ Therefore, fracture prediction tools typically incorporate other clinical risk factors with or without bone mineral density to improve fracture prediction.^56^, ^57^ However, unlike risk factors such as age, sex, and family history of fracture, bone mineral density is potentially modifiable through a range of non-pharmacological primary prevention and lifestyle modification approaches (eg, dietary calcium, vitamin D and protein intake, sunlight exposure, weight-bearing physical activity, optimisation of underlying comorbidities, and smoking and alcohol cessation) and pharmacological interventions.^58^, ^59^ Furthermore, bone mineral density can be measured by standard techniques that are objective, reproducible, non-invasive, and fast.^13^ Evidence supports a correlation between change in bone mineral density with osteoporosis treatment and fracture risk reduction, albeit recognising that the full benefit derived from osteoporosis drug therapy goes beyond reductions in bone mineral density alone.^60^

Despite data supporting the screening, diagnosis, prevention, and management of osteoporosis, notable gaps exist between evidence-based guidelines and clinical practice, even in high-income regions where DXA imaging, osteoporosis treatments, and trained health professionals are available.^7^, ^61^ These gaps are compounded in low-income and middle-income regions, where fundamental barriers to accessing such resources exist.^62^ The critical role of policy and public health messaging in promoting best practices can be seen through key examples around the world. Crisp and colleagues reported a 20% and 13% decrease in age-standardised hip fracture incidence rates among men and women, respectively, in Australia between 1997–98 and 2006–07, a period during which there was active roll-out of public health initiatives focused on osteoporosis.^63^ Screening and treatment for low bone mineral density is reimbursed by the Australian Government for all men and women aged 50 years and older who have sustained a fracture, and for all individuals aged 70 years and older whether they’ve sustained a fracture or not. By contrast, in the USA, Hayes and colleagues described associations between cuts in Medicare reimbursements for DXA and decreased provision of physician's office-based DXA services and prescriptions for US Food and Drug Administration-approved osteoporosis therapies 2 years later.^64^ Age-adjusted hip fracture rates also plateaued in the USA from 2012 to 2015, following a decade of steady decreases, in the setting of decreased DXA testing and osteoporosis diagnosis, coupled with a reduction in bisphosphonate prescriptions due to fear of rare side-effects and misperceptions regarding the risk and benefits of treatment.^65^

The role of secondary prevention in osteoporosis also cannot be understated. Studies have shown that women aged 50 years and older with a history of fracture have as high as a four-times increase in the relative risk of future fractures compared to those without previous fractures.^66^ Nevertheless, rates of initiation of osteoporosis treatment following a fracture are poor across regions and practice settings.^67^ Fracture liaison services are coordinated multidisciplinary programmes aimed at identifying patients in the peri-hospitalisation period following a sentinel fracture, and linking patients to osteoporosis care.^68^, ^69^ Fracture liaison services have been shown to be effective at reducing both fracture rates and health-care costs compared with usual care or no care, and have been widely promoted by osteoporosis societies worldwide as the single most important step by health systems to prevent fracture-associated burden.^67^, ^70^

The promotion of strategies to prevent falls and road traffic injuries is additionally important. Falls prevention strategies include individual-level or practice or hospital-level interventions that might comprise multifactorial components such as falls risk screening, education, exercise, home modifications, medication changes, referral to other health-care services, and recommended use of assistive devices or aids.^71^, ^72^ The effectiveness of road traffic injuries prevention strategies across the domains of legislation (eg, seat belt, helmet, and cell phone usage laws and increased penalties), enforcement (eg, specific groups and individuals subject to enforcement and technology for enforcement), public awareness and education (eg, mass media campaigns against drunk driving), speed control (eg, speed bumps and other road designs to reduce speed), and road improvement (eg, repaving damaged roads) have been shown in high-income countries.^73^ Data from LMICs are comparatively limited. However, given rapid urbanisation and motorisation in these settings, more research and advocacy are needed to guide and strengthen road safety enforcement policies, speed control efforts, and public education campaigns.^74^, ^75^

Limitations associated with the comparative risk assessment methodology of the present analyses need to be acknowledged. In these analyses, uncertainty was estimated based on 100 draws where 1000 draws would have been preferable. The TMREL was based on US cohorts, which could lead to overestimation or underestimation of risk factor attribution in some world regions. For example, regions with lower average BMI compared with the USA might have lower age-specific and gender-specific bone mineral density, leading to higher attribution of fractures to low bone mineral density. Furthermore, the choice of a stringent TMREL (99th percentile) could have led to an overall overestimation of the burden of low bone mineral density.

The methodology to account for fracture-related deaths might also be susceptible to error in the estimation of true mortality attributable to low bone mineral density. We used hospital and emergency department data from 35 countries with double coding (ie, ICD codes for cause and nature of injury) to derive the proportion of in-hospital deaths attributable to the fracture event if a more severe life-threatening injury wasn’t listed, an approach subject to variations in coding practices between hospitals and countries. In addition, the excess risk of mortality remains elevated beyond the initial evaluation or hospitalisation period for osteoporotic fractures, not just for hip fractures, but for other types of fragility fractures as well (vertebral, pelvis, distal femur, proximal tibia, proximal humerus, and multiple ribs) and might not be assigned to the fracture as the underlying cause.^3^, ^76^, ^77^

Furthermore, the 12 studies used in the meta-analysis from which we derived the relative risks for hip and non-hip fractures were largely based in high-income countries.^25^, ^26^, ^27^, ^28^, ^29^, ^30^, ^31^, ^32^, ^33^, ^34^, ^35^, ^36^ These studies represented diverse regions and showed some variability; however, the relationship between bone mineral density and fracture risk remains to be explored across all populations. More high-quality epidemiological data across racially, ethnically, and socioeconomically diverse populations is needed to better inform future analyses. In addition, variations in modifiable risk factors for low bone mineral density, as well as variations in attributable risk of low bone mineral density related to the social disadvantage index and access to diagnosis and treatment, were beyond the scope of this paper but are important to consider for the future.

In summary, low bone mineral density is a key, modifiable risk factor for fractures related to injury. The present analysis highlights the contribution of low bone mineral density to DALYs and deaths resulting not only from falls in older adults, but also from fractures sustained in road injuries, particularly among middle-aged men. Although age-standardised rates of DALYs and deaths attributable to low bone mineral density decreased in many countries from 1990 to 2020, the increase in absolute numbers due to a growing and ageing global population places a substantial burden on health-care systems. LMICs are less equipped than high-income countries to handle this increasing demand and will thus face greater challenges. We advocate for the addition of oral bisphosphonates to the WHO Essential Medicines List, and for expansion of the eligibility for intravenous bisphosphonates on the Essential Medicines List to include management of low bone mineral density and individuals with high fracture risk aged 50 years and older. We also advocate for support for implementation strategies to improve the uptake of evidence-based injury and fracture prevention approaches, and recommend lifestyle strategies to improve bone health across the life-course.

### GBD 2021 Low Bone Mineral Density Collaborators

### Affiliations

### Contributors

### Data sharing

The findings of this study are supported by data available in public online repositories, data publicly available upon request of the data provider, and data not publicly available due to restrictions by the data provider. To download citations and metadata for the input data used in these analyses, please visit the Global Health Data Exchange GBD 2021 website (https://ghdx.healthdata.org/gbd-2021/sources). The data sources used in this analysis are listed in appendix 1 (pp 14–21).

Editorial note: The Lancet Group takes a neutral position with respect to territorial claims in published maps, tables, and institutional affiliations.

## Declaration of interests

S Bhaskar reports grants or contracts from the Japan Society for the Promotion of Science (JSPS), Japanese Ministry of Education, Culture, Sports, Science and Technology (for a Grant-in-Aid for Scientific Research, grant ID: 23KF0126) and from JSPS and the Australian Academy of Science for a JSPS International Fellowship (grant ID: P23712); and leadership or fiduciary roles in board, society, committee, or advocacy groups, paid or unpaid, with Rotary District 9675, Sydney, Australia, as the District Chair (Diversity, Equity & Inclusion), with the Global Health & Migration Hub Community, Global Health Hub Germany, Berlin, Germany (Chair, Founding Member and Manager), with *PLOS One*, *BMC Neurology*, *Frontiers in Neurology*, *Frontiers in Stroke*, *Frontiers in Public Health*, *Journal of Aging Research*, *Neurology International*, *Diagnostics*, and *BMC Medical Research Methodology* (Editorial Board Member), with the College of Reviewers, Canadian Institutes of Health Research, Government of Canada (Member), with the World Headache Society, Bengaluru, India (Director of Research), with the Cariplo Foundation, Milan, Italy (Expert Adviser/Reviewer), with the National Cerebral and Cardiovascular Center, Department of Neurology, Division of Cerebrovascular Medicine and Neurology, Suita, Osaka, Japan (Visiting Director), with Cardiff University Biobank, Cardiff, UK (Member, Scientific Review Committee), and with the Rotary Reconciliation Action Plan (Chair); all outside the submitted work. N E Ismail reports unpaid leadership or fiduciary roles in board, society, committee, or advocacy groups with the Malaysian Academy of Pharmacy (Bursar and Council Member) and the Malaysian Pharmacists Society (Committee Member of the Education Chapter), outside the submitted work. K Krishan reports non-financial support from the University Grants Commission Centre of Advanced Study—Phase II, awarded to the Department of Anthropology, Panjab University, Chandigarh, India, outside the submitted work. M Lee reports support for the present manuscript from the Ministry of Education of the Republic of Korea and the National Research Foundation of Korea (NRF-2023S1A3A2A05095298). L M March reports grants or contracts paid to their institution from the Australian National Health and Medical Research Council and the Australian Medical Research Future Fund; royalties from UpToDate for co-authorship of a review titled *Epidemiology and Risk Factors for Osteoarthritis*; and unpaid leadership or fiduciary roles in board, society, committee, or advocacy groups with the Global Alliance for Musculoskeletal Health as Executive; all outside the submitted work. L Monasta reports support for the present manuscript from the Italian Ministry of Health (Ricerca Corrente 34/2017) via payments made to the Institute for Maternal and Child Health IRCCS Burlo Garofolo. D Prieto-Alhambra reports support paid to their institution from the European Medicines Agency and the Innovative Medicines Initiative; grants or contracts from Amgen, Chiesi-Taylor, Gilead, Lilly, Janssen, Novartis, and UCB; consulting fees paid to their institution from UCB Biopharma; other support from Janssen for training programme's organised by the author's affiliated department; and a position on the Board of the European Health Data & Evidence Network Foundation; all outside the submitted work. Y L Samodra and J H V Ticoalu report leadership or fiduciary roles in board, society, committee, or advocacy groups, paid or unpaid, with Benang Merah Research Center, Indonesia, as Co-founders, outside the submitted work. L M L R Silva reports research contracts with the Sport Physical Activity and Health Research & Innovation Center–Instituto Politécnico da Guarda, Portugal, and RISE-Health, Universidade da Beira Interior, Portugal, outside the submitted work. J A Singh reports consulting fees from ROMTech, Atheneum, Clearview Healthcare Partners, the American College of Rheumatology, Yale University, Hulio, Horizon Pharmaceuticals, DINORA, ANI/Exeltis USA, Frictionless Solutions, Schipher, Crealta/Horizon, Medisys, Fidia, PK Med, Two Labs, Adept Field Solutions, Clinical Care Options, Putnam Associates, Focus Forward, Navigant Consulting, Spherix, MedIQ, Jupiter Life Science, UBM, Trio Health, Medscape, WebMD, Practice Point Communications, and the National Institutes of Health; payment or honoraria for lectures, presentations, speakers bureaus, manuscript writing, or educational events as a member of the speaker's bureau of Simply Speaking; leadership or fiduciary roles in other board, society, committee, or advocacy groups, paid or unpaid, and support for attending meetings or travel, as a past steering committee member of OMERACT; participation on a data safety monitoring board or advisory board from the US Food and Drug Administration Arthritis Advisory Committee; stock or stock options in Atai Life Sciences, Kintara Therapeutics, Intelligent Biosolutions, Acumen Pharmaceutical, TPT Global Tech, Vaxart Pharmaceuticals, Atyu Biopharma, Adaptimmune Therapeutics, GeoVax Labs, Pieris Pharmaceuticals, Enzolytics, Seres Therapeutics, Tonix Pharmaceuticals, Aebona Pharmaceuticals, and Charlotte's Web Holdings; and previous stock options in Amarin, Viking, and Moderna; all outside the submitted work. M Zielińska reports other financial or non-financial interests from Alexion, AstraZeneca Rare Disease as an employee outside the submitted work. All other authors declare no competing interests.
