# Supplementary material for: The global, regional, and national burden attributable to low bone mineral density, 1990–2020: an analysis of a modifiable risk factor from the Global Burden of Disease Study 2021
Source: Lancet Rheumatol. 2025 Sep 16;7(12):e873–94. doi: 10.1016/S2665-9913(25)00105-5 (PMC12623303; doi:10.1016/S2665-9913(25)00105-5)
Supplement: Supplementary appendix 1 [file mmc1.pdf]

THE LANCET
Rheumatology

Supplementary appendix 1

This appendix formed part of the original submission and has been peer reviewed. We post it as supplied by the authors.

Supplement to: GBD 2021 Low Bone Mineral Density Collaborators. The global, regional, and national burden attributable to low bone mineral density, 1990–2020: an analysis of a modifiable risk factor from the Global Burden of Disease Study 2021. *Lancet Rheumatol* 2025; published online Sept 16. https://doi.org/10.1016/S2665-9913(25)00105-5.

Supplementary Figure 1: Conceptual diagram for the BMD theoretical minimum risk exposure level (TMREL)*

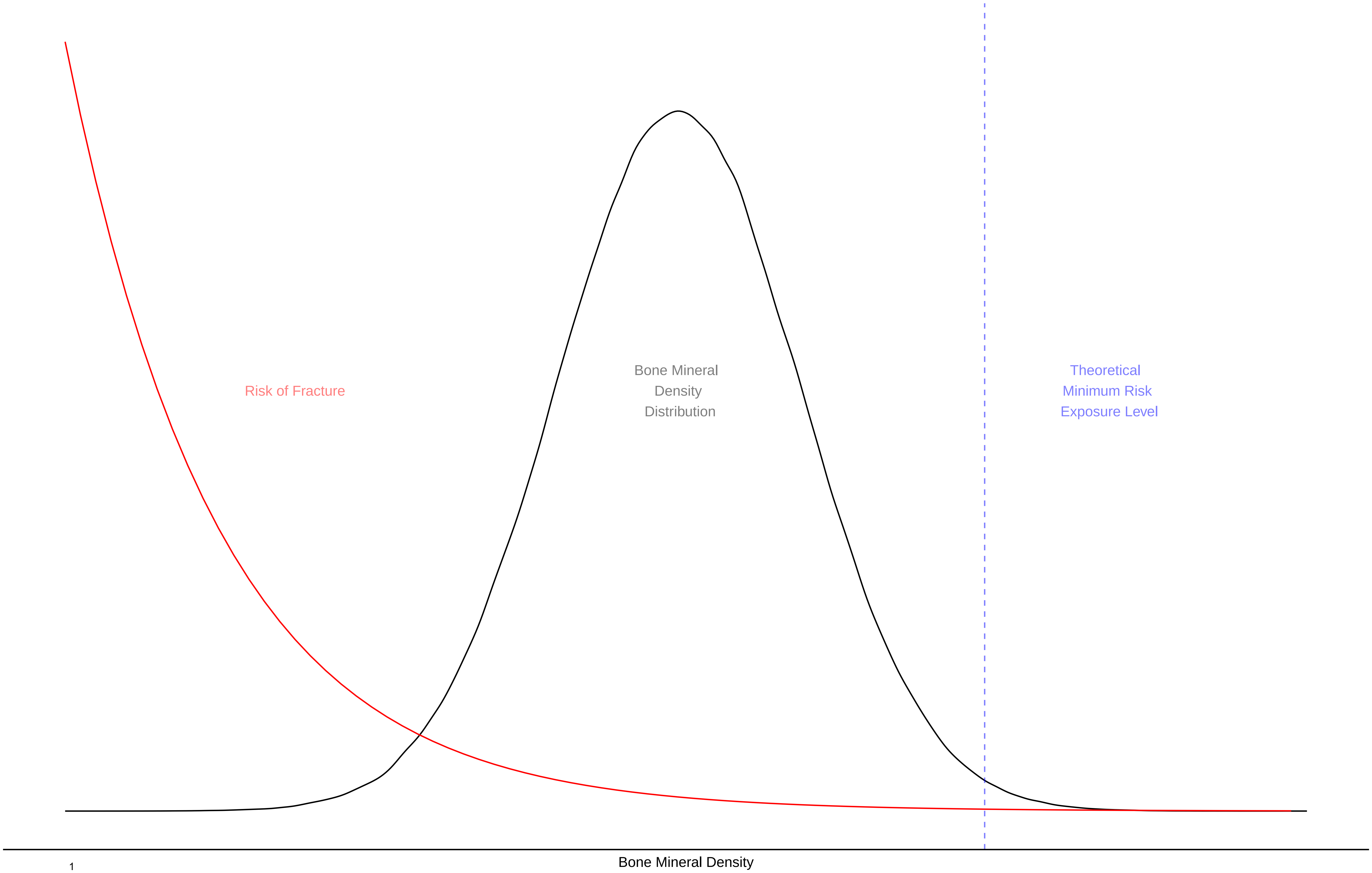

*In the case of low BMD as a risk factor, this means that the observed values in the population are compared to an ideal scenario where all individuals have an optimal BMD, irrespective of whether this is currently attainable in practice.

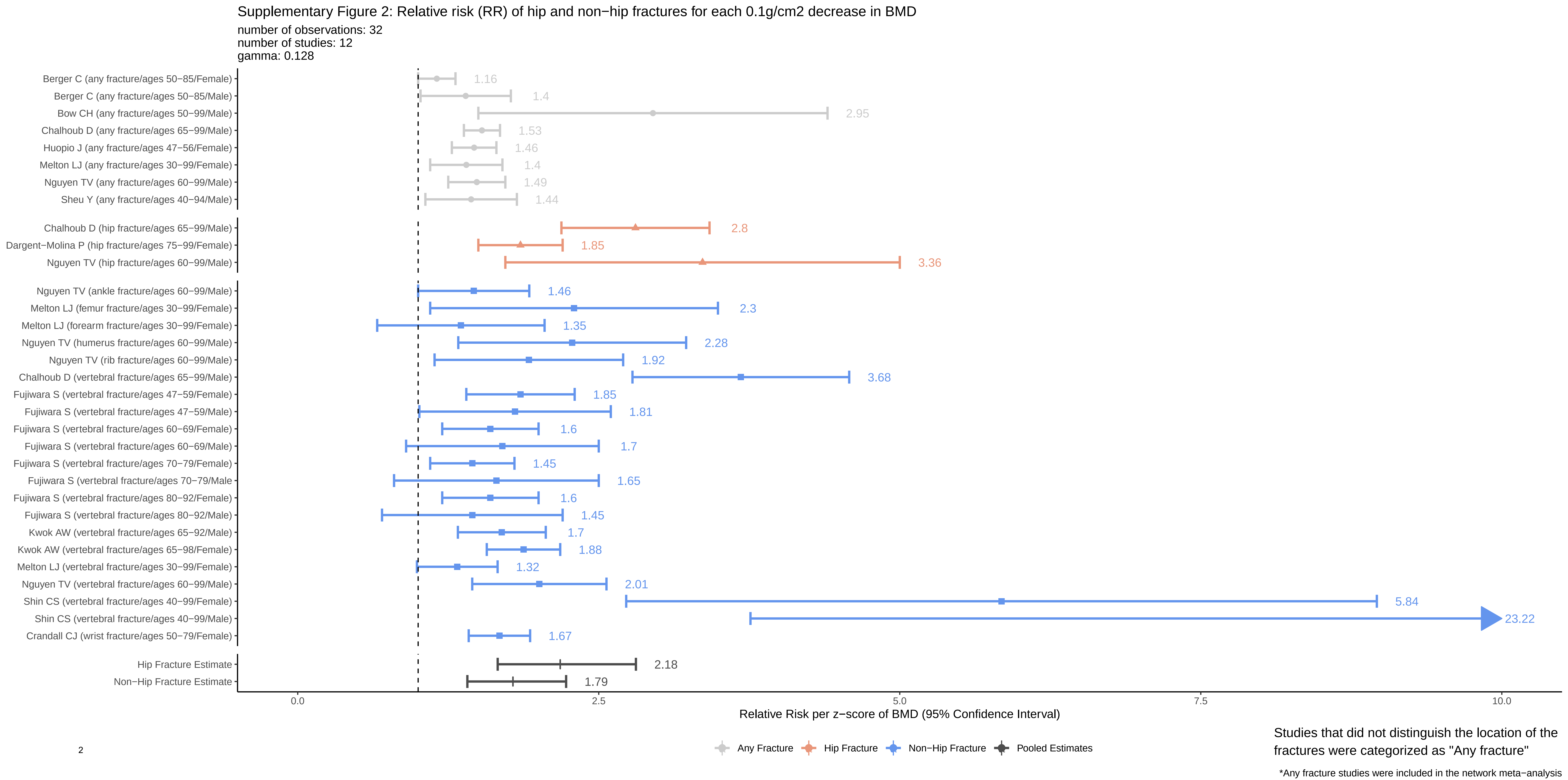

Supplementary Figure 2: Relative risk (RR) of hip and non−hip fractures for each 0.1g/cm2 decrease in BMD
number of observations: 32
number of studies: 12
gamma: 0.128
Berger C (any fracture/ages 50−85/Female)
1.16
Berger C (any fracture/ages 50−85/Male)
1.4
Bow CH (any fracture/ages 50−99/Male)
2.95
Chalhoub D (any fracture/ages 65−99/Male)
1.53
Huopio J (any fracture/ages 47−56/Female)
1.46
Melton LJ (any fracture/ages 30−99/Female)
1.4
Nguyen TV (any fracture/ages 60−99/Male)
1.49
Sheu Y (any fracture/ages 40−94/Male)
1.44
Chalhoub D (hip fracture/ages 65−99/Male)
2.8
Dargent−Molina P (hip fracture/ages 75−99/Female)
1.85
Nguyen TV (hip fracture/ages 60−99/Male)
3.36
Nguyen TV (ankle fracture/ages 60−99/Male)
1.46
Melton LJ (femur fracture/ages 30−99/Female)
2.3
Melton LJ (forearm fracture/ages 30−99/Female)
1.35
Nguyen TV (humerus fracture/ages 60−99/Male)
2.28
Nguyen TV (rib fracture/ages 60−99/Male)
1.92
Chalhoub D (vertebral fracture/ages 65−99/Male)
3.68
Fujiwara S (vertebral fracture/ages 47−59/Female)
1.85
Fujiwara S (vertebral fracture/ages 47−59/Male)
1.81
Fujiwara S (vertebral fracture/ages 60−69/Female)
1.6
Fujiwara S (vertebral fracture/ages 60−69/Male)
1.7
Fujiwara S (vertebral fracture/ages 70−79/Female)
1.45
Fujiwara S (vertebral fracture/ages 70−79/Male
1.65
Fujiwara S (vertebral fracture/ages 80−92/Female)
1.6
Fujiwara S (vertebral fracture/ages 80−92/Male)
1.45
Kwok AW (vertebral fracture/ages 65−92/Male)
1.7
Kwok AW (vertebral fracture/ages 65−98/Female)
1.88
Melton LJ (vertebral fracture/ages 30−99/Female)
1.32
Nguyen TV (vertebral fracture/ages 60−99/Male)
2.01
Shin CS (vertebral fracture/ages 40−99/Female)
5.84
Shin CS (vertebral fracture/ages 40−99/Male)
23.22
Crandall CJ (wrist fracture/ages 50−79/Female)
1.67
Hip Fracture Estimate
2.18
Non−Hip Fracture Estimate
1.79
0.0
2.5
5.0
7.5
10.0
Relative Risk per z−score of BMD (95% Confidence Interval)
Any Fracture
Hip Fracture
Non−Hip Fracture
Pooled Estimates
Studies that did not distinguish the location of the fractures were categorized as "Any fracture"
*Any fracture studies were included in the network meta−analysis

Supplemental Tables 1a and 1b. Number of data sources used for Exposure modeling and Relative Risk modeling, by country and territory

Exposure Data Sources

Country	Number of Sources
**Central Europe, Eastern Europe, and Central Asia**	
Bulgaria	2
Hungary	2
Poland	1
Romania	1
Slovakia	1
Lithuania	1
Russian Federation	1
Ukraine	1
**High-Income**	
Australia	6
Japan	11
Republic of Korea	3
Singapore	2
Canada	6
United States of America	20
Chile	1
Belgium	1
Denmark	2
Finland	6
France	4
Germany	2
Greece	1
Ireland	1
Italy	2
Netherlands	4
Norway	6
Portugal	2
Spain	5
Sweden	6
Switzerland	2
United Kingdom	11
**Latin America and Caribbean**	
Cuba	1
Trinidad and Tobago	1
Mexico	2
Brazil	4
**North Africa and Middle East**	
Iran (Islamic Republic of)	3
Jordan	1
Kuwait	2
Lebanon	3
Morocco	2
Qatar	2
Saudi Arabia	2
**South Asia**	
India	6
**Southeast Asia, East Asia, and Oceania**	
China	19
Taiwan (Province of China)	4
Sri Lanka	1
Thailand	6
**Sub-Saharan Africa**	
South Africa	2
Gambia	1
**TOTAL UNIQUE SOURCES***	**169**

** Some sources include data from multiple countries*

Relative Risk Data Sources

Country	Number of Sources
**Southeast Asia, East Asia, and Oceania**	
China	2
**High Income**	
Japan	1
Republic of Korea	1
Australia	1
Finland	1
France	1
Canada	1
United States of America	3
**Latin America and Caribbean**	
Trinidad and Tobago	1
**TOTAL UNIQUE SOURCES**	**12**

Supplemental Table 2. Mean Bone Mineral Density (g/cm^2) among men and women in 2020, and absolute and percent change in mean BMD from 1990-2020, by country						
	Men			Women		
**Location**	**2020 Mean BMD (g/cm2)**	**Change in mean BMD from 1990-2020**	**% Change in mean BMD from 1990-2020**	**2020 Mean BMD (g/cm2)**	**Change in mean BMD from 1990-2020**	**% Change in mean BMD from 1990-2020**
Global	0.877(0.851 - 0.904)	0.0317(0.0257 - 0.0378)	3.76% (3.03% - 4.49%)	0.769(0.746 - 0.793)	0.0194(0.014 - 0.025)	2.6% (1.87% - 3.33%)
Central Europe, eastern Europe, and central Asia	0.959(0.93 - 0.99)	0.0309(0.021 - 0.0406)	3.33% (2.25% - 4.45%)	0.809(0.787 - 0.833)	0.0232(0.0167 - 0.0298)	2.95% (2.11% - 3.81%)
Central Asia	0.941(0.911 - 0.973)	0.047(0.0308 - 0.0645)	5.27% (3.44% - 7.34%)	0.818(0.792 - 0.844)	0.0386(0.0255 - 0.0524)	4.98% (3.27% - 6.88%)
Armenia	0.944(0.909 - 0.981)	0.0528(0.0168 - 0.0871)	5.94% (1.85% - 9.89%)	0.818(0.789 - 0.849)	0.0409(0.0119 - 0.0717)	5.27% (1.49% - 9.43%)
Azerbaijan	0.954(0.915 - 0.992)	0.0589(0.0243 - 0.0934)	6.59% (2.67% - 10.6%)	0.828(0.797 - 0.861)	0.0461(0.0169 - 0.0759)	5.9% (2.14% - 9.87%)
Georgia	0.942(0.907 - 0.978)	0.0167(-0.0185 - 0.0514)	1.8% (-1.97% - 5.83%)	0.809(0.782 - 0.841)	0.00655(-0.0201 - 0.0364)	0.818% (-2.46% - 4.58%)
Kazakhstan	0.947(0.913 - 0.984)	0.0346(-0.000283 - 0.0676)	3.79% (-0.0306% - 7.52%)	0.822(0.787 - 0.854)	0.0304(0.00241 - 0.057)	3.84% (0.3% - 7.43%)
Kyrgyzstan	0.905(0.869 - 0.943)	0.0208(-0.0128 - 0.0554)	2.35% (-1.4% - 6.36%)	0.788(0.758 - 0.82)	0.0187(-0.00996 - 0.0467)	2.44% (-1.3% - 6.25%)
Mongolia	0.914(0.876 - 0.951)	0.0299(-0.00641 - 0.0687)	3.38% (-0.689% - 7.9%)	0.795(0.764 - 0.828)	0.0251(-0.00644 - 0.0541)	3.27% (-0.821% - 7.14%)
Tajikistan	0.895(0.859 - 0.934)	0.0513(0.0164 - 0.0865)	6.1% (1.94% - 10.5%)	0.78(0.748 - 0.81)	0.0438(0.0133 - 0.0734)	5.97% (1.74% - 10.1%)
Turkmenistan	0.956(0.917 - 0.997)	0.0842(0.0511 - 0.122)	9.65% (5.73% - 14.3%)	0.823(0.793 - 0.856)	0.061(0.0318 - 0.0914)	8% (4.11% - 12.2%)
Uzbekistan	0.955(0.918 - 0.998)	0.0638(0.028 - 0.101)	7.22% (3.15% - 11.3%)	0.831(0.8 - 0.865)	0.0537(0.0223 - 0.0832)	6.97% (2.84% - 10.9%)
Central Europe	1.01(0.969 - 1.04)	0.0359(0.0183 - 0.0536)	3.7% (1.85% - 5.64%)	0.801(0.779 - 0.825)	0.0244(0.0141 - 0.0344)	3.14% (1.78% - 4.45%)
Albania	0.985(0.944 - 1.03)	0.0536(0.0151 - 0.0896)	5.76% (1.57% - 10%)	0.809(0.777 - 0.84)	0.0402(0.0094 - 0.0732)	5.23% (1.18% - 9.61%)
Bosnia and Herzegovina	0.983(0.94 - 1.03)	0.0255(-0.0135 - 0.0642)	2.66% (-1.35% - 6.97%)	0.803(0.773 - 0.834)	0.0217(-0.00768 - 0.0514)	2.77% (-0.965% - 6.64%)
Bulgaria	0.985(0.944 - 1.03)	0.0164(-0.0211 - 0.0525)	1.69% (-2.13% - 5.52%)	0.797(0.768 - 0.825)	0.0119(-0.0167 - 0.0411)	1.51% (-2.09% - 5.32%)
Croatia	0.994(0.953 - 1.03)	0.0289(-0.0102 - 0.0681)	2.99% (-1.01% - 7.4%)	0.81(0.78 - 0.839)	0.0241(-0.00785 - 0.0525)	3.07% (-0.979% - 6.86%)
Czechia	1(0.96 - 1.04)	0.0286(-0.00984 - 0.0668)	2.94% (-0.988% - 7%)	0.816(0.786 - 0.848)	0.0241(-0.00331 - 0.0537)	3.04% (-0.408% - 6.84%)
Hungary	1.04(0.931 - 1.15)	0.0467(-0.0396 - 0.139)	4.69% (-3.97% - 15.3%)	0.808(0.781 - 0.839)	0.0192(-0.0158 - 0.05)	2.43% (-1.96% - 6.5%)
Montenegro	1(0.957 - 1.04)	0.0206(-0.0201 - 0.0577)	2.11% (-2.02% - 6.07%)	0.819(0.789 - 0.849)	0.0189(-0.0109 - 0.0474)	2.37% (-1.36% - 6.12%)
North Macedonia	0.989(0.945 - 1.03)	0.0424(0.00141 - 0.0815)	4.46% (0.149% - 8.81%)	0.811(0.78 - 0.843)	0.0348(0.00554 - 0.0637)	4.47% (0.704% - 8.3%)
Poland	1.01(0.983 - 1.05)	0.0413(0.027 - 0.0552)	4.25% (2.76% - 5.71%)	0.779(0.756 - 0.802)	0.0185(0.00817 - 0.0288)	2.44% (1.1% - 3.83%)
Romania	1.01(0.965 - 1.05)	0.0393(0.00113 - 0.075)	4.07% (0.114% - 8.04%)	0.807(0.78 - 0.834)	0.0395(0.0119 - 0.0686)	5.14% (1.54% - 9.09%)
Serbia	0.984(0.944 - 1.03)	0.0199(-0.0191 - 0.0598)	2.05% (-1.91% - 6.26%)	0.807(0.776 - 0.836)	0.0245(-0.00333 - 0.0525)	3.11% (-0.424% - 6.81%)
Slovakia	1.01(0.899 - 1.12)	0.0307(-0.0538 - 0.125)	3.14% (-5.1% - 13.7%)	0.873(0.841 - 0.904)	0.0318(5.2e-05 - 0.0642)	3.78% (0.00617% - 7.83%)
Slovenia	0.993(0.951 - 1.03)	0.0185(-0.0194 - 0.0591)	1.9% (-1.94% - 6.17%)	0.808(0.778 - 0.838)	0.0138(-0.0123 - 0.0421)	1.74% (-1.5% - 5.43%)
Eastern Europe	0.94(0.912 - 0.971)	0.026(0.015 - 0.036)	2.85% (1.61% - 4.01%)	0.81(0.787 - 0.834)	0.0173(0.00909 - 0.0254)	2.19% (1.14% - 3.23%)
Belarus	0.926(0.889 - 0.964)	0.0291(-0.00464 - 0.0648)	3.24% (-0.506% - 7.43%)	0.816(0.787 - 0.848)	0.013(-0.0175 - 0.0411)	1.62% (-2.15% - 5.22%)
Estonia	0.947(0.908 - 0.985)	0.0452(0.00971 - 0.0802)	5.01% (1.07% - 9.1%)	0.827(0.798 - 0.858)	0.0229(-0.00567 - 0.0506)	2.85% (-0.693% - 6.42%)
Latvia	0.945(0.907 - 0.983)	0.0315(-0.00482 - 0.0647)	3.45% (-0.512% - 7.22%)	0.828(0.799 - 0.859)	0.0127(-0.0186 - 0.0432)	1.56% (-2.23% - 5.42%)
Lithuania	0.953(0.916 - 0.991)	0.036(0.002 - 0.0729)	3.93% (0.212% - 8.24%)	0.863(0.831 - 0.895)	0.0169(-0.018 - 0.051)	2% (-2.13% - 6.06%)
Moldova	0.935(0.894 - 0.973)	0.054(0.0185 - 0.0902)	6.14% (2.08% - 10.5%)	0.824(0.791 - 0.856)	0.029(0.000538 - 0.058)	3.66% (0.067% - 7.49%)
Russia	0.94(0.912 - 0.97)	0.0245(0.0162 - 0.0332)	2.67% (1.78% - 3.64%)	0.82(0.797 - 0.845)	0.0212(0.0143 - 0.0284)	2.66% (1.75% - 3.54%)
Ukraine	0.945(0.912 - 0.982)	0.0267(-0.01 - 0.0591)	2.9% (-1.06% - 6.47%)	0.769(0.742 - 0.796)	0.00165(-0.0244 - 0.0267)	0.215% (-3.15% - 3.51%)
High income	0.918(0.894 - 0.944)	0.00959(0.00073 - 0.0184)	1.06% (0.0799% - 2.05%)	0.826(0.805 - 0.848)	-0.004(-0.0112 - 0.00339)	-0.482% (-1.35% - 0.41%)
Australasia	0.893(0.863 - 0.923)	0.0235(-0.00407 - 0.051)	2.7% (-0.455% - 5.88%)	0.848(0.818 - 0.878)	0.0198(-0.00769 - 0.0467)	2.39% (-0.917% - 5.66%)
Australia	0.893(0.863 - 0.927)	0.0224(-0.00941 - 0.0565)	2.57% (-1.06% - 6.59%)	0.849(0.818 - 0.881)	0.0178(-0.0144 - 0.0484)	2.14% (-1.73% - 5.86%)
New Zealand	0.888(0.859 - 0.92)	0.0288(0.00171 - 0.0546)	3.35% (0.192% - 6.43%)	0.841(0.812 - 0.871)	0.0293(0.00465 - 0.053)	3.61% (0.557% - 6.66%)
High-income Asia Pacific	0.91(0.89 - 0.93)	0.00634(-0.0107 - 0.0228)	0.702% (-1.16% - 2.6%)	0.797(0.779 - 0.814)	-0.00847(-0.022 - 0.00416)	-1.05% (-2.65% - 0.526%)
Brunei	0.921(0.883 - 0.96)	0.0505(0.0156 - 0.0847)	5.8% (1.75% - 10.2%)	0.824(0.79 - 0.857)	0.044(0.0127 - 0.0768)	5.64% (1.59% - 10%)
Japan	0.885(0.86 - 0.91)	-0.0117(-0.0232 - -0.000624)	-1.31% (-2.57% - -0.0705%)	0.777(0.755 - 0.799)	-0.0233(-0.0333 - -0.0139)	-2.92% (-4.11% - -1.72%)
South Korea	0.971(0.937 - 0.989)	0.0454(0.000527 - 0.0897)	4.91% (0.0555% - 10.1%)	0.846(0.822 - 0.857)	0.0252(-0.0115 - 0.0599)	3.08% (-1.34% - 7.6%)
Singapore	0.891(0.857 - 0.925)	0.00974(-0.0283 - 0.0465)	1.11% (-3.17% - 5.36%)	0.802(0.771 - 0.836)	0.00447(-0.0255 - 0.0369)	0.56% (-3.15% - 4.74%)
High-income North America	0.901(0.875 - 0.928)	0.00329(-0.00585 - 0.013)	0.366% (-0.665% - 1.44%)	0.816(0.793 - 0.841)	-0.0212(-0.029 - -0.0138)	-2.53% (-3.46% - -1.65%)
Canada	0.961(0.927 - 0.997)	0.0465(0.0105 - 0.084)	5.08% (1.13% - 9.33%)	0.858(0.83 - 0.892)	0.0341(0.00518 - 0.065)	4.13% (0.629% - 7.88%)
Greenland	0.924(0.886 - 0.964)	0.00308(-0.0353 - 0.0388)	0.334% (-3.76% - 4.18%)	0.841(0.809 - 0.873)	0.00352(-0.0298 - 0.0338)	0.42% (-3.47% - 4.12%)
USA	0.894(0.869 - 0.922)	-0.00154(-0.0105 - 0.00825)	-0.172% (-1.16% - 0.941%)	0.811(0.788 - 0.836)	-0.0273(-0.0352 - -0.0199)	-3.25% (-4.14% - -2.39%)
Southern Latin America	0.909(0.879 - 0.94)	0.0346(0.0122 - 0.0584)	3.96% (1.39% - 6.78%)	0.817(0.79 - 0.845)	0.0276(0.00772 - 0.0478)	3.5% (0.978% - 6.1%)
Argentina	0.905(0.869 - 0.94)	0.0321(-0.00171 - 0.0635)	3.68% (-0.192% - 7.45%)	0.813(0.783 - 0.845)	0.0263(0.000344 - 0.0542)	3.33% (0.0428% - 7.1%)
Chile	0.919(0.885 - 0.954)	0.0414(0.00545 - 0.0794)	4.71% (0.603% - 9.24%)	0.825(0.796 - 0.857)	0.0317(0.00339 - 0.0586)	3.99% (0.422% - 7.51%)
Uruguay	0.914(0.882 - 0.951)	0.0325(0.00206 - 0.0622)	3.68% (0.227% - 7.1%)	0.817(0.786 - 0.848)	0.025(-0.00381 - 0.0539)	3.15% (-0.473% - 6.92%)
Western Europe	0.939(0.913 - 0.967)	0.0139(0.00131 - 0.0272)	1.5% (0.143% - 2.94%)	0.847(0.824 - 0.87)	0.00575(-0.00499 - 0.0171)	0.684% (-0.594% - 2.03%)
Andorra	0.941(0.906 - 0.978)	0.000787(-0.0351 - 0.035)	0.0837% (-3.65% - 3.74%)	0.861(0.831 - 0.894)	-0.000504(-0.0317 - 0.0305)	-0.0585% (-3.57% - 3.63%)
Austria	0.905(0.872 - 0.939)	-0.00449(-0.0356 - 0.0282)	-0.494% (-3.87% - 3.05%)	0.82(0.79 - 0.85)	-0.00561(-0.0332 - 0.0257)	-0.679% (-3.96% - 3.15%)
Belgium	0.913(0.88 - 0.948)	0.0128(-0.0184 - 0.0492)	1.43% (-2.02% - 5.67%)	0.831(0.799 - 0.862)	0.00861(-0.0195 - 0.0377)	1.05% (-2.33% - 4.65%)
Cyprus	0.907(0.872 - 0.944)	0.0202(-0.0117 - 0.0569)	2.28% (-1.27% - 6.52%)	0.829(0.796 - 0.861)	0.0119(-0.0185 - 0.0435)	1.46% (-2.26% - 5.43%)
Denmark	0.933(0.9 - 0.967)	0.0154(-0.0183 - 0.0466)	1.68% (-1.94% - 5.17%)	0.882(0.852 - 0.913)	0.012(-0.0197 - 0.0434)	1.38% (-2.22% - 5.09%)
Finland	0.92(0.887 - 0.952)	0.0238(-0.00869 - 0.0588)	2.65% (-0.961% - 6.58%)	0.83(0.801 - 0.859)	0.0186(-0.00994 - 0.0489)	2.29% (-1.21% - 6.08%)
France	0.983(0.948 - 1.02)	0.0178(-0.0148 - 0.0508)	1.84% (-1.52% - 5.4%)	0.87(0.839 - 0.901)	0.0114(-0.0212 - 0.0425)	1.33% (-2.38% - 5.12%)
Germany	0.953(0.918 - 0.989)	0.00939(-0.0275 - 0.0486)	0.995% (-2.86% - 5.31%)	0.861(0.831 - 0.894)	0.00607(-0.0238 - 0.0369)	0.709% (-2.75% - 4.36%)

Greece	0.856(0.824 - 0.888)	0.0121(-0.0182 - 0.0448)	1.44% (-2.14% - 5.33%)	0.787(0.759 - 0.818)	-0.000426(-0.0291 - 0.0271)	-0.0542% (-3.69% - 3.5%)
Iceland	0.94(0.905 - 0.973)	0.0299(-0.00411 - 0.063)	3.29% (-0.45% - 7.08%)	0.856(0.825 - 0.888)	0.0185(-0.0101 - 0.049)	2.21% (-1.18% - 5.94%)
Ireland	0.935(0.9 - 0.97)	0.0295(-0.00357 - 0.0617)	3.26% (-0.374% - 6.97%)	0.868(0.836 - 0.899)	0.0236(-0.0104 - 0.0553)	2.8% (-1.23% - 6.64%)
Israel	0.915(0.881 - 0.954)	0.0242(-0.00665 - 0.0577)	2.71% (-0.748% - 6.63%)	0.84(0.809 - 0.872)	0.0138(-0.0181 - 0.0442)	1.67% (-2.14% - 5.48%)
Italy	0.929(0.902 - 0.957)	0.00303(-0.00921 - 0.0157)	0.327% (-0.966% - 1.71%)	0.824(0.8 - 0.848)	-0.0135(-0.0242 - -0.00217)	-1.62% (-2.85% - -0.263%)
Luxembourg	0.927(0.892 - 0.964)	0.0118(-0.0196 - 0.0456)	1.29% (-2.15% - 5.03%)	0.845(0.814 - 0.878)	0.0118(-0.0178 - 0.0457)	1.42% (-2.09% - 5.68%)
Malta	0.925(0.889 - 0.96)	0.0276(-0.00518 - 0.0634)	3.08% (-0.567% - 7.11%)	0.836(0.807 - 0.866)	0.00976(-0.0185 - 0.0396)	1.18% (-2.15% - 4.83%)
Monaco	0.94(0.905 - 0.976)	0.00514(-0.0285 - 0.0383)	0.549% (-2.98% - 4.15%)	0.85(0.82 - 0.88)	0.00182(-0.0271 - 0.0319)	0.215% (-3.16% - 3.83%)
Netherlands	0.878(0.844 - 0.91)	0.0101(-0.0236 - 0.0432)	1.17% (-2.68% - 5%)	0.849(0.819 - 0.88)	0.00814(-0.0242 - 0.0378)	0.968% (-2.83% - 4.59%)
Norway	0.932(0.906 - 0.961)	0.00319(-0.00997 - 0.0153)	0.343% (-1.06% - 1.69%)	0.854(0.829 - 0.879)	0.00988(5.37e-05 - 0.0195)	1.17% (0.00635% - 2.34%)
Portugal	0.928(0.897 - 0.964)	0.0171(-0.0173 - 0.0499)	1.87% (-1.86% - 5.58%)	0.821(0.793 - 0.851)	0.00742(-0.0215 - 0.0356)	0.912% (-2.55% - 4.46%)
San Marino	0.935(0.901 - 0.971)	0.00249(-0.0328 - 0.0355)	0.268% (-3.4% - 3.98%)	0.851(0.821 - 0.883)	-0.000379(-0.0284 - 0.0301)	-0.0444% (-3.3% - 3.6%)
Spain	0.921(0.887 - 0.959)	0.0218(-0.0153 - 0.0576)	2.42% (-1.69% - 6.46%)	0.822(0.793 - 0.853)	0.00846(-0.0224 - 0.0393)	1.04% (-2.71% - 4.9%)
Sweden	0.898(0.867 - 0.93)	0.000348(-0.0252 - 0.0259)	0.0388% (-2.75% - 2.91%)	0.946(0.916 - 0.979)	-0.0254(-0.0529 - 0.000771)	-2.61% (-5.34% - 0.082%)
Switzerland	0.924(0.89 - 0.959)	0.00754(-0.0242 - 0.0419)	0.823% (-2.56% - 4.66%)	0.838(0.808 - 0.869)	0.00833(-0.0222 - 0.0366)	1% (-2.65% - 4.49%)
UK	0.955(0.928 - 0.984)	0.0262(0.0193 - 0.0332)	2.83% (2.08% - 3.6%)	0.848(0.824 - 0.872)	0.0175(0.0122 - 0.023)	2.1% (1.45% - 2.75%)
Latin America and Caribbean	0.935(0.906 - 0.964)	0.0599(0.0498 - 0.0704)	6.84% (5.65% - 8.11%)	0.794(0.771 - 0.819)	0.0457(0.0357 - 0.0552)	6.11% (4.73% - 7.4%)
Andean Latin America	0.944(0.914 - 0.978)	0.0634(0.0403 - 0.0869)	7.19% (4.52% - 10.1%)	0.818(0.789 - 0.845)	0.048(0.0291 - 0.0674)	6.23% (3.75% - 8.98%)
Bolivia	0.921(0.886 - 0.961)	0.0696(0.035 - 0.103)	8.17% (4.03% - 12.3%)	0.795(0.761 - 0.827)	0.0497(0.0187 - 0.0798)	6.66% (2.4% - 10.8%)
Ecuador	0.944(0.908 - 0.981)	0.0576(0.0222 - 0.0934)	6.5% (2.42% - 10.8%)	0.815(0.782 - 0.849)	0.0407(0.00892 - 0.0682)	5.26% (1.07% - 8.97%)
Peru	0.952(0.916 - 0.991)	0.0654(0.0293 - 0.101)	7.35% (3.26% - 11.8%)	0.827(0.795 - 0.858)	0.0519(0.0225 - 0.0837)	6.69% (2.89% - 11.2%)
Caribbean	0.924(0.895 - 0.956)	0.0384(0.0203 - 0.0564)	4.33% (2.31% - 6.36%)	0.83(0.803 - 0.857)	0.0271(0.0126 - 0.0426)	3.38% (1.58% - 5.36%)
Antigua and Barbuda	0.947(0.91 - 0.984)	0.0355(-0.0011 - 0.0716)	3.89% (-0.117% - 7.96%)	0.851(0.818 - 0.884)	0.0318(-2.74e-05 - 0.0622)	3.88% (-0.0033% - 7.73%)
The Bahamas	0.96(0.922 - 0.998)	0.0285(-0.00866 - 0.0665)	3.06% (-0.9% - 7.34%)	0.864(0.831 - 0.899)	0.0257(-0.00807 - 0.0588)	3.06% (-0.912% - 7.18%)
Barbados	0.967(0.932 - 1)	0.0205(-0.0149 - 0.0531)	2.16% (-1.56% - 5.69%)	0.866(0.832 - 0.9)	0.0207(-0.00938 - 0.0521)	2.45% (-1.07% - 6.27%)
Belize	0.946(0.907 - 0.988)	0.0726(0.0333 - 0.113)	8.31% (3.71% - 13.2%)	0.85(0.816 - 0.886)	0.065(0.032 - 0.0993)	8.27% (4.05% - 12.8%)
Bermuda	0.948(0.914 - 0.985)	-0.00342(-0.0388 - 0.0339)	-0.359% (-3.98% - 3.66%)	0.847(0.817 - 0.879)	-0.0068(-0.041 - 0.0281)	-0.797% (-4.71% - 3.34%)
Cuba	0.924(0.889 - 0.959)	0.0287(-0.00768 - 0.065)	3.21% (-0.848% - 7.39%)	0.828(0.795 - 0.859)	0.0132(-0.0185 - 0.0451)	1.62% (-2.26% - 5.54%)
Dominica	0.964(0.925 - 1)	0.061(0.0277 - 0.0954)	6.75% (3.01% - 10.8%)	0.86(0.828 - 0.892)	0.0546(0.0232 - 0.0845)	6.76% (2.78% - 10.8%)
Dominican Republic	0.932(0.895 - 0.971)	0.048(0.0135 - 0.0819)	5.43% (1.5% - 9.56%)	0.836(0.803 - 0.871)	0.0356(0.00414 - 0.0701)	4.45% (0.501% - 9.02%)
Grenada	0.956(0.918 - 0.992)	0.0762(0.038 - 0.115)	8.65% (4.23% - 13.2%)	0.852(0.819 - 0.885)	0.0638(0.0324 - 0.0945)	8.1% (4.1% - 12.2%)
Guyana	0.927(0.889 - 0.967)	0.0491(0.0114 - 0.0856)	5.59% (1.25% - 9.82%)	0.834(0.801 - 0.869)	0.0411(0.00932 - 0.0731)	5.19% (1.15% - 9.38%)
Haiti	0.892(0.853 - 0.929)	0.0519(0.016 - 0.0827)	6.17% (1.86% - 9.95%)	0.806(0.775 - 0.841)	0.0417(0.0113 - 0.0745)	5.45% (1.44% - 9.88%)
Jamaica	0.953(0.914 - 0.993)	0.064(0.0268 - 0.103)	7.2% (3.01% - 11.8%)	0.853(0.821 - 0.886)	0.0522(0.0186 - 0.0835)	6.52% (2.31% - 10.6%)
Puerto Rico	0.966(0.928 - 1.01)	0.0425(0.00824 - 0.0773)	4.61% (0.862% - 8.43%)	0.86(0.83 - 0.893)	0.0259(-0.00506 - 0.0567)	3.11% (-0.589% - 6.87%)
Saint Kitts and Nevis	0.97(0.929 - 1.01)	0.0609(0.0241 - 0.101)	6.7% (2.62% - 11.2%)	0.871(0.838 - 0.906)	0.0584(0.0258 - 0.0909)	7.19% (3.15% - 11.4%)
Saint Lucia	0.956(0.921 - 0.992)	0.0714(0.0364 - 0.105)	8.08% (3.95% - 12.1%)	0.856(0.823 - 0.89)	0.0606(0.0261 - 0.0917)	7.61% (3.19% - 11.8%)
Saint Vincent and the Grenadines	0.938(0.903 - 0.977)	0.0516(0.016 - 0.0866)	5.82% (1.78% - 9.92%)	0.84(0.811 - 0.873)	0.0475(0.0195 - 0.0772)	5.99% (2.45% - 9.82%)
Suriname	0.922(0.886 - 0.959)	0.0303(-0.00519 - 0.0643)	3.4% (-0.576% - 7.32%)	0.828(0.797 - 0.86)	0.0331(0.00308 - 0.0634)	4.16% (0.393% - 8.16%)
Trinidad and Tobago	0.949(0.914 - 0.985)	0.0453(0.00793 - 0.0814)	5.02% (0.87% - 9.14%)	0.85(0.818 - 0.884)	0.0388(0.00523 - 0.0691)	4.78% (0.606% - 8.62%)
Virgin Islands	0.956(0.92 - 0.99)	0.0408(0.0043 - 0.0779)	4.48% (0.456% - 8.53%)	0.854(0.823 - 0.886)	0.0226(-0.00948 - 0.0538)	2.73% (-1.12% - 6.63%)
Central Latin America	0.943(0.913 - 0.974)	0.0633(0.0502 - 0.0758)	7.19% (5.61% - 8.73%)	0.785(0.761 - 0.812)	0.0424(0.0312 - 0.053)	5.7% (4.16% - 7.18%)
Colombia	0.955(0.917 - 0.995)	0.0645(0.0294 - 0.0995)	7.23% (3.3% - 11.3%)	0.804(0.772 - 0.835)	0.0457(0.0151 - 0.0778)	6.02% (1.98% - 10.4%)
Costa Rica	0.944(0.908 - 0.984)	0.0662(0.0322 - 0.102)	7.54% (3.53% - 11.9%)	0.797(0.767 - 0.828)	0.052(0.0225 - 0.0819)	6.98% (2.95% - 11.1%)
El Salvador	0.92(0.885 - 0.957)	0.0465(0.0132 - 0.0804)	5.32% (1.45% - 9.33%)	0.784(0.754 - 0.815)	0.0382(0.00809 - 0.0675)	5.11% (1.05% - 9.28%)
Guatemala	0.903(0.865 - 0.941)	0.0818(0.0472 - 0.115)	9.93% (5.58% - 14.3%)	0.764(0.734 - 0.796)	0.0604(0.0327 - 0.0906)	8.57% (4.53% - 13.2%)
Honduras	0.897(0.858 - 0.936)	0.07(0.0347 - 0.106)	8.47% (4.07% - 13%)	0.76(0.729 - 0.792)	0.0539(0.0264 - 0.0822)	7.64% (3.69% - 11.9%)
Mexico	0.957(0.928 - 0.987)	0.0698(0.0576 - 0.0813)	7.85% (6.43% - 9.34%)	0.786(0.763 - 0.812)	0.0414(0.0293 - 0.0528)	5.55% (3.89% - 7.07%)
Nicaragua	0.912(0.874 - 0.952)	0.0764(0.0419 - 0.112)	9.15% (4.88% - 13.8%)	0.769(0.739 - 0.802)	0.0547(0.0197 - 0.0833)	7.65% (2.74% - 12%)
Panama	0.919(0.884 - 0.956)	0.0461(0.0129 - 0.0801)	5.27% (1.45% - 9.3%)	0.775(0.746 - 0.805)	0.0363(0.00731 - 0.0674)	4.91% (0.971% - 9.3%)
Venezuela	0.92(0.883 - 0.957)	0.0374(-0.000258 - 0.0773)	4.28% (-0.028% - 8.98%)	0.776(0.746 - 0.81)	0.0352(0.00807 - 0.0654)	4.75% (1.08% - 8.89%)
Tropical Latin America	0.925(0.897 - 0.954)	0.0592(0.0459 - 0.073)	6.83% (5.26% - 8.59%)	0.789(0.767 - 0.814)	0.0537(0.0417 - 0.0654)	7.3% (5.6% - 8.96%)
Brazil	0.926(0.899 - 0.955)	0.059(0.0459 - 0.0735)	6.81% (5.2% - 8.6%)	0.79(0.767 - 0.815)	0.0538(0.0416 - 0.0657)	7.31% (5.57% - 9.04%)
Paraguay	0.895(0.858 - 0.934)	0.0692(0.0361 - 0.103)	8.26% (4.16% - 12.4%)	0.761(0.732 - 0.792)	0.0549(0.0267 - 0.0832)	7.68% (3.63% - 11.9%)
North Africa and Middle East	0.859(0.834 - 0.886)	0.0464(0.0351 - 0.0585)	5.72% (4.29% - 7.27%)	0.772(0.749 - 0.798)	0.0477(0.0371 - 0.0589)	6.58% (5.11% - 8.18%)
North Africa and Middle East	0.859(0.834 - 0.886)	0.0464(0.0351 - 0.0585)	5.72% (4.29% - 7.27%)	0.772(0.749 - 0.798)	0.0477(0.0371 - 0.0589)	6.58% (5.11% - 8.18%)
Afghanistan	0.798(0.764 - 0.836)	-0.00298(-0.0349 - 0.032)	-0.372% (-4.32% - 4.09%)	0.716(0.685 - 0.749)	-0.0131(-0.0436 - 0.0174)	-1.79% (-5.79% - 2.44%)
Algeria	0.848(0.815 - 0.881)	0.0425(0.00895 - 0.0751)	5.27% (1.09% - 9.55%)	0.769(0.741 - 0.8)	0.0501(0.0199 - 0.0807)	6.97% (2.76% - 11.3%)
Bahrain	0.916(0.876 - 0.958)	0.0493(0.0139 - 0.0875)	5.69% (1.59% - 10.3%)	0.804(0.772 - 0.838)	0.0526(0.0227 - 0.0812)	7% (2.96% - 11%)
Egypt	0.853(0.82 - 0.887)	0.033(-0.000759 - 0.067)	4.05% (-0.0913% - 8.3%)	0.77(0.739 - 0.803)	0.0353(0.00525 - 0.0621)	4.84% (0.694% - 8.79%)
Iran	0.85(0.824 - 0.879)	0.0408(0.0246 - 0.0579)	5.04% (2.92% - 7.24%)	0.764(0.741 - 0.791)	0.059(0.0448 - 0.0731)	8.36% (6.23% - 10.5%)
Iraq	0.848(0.813 - 0.883)	0.0451(0.00976 - 0.0778)	5.68% (1.16% - 9.95%)	0.767(0.737 - 0.798)	0.0497(0.0216 - 0.078)	7.04% (3.01% - 11.2%)
Jordan	0.865(0.83 - 0.904)	0.0623(0.0266 - 0.0965)	7.77% (3.31% - 12.5%)	0.759(0.726 - 0.79)	0.0647(0.0318 - 0.095)	9.32% (4.52% - 13.9%)
Kuwait	0.907(0.87 - 0.947)	0.0413(0.00334 - 0.0797)	4.76% (0.383% - 9.37%)	0.811(0.779 - 0.845)	0.0705(0.0386 - 0.104)	9.52% (5.12% - 14.4%)

Lebanon	0.825(0.793 - 0.856)	0.0487(0.0117 - 0.0833)	6.25% (1.5% - 11%)	0.75(0.723 - 0.779)	0.0373(0.0107 - 0.0696)	5.24% (1.5% - 9.89%)
Libya	0.88(0.847 - 0.915)	0.0583(0.0239 - 0.0915)	7.09% (2.81% - 11.5%)	0.797(0.766 - 0.83)	0.0772(0.042 - 0.11)	10.7% (5.7% - 15.4%)
Morocco	0.882(0.849 - 0.919)	0.0598(0.03 - 0.0934)	7.32% (3.53% - 11.8%)	0.91(0.877 - 0.945)	0.0697(0.0374 - 0.102)	8.34% (4.35% - 12.4%)
Oman	0.911(0.874 - 0.952)	0.0836(0.0451 - 0.12)	10.1% (5.3% - 14.8%)	0.792(0.757 - 0.826)	0.0941(0.0637 - 0.125)	13.5% (8.89% - 18.4%)
Palestine	0.825(0.791 - 0.863)	0.0683(0.036 - 0.1)	9.03% (4.67% - 13.6%)	0.74(0.708 - 0.772)	0.0651(0.0373 - 0.0933)	9.64% (5.38% - 14%)
Qatar	0.956(0.907 - 1.01)	0.056(0.014 - 0.101)	6.28% (1.51% - 11.5%)	0.785(0.752 - 0.821)	0.0688(0.0361 - 0.102)	9.57% (5.04% - 14.5%)
Saudi Arabia	0.912(0.871 - 0.953)	0.0875(0.0481 - 0.124)	10.6% (5.76% - 15.3%)	0.817(0.782 - 0.852)	0.103(0.0703 - 0.134)	14.4% (9.68% - 19.2%)
Sudan	0.832(0.797 - 0.868)	0.0473(0.0148 - 0.0776)	6.03% (1.86% - 10.1%)	0.754(0.723 - 0.789)	0.0434(0.0162 - 0.0733)	6.12% (2.22% - 10.4%)
Syria	0.86(0.825 - 0.895)	0.0584(0.0238 - 0.0961)	7.38% (2.93% - 12.4%)	0.775(0.742 - 0.808)	0.0765(0.0449 - 0.107)	10.8% (6.21% - 15.4%)
Tunisia	0.852(0.821 - 0.886)	0.0458(0.0126 - 0.079)	5.69% (1.51% - 10.2%)	0.778(0.747 - 0.808)	0.0548(0.0254 - 0.0829)	7.58% (3.44% - 11.7%)
Türkiye	0.909(0.875 - 0.945)	0.0668(0.0307 - 0.102)	7.93% (3.61% - 12.2%)	0.771(0.742 - 0.8)	0.049(0.0188 - 0.0819)	6.8% (2.55% - 11.4%)
United Arab Emirates	0.923(0.881 - 0.972)	0.0525(0.00602 - 0.103)	5.93% (0.66% - 11.9%)	0.832(0.796 - 0.871)	0.0864(0.0465 - 0.126)	11.6% (6.22% - 17.4%)
Yemen	0.784(0.748 - 0.819)	0.0404(0.00521 - 0.0729)	5.43% (0.686% - 10.1%)	0.707(0.679 - 0.74)	0.0404(0.0102 - 0.0694)	6.05% (1.5% - 10.8%)
South Asia	0.858(0.831 - 0.887)	0.0538(0.0451 - 0.0618)	6.69% (5.59% - 7.72%)	0.765(0.741 - 0.79)	0.0523(0.0446 - 0.0598)	7.33% (6.17% - 8.48%)
South Asia	0.858(0.831 - 0.887)	0.0538(0.0451 - 0.0618)	6.69% (5.59% - 7.72%)	0.765(0.741 - 0.79)	0.0523(0.0446 - 0.0598)	7.33% (6.17% - 8.48%)
Bangladesh	0.838(0.804 - 0.875)	0.0638(0.031 - 0.0939)	8.24% (3.9% - 12.4%)	0.761(0.73 - 0.792)	0.064(0.0363 - 0.0922)	9.19% (5.03% - 13.5%)
Bhutan	0.927(0.885 - 0.969)	0.0763(0.0393 - 0.115)	8.98% (4.52% - 14%)	0.819(0.786 - 0.853)	0.072(0.0387 - 0.104)	9.63% (5.06% - 14.3%)
India	0.86(0.833 - 0.889)	0.0509(0.0417 - 0.0598)	6.28% (5.1% - 7.38%)	0.765(0.742 - 0.791)	0.0498(0.0414 - 0.0576)	6.95% (5.76% - 8.12%)
Nepal	0.844(0.811 - 0.879)	0.0777(0.048 - 0.106)	10.1% (6.1% - 14.1%)	0.768(0.736 - 0.802)	0.0725(0.0438 - 0.0992)	10.4% (6.27% - 14.5%)
Pakistan	0.858(0.829 - 0.889)	0.0606(0.0414 - 0.0795)	7.6% (5.1% - 10.1%)	0.766(0.739 - 0.795)	0.056(0.0391 - 0.0739)	7.9% (5.32% - 10.5%)
Southeast Asia, east Asia, and Oceania	0.882(0.857 - 0.908)	0.0409(0.0293 - 0.0525)	4.9% (3.47% - 6.43%)	0.758(0.736 - 0.782)	0.0147(0.00418 - 0.0262)	1.99% (0.557% - 3.6%)
East Asia	0.88(0.855 - 0.908)	0.0366(0.0223 - 0.0517)	4.37% (2.62% - 6.26%)	0.776(0.753 - 0.802)	0.00994(-0.00276 - 0.0247)	1.31% (-0.364% - 3.27%)
China	0.881(0.855 - 0.908)	0.0363(0.0218 - 0.0514)	4.34% (2.57% - 6.28%)	0.775(0.752 - 0.801)	0.00958(-0.00342 - 0.0245)	1.26% (-0.447% - 3.25%)
North Korea	0.858(0.822 - 0.895)	0.0242(-0.00651 - 0.0575)	2.9% (-0.779% - 6.9%)	0.776(0.747 - 0.807)	0.00187(-0.0268 - 0.028)	0.241% (-3.45% - 3.66%)
Taiwan (province of China)	0.891(0.864 - 0.92)	0.0626(0.0275 - 0.1)	7.55% (3.22% - 12.5%)	0.828(0.802 - 0.855)	0.0417(0.00815 - 0.0731)	5.31% (0.993% - 9.47%)
Oceania	0.848(0.815 - 0.882)	0.00861(-0.0162 - 0.0325)	1.03% (-1.88% - 3.94%)	0.74(0.712 - 0.771)	0.0128(-0.00704 - 0.0333)	1.75% (-0.942% - 4.56%)
American Samoa	0.93(0.893 - 0.969)	0.0341(0.00124 - 0.0723)	3.81% (0.139% - 8.22%)	0.805(0.773 - 0.838)	0.027(-0.00347 - 0.0567)	3.47% (-0.453% - 7.54%)
Cook Islands	0.934(0.9 - 0.97)	0.0426(0.00882 - 0.0758)	4.78% (0.948% - 8.71%)	0.805(0.773 - 0.838)	0.0359(0.00695 - 0.065)	4.68% (0.885% - 8.68%)
Fiji	0.901(0.866 - 0.936)	0.0298(-0.00924 - 0.0667)	3.42% (-1.02% - 7.87%)	0.78(0.75 - 0.81)	0.0246(-0.00617 - 0.0534)	3.26% (-0.808% - 7.26%)
Guam	0.919(0.885 - 0.954)	0.0208(-0.015 - 0.0536)	2.3% (-1.6% - 6.05%)	0.786(0.757 - 0.817)	0.0209(-0.00953 - 0.0514)	2.73% (-1.21% - 6.88%)
Kiribati	0.858(0.823 - 0.894)	0.018(-0.0164 - 0.0539)	2.14% (-1.91% - 6.7%)	0.751(0.723 - 0.782)	0.0199(-0.00687 - 0.0461)	2.72% (-0.92% - 6.43%)
Marshall Islands	0.898(0.862 - 0.935)	0.0571(0.0209 - 0.0938)	6.78% (2.34% - 11.3%)	0.78(0.749 - 0.814)	0.0488(0.0174 - 0.0791)	6.66% (2.32% - 10.9%)
Federated States of Micronesia	0.907(0.869 - 0.945)	0.0533(0.0192 - 0.0866)	6.24% (2.22% - 10.3%)	0.782(0.751 - 0.814)	0.0412(0.012 - 0.0732)	5.55% (1.56% - 10.1%)
Nauru	0.875(0.839 - 0.914)	0.0188(-0.0137 - 0.0509)	2.19% (-1.61% - 6.11%)	0.764(0.733 - 0.795)	0.0193(-0.0106 - 0.0479)	2.6% (-1.39% - 6.53%)
Niue	0.922(0.886 - 0.957)	0.0462(0.0134 - 0.0768)	5.27% (1.52% - 9.02%)	0.787(0.756 - 0.818)	0.0395(0.011 - 0.0684)	5.28% (1.44% - 9.37%)
Northern Mariana Islands	0.949(0.912 - 0.986)	-0.00823(-0.0449 - 0.0304)	-0.872% (-4.62% - 3.27%)	0.809(0.779 - 0.842)	-0.0058(-0.038 - 0.0276)	-0.714% (-4.57% - 3.51%)
Palau	0.943(0.905 - 0.981)	0.0319(-0.00524 - 0.0664)	3.5% (-0.554% - 7.44%)	0.799(0.768 - 0.828)	0.0204(-0.0115 - 0.0554)	2.62% (-1.44% - 7.22%)
Papua New Guinea	0.839(0.802 - 0.876)	0.00919(-0.024 - 0.0418)	1.11% (-2.8% - 5.1%)	0.733(0.702 - 0.766)	0.0138(-0.0139 - 0.0427)	1.92% (-1.88% - 6.07%)
Samoa	0.869(0.835 - 0.905)	0.00504(-0.0295 - 0.0351)	0.583% (-3.37% - 4.19%)	0.753(0.721 - 0.785)	0.0112(-0.0211 - 0.0408)	1.51% (-2.78% - 5.53%)
Solomon Islands	0.853(0.817 - 0.89)	0.028(-0.00398 - 0.0606)	3.39% (-0.474% - 7.42%)	0.747(0.716 - 0.778)	0.0289(0.00061 - 0.0565)	4.03% (0.0842% - 8.03%)
Tokelau	0.895(0.86 - 0.93)	0.0488(0.0161 - 0.0814)	5.76% (1.87% - 9.84%)	0.772(0.742 - 0.801)	0.0403(0.0137 - 0.0692)	5.52% (1.85% - 9.61%)
Tonga	0.87(0.837 - 0.906)	0.024(-0.00725 - 0.0587)	2.84% (-0.842% - 7.07%)	0.762(0.733 - 0.792)	0.0267(-0.00294 - 0.0557)	3.63% (-0.383% - 7.84%)
Tuvalu	0.885(0.852 - 0.922)	0.0546(0.0221 - 0.0866)	6.6% (2.63% - 10.7%)	0.759(0.73 - 0.788)	0.0277(0.00129 - 0.054)	3.79% (0.173% - 7.64%)
Vanuatu	0.837(0.801 - 0.876)	0.0361(0.00435 - 0.068)	4.5% (0.521% - 8.63%)	0.732(0.701 - 0.764)	0.0325(0.00391 - 0.063)	4.65% (0.549% - 9.16%)
Southeast Asia	0.886(0.858 - 0.914)	0.0522(0.0412 - 0.0637)	6.27% (4.89% - 7.78%)	0.721(0.7 - 0.744)	0.0334(0.0243 - 0.0429)	4.86% (3.53% - 6.29%)
Cambodia	0.836(0.801 - 0.871)	0.0682(0.0378 - 0.0997)	8.88% (4.85% - 13.3%)	0.681(0.654 - 0.708)	0.0381(0.0132 - 0.0654)	5.92% (2.03% - 10.5%)
Indonesia	0.886(0.859 - 0.914)	0.0514(0.0388 - 0.0642)	6.15% (4.6% - 7.76%)	0.72(0.699 - 0.743)	0.0309(0.0198 - 0.0421)	4.47% (2.83% - 6.22%)
Laos	0.853(0.82 - 0.889)	0.0621(0.031 - 0.0953)	7.85% (3.84% - 12.4%)	0.695(0.667 - 0.725)	0.0377(0.0115 - 0.0638)	5.74% (1.73% - 9.84%)
Malaysia	0.928(0.89 - 0.967)	0.0731(0.0422 - 0.106)	8.54% (4.78% - 12.7%)	0.75(0.721 - 0.779)	0.0496(0.0225 - 0.0778)	7.08% (3.13% - 11.4%)
Maldives	0.962(0.918 - 1.01)	0.133(0.0937 - 0.176)	16% (11.1% - 21.5%)	0.757(0.725 - 0.789)	0.0755(0.0449 - 0.107)	11% (6.45% - 15.9%)
Mauritius	0.935(0.9 - 0.971)	0.0477(0.0111 - 0.0816)	5.37% (1.21% - 9.53%)	0.752(0.724 - 0.78)	0.0338(0.00515 - 0.0604)	4.71% (0.683% - 8.57%)
Myanmar	0.873(0.836 - 0.908)	0.0575(0.0237 - 0.0907)	7.1% (2.87% - 11.7%)	0.713(0.684 - 0.741)	0.0456(0.0199 - 0.0716)	6.87% (2.91% - 11.1%)
Philippines	0.873(0.845 - 0.903)	0.0528(0.045 - 0.0615)	6.43% (5.43% - 7.51%)	0.701(0.68 - 0.724)	0.036(0.0296 - 0.0427)	5.4% (4.42% - 6.45%)
Seychelles	0.92(0.882 - 0.958)	0.0418(0.00768 - 0.0765)	4.76% (0.856% - 8.85%)	0.74(0.712 - 0.772)	0.0295(0.00092 - 0.0568)	4.16% (0.128% - 8.29%)
Sri Lanka	0.898(0.862 - 0.934)	0.0398(0.00705 - 0.0741)	4.64% (0.814% - 8.88%)	0.694(0.669 - 0.721)	0.0288(0.00364 - 0.0554)	4.33% (0.543% - 8.51%)
Thailand	0.918(0.881 - 0.955)	0.0454(0.00846 - 0.0803)	5.22% (0.953% - 9.39%)	0.781(0.752 - 0.81)	0.0279(-0.00242 - 0.0587)	3.7% (-0.322% - 7.85%)
Timor-Leste	0.826(0.792 - 0.864)	0.027(-0.00561 - 0.0596)	3.38% (-0.689% - 7.63%)	0.675(0.647 - 0.704)	0.0216(-0.00394 - 0.0496)	3.29% (-0.595% - 7.67%)
Viet Nam	0.874(0.839 - 0.909)	0.0563(0.0221 - 0.0885)	6.9% (2.67% - 11.1%)	0.712(0.684 - 0.74)	0.0397(0.015 - 0.0643)	5.92% (2.21% - 9.78%)
Sub-Saharan Africa	0.809(0.782 - 0.838)	0.0358(0.0307 - 0.0418)	4.64% (3.97% - 5.38%)	0.708(0.683 - 0.733)	0.0285(0.024 - 0.0331)	4.2% (3.53% - 4.87%)
Central sub-Saharan Africa	0.793(0.762 - 0.826)	0.0233(2.41e-05 - 0.0469)	3.02% (0.00306% - 6.14%)	0.699(0.673 - 0.727)	0.0158(-0.00359 - 0.0358)	2.3% (-0.519% - 5.3%)
Angola	0.77(0.736 - 0.803)	0.0165(-0.0144 - 0.0492)	2.18% (-1.87% - 6.64%)	0.683(0.655 - 0.713)	0.0185(-0.00954 - 0.0464)	2.78% (-1.4% - 7.09%)
Central African Republic	0.793(0.76 - 0.827)	0.0247(-0.00506 - 0.0554)	3.22% (-0.642% - 7.43%)	0.701(0.672 - 0.73)	0.0202(-0.00571 - 0.046)	2.96% (-0.839% - 6.95%)
Congo (Brazzaville)	0.816(0.782 - 0.853)	0.0331(0.000597 - 0.0629)	4.22% (0.0749% - 8.31%)	0.72(0.687 - 0.751)	0.0267(-0.00302 - 0.0557)	3.83% (-0.426% - 8.19%)
DR Congo	0.798(0.763 - 0.835)	0.0243(-0.00826 - 0.0576)	3.13% (-1.05% - 7.61%)	0.701(0.672 - 0.734)	0.0142(-0.0138 - 0.0446)	2.07% (-1.94% - 6.6%)

Equatorial Guinea	0.855(0.815 - 0.899)	0.111(0.0753 - 0.146)	14.9% (10% - 20%)	0.746(0.715 - 0.779)	0.0726(0.0437 - 0.0999)	10.8% (6.41% - 15.1%)
Gabon	0.824(0.79 - 0.859)	0.0497(0.0167 - 0.0801)	6.42% (2.14% - 10.5%)	0.73(0.698 - 0.761)	0.0469(0.0164 - 0.0759)	6.86% (2.37% - 11.2%)
Eastern sub-Saharan Africa	0.806(0.779 - 0.836)	0.0466(0.0383 - 0.0548)	6.13% (5.05% - 7.25%)	0.705(0.681 - 0.731)	0.0355(0.028 - 0.0426)	5.3% (4.19% - 6.38%)
Burundi	0.779(0.745 - 0.814)	0.0324(-0.000597 - 0.0649)	4.32% (-0.077% - 8.91%)	0.687(0.658 - 0.719)	0.0235(-0.00326 - 0.0544)	3.54% (-0.481% - 8.37%)
Comoros	0.815(0.782 - 0.85)	0.0614(0.0271 - 0.093)	8.15% (3.53% - 12.7%)	0.715(0.687 - 0.745)	0.0477(0.0201 - 0.075)	7.13% (2.92% - 11.4%)
Djibouti	0.797(0.765 - 0.832)	0.0417(0.00749 - 0.0748)	5.43% (0.958% - 9.9%)	0.7(0.673 - 0.73)	0.0336(0.00604 - 0.062)	4.98% (0.884% - 9.39%)
Eritrea	0.787(0.754 - 0.822)	0.0479(0.0142 - 0.0831)	6.45% (1.91% - 11.7%)	0.692(0.663 - 0.721)	0.0318(0.00172 - 0.0603)	4.82% (0.258% - 9.34%)
Ethiopia	0.838(0.807 - 0.871)	0.0634(0.0469 - 0.0802)	8.17% (5.95% - 10.4%)	0.72(0.692 - 0.747)	0.0439(0.0293 - 0.0586)	6.49% (4.32% - 8.72%)
Kenya	0.833(0.805 - 0.863)	0.0696(0.0618 - 0.0778)	9.04% (8.02% - 10.1%)	0.72(0.696 - 0.745)	0.0541(0.0475 - 0.0612)	8.05% (7% - 9.15%)
Madagascar	0.787(0.754 - 0.821)	0.0385(0.00627 - 0.0703)	5.14% (0.799% - 9.54%)	0.694(0.666 - 0.726)	0.0318(0.00511 - 0.0582)	4.81% (0.744% - 9.03%)
Malawi	0.788(0.752 - 0.824)	0.0338(0.00308 - 0.0649)	4.48% (0.409% - 8.66%)	0.696(0.665 - 0.726)	0.0275(0.000247 - 0.0558)	4.13% (0.037% - 8.6%)
Mozambique	0.776(0.741 - 0.814)	0.031(0.00156 - 0.0612)	4.17% (0.206% - 8.34%)	0.69(0.66 - 0.721)	0.023(-0.00533 - 0.05)	3.45% (-0.773% - 7.55%)
Rwanda	0.803(0.77 - 0.84)	0.047(0.0134 - 0.0793)	6.23% (1.69% - 10.5%)	0.707(0.677 - 0.738)	0.0368(0.0104 - 0.0639)	5.48% (1.55% - 9.75%)
Somalia	0.777(0.742 - 0.814)	0.00895(-0.021 - 0.0409)	1.17% (-2.65% - 5.38%)	0.682(0.649 - 0.714)	0.0043(-0.0229 - 0.0321)	0.635% (-3.36% - 4.79%)
South Sudan	0.802(0.767 - 0.84)	0.016(-0.0172 - 0.0498)	2.04% (-2.15% - 6.52%)	0.708(0.676 - 0.738)	0.0231(-0.0064 - 0.0528)	3.35% (-0.885% - 7.85%)
Uganda	0.792(0.755 - 0.829)	0.0365(0.00367 - 0.0708)	4.83% (0.486% - 9.56%)	0.702(0.673 - 0.734)	0.0305(0.00165 - 0.0582)	4.54% (0.243% - 8.9%)
Tanzania	0.791(0.757 - 0.826)	0.0477(0.0182 - 0.0801)	6.42% (2.34% - 10.9%)	0.702(0.672 - 0.732)	0.0383(0.0126 - 0.0648)	5.78% (1.87% - 10.1%)
Zambia	0.8(0.766 - 0.836)	0.0408(0.00906 - 0.0715)	5.37% (1.14% - 9.54%)	0.704(0.674 - 0.736)	0.0299(0.00199 - 0.0587)	4.42% (0.288% - 8.93%)
Southern sub-Saharan Africa	0.847(0.819 - 0.879)	0.0456(0.0333 - 0.059)	5.69% (4.11% - 7.44%)	0.754(0.731 - 0.78)	0.0289(0.0178 - 0.0402)	3.98% (2.46% - 5.6%)
Botswana	0.858(0.822 - 0.896)	0.0811(0.0505 - 0.114)	10.4% (6.36% - 15%)	0.769(0.736 - 0.803)	0.0553(0.026 - 0.0862)	7.76% (3.65% - 12.4%)
Eswatini	0.866(0.83 - 0.905)	0.0721(0.0359 - 0.106)	9.07% (4.37% - 13.7%)	0.781(0.747 - 0.818)	0.0488(0.0159 - 0.0807)	6.66% (2.13% - 11.2%)
Lesotho	0.803(0.769 - 0.841)	0.0677(0.0327 - 0.101)	9.21% (4.37% - 13.9%)	0.719(0.686 - 0.753)	0.0352(0.00704 - 0.0637)	5.13% (0.971% - 9.59%)
Namibia	0.838(0.804 - 0.874)	0.0556(0.0232 - 0.0884)	7.07% (2.85% - 11.4%)	0.751(0.716 - 0.787)	0.0383(0.00446 - 0.0689)	5.34% (0.62% - 9.86%)
South Africa	0.864(0.835 - 0.896)	0.0466(0.0327 - 0.0619)	5.7% (3.97% - 7.76%)	0.767(0.744 - 0.793)	0.03(0.0175 - 0.0433)	4.08% (2.34% - 5.96%)
Zimbabwe	0.787(0.754 - 0.822)	0.0274(-0.0049 - 0.0597)	3.61% (-0.628% - 8.11%)	0.71(0.678 - 0.743)	0.0162(-0.0128 - 0.0453)	2.34% (-1.8% - 6.72%)
Western sub-Saharan Africa	0.809(0.782 - 0.838)	0.0299(0.0237 - 0.0361)	3.84% (3.05% - 4.62%)	0.705(0.678 - 0.731)	0.03(0.0244 - 0.0358)	4.45% (3.62% - 5.27%)
Benin	0.79(0.753 - 0.824)	0.0587(0.025 - 0.0897)	8.03% (3.41% - 12.5%)	0.691(0.658 - 0.725)	0.0405(0.00972 - 0.069)	6.23% (1.44% - 10.7%)
Burkina Faso	0.781(0.747 - 0.816)	0.0389(0.00641 - 0.0714)	5.24% (0.841% - 9.92%)	0.688(0.655 - 0.721)	0.0316(0.00216 - 0.0592)	4.82% (0.317% - 9.24%)
Cabo Verde	0.872(0.836 - 0.908)	0.106(0.0715 - 0.141)	13.9% (9.15% - 18.8%)	0.749(0.715 - 0.784)	0.0752(0.0443 - 0.106)	11.1% (6.39% - 16.1%)
Cameroon	0.825(0.789 - 0.862)	0.0503(0.018 - 0.0834)	6.49% (2.29% - 11%)	0.719(0.683 - 0.754)	0.0359(0.0038 - 0.0669)	5.27% (0.546% - 10.1%)
Chad	0.756(0.724 - 0.79)	0.0168(-0.0133 - 0.05)	2.27% (-1.77% - 6.86%)	0.662(0.629 - 0.694)	0.0104(-0.0175 - 0.0396)	1.59% (-2.67% - 6.17%)
Côte d'Ivoire	0.825(0.789 - 0.861)	0.0332(0.00187 - 0.0688)	4.2% (0.237% - 8.66%)	0.714(0.678 - 0.752)	0.0261(-0.00546 - 0.0562)	3.79% (-0.787% - 8.41%)
The Gambia	0.797(0.76 - 0.832)	0.0487(0.019 - 0.0807)	6.51% (2.48% - 10.9%)	0.695(0.663 - 0.733)	0.042(0.0127 - 0.0747)	6.43% (1.92% - 11.6%)
Ghana	0.833(0.798 - 0.869)	0.0526(0.0193 - 0.0841)	6.77% (2.43% - 10.9%)	0.728(0.693 - 0.764)	0.045(0.0151 - 0.0757)	6.62% (2.18% - 11.4%)
Guinea	0.756(0.723 - 0.789)	0.0241(-0.00665 - 0.0539)	3.28% (-0.899% - 7.45%)	0.668(0.634 - 0.703)	0.0196(-0.00704 - 0.0468)	3.02% (-1.06% - 7.4%)
Guinea-Bissau	0.792(0.759 - 0.828)	0.0228(-0.00799 - 0.0545)	2.96% (-1.03% - 7.27%)	0.695(0.662 - 0.731)	0.0172(-0.0123 - 0.0479)	2.54% (-1.75% - 7.19%)
Liberia	0.83(0.794 - 0.865)	0.0431(0.00982 - 0.0758)	5.46% (1.24% - 9.83%)	0.723(0.687 - 0.761)	0.0328(0.00112 - 0.065)	4.74% (0.156% - 9.41%)
Mali	0.775(0.741 - 0.811)	0.017(-0.0158 - 0.0496)	2.24% (-1.97% - 6.69%)	0.679(0.642 - 0.713)	0.0098(-0.0186 - 0.0365)	1.46% (-2.69% - 5.61%)
Mauritania	0.827(0.792 - 0.864)	0.0387(0.00752 - 0.0705)	4.93% (0.944% - 9.18%)	0.725(0.688 - 0.761)	0.0341(0.00416 - 0.0633)	4.96% (0.594% - 9.44%)
Niger	0.766(0.731 - 0.801)	0.0131(-0.0213 - 0.0486)	1.74% (-2.68% - 6.58%)	0.67(0.636 - 0.705)	0.0078(-0.0218 - 0.0366)	1.17% (-3.29% - 5.61%)
Nigeria	0.822(0.795 - 0.851)	0.0224(0.0157 - 0.0295)	2.8% (1.97% - 3.68%)	0.714(0.691 - 0.739)	0.0315(0.0259 - 0.0368)	4.62% (3.77% - 5.44%)
São Tomé and Príncipe	0.841(0.808 - 0.875)	0.059(0.0242 - 0.0913)	7.55% (3.01% - 11.9%)	0.729(0.694 - 0.763)	0.0472(0.0167 - 0.0765)	6.92% (2.36% - 11.2%)
Senegal	0.807(0.774 - 0.841)	0.0542(0.021 - 0.0837)	7.2% (2.73% - 11.3%)	0.702(0.668 - 0.737)	0.0419(0.0124 - 0.0727)	6.34% (1.87% - 11.1%)
Sierra Leone	0.79(0.757 - 0.824)	0.0392(0.00913 - 0.0702)	5.21% (1.18% - 9.52%)	0.688(0.651 - 0.723)	0.0258(-0.00199 - 0.0531)	3.89% (-0.295% - 8.22%)
Togo	0.785(0.752 - 0.82)	0.0513(0.0208 - 0.0842)	6.99% (2.75% - 11.6%)	0.684(0.651 - 0.717)	0.0377(0.00863 - 0.066)	5.83% (1.3% - 10.6%)

Supplemental Table 3. Percentage of fall and road injury-related all-ages burden attributable to low bone mineral density in 1990 and 2020, by country

Location	YLDs (Years Lived with Disability)				DALYs (Disability-Adjusted Life Years)				Deaths			
	Falls		Road injuries		Falls		Road injuries		Falls		Road injuries	
	1990	2020	1990	2020	1990	2020	1990	2020	1990	2020	1990	2020
Global	19.62% (17.12%–21.21%)	26.57% (23.16%–28.68%)	9.90% (8.48%–10.63%)	12.62% (10.82%–13.48%)	16.70% (14.63%–18.60%)	25.57% (22.13%–27.39%)	3.94% (3.43%–4.30%)	6.33% (5.35%–6.94%)	29.53% (25.99%–32.35%)	40.63% (35.37%–43.98%)	5.91% (5.13%–6.33%)	8.94% (7.55%–9.63%)
Central Asia	11.58% (10.01%–12.49%)	13.78% (11.82%–14.94%)	8.83% (7.40%–9.59%)	10.32% (8.62%–11.19%)	7.74% (6.77%–8.72%)	11.48% (9.84%–12.57%)	3.58% (2.98%–3.95%)	5.66% (4.67%–6.26%)	10.53% (9.19%–11.48%)	17.23% (14.52%–19.25%)	5.37% (4.45%–5.86%)	7.56% (6.24%–8.30%)
Armenia	14.10% (12.24%–15.16%)	21.79% (18.84%–23.60%)	10.20% (8.61%–10.98%)	12.84% (10.71%–13.86%)	10.62% (9.41%–11.57%)	19.25% (16.83%–20.81%)	4.63% (3.93%–5.08%)	7.53% (6.28%–8.34%)	15.48% (13.69%–16.65%)	28.71% (25.00%–31.47%)	6.79% (5.76%–7.35%)	10.41% (8.68%–11.52%)
Azerbaijan	11.20% (9.83%–12.11%)	13.68% (11.85%–14.82%)	9.09% (7.58%–9.86%)	10.62% (8.87%–11.50%)	7.63% (6.61%–9.06%)	11.36% (9.70%–12.83%)	3.87% (3.17%–4.39%)	6.12% (4.90%–6.90%)	10.30% (8.57%–13.59%)	14.28% (11.07%–18.77%)	5.47% (4.49%–6.22%)	8.26% (6.54%–9.36%)
Georgia	15.42% (13.36%–16.61%)	22.22% (19.04%–23.91%)	11.35% (9.53%–12.28%)	13.55% (11.23%–14.70%)	12.05% (10.58%–13.07%)	21.89% (18.82%–23.41%)	5.60% (4.74%–6.19%)	8.19% (6.84%–9.02%)	16.69% (14.41%–18.07%)	35.91% (30.91%–39.23%)	7.99% (6.70%–8.74%)	11.17% (9.35%–12.29%)
Kazakhstan	12.01% (10.32%–13.01%)	15.41% (13.12%–16.65%)	9.05% (7.57%–9.85%)	11.06% (9.14%–12.01%)	8.50% (7.44%–9.44%)	12.99% (11.20%–14.14%)	3.73% (3.03%–4.16%)	6.07% (4.93%–6.80%)	11.17% (9.52%–12.31%)	16.53% (14.10%–18.08%)	5.40% (4.38%–6.10%)	7.85% (6.36%–8.85%)
Kyrgyzstan	11.17% (9.76%–12.17%)	12.63% (11.03%–13.70%)	8.93% (7.48%–9.67%)	10.34% (8.81%–11.19%)	7.24% (6.26%–8.15%)	10.08% (9.01%–11.02%)	3.35% (2.82%–3.72%)	5.37% (4.57%–5.93%)	9.51% (8.22%–10.32%)	14.10% (12.59%–15.39%)	5.04% (4.24%–5.57%)	7.12% (6.11%–7.80%)
Mongolia	10.86% (9.31%–11.87%)	12.70% (11.00%–13.76%)	6.93% (5.75%–7.55%)	10.14% (8.46%–10.94%)	5.84% (4.57%–7.37%)	10.19% (8.72%–11.25%)	2.80% (2.28%–3.27%)	6.16% (5.14%–7.13%)	8.83% (7.07%–11.33%)	12.43% (9.86%–14.27%)	4.40% (3.68%–5.16%)	7.72% (6.42%–8.94%)
Tajikistan	9.98% (8.74%–10.81%)	9.97% (8.66%–10.80%)	8.32% (6.98%–9.02%)	9.31% (7.91%–10.08%)	5.78% (4.58%–7.72%)	6.36% (4.83%–7.75%)	3.16% (2.68%–3.68%)	4.78% (3.93%–5.57%)	7.56% (6.04%–11.89%)	8.07% (5.73%–11.20%)	4.65% (3.95%–5.33%)	6.62% (5.36%–7.48%)
Turkmenistan	8.80% (7.67%–9.53%)	12.55% (10.90%–13.63%)	7.09% (6.00%–7.70%)	9.29% (7.76%–10.11%)	5.48% (4.81%–6.12%)	9.71% (8.28%–10.85%)	2.67% (2.25%–2.95%)	4.71% (3.93%–5.29%)	6.89% (6.09%–7.57%)	11.50% (9.81%–12.84%)	4.05% (3.41%–4.46%)	6.55% (5.44%–7.25%)
Uzbekistan	9.54% (8.26%–10.32%)	10.62% (9.06%–11.56%)	7.82% (6.51%–8.52%)	9.26% (7.75%–10.11%)	5.83% (4.98%–6.50%)	8.98% (7.73%–9.92%)	2.85% (2.37%–3.19%)	5.01% (4.04%–5.60%)	7.90% (6.81%–8.84%)	12.08% (10.09%–13.30%)	4.53% (3.77%–4.96%)	6.64% (5.33%–7.35%)
Central Europe	22.62% (19.48%–24.56%)	27.65% (23.39%–30.03%)	11.25% (9.49%–12.24%)	13.91% (11.54%–15.17%)	22.56% (19.30%–24.27%)	27.78% (23.53%–29.89%)	6.05% (5.12%–6.70%)	8.86% (7.38%–9.91%)	37.67% (32.30%–40.70%)	41.21% (35.22%–45.25%)	8.26% (6.84%–9.02%)	11.40% (9.37%–12.57%)
Albania	12.40% (10.77%–13.55%)	21.68% (18.55%–23.38%)	7.99% (6.85%–8.77%)	12.77% (10.67%–13.92%)	12.01% (10.52%–13.78%)	21.90% (18.96%–23.75%)	3.37% (2.73%–3.85%)	8.30% (6.75%–9.37%)	25.38% (21.72%–29.61%)	37.93% (32.22%–43.60%)	4.69% (3.81%–5.25%)	10.76% (8.64%–12.04%)
Bosnia and Herzegovina	16.05% (13.89%–17.39%)	24.22% (20.84%–26.16%)	10.06% (8.47%–10.95%)	13.81% (11.60%–14.96%)	15.93% (13.67%–17.46%)	24.47% (21.03%–26.46%)	5.84% (4.54%–6.65%)	8.73% (7.23%–9.88%)	26.09% (21.45%–30.36%)	37.51% (32.58%–41.48%)	8.28% (6.60%–9.26%)	11.38% (9.43%–12.70%)
Bulgaria	18.23% (15.80%–19.58%)	24.62% (21.13%–26.39%)	12.10% (9.99%–13.16%)	14.23% (11.88%–15.50%)	17.27% (14.87%–18.60%)	24.38% (20.87%–26.16%)	6.43% (5.37%–7.20%)	8.71% (7.31%–9.80%)	24.87% (21.70%–26.88%)	36.56% (31.76%–40.03%)	8.37% (6.91%–9.41%)	11.00% (9.13%–12.18%)
Croatia	26.15% (22.58%–28.47%)	35.38% (29.86%–38.75%)	11.31% (9.60%–12.36%)	13.85% (11.35%–15.17%)	26.98% (23.35%–29.11%)	36.93% (31.32%–40.07%)	6.85% (5.75%–7.61%)	9.53% (7.87%–10.65%)	42.63% (37.01%–45.95%)	49.92% (42.27%–54.65%)	9.29% (7.80%–10.17%)	12.81% (10.61%–14.19%)
Czechia	30.31% (26.20%–33.11%)	30.50% (25.92%–33.36%)	11.80% (9.94%–12.92%)	14.24% (11.87%–15.64%)	31.77% (27.34%–34.39%)	31.67% (27.01%–34.36%)	6.90% (5.76%–7.67%)	9.64% (8.21%–10.80%)	46.77% (40.23%–50.67%)	45.67% (39.09%–50.13%)	9.67% (7.95%–10.62%)	12.63% (10.62%–13.97%)
Hungary	31.21% (26.88%–34.02%)	32.61% (27.52%–35.46%)	11.87% (9.86%–12.91%)	14.65% (11.77%–16.01%)	30.67% (26.28%–33.02%)	32.93% (27.76%–35.48%)	6.77% (5.63%–7.45%)	9.95% (7.99%–11.09%)	43.67% (37.52%–47.17%)	44.76% (38.04%–49.16%)	9.31% (7.61%–10.19%)	12.52% (10.07%–13.86%)
Montenegro	17.54% (14.95%–19.10%)	22.88% (19.35%–24.68%)	10.38% (8.64%–11.38%)	13.12% (10.89%–14.28%)	17.40% (14.81%–19.06%)	22.74% (19.31%–24.84%)	4.89% (3.88%–5.53%)	7.97% (6.43%–8.95%)	31.07% (26.16%–34.13%)	35.09% (29.93%–39.36%)	6.40% (5.09%–7.17%)	10.33% (8.29%–11.52%)
North Macedonia	15.86% (13.81%–17.25%)	22.57% (19.34%–24.50%)	9.83% (8.40%–10.75%)	12.57% (10.24%–13.70%)	16.50% (14.35%–17.98%)	23.84% (20.62%–26.30%)	5.03% (4.21%–5.69%)	8.55% (6.81%–9.63%)	32.71% (28.09%–35.79%)	40.05% (33.35%–44.55%)	6.96% (5.84%–7.79%)	11.26% (9.00%–12.50%)
Poland	22.49% (19.52%–24.67%)	26.95% (22.98%–29.36%)	11.04% (9.32%–11.97%)	13.69% (11.44%–14.89%)	22.47% (19.40%–24.31%)	26.74% (22.79%–28.92%)	5.65% (4.76%–6.25%)	8.33% (6.94%–9.29%)	38.12% (33.04%–41.17%)	40.40% (34.46%–44.69%)	7.79% (6.51%–8.49%)	10.66% (8.76%–11.73%)
Romania	17.38% (14.85%–18.75%)	24.94% (21.07%–26.90%)	11.60% (9.72%–12.64%)	14.27% (11.83%–15.62%)	15.05% (13.04%–16.24%)	23.82% (20.17%–25.56%)	6.47% (5.45%–7.15%)	9.16% (7.55%–10.36%)	19.75% (16.97%–21.34%)	31.80% (27.39%–34.31%)	8.73% (7.20%–9.52%)	11.77% (9.52%–13.09%)
Serbia	19.44% (16.70%–21.00%)	26.18% (22.36%–28.29%)	10.73% (9.14%–11.72%)	14.02% (11.70%–15.32%)	19.26% (16.30%–21.16%)	26.83% (23.00%–29.15%)	5.33% (4.37%–6.04%)	9.35% (7.76%–10.61%)	32.23% (27.26%–36.15%)	40.11% (34.67%–44.59%)	6.71% (5.62%–7.66%)	12.03% (10.06%–13.46%)
Slovakia	22.42% (18.46%–24.55%)	25.40% (20.78%–28.13%)	10.45% (8.26%–11.58%)	12.83% (10.06%–14.39%)	21.72% (17.70%–23.91%)	24.98% (20.01%–27.55%)	5.56% (4.32%–6.40%)	7.97% (6.25%–9.02%)	34.04% (27.31%–38.81%)	36.43% (29.33%–41.67%)	7.45% (5.80%–8.41%)	10.20% (7.86%–11.55%)
Slovenia	26.38% (22.78%–28.72%)	34.02% (29.06%–37.31%)	11.13% (9.43%–12.20%)	14.02% (11.27%–15.34%)	25.58% (21.93%–27.61%)	36.31% (31.07%–39.55%)	6.47% (5.45%–7.19%)	10.83% (8.58%–12.02%)	38.67% (33.30%–41.70%)	51.16% (42.63%–56.67%)	8.73% (7.33%–9.67%)	14.29% (11.50%–15.84%)
Eastern Europe	17.99% (15.52%–19.35%)	22.02% (18.93%–23.70%)	10.89% (9.20%–11.79%)	12.89% (10.81%–13.96%)	15.82% (13.86%–17.07%)	20.68% (17.94%–22.20%)	5.24% (4.41%–5.80%)	7.57% (6.39%–8.40%)	20.95% (18.25%–22.63%)	28.84% (25.01%–31.52%)	6.87% (5.74%–7.45%)	8.88% (7.42%–9.75%)
Belarus	17.96% (15.48%–19.26%)	22.70% (19.53%–24.45%)	10.84% (9.15%–11.79%)	13.10% (11.04%–14.24%)	15.09% (13.32%–16.46%)	20.85% (17.99%–22.25%)	5.13% (4.33%–5.74%)	8.45% (7.03%–9.38%)	18.06% (15.77%–19.71%)	26.36% (22.46%–28.66%)	6.91% (5.80%–7.55%)	10.34% (8.56%–11.40%)
Estonia	20.95% (18.20%–22.53%)	25.60% (21.97%–27.71%)	11.07% (9.41%–12.01%)	14.22% (11.89%–15.51%)	17.96% (15.77%–19.39%)	24.87% (21.39%–26.84%)	5.43% (4.59%–6.13%)	10.20% (8.62%–11.29%)	24.05% (20.66%–26.15%)	36.30% (31.14%–40.17%)	7.27% (6.24%–8.06%)	13.25% (11.06%–14.61%)
Latvia	21.98% (18.80%–23.78%)	27.05% (23.23%–29.24%)	11.37% (9.44%–12.41%)	14.20% (11.84%–15.47%)	19.02% (16.44%–20.51%)	25.77% (22.39%–27.75%)	5.66% (4.66%–6.30%)	9.84% (8.14%–10.95%)	26.98% (23.18%–29.33%)	36.88% (32.03%–40.80%)	7.65% (6.37%–8.42%)	12.61% (10.53%–13.81%)
Lithuania	20.31% (17.55%–21.94%)	28.97% (24.27%–31.44%)	11.00% (9.27%–12.01%)	14.26% (11.57%–15.66%)	16.77% (14.56%–18.28%)	27.00% (22.92%–29.23%)	5.71% (4.64%–6.37%)	9.30% (7.60%–10.46%)	20.57% (17.65%–22.23%)	36.10% (30.78%–39.79%)	7.89% (6.54%–8.68%)	11.95% (9.72%–13.23%)
Moldova	17.83% (15.33%–19.18%)	22.29% (19.10%–23.92%)	9.74% (8.05%–10.56%)	12.22% (10.21%–13.28%)	14.96% (12.94%–16.19%)	20.38% (17.70%–21.83%)	4.37% (3.66%–4.89%)	7.59% (6.35%–8.50%)	19.35% (16.90%–20.79%)	26.54% (23.24%–28.81%)	5.88% (4.94%–6.44%)	9.60% (8.03%–10.57%)
Russia	17.38% (14.93%–18.74%)	22.13% (18.97%–23.85%)	10.70% (9.03%–11.58%)	12.92% (10.77%–14.01%)	15.46% (13.48%–16.67%)	20.78% (17.96%–22.34%)	5.15% (4.33%–5.69%)	7.52% (6.35%–8.35%)	22.06% (19.21%–23.89%)	29.60% (25.46%–32.22%)	6.69% (5.56%–7.25%)	8.81% (7.33%–9.68%)
Ukraine	18.75% (16.35%–20.13%)	20.81% (18.09%–22.28%)	11.58% (9.82%–12.53%)	12.70% (10.74%–13.74%)	16.37% (14.47%–17.72%)	19.48% (17.17%–21.01%)	5.54% (4.65%–6.18%)	7.46% (6.32%–8.25%)	18.36% (15.99%–19.75%)	24.22% (21.34%–27.13%)	7.33% (6.14%–8.06%)	8.52% (7.20%–9.45%)
Australasia	22.83% (19.82%–25.05%)	31.30% (26.83%–34.19%)	11.13% (9.48%–11.92%)	14.17% (11.96%–15.29%)	24.15% (21.03%–26.21%)	33.99% (29.11%–36.90%)	4.80% (4.09%–5.35%)	8.48% (7.33%–9.39%)	46.47% (40.54%–50.04%)	53.53% (45.80%–58.30%)	6.51% (5.53%–7.07%)	10.85% (9.23%–11.88%)
Australia	22.55% (19.57%–24.69%)	31.84% (27.30%–34.82%)	11.13% (9.48%–11.93%)	14.17% (11.92%–15.28%)	23.92% (20.81%–26.02%)	34.55% (29.64%–37.48%)	4.95% (4.20%–5.52%)	8.60% (7.45%–9.55%)	46.34% (40.11%–49.91%)	53.58% (45.93%–58.27%)	6.86% (5.81%–7.48%)	11.08% (9.47%–12.17%)
New Zealand	24.02% (20.89%–26.40%)	28.62% (24.46%–31.33%)	11.13% (9.50%–11.96%)	14.17% (12.07%–15.25%)	25.06% (21.94%–27.23%)	31.05% (26.55%–33.97%)	4.30% (3.67%–4.85%)	8.06% (6.90%–8.95%)	46.95% (41.61%–50.48%)	53.17% (44.94%–58.14%)	5.32% (4.53%–5.74%)	9.99% (8.33%–10.89%)

High-income Asia Pacific	21.19% (18.75%–22.62%)	34.00% (29.47%–36.55%)	11.62% (10.07%–12.43%)	16.78% (14.33%–18.01%)	19.80% (17.74%–21.42%)	34.84% (30.20%–37.38%)	6.51% (5.63%–7.15%)	13.83% (11.69%–15.03%)	29.37% (25.61%–33.70%)	49.37% (43.42%–53.65%)	8.89% (7.61%–9.54%)	17.76% (14.96%–19.35%)
Brunei	9.86% (8.60%–10.71%)	15.60% (13.55%–16.79%)	6.68% (5.73%–7.16%)	10.05% (8.63%–10.75%)	9.02% (7.95%–9.75%)	14.79% (12.81%–15.91%)	2.83% (2.44%–3.21%)	6.60% (5.64%–7.30%)	17.04% (14.41%–18.91%)	23.59% (19.58%–26.40%)	3.84% (3.25%–4.33%)	8.15% (6.84%–8.90%)
Japan	24.13% (21.34%–25.81%)	37.02% (32.29%–39.66%)	13.34% (11.64%–14.24%)	18.06% (15.54%–19.27%)	23.82% (20.94%–25.38%)	38.34% (33.49%–40.96%)	7.77% (6.79%–8.50%)	15.08% (13.12%–16.30%)	36.19% (32.15%–38.72%)	52.08% (45.47%–56.28%)	10.49% (9.11%–11.22%)	19.31% (16.71%–20.90%)
South Korea	14.75% (12.98%–15.89%)	29.27% (25.13%–31.59%)	9.38% (8.06%–10.10%)	15.34% (12.93%–16.60%)	12.89% (11.25%–15.48%)	29.07% (24.70%–31.48%)	5.33% (4.55%–5.84%)	12.78% (10.51%–13.95%)	18.81% (14.91%–28.52%)	41.41% (34.93%–46.29%)	7.32% (6.17%–8.02%)	16.50% (13.71%–18.10%)
Singapore	14.09% (12.35%–15.18%)	22.59% (19.84%–24.11%)	9.13% (7.98%–9.72%)	12.58% (10.93%–13.41%)	13.52% (12.01%–14.44%)	23.12% (20.36%–24.63%)	5.04% (4.37%–5.52%)	9.98% (8.82%–10.83%)	23.51% (20.85%–24.99%)	39.43% (35.43%–42.14%)	7.09% (6.17%–7.70%)	12.07% (10.66%–12.98%)
High-income North America	28.44% (24.79%–30.85%)	38.30% (33.19%–40.96%)	11.31% (9.56%–12.19%)	14.35% (12.24%–15.41%)	28.23% (24.49%–30.40%)	38.71% (33.62%–41.36%)	5.32% (4.56%–5.93%)	8.71% (7.55%–9.54%)	43.81% (38.65%–47.14%)	50.22% (43.62%–54.39%)	7.00% (5.93%–7.60%)	10.75% (9.19%–11.65%)
Canada	30.76% (26.43%–33.45%)	39.49% (33.49%–42.76%)	10.94% (9.23%–11.81%)	13.98% (11.51%–15.20%)	31.20% (26.99%–33.64%)	40.58% (34.50%–43.87%)	5.18% (4.41%–5.78%)	9.15% (7.60%–10.19%)	47.37% (41.30%–51.09%)	52.46% (44.27%–57.44%)	6.90% (5.82%–7.44%)	11.64% (9.55%–12.77%)
Greenland	21.80% (18.88%–23.94%)	29.29% (25.39%–31.57%)	7.87% (6.59%–8.49%)	11.55% (9.69%–12.49%)	19.02% (15.85%–21.53%)	29.19% (24.80%–31.85%)	2.94% (2.21%–3.59%)	6.78% (5.24%–7.78%)	29.95% (24.50%–34.48%)	41.93% (35.39%–46.59%)	3.70% (2.76%–4.51%)	8.92% (6.94%–10.24%)
USA	28.25% (24.67%–30.67%)	38.07% (33.08%–40.68%)	11.36% (9.60%–12.25%)	14.29% (12.21%–15.34%)	27.95% (24.29%–30.08%)	38.42% (33.46%–41.01%)	5.33% (4.58%–5.95%)	8.67% (7.53%–9.49%)	43.26% (38.23%–46.56%)	49.86% (43.52%–53.91%)	7.01% (5.94%–7.61%)	10.71% (9.17%–11.60%)
Southern Latin America	21.40% (18.59%–23.26%)	24.77% (21.57%–26.79%)	10.90% (9.25%–11.68%)	12.61% (10.78%–13.54%)	20.94% (18.35%–22.57%)	25.61% (22.21%–27.63%)	6.04% (5.15%–6.60%)	6.96% (5.91%–7.59%)	37.14% (33.02%–39.95%)	45.34% (39.60%–49.29%)	8.47% (7.20%–9.14%)	9.46% (8.00%–10.25%)
Argentina	22.36% (19.51%–24.46%)	23.96% (20.89%–26.00%)	11.12% (9.42%–11.90%)	12.43% (10.64%–13.35%)	21.92% (19.26%–23.63%)	24.66% (21.51%–26.66%)	6.14% (5.22%–6.72%)	6.64% (5.63%–7.27%)	38.52% (34.21%–41.49%)	44.22% (38.59%–48.18%)	8.66% (7.34%–9.37%)	8.98% (7.60%–9.79%)
Chile	18.15% (15.65%–19.74%)	25.12% (21.74%–27.26%)	9.97% (8.43%–10.70%)	12.72% (10.75%–13.66%)	17.69% (15.36%–19.01%)	26.04% (22.44%–28.06%)	5.56% (4.76%–6.09%)	7.63% (6.39%–8.39%)	32.40% (28.65%–34.79%)	45.69% (40.03%–49.46%)	7.70% (6.54%–8.39%)	10.15% (8.44%–11.00%)
Uruguay	26.24% (23.05%–28.52%)	29.99% (25.82%–32.59%)	12.30% (10.56%–13.19%)	14.23% (12.13%–15.29%)	26.10% (22.95%–27.97%)	31.37% (26.87%–33.96%)	7.12% (6.14%–7.73%)	8.05% (6.87%–8.86%)	42.14% (37.33%–45.11%)	50.33% (43.35%–54.90%)	9.92% (8.52%–10.67%)	11.62% (9.93%–12.68%)
Western Europe	27.75% (23.85%–30.35%)	33.52% (28.16%–36.66%)	12.38% (10.44%–13.41%)	14.88% (12.38%–16.14%)	28.98% (24.71%–31.43%)	35.67% (30.09%–38.74%)	6.27% (5.36%–6.98%)	10.60% (8.91%–11.68%)	46.82% (40.04%–51.07%)	51.48% (43.09%–56.59%)	8.62% (7.26%–9.42%)	14.00% (11.68%–15.44%)
Andorra	21.63% (18.56%–23.47%)	32.21% (26.98%–35.24%)	10.09% (8.43%–10.89%)	14.15% (11.79%–15.34%)	22.33% (19.37%–24.44%)	33.31% (27.93%–36.33%)	4.84% (3.93%–5.52%)	9.32% (7.88%–10.32%)	39.87% (35.12%–44.02%)	51.69% (42.74%–57.52%)	6.47% (5.42%–7.35%)	11.89% (10.04%–13.18%)
Austria	27.47% (23.76%–29.86%)	32.24% (27.59%–34.80%)	12.04% (10.16%–12.97%)	14.63% (12.29%–15.75%)	28.50% (24.48%–30.77%)	34.04% (29.26%–36.56%)	6.29% (5.35%–6.99%)	10.59% (8.93%–11.62%)	45.71% (39.59%–49.37%)	50.42% (43.33%–55.06%)	8.78% (7.44%–9.53%)	13.92% (11.68%–15.19%)
Belgium	28.63% (24.68%–31.02%)	35.70% (30.58%–38.83%)	12.22% (10.37%–13.14%)	14.69% (12.26%–15.81%)	29.29% (25.16%–31.61%)	37.00% (31.67%–39.93%)	6.22% (5.33%–6.92%)	10.42% (8.78%–11.55%)	46.65% (40.12%–50.39%)	51.28% (43.24%–55.99%)	8.49% (7.10%–9.22%)	13.42% (11.35%–14.79%)
Cyprus	23.26% (20.29%–25.28%)	26.81% (23.29%–29.17%)	11.17% (9.44%–12.03%)	12.64% (10.71%–13.60%)	25.90% (22.49%–28.29%)	29.00% (24.93%–31.70%)	6.15% (5.22%–6.79%)	8.44% (7.17%–9.37%)	44.10% (38.25%–48.45%)	48.20% (41.91%–52.57%)	8.63% (7.37%–9.54%)	11.73% (9.98%–12.92%)
Denmark	33.51% (28.42%–36.84%)	32.10% (26.82%–35.23%)	12.70% (10.61%–13.82%)	14.37% (11.81%–15.57%)	35.35% (30.12%–38.55%)	33.86% (28.38%–37.04%)	7.46% (6.27%–8.22%)	10.51% (8.79%–11.64%)	49.90% (42.44%–54.56%)	49.59% (41.05%–55.06%)	10.42% (8.66%–11.29%)	13.84% (11.45%–15.16%)
Finland	26.61% (22.90%–28.85%)	33.76% (28.93%–36.53%)	12.71% (10.82%–13.74%)	14.96% (12.60%–16.22%)	26.69% (22.93%–28.68%)	34.77% (29.98%–37.44%)	7.28% (6.17%–8.01%)	10.63% (9.12%–11.73%)	41.68% (36.19%–45.14%)	49.09% (42.55%–53.25%)	9.96% (8.38%–10.75%)	14.08% (11.90%–15.55%)
France	28.94% (24.57%–31.97%)	34.79% (28.72%–38.34%)	11.94% (9.87%–13.00%)	14.30% (11.65%–15.69%)	30.42% (25.56%–33.29%)	36.29% (30.07%–39.89%)	5.88% (4.93%–6.58%)	8.85% (7.33%–9.97%)	48.07% (40.25%–52.86%)	51.21% (41.39%–57.27%)	7.94% (6.49%–8.80%)	11.26% (9.19%–12.63%)
Germany	28.40% (24.38%–31.15%)	34.70% (28.88%–37.88%)	12.01% (9.99%–13.05%)	14.74% (12.04%–16.12%)	29.72% (25.36%–32.28%)	37.16% (31.07%–40.59%)	6.19% (5.21%–6.95%)	10.72% (8.85%–11.94%)	46.89% (40.44%–51.16%)	50.84% (42.45%–55.83%)	8.61% (7.19%–9.49%)	13.71% (11.25%–15.20%)
Greece	24.47% (21.52%–26.22%)	30.25% (26.31%–32.37%)	13.25% (11.45%–14.06%)	15.91% (13.74%–16.92%)	24.85% (21.86%–26.50%)	32.96% (28.71%–35.47%)	7.08% (6.22%–7.74%)	10.92% (9.46%–11.95%)	42.18% (38.06%–44.83%)	52.34% (46.01%–56.04%)	9.59% (8.38%–10.27%)	13.82% (11.99%–14.92%)
Iceland	23.13% (19.84%–25.13%)	27.64% (23.35%–30.19%)	11.13% (9.46%–12.08%)	13.24% (11.09%–14.48%)	24.46% (20.98%–26.53%)	30.36% (25.84%–33.17%)	4.81% (4.14%–5.35%)	8.91% (7.47%–9.89%)	45.39% (39.13%–49.58%)	50.39% (43.23%–55.33%)	7.05% (5.99%–7.72%)	12.34% (10.32%–13.66%)
Ireland	23.84% (20.69%–26.08%)	26.43% (22.04%–28.90%)	11.12% (9.38%–12.02%)	13.15% (10.94%–14.35%)	25.64% (22.19%–27.88%)	27.99% (23.44%–30.61%)	5.35% (4.58%–5.99%)	9.26% (7.65%–10.36%)	45.38% (39.40%–49.14%)	47.88% (40.95%–52.54%)	7.67% (6.52%–8.36%)	12.07% (9.91%–13.34%)
Israel	20.36% (17.27%–22.33%)	23.55% (20.23%–25.80%)	11.00% (9.23%–11.92%)	13.29% (11.05%–14.42%)	22.12% (18.88%–24.22%)	25.53% (21.98%–27.90%)	5.33% (4.53%–5.95%)	7.99% (6.71%–8.89%)	44.97% (39.01%–48.47%)	49.18% (42.69%–53.97%)	8.14% (6.76%–8.89%)	11.33% (9.49%–12.52%)
Italy	27.69% (23.74%–30.37%)	32.79% (27.86%–35.60%)	13.12% (11.06%–14.23%)	15.75% (13.18%–16.99%)	29.46% (25.12%–31.93%)	35.57% (30.32%–38.51%)	7.27% (6.18%–8.05%)	12.37% (10.42%–13.56%)	48.58% (41.58%–52.90%)	53.76% (45.59%–58.71%)	10.02% (8.45%–10.94%)	17.26% (14.53%–19.06%)
Luxembourg	26.07% (22.23%–28.23%)	28.65% (24.47%–31.33%)	11.62% (9.83%–12.53%)	13.05% (11.00%–14.08%)	26.59% (22.64%–28.59%)	31.13% (26.78%–33.76%)	5.91% (4.93%–6.57%)	9.31% (7.74%–10.34%)	43.09% (37.56%–46.49%)	50.76% (43.59%–55.36%)	7.93% (6.49%–8.63%)	12.51% (10.43%–13.72%)
Malta	23.60% (20.43%–25.64%)	30.45% (25.91%–33.02%)	11.26% (9.54%–12.15%)	13.94% (11.69%–15.13%)	25.48% (21.93%–27.57%)	32.11% (27.41%–34.56%)	6.25% (5.33%–6.98%)	9.43% (7.93%–10.68%)	45.34% (39.48%–48.67%)	49.27% (42.49%–53.46%)	8.39% (7.23%–9.18%)	11.51% (9.58%–12.67%)
Monaco	31.36% (26.65%–34.03%)	32.49% (27.51%–35.35%)	14.14% (11.71%–15.28%)	14.83% (12.25%–16.06%)	32.58% (27.84%–35.40%)	33.92% (28.85%–36.85%)	8.21% (6.70%–9.35%)	9.62% (7.65%–11.19%)	49.59% (42.22%–54.16%)	51.22% (43.72%–56.00%)	10.68% (8.86%–11.87%)	12.01% (9.87%–13.51%)
Netherlands	28.71% (24.74%–31.58%)	38.23% (32.43%–41.60%)	11.81% (10.10%–12.72%)	14.77% (12.42%–15.93%)	30.45% (26.33%–33.03%)	41.05% (35.05%–44.57%)	6.10% (5.28%–6.76%)	10.84% (9.24%–11.88%)	48.85% (41.79%–53.44%)	53.75% (45.34%–58.67%)	8.82% (7.56%–9.50%)	15.33% (13.01%–16.79%)
Norway	32.42% (27.73%–35.54%)	35.09% (29.52%–38.62%)	13.88% (11.93%–14.92%)	13.87% (11.50%–15.05%)	34.99% (29.86%–38.01%)	38.11% (32.31%–41.63%)	7.57% (6.52%–8.45%)	10.09% (8.47%–11.15%)	50.90% (43.72%–55.35%)	53.40% (44.53%–58.85%)	9.74% (8.18%–10.64%)	12.93% (10.66%–14.30%)
Portugal	22.24% (19.43%–23.92%)	32.72% (28.02%–35.27%)	11.57% (9.94%–12.41%)	15.79% (13.19%–17.08%)	22.10% (19.20%–23.70%)	34.37% (29.54%–36.99%)	5.65% (4.89%–6.24%)	11.41% (9.57%–12.55%)	37.17% (32.50%–39.96%)	49.65% (42.81%–53.98%)	7.70% (6.56%–8.38%)	14.94% (12.67%–16.46%)
San Marino	23.98% (20.51%–26.17%)	30.26% (25.53%–32.93%)	12.68% (10.76%–13.74%)	15.48% (13.12%–16.70%)	24.81% (21.17%–27.08%)	31.77% (26.87%–34.54%)	6.57% (5.60%–7.41%)	10.60% (8.74%–11.81%)	43.67% (37.15%–48.19%)	50.62% (42.86%–56.28%)	8.89% (7.45%–9.95%)	14.10% (11.63%–15.88%)
Spain	22.93% (19.99%–24.62%)	31.03% (26.39%–33.61%)	12.49% (10.55%–13.40%)	15.25% (12.89%–16.47%)	21.81% (19.15%–23.30%)	32.17% (27.38%–34.80%)	5.66% (4.77%–6.34%)	11.00% (9.36%–12.00%)	33.80% (29.91%–36.17%)	49.50% (42.82%–53.96%)	7.51% (6.40%–8.15%)	13.59% (11.38%–14.86%)
Sweden	32.10% (27.88%–34.80%)	36.28% (31.48%–39.27%)	14.25% (12.22%–15.34%)	15.23% (13.02%–16.39%)	34.05% (29.40%–36.70%)	38.78% (33.85%–41.80%)	7.86% (6.79%–8.74%)	11.28% (9.72%–12.42%)	50.26% (43.93%–54.05%)	53.85% (46.67%–57.92%)	10.44% (8.92%–11.32%)	14.45% (12.32%–15.68%)
Switzerland	29.23% (25.23%–31.93%)	33.75% (28.69%–36.81%)	12.47% (10.51%–13.47%)	14.54% (12.17%–15.79%)	30.92% (26.62%–33.40%)	35.97% (30.70%–39.20%)	6.33% (5.42%–7.02%)	10.85% (9.12%–11.95%)	48.59% (42.07%–52.84%)	52.90% (44.54%–57.99%)	9.02% (7.62%–9.94%)	14.20% (11.83%–15.62%)
UK	27.70% (23.78%–30.16%)	32.30% (27.22%–35.32%)	12.53% (10.59%–13.59%)	14.11% (11.73%–15.36%)	28.60% (24.45%–30.95%)	34.34% (29.02%–37.28%)	6.14% (5.23%–6.85%)	10.02% (8.38%–11.11%)	46.01% (39.57%–49.95%)	50.53% (42.74%–55.37%)	8.58% (7.18%–9.36%)	12.41% (10.25%–13.72%)
Andean Latin America	12.38% (10.83%–13.52%)	17.26% (14.73%–18.77%)	8.52% (7.20%–9.17%)	11.56% (9.67%–12.51%)	10.65% (9.34%–11.63%)	16.84% (14.41%–18.39%)	3.57% (3.10%–3.98%)	5.64% (4.70%–6.19%)	21.13% (18.49%–23.15%)	30.97% (26.44%–34.66%)	5.98% (5.12%–6.59%)	8.86% (7.31%–9.70%)
Bolivia	12.07% (10.72%–13.19%)	16.75% (14.44%–18.29%)	8.02% (6.85%–8.65%)	11.56% (9.76%–12.50%)	10.01% (8.73%–11.19%)	15.63% (13.24%–18.09%)	3.33% (2.74%–4.06%)	6.41% (5.24%–7.12%)	19.71% (17.17%–21.65%)	27.29% (22.45%–32.03%)	5.94% (4.97%–6.91%)	10.08% (8.26%–11.37%)
Ecuador	12.11% (10.62%–13.14%)	16.55% (14.29%–17.97%)	8.62% (7.21%–9.26%)	10.91% (9.10%–11.79%)	10.80% (9.50%–11.64%)	16.75% (14.52%–18.20%)	3.78% (3.19%–4.13%)	4.87% (4.05%–5.41%)	20.81% (18.15%–22.55%)	31.85% (27.49%–35.45%)	6.17% (5.20%–6.69%)	7.68% (6.43%–8.49%)

Peru	12.67% (11.04%–13.93%)	17.89% (15.22%–19.50%)	8.66% (7.34%–9.32%)	12.19% (10.19%–13.22%)	10.84% (9.45%–12.12%)	17.36% (14.52%–19.11%)	3.58% (2.94%–4.06%)	5.99% (4.96%–6.69%)	22.01% (18.95%–24.97%)	31.71% (25.89%–35.99%)	5.87% (4.88%–6.65%)	9.31% (7.65%–10.31%)
Caribbean	21.04% (18.21%–23.42%)	28.50% (23.92%–31.63%)	8.87% (7.51%–9.51%)	10.68% (8.94%–11.53%)	22.57% (19.78%–24.65%)	30.56% (25.99%–33.70%)	3.21% (2.71%–3.52%)	4.56% (3.80%–5.14%)	40.58% (35.60%–44.47%)	46.10% (38.40%–51.01%)	5.19% (4.39%–5.63%)	6.92% (5.72%–7.62%)
Antigua and Barbuda	18.89% (16.05%–20.84%)	20.52% (17.43%–22.43%)	10.33% (8.73%–11.18%)	12.03% (10.21%–13.00%)	21.80% (18.93%–23.95%)	23.20% (19.75%–25.35%)	5.34% (4.46%–5.86%)	7.09% (5.75%–7.90%)	40.89% (34.94%–44.76%)	40.71% (34.32%–44.70%)	8.11% (6.75%–8.90%)	9.82% (7.94%–10.86%)
The Bahamas	14.70% (12.65%–16.43%)	19.92% (17.00%–21.86%)	8.72% (7.28%–9.45%)	11.44% (9.57%–12.36%)	15.85% (13.67%–17.43%)	21.36% (18.42%–23.43%)	3.82% (3.11%–4.25%)	5.83% (4.89%–6.54%)	32.62% (28.20%–36.19%)	38.36% (32.59%–42.91%)	5.52% (4.51%–6.13%)	7.70% (6.51%–8.53%)
Barbados	22.40% (19.09%–25.14%)	27.32% (22.89%–30.66%)	10.84% (8.88%–11.74%)	13.67% (11.22%–14.81%)	26.48% (22.59%–29.68%)	31.57% (26.68%–35.11%)	4.70% (3.95%–5.22%)	8.22% (6.64%–9.25%)	45.13% (37.72%–51.07%)	46.82% (38.65%–53.31%)	7.48% (6.25%–8.17%)	11.50% (9.34%–12.78%)
Belize	13.31% (11.46%–14.81%)	15.16% (12.85%–16.59%)	7.53% (6.27%–8.12%)	9.67% (8.09%–10.43%)	13.86% (12.15%–15.07%)	16.34% (13.93%–17.91%)	2.49% (2.11%–2.73%)	3.92% (3.24%–4.32%)	34.24% (29.72%–37.28%)	34.81% (30.17%–38.87%)	4.29% (3.62%–4.73%)	5.42% (4.49%–5.92%)
Bermuda	19.42% (16.84%–21.23%)	28.65% (23.97%–31.70%)	10.81% (8.94%–11.71%)	14.47% (11.90%–15.74%)	22.16% (19.17%–24.10%)	31.65% (26.68%–34.78%)	6.26% (5.20%–6.90%)	10.31% (8.22%–11.34%)	39.11% (33.79%–42.65%)	46.89% (39.34%–51.63%)	8.86% (7.45%–9.71%)	13.59% (10.96%–14.91%)
Cuba	26.06% (22.67%–29.17%)	36.00% (29.90%–39.80%)	10.00% (8.54%–10.63%)	14.06% (11.89%–15.17%)	28.67% (25.08%–31.26%)	38.38% (32.22%–42.13%)	4.22% (3.70%–4.63%)	8.88% (7.52%–9.59%)	45.08% (39.25%–48.95%)	49.39% (40.37%–54.69%)	6.29% (5.43%–6.76%)	11.90% (10.01%–12.80%)
Dominica	19.35% (16.82%–21.41%)	21.29% (18.23%–23.45%)	10.76% (9.03%–11.61%)	12.17% (10.23%–13.18%)	22.24% (19.15%–24.03%)	24.89% (21.02%–27.67%)	4.98% (4.22%–5.58%)	6.84% (5.80%–7.77%)	40.37% (34.48%–43.75%)	41.93% (35.25%–46.52%)	7.93% (6.63%–8.87%)	9.89% (8.34%–11.06%)
Dominican Republic	13.06% (11.28%–14.45%)	18.21% (15.51%–20.16%)	8.05% (6.82%–8.67%)	11.03% (9.19%–11.99%)	13.19% (11.23%–16.32%)	19.06% (16.46%–21.14%)	2.77% (2.37%–3.05%)	4.54% (3.58%–5.07%)	31.47% (26.86%–37.27%)	38.73% (32.68%–43.78%)	4.53% (3.88%–4.95%)	6.65% (5.25%–7.42%)
Grenada	19.58% (16.98%–21.58%)	19.48% (16.78%–21.25%)	10.33% (8.65%–11.17%)	11.72% (9.86%–12.71%)	22.68% (19.54%–24.76%)	23.28% (20.15%–25.45%)	3.92% (3.25%–4.38%)	6.62% (5.54%–7.26%)	42.30% (36.23%–46.36%)	39.14% (33.75%–42.79%)	6.38% (5.28%–7.00%)	9.27% (7.74%–10.09%)
Guyana	13.16% (11.31%–14.41%)	16.76% (14.44%–18.39%)	7.61% (6.45%–8.20%)	10.06% (8.30%–10.90%)	12.88% (11.27%–13.90%)	17.45% (14.97%–19.02%)	3.16% (2.71%–3.56%)	4.79% (3.87%–5.42%)	26.55% (23.26%–28.85%)	32.51% (27.65%–36.02%)	4.83% (4.15%–5.46%)	6.52% (5.31%–7.26%)
Haiti	11.93% (10.46%–13.09%)	12.93% (11.14%–14.26%)	6.75% (5.65%–7.34%)	7.62% (6.36%–8.23%)	12.65% (9.48%–14.91%)	14.82% (12.31%–17.32%)	2.07% (1.61%–2.48%)	2.86% (2.17%–3.45%)	26.89% (21.30%–31.57%)	31.31% (26.03%–36.12%)	3.59% (2.81%–4.22%)	4.48% (3.41%–5.35%)
Jamaica	15.86% (13.67%–17.67%)	20.66% (17.43%–23.02%)	9.12% (7.67%–9.92%)	11.68% (9.93%–12.67%)	18.50% (15.98%–20.40%)	24.42% (20.76%–27.09%)	4.50% (3.80%–4.95%)	6.89% (5.78%–7.67%)	42.22% (36.51%–46.52%)	45.64% (37.23%–51.08%)	7.50% (6.23%–8.25%)	9.62% (8.02%–10.55%)
Puerto Rico	20.34% (17.74%–22.33%)	30.06% (25.64%–32.96%)	11.08% (9.27%–11.91%)	14.59% (12.19%–15.86%)	21.50% (18.55%–23.51%)	31.30% (26.78%–34.10%)	5.26% (4.42%–5.76%)	7.85% (6.59%–8.66%)	38.54% (33.01%–42.50%)	45.42% (38.64%–50.47%)	7.86% (6.57%–8.60%)	11.04% (9.10%–12.11%)
Saint Kitts and Nevis	19.81% (17.14%–21.72%)	18.46% (15.74%–20.32%)	10.01% (8.35%–10.86%)	11.39% (9.51%–12.37%)	22.40% (19.77%–24.14%)	21.13% (18.11%–23.25%)	4.60% (3.84%–5.09%)	8.06% (6.39%–9.21%)	38.32% (33.97%–41.27%)	35.67% (30.63%–39.60%)	7.12% (5.86%–7.88%)	10.60% (8.49%–11.98%)
Saint Lucia	15.06% (13.00%–16.75%)	20.66% (17.83%–22.77%)	9.29% (7.77%–10.03%)	12.38% (10.51%–13.37%)	17.27% (14.85%–19.02%)	22.52% (19.46%–24.65%)	3.79% (3.13%–4.19%)	6.78% (5.73%–7.63%)	36.18% (31.20%–39.33%)	40.15% (34.55%–44.35%)	5.95% (4.91%–6.54%)	9.02% (7.59%–9.82%)
Saint Vincent and the Grenadines	14.73% (12.72%–16.24%)	19.86% (17.28%–21.52%)	8.68% (7.31%–9.36%)	12.25% (10.37%–13.23%)	15.73% (13.65%–17.11%)	21.68% (18.80%–23.39%)	3.19% (2.63%–3.59%)	6.85% (5.91%–7.58%)	32.43% (28.25%–35.08%)	37.54% (32.95%–40.17%)	5.04% (4.15%–5.57%)	9.10% (7.73%–9.93%)
Suriname	16.58% (14.22%–18.39%)	21.46% (18.51%–23.65%)	8.96% (7.52%–9.63%)	11.65% (9.78%–12.57%)	18.00% (15.57%–21.37%)	23.34% (20.20%–25.71%)	3.55% (2.91%–4.38%)	5.95% (5.11%–6.71%)	36.66% (31.10%–42.27%)	40.15% (33.85%–44.69%)	5.47% (4.46%–6.58%)	8.20% (7.02%–9.18%)
Trinidad and Tobago	14.89% (12.89%–16.27%)	19.89% (17.22%–21.74%)	8.75% (7.34%–9.44%)	12.09% (10.08%–13.05%)	16.91% (14.36%–18.49%)	22.10% (19.12%–24.15%)	3.96% (3.33%–4.43%)	6.05% (5.03%–6.66%)	34.81% (29.63%–38.06%)	39.06% (33.66%–43.25%)	6.32% (5.21%–6.97%)	8.37% (6.92%–9.22%)
Virgin Islands	16.78% (14.56%–18.42%)	28.08% (23.91%–30.67%)	10.49% (8.68%–11.32%)	14.86% (12.21%–16.10%)	18.19% (15.99%–20.03%)	29.44% (25.13%–32.33%)	4.77% (3.92%–5.35%)	10.39% (8.28%–12.04%)	34.29% (29.61%–38.20%)	41.32% (34.95%–46.34%)	6.70% (5.48%–7.51%)	13.10% (10.60%–14.82%)
Central Latin America	12.46% (10.73%–13.64%)	19.21% (16.65%–20.93%)	8.14% (6.97%–8.75%)	11.53% (9.78%–12.42%)	11.44% (9.97%–12.26%)	19.13% (16.43%–20.69%)	3.35% (2.89%–3.65%)	5.29% (4.42%–5.89%)	23.63% (20.78%–25.32%)	34.26% (29.62%–37.47%)	5.40% (4.61%–5.83%)	7.59% (6.31%–8.25%)
Colombia	12.00% (10.39%–13.12%)	19.57% (16.92%–21.40%)	8.29% (7.05%–8.93%)	11.94% (9.92%–12.96%)	10.98% (9.60%–11.71%)	20.21% (17.41%–21.77%)	3.62% (3.10%–4.02%)	5.30% (4.39%–5.91%)	21.54% (19.09%–23.17%)	37.20% (32.41%–40.97%)	5.82% (4.98%–6.37%)	7.90% (6.56%–8.64%)
Costa Rica	16.31% (13.85%–18.22%)	23.31% (19.77%–25.95%)	8.95% (7.60%–9.58%)	12.00% (10.10%–12.94%)	17.32% (14.93%–18.98%)	24.95% (21.28%–27.32%)	4.21% (3.58%–4.61%)	5.91% (4.97%–6.56%)	36.07% (30.82%–39.22%)	43.33% (37.42%–47.69%)	6.79% (5.76%–7.38%)	8.64% (7.26%–9.48%)
El Salvador	13.41% (11.64%–14.76%)	21.73% (18.78%–24.13%)	8.87% (7.58%–9.54%)	12.72% (10.81%–13.69%)	12.11% (10.44%–14.31%)	21.51% (18.54%–23.56%)	3.52% (2.97%–3.93%)	7.00% (5.89%–7.84%)	25.42% (21.53%–31.32%)	37.59% (31.78%–41.70%)	5.93% (5.10%–6.52%)	10.17% (8.52%–11.15%)
Guatemala	11.22% (9.80%–12.18%)	15.20% (13.14%–16.59%)	8.07% (6.96%–8.65%)	9.49% (8.14%–10.20%)	8.84% (7.81%–9.54%)	14.30% (12.45%–15.41%)	3.20% (2.76%–3.53%)	3.85% (3.28%–4.22%)	17.84% (15.77%–19.23%)	25.91% (22.74%–28.00%)	5.03% (4.27%–5.44%)	5.46% (4.64%–5.95%)
Honduras	10.02% (8.79%–10.99%)	13.84% (12.09%–15.21%)	7.72% (6.69%–8.31%)	11.24% (9.57%–12.07%)	9.42% (8.06%–10.96%)	15.00% (12.44%–17.63%)	2.99% (2.43%–3.45%)	7.40% (5.88%–8.81%)	20.29% (16.70%–24.77%)	29.36% (22.58%–35.08%)	4.92% (4.02%–5.67%)	10.81% (8.67%–12.75%)
Mexico	12.66% (10.88%–13.86%)	19.47% (16.86%–21.26%)	8.10% (6.95%–8.72%)	11.48% (9.71%–12.38%)	11.62% (10.12%–12.41%)	19.31% (16.55%–20.89%)	3.30% (2.83%–3.58%)	5.49% (4.62%–6.10%)	23.90% (21.03%–25.60%)	33.86% (29.09%–36.92%)	5.33% (4.54%–5.74%)	7.66% (6.37%–8.35%)
Nicaragua	10.92% (9.45%–12.13%)	17.34% (14.79%–19.26%)	7.51% (6.50%–8.10%)	10.07% (8.46%–10.84%)	9.55% (8.44%–11.20%)	17.47% (14.85%–19.25%)	2.39% (2.05%–2.70%)	4.37% (3.57%–4.94%)	22.06% (18.71%–25.93%)	33.64% (27.22%–38.04%)	3.97% (3.36%–4.49%)	6.53% (5.37%–7.29%)
Panama	13.68% (11.83%–15.13%)	17.78% (15.47%–19.39%)	9.29% (7.92%–9.98%)	11.87% (9.97%–12.78%)	13.73% (12.05%–14.88%)	18.11% (15.80%–19.50%)	4.04% (3.47%–4.46%)	5.88% (4.90%–6.48%)	29.28% (26.08%–31.54%)	35.27% (30.41%–38.52%)	6.29% (5.39%–6.77%)	8.76% (7.26%–9.50%)
Venezuela	11.90% (10.37%–13.11%)	20.10% (17.52%–21.92%)	7.90% (6.79%–8.48%)	11.89% (10.26%–12.72%)	11.83% (10.43%–12.70%)	19.74% (17.06%–21.37%)	3.28% (2.79%–3.57%)	4.72% (4.01%–5.30%)	26.92% (23.27%–29.20%)	35.57% (30.66%–39.42%)	5.05% (4.29%–5.43%)	6.71% (5.70%–7.36%)
Tropical Latin America	12.36% (10.75%–13.34%)	20.52% (17.72%–22.32%)	8.60% (7.44%–9.23%)	11.54% (9.86%–12.46%)	11.83% (10.39%–12.63%)	22.71% (19.44%–24.63%)	3.95% (3.45%–4.28%)	6.32% (5.38%–6.95%)	23.85% (21.02%–25.66%)	40.95% (34.83%–45.26%)	5.87% (5.07%–6.30%)	8.30% (7.00%–9.04%)
Brazil	12.37% (10.76%–13.36%)	20.62% (17.81%–22.43%)	8.60% (7.45%–9.23%)	11.58% (9.89%–12.49%)	11.82% (10.38%–12.62%)	22.82% (19.54%–24.74%)	3.96% (3.46%–4.29%)	6.39% (5.45%–7.03%)	23.75% (20.92%–25.57%)	40.99% (34.86%–45.29%)	5.88% (5.08%–6.30%)	8.37% (7.07%–9.11%)
Paraguay	11.66% (10.13%–12.87%)	15.62% (13.49%–17.07%)	8.50% (7.25%–9.11%)	10.17% (8.59%–10.98%)	12.11% (10.74%–13.32%)	17.31% (14.63%–19.23%)	3.35% (2.75%–3.75%)	4.52% (3.74%–5.04%)	31.56% (26.40%–35.30%)	38.61% (31.76%–43.42%)	5.53% (4.61%–6.08%)	6.64% (5.52%–7.36%)
North Africa and Middle East	11.42% (10.05%–12.38%)	14.53% (12.73%–15.63%)	7.65% (6.56%–8.20%)	10.39% (8.91%–11.10%)	8.14% (6.44%–10.53%)	12.77% (11.01%–14.03%)	2.96% (2.60%–3.26%)	5.41% (4.62%–5.95%)	18.35% (14.39%–24.40%)	26.96% (22.85%–30.64%)	4.80% (4.22%–5.22%)	7.68% (6.56%–8.30%)
Afghanistan	13.04% (11.59%–14.04%)	9.07% (8.03%–9.87%)	8.81% (7.45%–9.47%)	7.34% (6.29%–7.88%)	6.43% (3.37%–12.19%)	4.71% (3.20%–7.11%)	3.50% (2.76%–4.24%)	3.31% (2.67%–3.88%)	17.73% (11.12%–27.18%)	13.83% (9.89%–18.92%)	5.95% (4.93%–7.13%)	4.97% (4.08%–5.73%)
Algeria	11.03% (9.79%–12.02%)	15.50% (13.63%–16.69%)	7.52% (6.46%–8.10%)	10.98% (9.45%–11.70%)	7.67% (6.26%–9.34%)	13.88% (12.04%–15.20%)	2.48% (2.10%–2.85%)	5.52% (4.68%–6.13%)	13.49% (11.32%–18.38%)	26.09% (21.38%–30.15%)	3.99% (3.26%–4.53%)	8.11% (6.87%–8.87%)
Bahrain	7.45% (6.50%–8.12%)	11.59% (10.01%–12.41%)	6.17% (5.19%–6.65%)	10.03% (8.54%–10.73%)	6.98% (6.12%–7.67%)	11.01% (9.61%–11.81%)	3.78% (3.25%–4.24%)	5.44% (4.59%–6.01%)	14.78% (11.34%–16.65%)	19.69% (15.70%–22.16%)	6.04% (5.19%–6.73%)	7.25% (6.04%–8.06%)
Egypt	11.89% (10.47%–12.89%)	13.68% (11.99%–14.82%)	7.64% (6.49%–8.23%)	10.57% (8.93%–11.33%)	7.96% (5.97%–11.21%)	10.42% (8.62%–13.05%)	2.98% (2.43%–3.45%)	5.20% (4.37%–5.85%)	15.71% (10.67%–24.80%)	18.09% (13.82%–25.89%)	3.64% (3.06%–4.43%)	6.17% (5.23%–6.91%)
Iran	10.38% (9.10%–11.36%)	15.69% (13.92%–16.80%)	7.05% (6.04%–7.61%)	10.97% (9.58%–11.65%)	6.89% (5.70%–8.82%)	15.18% (13.55%–16.64%)	2.50% (2.10%–2.78%)	5.90% (5.17%–6.41%)	12.60% (10.31%–18.68%)	31.39% (27.11%–36.01%)	4.23% (3.56%–4.66%)	8.50% (7.38%–9.12%)
Iraq	10.81% (9.53%–11.68%)	11.87% (10.43%–12.87%)	7.81% (6.62%–8.36%)	10.01% (8.57%–10.70%)	8.16% (6.73%–10.22%)	10.61% (8.94%–11.87%)	3.18% (2.66%–3.62%)	5.47% (4.52%–6.38%)	16.11% (12.32%–24.87%)	20.60% (15.50%–26.94%)	4.95% (4.12%–5.70%)	7.73% (6.37%–8.84%)

Jordan	9.06% (8.08%–9.97%)	12.01% (10.53%–13.00%)	7.25% (6.24%–7.77%)	9.60% (8.27%–10.21%)	7.31% (6.36%–8.23%)	11.20% (9.88%–12.15%)	2.82% (2.47%–3.14%)	4.64% (3.78%–5.16%)	16.98% (14.49%–20.00%)	23.46% (20.36%–26.07%)	4.71% (4.12%–5.22%)	6.97% (5.73%–7.67%)
Kuwait	7.56% (6.62%–8.23%)	10.83% (9.41%–11.67%)	6.57% (5.54%–7.01%)	9.58% (8.26%–10.23%)	6.67% (5.94%–7.26%)	11.06% (9.68%–11.83%)	3.37% (2.89%–3.67%)	5.61% (4.82%–6.23%)	13.92% (12.20%–15.10%)	27.94% (24.02%–30.64%)	5.06% (4.32%–5.50%)	7.96% (6.79%–8.71%)
Lebanon	15.52% (13.86%–16.72%)	19.33% (16.89%–20.91%)	10.70% (9.42%–11.33%)	11.96% (10.45%–12.67%)	15.05% (12.19%–17.74%)	20.21% (17.95%–21.88%)	4.74% (4.12%–5.35%)	6.98% (6.03%–7.70%)	35.10% (25.30%–42.04%)	40.20% (34.92%–44.14%)	7.17% (6.18%–8.00%)	10.41% (9.09%–11.44%)
Libya	11.24% (9.93%–12.22%)	14.82% (12.97%–15.86%)	7.87% (6.69%–8.44%)	11.21% (9.64%–11.98%)	8.77% (7.20%–11.25%)	13.50% (11.09%–16.27%)	3.01% (2.48%–3.48%)	5.42% (4.58%–6.10%)	19.40% (14.94%–28.19%)	26.46% (20.66%–35.02%)	4.94% (4.11%–5.66%)	7.69% (6.50%–8.56%)
Morocco	13.26% (11.22%–14.48%)	18.52% (15.54%–20.31%)	7.80% (6.59%–8.42%)	11.33% (9.57%–12.19%)	8.89% (7.16%–10.86%)	15.29% (12.19%–17.58%)	3.02% (2.56%–3.48%)	5.82% (4.88%–6.79%)	16.16% (13.07%–22.29%)	25.54% (20.47%–30.38%)	4.86% (4.16%–5.49%)	8.39% (6.95%–9.65%)
Oman	8.96% (7.78%–9.72%)	7.88% (6.83%–8.59%)	6.44% (5.58%–6.96%)	6.20% (5.31%–6.68%)	7.44% (6.24%–8.40%)	7.83% (6.71%–8.57%)	3.32% (2.80%–3.64%)	3.96% (3.36%–4.35%)	14.81% (12.55%–17.71%)	17.74% (14.73%–20.96%)	5.06% (4.30%–5.52%)	5.75% (4.83%–6.39%)
Palestine	11.49% (10.24%–12.54%)	11.62% (10.23%–12.54%)	7.10% (6.17%–7.64%)	8.69% (7.56%–9.24%)	8.85% (7.35%–10.07%)	10.12% (8.88%–11.13%)	2.52% (2.18%–3.04%)	4.03% (3.35%–4.51%)	21.90% (18.09%–24.98%)	21.83% (18.30%–25.03%)	4.78% (4.15%–5.59%)	6.45% (5.39%–7.13%)
Qatar	5.56% (4.93%–6.02%)	6.57% (5.73%–7.09%)	5.85% (4.96%–6.28%)	6.63% (5.73%–7.13%)	5.14% (4.48%–5.63%)	5.96% (5.10%–6.57%)	3.70% (3.20%–4.18%)	4.19% (3.49%–4.65%)	10.80% (8.91%–12.39%)	9.81% (7.96%–12.26%)	5.48% (4.72%–6.13%)	5.73% (4.76%–6.34%)
Saudi Arabia	10.54% (9.26%–11.35%)	10.40% (9.10%–11.29%)	8.15% (6.93%–8.76%)	10.04% (8.45%–10.79%)	9.02% (7.69%–10.14%)	10.04% (8.74%–10.86%)	4.22% (3.51%–4.78%)	6.38% (5.41%–7.24%)	15.58% (12.79%–21.22%)	16.57% (13.29%–21.54%)	6.39% (5.30%–7.17%)	7.87% (6.64%–8.88%)
Sudan	10.80% (9.63%–11.63%)	10.85% (9.57%–11.76%)	8.21% (7.13%–8.83%)	9.26% (7.91%–9.94%)	6.66% (4.31%–9.73%)	7.95% (6.78%–9.38%)	3.85% (3.16%–4.79%)	5.43% (4.14%–6.29%)	13.80% (9.27%–21.41%)	16.50% (13.70%–21.89%)	6.96% (5.86%–8.22%)	8.87% (6.87%–10.19%)
Syria	10.53% (9.17%–11.53%)	16.57% (14.46%–17.88%)	7.46% (6.45%–7.98%)	12.33% (10.69%–13.17%)	8.32% (6.73%–9.72%)	15.52% (13.65%–16.86%)	2.36% (1.97%–2.73%)	6.44% (5.37%–7.20%)	19.12% (14.67%–23.72%)	28.44% (24.67%–31.42%)	4.02% (3.41%–4.60%)	9.70% (8.18%–10.61%)
Tunisia	12.72% (11.30%–13.75%)	18.82% (16.58%–19.99%)	8.70% (7.49%–9.30%)	12.99% (11.32%–13.77%)	9.57% (8.23%–11.65%)	17.94% (15.82%–19.65%)	3.49% (3.05%–3.97%)	7.47% (6.30%–8.34%)	18.75% (15.51%–24.84%)	33.01% (28.04%–38.42%)	5.78% (5.02%–6.46%)	10.49% (8.82%–11.57%)
Türkiye	13.16% (11.58%–14.32%)	22.55% (19.68%–24.52%)	8.93% (7.73%–9.58%)	12.53% (10.76%–13.37%)	10.78% (8.54%–12.96%)	23.67% (20.34%–26.14%)	3.47% (2.88%–3.96%)	6.65% (5.67%–7.28%)	28.63% (21.35%–34.14%)	43.90% (36.62%–48.89%)	5.66% (4.74%–6.47%)	9.93% (8.44%–10.73%)
United Arab Emirates	5.45% (4.78%–5.91%)	8.21% (7.12%–8.80%)	5.99% (5.14%–6.45%)	9.55% (8.08%–10.24%)	4.92% (4.17%–5.48%)	7.73% (6.74%–8.39%)	3.85% (3.18%–4.32%)	6.51% (5.64%–7.24%)	8.77% (6.72%–10.40%)	9.82% (8.19%–11.04%)	5.44% (4.59%–6.12%)	7.30% (6.27%–8.23%)
Yemen	10.06% (8.93%–10.97%)	11.09% (9.88%–11.95%)	6.79% (5.88%–7.34%)	8.38% (7.22%–8.95%)	6.22% (4.54%–9.11%)	8.54% (7.36%–10.07%)	2.53% (2.06%–3.13%)	3.88% (3.21%–4.50%)	12.52% (8.97%–19.71%)	17.60% (14.56%–23.04%)	4.30% (3.64%–5.23%)	5.99% (4.99%–6.75%)
South Asia	16.52% (14.51%–17.87%)	24.82% (21.84%–26.98%)	8.74% (7.55%–9.33%)	11.32% (9.81%–12.09%)	16.04% (13.87%–18.05%)	25.28% (22.55%–27.73%)	3.78% (3.27%–4.14%)	5.92% (5.01%–6.45%)	30.00% (26.76%–33.14%)	39.87% (35.33%–43.02%)	5.97% (5.16%–6.49%)	8.61% (7.36%–9.26%)
Bangladesh	10.83% (9.61%–11.76%)	15.99% (14.27%–17.32%)	7.06% (6.12%–7.53%)	10.56% (9.22%–11.27%)	8.66% (6.14%–14.46%)	16.88% (13.89%–21.10%)	2.41% (2.02%–2.86%)	4.81% (3.93%–5.55%)	20.34% (14.48%–34.60%)	34.41% (27.89%–43.11%)	4.47% (3.76%–5.17%)	7.88% (6.71%–8.83%)
Bhutan	12.95% (11.40%–14.22%)	22.94% (20.10%–25.04%)	7.86% (6.79%–8.46%)	10.62% (9.10%–11.41%)	13.42% (10.84%–15.98%)	23.93% (20.98%–26.91%)	2.99% (2.25%–4.17%)	5.06% (4.12%–5.74%)	28.07% (23.72%–32.86%)	40.36% (34.56%–45.66%)	4.78% (3.70%–6.39%)	8.11% (6.65%–9.06%)
India	16.90% (14.82%–18.30%)	25.83% (22.75%–28.06%)	8.81% (7.61%–9.40%)	11.63% (10.10%–12.42%)	16.61% (14.36%–18.76%)	26.53% (23.43%–29.02%)	3.93% (3.38%–4.28%)	6.46% (5.48%–7.00%)	30.48% (27.00%–33.86%)	40.78% (35.92%–44.15%)	6.09% (5.23%–6.61%)	9.14% (7.81%–9.79%)
Nepal	14.36% (12.69%–15.44%)	20.42% (18.03%–21.87%)	8.81% (7.69%–9.40%)	11.45% (9.88%–12.21%)	10.90% (7.77%–16.87%)	19.08% (15.12%–25.73%)	3.23% (2.72%–3.87%)	5.58% (4.67%–6.55%)	21.00% (14.31%–35.37%)	33.03% (25.17%–44.92%)	5.50% (4.60%–6.42%)	9.74% (8.10%–11.41%)
Pakistan	13.99% (12.42%–15.34%)	12.93% (11.45%–14.01%)	9.19% (7.95%–9.82%)	8.92% (7.63%–9.55%)	13.27% (10.57%–15.67%)	11.79% (9.89%–14.19%)	3.38% (2.93%–3.83%)	3.49% (2.88%–3.90%)	28.95% (25.00%–32.93%)	25.52% (22.47%–29.81%)	5.80% (5.01%–6.52%)	5.62% (4.72%–6.13%)
East Asia	16.75% (14.74%–18.07%)	27.03% (23.91%–29.00%)	9.50% (8.29%–10.13%)	13.99% (12.07%–14.87%)	11.30% (9.63%–12.80%)	25.44% (22.40%–27.53%)	3.68% (3.16%–4.04%)	8.58% (7.27%–9.32%)	18.59% (16.66%–20.45%)	39.92% (34.05%–44.39%)	5.18% (4.50%–5.63%)	11.07% (9.37%–11.99%)
China	16.70% (14.70%–18.03%)	27.01% (23.90%–28.99%)	9.45% (8.25%–10.08%)	14.00% (12.08%–14.88%)	11.23% (9.57%–12.73%)	25.48% (22.43%–27.58%)	3.61% (3.09%–3.97%)	8.60% (7.28%–9.34%)	18.51% (16.57%–20.38%)	40.03% (34.14%–44.53%)	5.06% (4.39%–5.51%)	11.08% (9.37%–12.02%)
North Korea	18.09% (15.95%–19.52%)	25.42% (22.50%–27.22%)	10.72% (9.16%–11.47%)	13.12% (11.35%–14.01%)	13.26% (10.74%–15.40%)	20.07% (16.82%–22.64%)	5.04% (4.28%–5.68%)	7.31% (6.32%–8.19%)	23.31% (18.86%–28.02%)	33.37% (27.76%–38.76%)	6.68% (5.69%–7.43%)	9.14% (8.09%–9.97%)
Taiwan (province of China)	18.57% (16.57%–19.87%)	29.77% (26.17%–31.91%)	10.89% (9.44%–11.53%)	15.05% (12.97%–16.13%)	14.32% (12.85%–15.45%)	27.45% (24.34%–29.39%)	5.59% (4.85%–5.96%)	10.35% (8.94%–11.24%)	19.87% (17.66%–21.34%)	39.39% (34.87%–42.74%)	8.56% (7.44%–9.07%)	14.46% (12.45%–15.65%)
Oceania	15.96% (14.06%–17.35%)	18.01% (15.84%–19.50%)	8.36% (7.07%–8.99%)	9.34% (7.93%–9.99%)	11.95% (10.05%–13.72%)	13.76% (12.14%–15.38%)	3.47% (3.00%–4.02%)	3.97% (3.36%–4.61%)	20.48% (17.24%–23.92%)	22.81% (19.74%–26.37%)	5.22% (4.50%–6.02%)	5.79% (4.91%–6.49%)
American Samoa	12.83% (11.19%–13.99%)	19.87% (17.36%–21.58%)	8.30% (6.97%–8.97%)	11.59% (9.72%–12.51%)	11.12% (9.84%–12.46%)	19.94% (17.04%–21.87%)	2.48% (2.02%–2.97%)	4.84% (3.96%–5.75%)	22.25% (19.37%–25.22%)	35.86% (30.45%–40.44%)	3.68% (2.90%–4.37%)	6.77% (5.50%–7.86%)
Cook Islands	16.41% (14.36%–17.83%)	25.00% (21.79%–26.86%)	9.93% (8.41%–10.70%)	14.18% (11.83%–15.37%)	15.76% (13.85%–17.50%)	25.36% (21.69%–27.47%)	3.75% (2.98%–4.27%)	7.37% (5.89%–8.51%)	29.74% (25.67%–33.57%)	40.32% (34.34%–44.46%)	5.81% (4.62%–6.64%)	10.29% (8.25%–11.63%)
Fiji	12.45% (10.84%–13.54%)	17.62% (15.50%–18.95%)	8.16% (6.81%–8.78%)	10.48% (8.72%–11.25%)	11.68% (10.33%–12.99%)	17.19% (14.81%–18.50%)	2.95% (2.40%–3.39%)	4.45% (3.58%–5.04%)	24.25% (20.30%–27.83%)	32.35% (27.34%–35.73%)	4.30% (3.57%–4.91%)	6.50% (5.25%–7.40%)
Guam	14.20% (12.37%–15.34%)	23.39% (20.40%–25.27%)	8.60% (7.15%–9.31%)	12.64% (10.73%–13.60%)	13.01% (11.38%–14.22%)	24.37% (20.90%–26.73%)	3.00% (2.49%–3.48%)	5.41% (4.50%–6.07%)	24.51% (20.55%–27.62%)	44.48% (37.71%–49.62%)	4.63% (3.83%–5.29%)	7.84% (6.63%–8.71%)
Kiribati	13.05% (11.53%–14.23%)	14.55% (12.91%–15.76%)	7.79% (6.66%–8.36%)	8.91% (7.47%–9.55%)	11.90% (10.46%–14.58%)	13.82% (11.99%–15.93%)	2.55% (2.11%–3.02%)	3.55% (2.85%–4.19%)	24.20% (19.92%–32.19%)	27.97% (22.80%–34.83%)	3.49% (2.86%–4.07%)	5.19% (4.24%–6.07%)
Marshall Islands	11.34% (9.98%–12.35%)	14.30% (12.49%–15.47%)	7.67% (6.51%–8.27%)	9.77% (8.26%–10.58%)	10.31% (8.82%–11.59%)	12.75% (10.94%–14.02%)	2.96% (2.40%–3.57%)	4.72% (3.77%–5.39%)	22.02% (18.63%–25.25%)	22.28% (18.90%–25.43%)	5.17% (4.17%–6.25%)	7.09% (5.85%–8.00%)
Federated States of Micronesia	13.04% (11.45%–14.23%)	16.46% (14.40%–17.77%)	8.21% (6.85%–8.90%)	10.60% (8.92%–11.41%)	11.87% (9.85%–13.45%)	15.47% (13.39%–16.78%)	3.36% (2.61%–3.86%)	5.23% (4.20%–6.14%)	24.16% (20.16%–27.78%)	28.29% (24.25%–32.07%)	5.83% (4.60%–6.67%)	7.95% (6.47%–9.10%)
Nauru	10.41% (9.09%–11.31%)	10.47% (9.16%–11.45%)	7.44% (6.26%–8.07%)	7.62% (6.47%–8.19%)	9.09% (7.84%–10.34%)	8.88% (7.57%–9.92%)	2.89% (2.37%–3.50%)	2.92% (2.35%–3.47%)	18.33% (15.26%–21.31%)	15.43% (12.72%–18.70%)	4.02% (3.24%–4.85%)	4.01% (3.23%–4.73%)
Niue	24.39% (21.23%–26.64%)	25.57% (22.31%–27.78%)	11.72% (9.87%–12.61%)	13.15% (11.09%–14.13%)	25.34% (21.82%–27.90%)	26.49% (22.71%–29.22%)	4.54% (3.42%–5.33%)	6.04% (4.77%–7.10%)	45.34% (38.98%–49.82%)	44.41% (37.84%–49.01%)	7.03% (5.44%–8.16%)	8.92% (7.31%–10.42%)
Northern Mariana Islands	9.57% (8.17%–10.42%)	22.82% (19.86%–24.49%)	7.90% (6.71%–8.53%)	14.12% (11.87%–15.18%)	9.61% (8.37%–10.45%)	22.99% (19.65%–25.05%)	3.51% (2.81%–4.00%)	8.05% (6.82%–8.87%)	19.17% (16.41%–21.61%)	35.41% (29.91%–39.69%)	4.67% (3.76%–5.26%)	10.15% (8.62%–11.16%)
Palau	16.34% (14.34%–17.71%)	20.11% (17.62%–21.63%)	8.89% (7.54%–9.57%)	12.15% (10.39%–13.07%)	16.25% (14.08%–18.32%)	20.45% (17.84%–22.54%)	3.31% (2.72%–4.00%)	5.59% (4.67%–6.55%)	31.99% (27.51%–36.40%)	36.10% (31.22%–40.22%)	4.84% (3.98%–5.87%)	7.28% (6.12%–8.30%)
Papua New Guinea	16.83% (14.83%–18.35%)	17.94% (15.80%–19.49%)	8.29% (7.02%–8.92%)	9.13% (7.76%–9.76%)	11.73% (9.47%–13.70%)	13.05% (11.28%–14.82%)	3.53% (3.00%–4.18%)	3.87% (3.24%–4.57%)	18.98% (14.84%–22.81%)	20.60% (17.29%–24.58%)	5.35% (4.49%–6.29%)	5.66% (4.77%–6.47%)
Samoa	15.99% (14.05%–17.45%)	19.44% (16.87%–21.39%)	9.12% (7.61%–9.85%)	10.82% (9.10%–11.62%)	14.92% (12.72%–16.85%)	19.02% (16.71%–21.14%)	2.91% (2.39%–3.39%)	4.15% (3.40%–4.82%)	31.25% (25.99%–36.18%)	36.54% (31.78%–40.87%)	4.65% (3.71%–5.35%)	6.19% (5.17%–6.99%)
Solomon Islands	16.64% (14.73%–18.08%)	17.60% (15.44%–18.99%)	8.98% (7.71%–9.68%)	9.49% (8.13%–10.14%)	12.56% (11.10%–14.14%)	14.49% (12.57%–16.14%)	4.36% (3.56%–5.19%)	4.83% (3.90%–5.66%)	17.72% (14.97%–20.48%)	20.88% (18.04%–24.15%)	5.96% (4.96%–7.00%)	6.27% (5.08%–7.24%)
Tokelau	22.30% (19.71%–24.09%)	23.30% (20.54%–25.14%)	11.48% (9.90%–12.29%)	12.73% (10.84%–13.61%)	22.49% (19.81%–24.70%)	23.48% (20.46%–25.77%)	4.08% (3.21%–4.80%)	5.29% (4.43%–6.31%)	40.19% (34.80%–43.92%)	40.71% (35.06%–44.60%)	6.52% (5.03%–7.52%)	7.76% (6.60%–9.17%)

Tonga	18.11% (15.94%–19.83%)	23.38% (20.30%–25.45%)	10.36% (8.81%–11.10%)	11.44% (9.57%–12.27%)	18.87% (16.42%–20.63%)	25.13% (21.71%–27.60%)	3.74% (3.16%–4.28%)	4.29% (3.44%–4.98%)	38.62% (32.93%–42.38%)	47.13% (40.44%–52.05%)	5.87% (4.98%–6.65%)	6.66% (5.39%–7.60%)
Tuvalu	17.45% (15.41%–18.72%)	20.07% (17.75%–21.61%)	9.62% (8.22%–10.35%)	10.95% (9.29%–11.77%)	15.82% (12.97%–19.09%)	19.37% (16.81%–21.47%)	3.40% (2.83%–3.91%)	4.18% (3.49%–4.70%)	29.34% (24.73%–35.51%)	34.89% (29.57%–38.98%)	5.14% (4.34%–5.84%)	6.06% (5.06%–6.78%)
Vanuatu	12.08% (10.65%–13.22%)	15.33% (13.41%–16.50%)	8.41% (7.16%–9.03%)	9.42% (8.07%–10.08%)	11.36% (10.09%–12.67%)	14.11% (12.16%–15.90%)	3.13% (2.63%–3.57%)	3.40% (2.68%–3.93%)	22.36% (19.09%–25.65%)	26.91% (23.72%–30.51%)	4.39% (3.78%–4.99%)	4.82% (3.87%–5.54%)
Southeast Asia	16.79% (14.79%–18.24%)	22.58% (20.02%–24.30%)	8.16% (7.05%–8.71%)	11.82% (10.16%–12.61%)	14.78% (11.34%–17.16%)	20.92% (17.84%–23.12%)	3.21% (2.80%–3.50%)	5.83% (4.93%–6.42%)	28.17% (21.03%–33.00%)	34.87% (27.58%–39.69%)	5.19% (4.46%–5.59%)	8.53% (7.24%–9.20%)
Cambodia	13.66% (12.12%–14.79%)	20.13% (17.87%–21.75%)	7.51% (6.52%–8.02%)	10.21% (8.76%–10.81%)	11.24% (8.46%–13.65%)	18.75% (15.88%–21.68%)	2.69% (2.19%–3.06%)	4.79% (3.93%–5.51%)	23.54% (17.90%–27.46%)	33.19% (26.96%–39.33%)	4.40% (3.64%–4.99%)	7.33% (6.02%–8.33%)
Indonesia	17.55% (15.41%–19.07%)	22.50% (19.91%–24.31%)	7.13% (6.19%–7.64%)	10.99% (9.49%–11.71%)	15.30% (11.66%–17.99%)	20.44% (17.02%–22.87%)	2.76% (2.38%–3.04%)	5.03% (4.19%–5.70%)	27.80% (20.46%–33.00%)	33.88% (26.68%–38.35%)	4.68% (4.00%–5.15%)	7.77% (6.56%–8.79%)
Laos	13.18% (11.71%–14.21%)	16.44% (14.78%–17.69%)	7.80% (6.74%–8.41%)	9.54% (8.22%–10.19%)	11.07% (8.45%–13.39%)	14.21% (12.49%–15.72%)	2.98% (2.50%–3.41%)	4.07% (3.34%–4.61%)	20.93% (16.51%–25.00%)	26.10% (22.05%–29.43%)	4.75% (3.92%–5.44%)	6.19% (5.10%–6.83%)
Malaysia	16.61% (14.61%–18.07%)	21.30% (18.84%–22.98%)	8.85% (7.55%–9.41%)	11.06% (9.40%–11.89%)	15.07% (13.29%–16.68%)	20.75% (18.41%–22.65%)	3.90% (3.26%–4.45%)	6.32% (5.40%–6.94%)	27.20% (23.60%–30.65%)	33.23% (29.18%–36.76%)	6.27% (5.21%–7.08%)	9.42% (8.06%–10.28%)
Maldives	12.30% (10.72%–13.31%)	14.13% (12.38%–15.42%)	7.93% (6.68%–8.49%)	8.02% (6.82%–8.66%)	10.18% (8.25%–11.99%)	14.72% (12.04%–16.46%)	3.02% (2.53%–3.61%)	4.24% (3.40%–4.88%)	18.99% (15.40%–22.82%)	31.57% (21.21%–36.54%)	4.51% (3.80%–5.30%)	6.63% (5.31%–7.54%)
Mauritius	13.19% (11.73%–14.23%)	21.24% (18.82%–22.85%)	9.31% (7.98%–9.93%)	12.98% (11.18%–13.88%)	12.37% (11.15%–13.23%)	19.39% (17.15%–20.64%)	4.96% (4.22%–5.39%)	6.90% (5.95%–7.52%)	19.91% (17.96%–21.16%)	27.97% (24.50%–30.22%)	7.48% (6.32%–8.07%)	9.70% (8.35%–10.58%)
Myanmar	13.46% (12.06%–14.49%)	19.30% (17.25%–20.66%)	8.36% (7.29%–8.93%)	11.24% (9.67%–11.98%)	11.73% (8.68%–13.58%)	17.36% (14.65%–19.21%)	3.07% (2.53%–3.53%)	5.06% (4.18%–5.72%)	23.13% (16.89%–27.11%)	31.22% (24.50%–35.51%)	4.77% (4.05%–5.36%)	7.51% (6.31%–8.35%)
Philippines	13.37% (11.92%–14.52%)	17.59% (15.61%–19.03%)	8.57% (7.44%–9.11%)	10.82% (9.34%–11.52%)	10.37% (8.11%–12.73%)	15.95% (13.75%–17.31%)	2.66% (2.25%–2.97%)	4.87% (4.00%–5.32%)	18.78% (14.34%–23.21%)	28.72% (24.36%–31.48%)	3.89% (3.20%–4.27%)	7.10% (5.89%–7.71%)
Seychelles	18.38% (16.21%–19.86%)	18.62% (16.36%–20.03%)	10.36% (8.84%–11.10%)	12.01% (10.09%–12.90%)	15.56% (13.84%–16.98%)	17.14% (15.07%–18.35%)	4.82% (4.14%–5.39%)	7.00% (5.68%–7.79%)	25.95% (23.15%–28.54%)	26.10% (22.89%–28.43%)	7.16% (6.27%–7.98%)	9.57% (7.78%–10.61%)
Sri Lanka	15.66% (13.92%–16.90%)	25.00% (22.42%–26.72%)	9.78% (8.49%–10.40%)	13.52% (11.57%–14.41%)	13.13% (11.51%–14.98%)	22.83% (20.38%–24.91%)	4.30% (3.79%–4.82%)	7.16% (5.99%–7.97%)	24.23% (21.54%–26.50%)	33.67% (29.83%–37.39%)	6.79% (5.93%–7.52%)	10.03% (8.42%–10.91%)
Thailand	15.49% (13.56%–16.87%)	24.32% (21.45%–26.12%)	8.95% (7.66%–9.66%)	13.37% (11.45%–14.35%)	14.38% (11.79%–16.02%)	22.32% (19.69%–25.27%)	3.72% (3.13%–4.17%)	6.25% (5.34%–7.08%)	27.39% (22.02%–31.76%)	33.11% (28.78%–39.49%)	5.48% (4.66%–6.10%)	8.58% (7.33%–9.66%)
Timor-Leste	10.86% (9.67%–11.73%)	18.87% (16.83%–20.45%)	7.07% (6.08%–7.53%)	10.28% (8.88%–10.98%)	7.97% (5.88%–10.10%)	16.01% (13.70%–18.36%)	2.18% (1.79%–2.66%)	3.95% (3.19%–4.50%)	16.26% (12.75%–19.77%)	28.47% (24.29%–32.58%)	3.43% (2.89%–4.19%)	6.50% (5.29%–7.37%)
Viet Nam	23.20% (20.24%–25.33%)	26.76% (23.67%–28.98%)	9.96% (8.63%–10.67%)	12.59% (10.88%–13.42%)	20.45% (14.58%–25.30%)	26.05% (20.58%–30.39%)	4.09% (3.53%–4.60%)	7.97% (6.69%–8.76%)	36.24% (25.24%–44.00%)	40.75% (29.02%–48.47%)	6.71% (5.81%–7.49%)	10.84% (9.19%–11.89%)
Central sub-Saharan Africa	13.15% (11.63%–14.43%)	14.39% (12.69%–15.68%)	6.79% (5.92%–7.29%)	8.00% (6.95%–8.51%)	12.00% (9.31%–14.05%)	14.43% (12.31%–16.02%)	1.80% (1.47%–2.14%)	2.83% (2.40%–3.23%)	23.91% (19.14%–27.24%)	29.44% (25.02%–32.49%)	3.39% (2.85%–3.96%)	4.75% (4.05%–5.30%)
Angola	11.31% (10.09%–12.56%)	13.32% (11.78%–14.47%)	6.49% (5.71%–6.95%)	7.68% (6.69%–8.19%)	10.58% (7.10%–12.83%)	13.20% (11.01%–15.07%)	1.56% (1.26%–1.85%)	2.52% (2.07%–2.97%)	22.15% (15.52%–26.29%)	27.54% (22.45%–31.99%)	2.93% (2.42%–3.41%)	4.24% (3.54%–4.84%)
Central African Republic	12.71% (11.36%–13.88%)	12.28% (10.95%–13.37%)	7.16% (6.23%–7.77%)	7.24% (6.30%–7.80%)	12.19% (9.74%–14.11%)	12.28% (10.36%–14.03%)	2.40% (1.97%–2.79%)	2.77% (2.31%–3.28%)	24.03% (19.45%–27.57%)	24.51% (19.90%–28.39%)	4.18% (3.53%–4.78%)	4.48% (3.81%–5.10%)
Congo (Brazzaville)	14.73% (13.04%–16.26%)	15.84% (14.13%–17.14%)	7.87% (6.85%–8.49%)	9.47% (8.23%–10.09%)	15.13% (12.50%–17.43%)	16.69% (14.56%–18.79%)	2.81% (2.27%–3.33%)	3.98% (3.33%–4.52%)	28.32% (23.65%–31.51%)	31.44% (27.36%–35.69%)	4.80% (4.00%–5.59%)	6.10% (5.20%–6.79%)
DR Congo	13.37% (11.78%–14.66%)	14.69% (12.88%–16.05%)	6.72% (5.85%–7.23%)	8.05% (6.99%–8.60%)	12.00% (9.55%–14.25%)	14.80% (12.59%–16.60%)	1.74% (1.34%–2.17%)	2.86% (2.39%–3.36%)	23.63% (18.92%–27.15%)	30.11% (25.70%–33.62%)	3.32% (2.67%–4.04%)	4.84% (4.07%–5.53%)
Equatorial Guinea	14.47% (13.04%–15.72%)	13.10% (11.40%–14.54%)	7.79% (6.77%–8.36%)	7.24% (6.21%–7.84%)	14.87% (11.81%–17.45%)	13.91% (11.38%–16.34%)	2.62% (2.12%–3.03%)	2.29% (1.80%–2.74%)	28.64% (23.34%–32.90%)	30.56% (24.87%–36.11%)	4.66% (3.92%–5.30%)	3.94% (3.16%–4.68%)
Gabon	18.35% (16.12%–19.96%)	18.23% (16.11%–19.67%)	8.98% (7.87%–9.64%)	9.95% (8.55%–10.61%)	19.21% (16.75%–20.95%)	19.26% (16.85%–21.77%)	3.46% (2.87%–3.98%)	4.35% (3.49%–5.04%)	35.26% (30.98%–38.70%)	36.28% (31.19%–41.35%)	6.00% (5.03%–6.77%)	6.85% (5.52%–7.83%)
Eastern sub-Saharan Africa	13.22% (11.68%–14.47%)	14.71% (12.97%–16.09%)	7.59% (6.64%–8.13%)	8.86% (7.77%–9.42%)	13.44% (11.74%–15.08%)	15.52% (13.66%–17.26%)	3.67% (3.11%–4.09%)	4.48% (3.86%–4.93%)	27.86% (24.85%–30.67%)	32.31% (28.44%–35.51%)	6.94% (6.02%–7.53%)	7.99% (6.93%–8.64%)
Burundi	14.19% (12.44%–15.54%)	12.75% (11.29%–14.03%)	7.68% (6.82%–8.25%)	8.47% (7.45%–9.00%)	14.42% (12.62%–15.97%)	13.91% (12.54%–15.49%)	3.55% (2.97%–4.07%)	4.34% (3.71%–5.14%)	29.80% (26.13%–33.05%)	28.82% (25.91%–31.81%)	6.82% (5.87%–7.62%)	7.53% (6.46%–8.54%)
Comoros	15.81% (14.04%–17.31%)	19.98% (17.61%–21.60%)	8.85% (7.77%–9.47%)	11.57% (10.11%–12.23%)	16.88% (14.59%–20.11%)	23.26% (20.12%–25.87%)	3.96% (3.03%–6.22%)	6.24% (5.12%–7.23%)	33.08% (29.66%–37.19%)	40.72% (35.23%–44.62%)	7.86% (6.49%–10.55%)	10.90% (9.28%–12.00%)
Djibouti	9.87% (8.82%–10.88%)	14.19% (12.53%–15.33%)	6.57% (5.80%–7.01%)	9.49% (8.37%–10.06%)	10.20% (8.96%–11.55%)	16.63% (14.61%–18.94%)	3.29% (2.83%–3.82%)	6.37% (5.30%–7.45%)	22.13% (19.43%–24.96%)	30.67% (26.92%–34.04%)	5.89% (5.19%–6.69%)	9.71% (8.27%–10.88%)
Eritrea	10.62% (9.44%–11.64%)	13.27% (11.81%–14.33%)	7.30% (6.36%–7.82%)	8.78% (7.74%–9.38%)	11.00% (9.35%–12.60%)	13.92% (12.07%–15.50%)	3.71% (2.94%–4.38%)	4.64% (3.98%–5.30%)	22.43% (19.23%–25.66%)	27.92% (24.76%–31.56%)	6.30% (5.17%–7.18%)	7.52% (6.49%–8.50%)
Ethiopia	12.74% (11.20%–14.00%)	15.74% (13.71%–17.31%)	7.30% (6.35%–7.90%)	9.10% (7.98%–9.71%)	13.16% (11.31%–14.87%)	16.78% (14.64%–19.12%)	4.05% (3.31%–4.75%)	4.42% (3.73%–4.88%)	26.14% (22.66%–29.68%)	34.91% (30.79%–38.54%)	7.23% (6.11%–7.86%)	8.30% (7.18%–8.99%)
Kenya	15.14% (13.32%–16.86%)	16.80% (14.79%–18.37%)	8.66% (7.66%–9.21%)	10.09% (8.82%–10.70%)	16.18% (14.38%–17.85%)	18.61% (16.14%–21.12%)	4.13% (3.54%–4.66%)	6.04% (5.10%–6.71%)	32.85% (29.41%–36.10%)	34.56% (30.12%–38.59%)	7.97% (7.01%–8.74%)	9.82% (8.38%–10.71%)
Madagascar	12.50% (11.11%–13.66%)	12.69% (11.44%–13.73%)	7.39% (6.53%–7.97%)	9.13% (8.06%–9.74%)	12.86% (9.98%–14.77%)	13.50% (11.55%–14.88%)	2.84% (2.38%–3.33%)	4.31% (3.63%–5.03%)	28.76% (24.28%–32.69%)	28.16% (23.91%–30.88%)	5.65% (4.91%–6.45%)	7.32% (6.36%–8.40%)
Malawi	12.76% (11.32%–13.97%)	14.32% (12.65%–15.71%)	7.09% (6.20%–7.58%)	8.35% (7.36%–8.90%)	11.90% (9.60%–14.71%)	15.36% (13.62%–17.27%)	2.91% (2.38%–3.56%)	4.22% (3.52%–4.72%)	25.95% (22.85%–30.02%)	32.17% (28.04%–35.42%)	5.87% (4.91%–6.88%)	7.46% (6.33%–8.20%)
Mozambique	13.85% (12.40%–15.11%)	13.37% (11.80%–14.67%)	8.56% (7.49%–9.14%)	8.44% (7.43%–9.03%)	14.01% (12.04%–16.29%)	14.50% (12.40%–17.02%)	3.97% (3.25%–4.75%)	4.78% (3.91%–5.61%)	28.48% (25.58%–31.94%)	30.07% (26.17%–33.99%)	7.56% (6.45%–8.63%)	8.14% (6.85%–9.28%)
Rwanda	13.12% (11.54%–14.41%)	15.77% (13.92%–17.21%)	7.07% (6.24%–7.63%)	9.15% (7.98%–9.79%)	13.79% (11.63%–15.32%)	17.19% (15.00%–19.97%)	2.94% (2.45%–3.51%)	4.65% (3.95%–5.38%)	28.39% (25.02%–31.36%)	33.95% (29.38%–38.65%)	5.87% (4.97%–6.75%)	8.43% (7.36%–9.46%)
Somalia	11.38% (10.28%–12.45%)	11.22% (10.06%–12.31%)	7.91% (6.92%–8.43%)	7.81% (6.84%–8.31%)	12.59% (10.67%–14.70%)	12.41% (10.73%–14.23%)	4.23% (3.44%–5.18%)	4.18% (3.44%–4.98%)	25.61% (21.86%–29.98%)	25.88% (22.20%–29.81%)	7.20% (5.99%–8.28%)	7.05% (5.76%–8.16%)
South Sudan	12.30% (10.83%–13.50%)	13.77% (12.20%–15.14%)	7.45% (6.56%–7.98%)	9.06% (7.89%–9.62%)	14.19% (12.34%–16.26%)	13.22% (11.42%–14.94%)	3.64% (2.94%–4.44%)	3.71% (3.10%–4.63%)	29.96% (26.61%–33.07%)	28.73% (25.28%–32.06%)	7.21% (6.03%–8.35%)	7.07% (6.18%–8.33%)
Uganda	13.07% (11.51%–14.43%)	13.40% (11.70%–14.75%)	7.55% (6.62%–8.06%)	8.06% (7.02%–8.63%)	14.32% (12.32%–16.18%)	13.40% (11.73%–15.27%)	3.77% (3.08%–4.60%)	3.64% (2.99%–4.26%)	30.02% (26.76%–33.13%)	30.44% (26.85%–34.47%)	7.68% (6.58%–8.81%)	7.20% (6.06%–8.07%)
Tanzania	13.78% (12.15%–15.05%)	15.39% (13.46%–16.92%)	7.88% (6.91%–8.40%)	9.09% (7.99%–9.74%)	12.70% (10.69%–15.50%)	15.34% (12.35%–17.99%)	3.49% (2.87%–4.00%)	4.64% (3.92%–5.25%)	27.08% (23.30%–31.27%)	32.86% (27.73%–37.54%)	6.79% (5.88%–7.60%)	8.29% (7.08%–9.24%)
Zambia	12.63% (11.04%–13.87%)	13.45% (12.01%–14.76%)	6.95% (6.05%–7.49%)	8.33% (7.34%–8.90%)	12.64% (10.48%–14.51%)	14.13% (12.19%–16.12%)	3.09% (2.52%–3.73%)	4.30% (3.49%–4.98%)	26.98% (24.01%–30.12%)	29.51% (25.47%–32.72%)	6.26% (5.26%–7.11%)	7.36% (6.11%–8.29%)
Southern sub-Saharan Africa	13.82% (12.26%–14.95%)	15.31% (13.55%–16.45%)	9.18% (7.94%–9.81%)	10.40% (8.95%–11.09%)	12.33% (10.93%–13.46%)	13.90% (12.43%–14.98%)	3.19% (2.76%–3.49%)	4.67% (4.02%–5.09%)	24.85% (21.29%–27.69%)	26.01% (23.04%–28.70%)	4.54% (3.93%–4.93%)	6.63% (5.77%–7.13%)

Botswana	12.57% (11.17%–13.64%)	13.47% (11.88%–14.52%)	8.41% (7.30%–9.01%)	9.05% (7.74%–9.73%)	10.61% (9.19%–11.95%)	12.17% (10.28%–13.83%)	3.01% (2.49%–3.54%)	4.07% (3.23%–4.77%)	19.86% (16.70%–22.92%)	22.18% (17.89%–26.54%)	4.99% (4.20%–5.74%)	6.22% (4.96%–7.19%)
Eswatini	11.68% (10.31%–12.77%)	12.21% (10.72%–13.23%)	8.10% (6.93%–8.70%)	8.41% (7.22%–9.09%)	10.29% (8.92%–11.41%)	10.64% (8.94%–12.05%)	3.50% (2.95%–4.01%)	4.10% (3.42%–4.84%)	20.62% (17.48%–23.40%)	20.17% (17.33%–23.18%)	5.55% (4.69%–6.27%)	5.87% (4.87%–6.71%)
Lesotho	14.67% (13.10%–15.85%)	13.71% (12.25%–14.77%)	9.58% (8.38%–10.25%)	8.96% (7.80%–9.62%)	13.04% (11.51%–14.28%)	12.14% (10.87%–13.42%)	4.47% (3.76%–5.00%)	4.69% (3.91%–5.27%)	24.07% (20.43%–27.05%)	22.21% (19.71%–25.33%)	6.94% (5.94%–7.73%)	6.64% (5.65%–7.34%)
Namibia	14.58% (12.98%–15.93%)	15.31% (13.70%–16.57%)	9.60% (8.38%–10.24%)	10.03% (8.58%–10.75%)	13.34% (11.63%–14.86%)	13.96% (11.64%–15.85%)	4.25% (3.67%–4.85%)	4.89% (4.07%–5.48%)	24.77% (21.44%–28.11%)	26.84% (21.87%–30.56%)	6.77% (5.92%–7.60%)	7.26% (6.00%–7.99%)
South Africa	13.84% (12.26%–14.95%)	15.75% (13.94%–16.97%)	9.20% (7.93%–9.84%)	10.64% (9.15%–11.34%)	11.97% (10.26%–13.30%)	14.52% (13.15%–15.73%)	3.03% (2.59%–3.34%)	4.65% (3.97%–5.09%)	24.07% (19.18%–27.74%)	26.73% (23.58%–29.83%)	4.19% (3.59%–4.59%)	6.47% (5.60%–7.01%)
Zimbabwe	13.78% (12.12%–15.11%)	14.31% (12.74%–15.45%)	8.99% (7.96%–9.59%)	9.28% (8.06%–9.86%)	14.05% (11.85%–15.63%)	12.88% (10.70%–15.14%)	4.71% (3.91%–5.49%)	4.99% (4.32%–5.63%)	28.44% (23.56%–32.25%)	25.65% (20.49%–30.68%)	7.45% (6.35%–8.53%)	7.90% (6.83%–8.70%)
Western sub-Saharan Africa	15.51% (13.64%–16.99%)	15.42% (13.53%–16.89%)	8.37% (7.33%–8.92%)	8.86% (7.70%–9.44%)	14.58% (12.59%–16.14%)	15.07% (13.29%–16.87%)	3.23% (2.80%–3.72%)	4.11% (3.47%–4.66%)	29.68% (26.31%–32.00%)	31.39% (27.79%–34.34%)	6.41% (5.66%–7.22%)	7.43% (6.33%–8.22%)
Benin	15.19% (13.35%–16.37%)	14.21% (12.38%–15.47%)	7.92% (7.06%–8.43%)	8.54% (7.41%–9.17%)	13.44% (11.30%–15.83%)	13.47% (11.64%–15.67%)	2.67% (2.16%–3.41%)	3.45% (2.81%–4.15%)	30.02% (26.77%–33.49%)	29.17% (25.48%–32.90%)	5.76% (4.79%–7.00%)	6.43% (5.46%–7.45%)
Burkina Faso	16.22% (14.12%–17.69%)	14.71% (12.94%–16.00%)	9.07% (7.95%–9.62%)	8.64% (7.61%–9.24%)	14.32% (12.29%–17.31%)	14.36% (12.06%–16.99%)	3.40% (2.81%–4.07%)	3.79% (3.07%–4.44%)	29.24% (26.50%–32.49%)	30.76% (26.69%–34.92%)	6.78% (5.81%–7.90%)	6.91% (5.68%–7.90%)
Cabo Verde	22.04% (19.33%–23.93%)	20.96% (18.47%–22.87%)	10.59% (9.50%–11.22%)	11.93% (10.39%–12.67%)	21.82% (19.58%–23.51%)	22.75% (19.61%–26.32%)	3.98% (3.36%–4.69%)	7.23% (5.97%–8.24%)	40.01% (35.49%–43.00%)	41.50% (35.35%–47.19%)	7.39% (6.25%–8.32%)	10.91% (9.23%–12.19%)
Cameroon	15.65% (13.85%–17.05%)	14.30% (12.50%–15.66%)	8.92% (7.67%–9.55%)	8.63% (7.32%–9.24%)	15.20% (13.13%–17.08%)	14.68% (12.93%–16.50%)	3.64% (3.07%–4.24%)	3.87% (3.22%–4.62%)	30.19% (27.15%–33.29%)	30.08% (26.34%–33.37%)	6.93% (5.99%–7.78%)	6.89% (5.82%–7.89%)
Chad	16.30% (14.48%–17.81%)	12.67% (11.26%–13.82%)	8.64% (7.61%–9.26%)	7.68% (6.70%–8.22%)	15.39% (12.33%–18.26%)	12.07% (10.38%–13.79%)	3.24% (2.68%–4.02%)	3.01% (2.52%–3.49%)	31.36% (27.69%–35.57%)	26.87% (23.68%–29.95%)	6.70% (5.71%–7.93%)	5.85% (5.01%–6.62%)
Côte d'Ivoire	10.89% (9.68%–11.85%)	13.14% (11.63%–14.45%)	7.29% (6.42%–7.87%)	8.79% (7.59%–9.42%)	10.37% (9.14%–11.55%)	13.43% (11.95%–15.14%)	3.27% (2.73%–3.98%)	4.41% (3.68%–5.33%)	22.05% (20.03%–24.28%)	27.51% (24.26%–30.28%)	5.97% (5.08%–6.98%)	7.44% (6.37%–8.65%)
The Gambia	13.27% (11.52%–14.55%)	17.20% (15.24%–18.89%)	8.23% (7.25%–8.78%)	10.13% (8.90%–10.78%)	12.23% (10.78%–13.69%)	17.45% (14.92%–20.64%)	3.26% (2.67%–3.90%)	4.77% (3.94%–5.48%)	26.78% (24.11%–29.41%)	34.81% (30.65%–38.89%)	6.38% (5.36%–7.23%)	8.55% (7.20%–9.60%)
Ghana	15.25% (13.38%–16.53%)	17.01% (15.03%–18.56%)	8.40% (7.43%–8.98%)	9.32% (8.05%–9.91%)	14.83% (12.76%–16.70%)	18.41% (16.21%–20.56%)	2.34% (1.91%–2.79%)	3.87% (3.29%–4.52%)	29.97% (26.23%–33.15%)	35.05% (30.81%–38.42%)	4.25% (3.57%–4.93%)	6.34% (5.40%–7.24%)
Guinea	16.58% (14.60%–17.93%)	14.84% (12.97%–16.28%)	8.83% (7.75%–9.41%)	8.68% (7.59%–9.23%)	14.75% (11.99%–17.20%)	15.13% (13.29%–16.98%)	2.74% (2.16%–3.55%)	3.68% (3.04%–4.27%)	30.73% (26.99%–33.84%)	32.29% (28.40%–35.71%)	5.90% (4.82%–7.31%)	7.00% (5.98%–7.88%)
Guinea-Bissau	13.07% (11.55%–14.21%)	13.41% (11.87%–14.55%)	7.26% (6.30%–7.78%)	7.86% (6.90%–8.41%)	12.24% (10.09%–14.24%)	13.25% (10.96%–14.99%)	1.81% (1.43%–2.32%)	2.65% (2.23%–3.12%)	25.24% (21.59%–28.74%)	27.19% (23.54%–29.95%)	3.32% (2.72%–4.20%)	4.31% (3.65%–4.97%)
Liberia	16.95% (15.00%–18.41%)	14.73% (13.04%–16.09%)	8.76% (7.66%–9.38%)	9.11% (7.84%–9.71%)	14.95% (11.78%–17.82%)	15.48% (13.58%–17.34%)	2.58% (2.07%–3.33%)	4.36% (3.49%–5.27%)	29.93% (25.89%–33.09%)	31.40% (27.71%–35.25%)	5.69% (4.76%–7.01%)	7.36% (6.20%–8.50%)
Mali	16.19% (14.12%–17.56%)	14.79% (12.89%–16.31%)	8.38% (7.35%–8.95%)	8.25% (7.24%–8.81%)	14.69% (11.87%–17.40%)	14.18% (12.25%–16.00%)	3.05% (2.41%–3.78%)	3.22% (2.71%–3.80%)	28.87% (25.54%–32.42%)	30.33% (26.99%–33.68%)	6.25% (5.18%–7.44%)	6.48% (5.58%–7.39%)
Mauritania	17.48% (15.48%–19.03%)	18.18% (15.96%–19.85%)	9.01% (7.76%–9.63%)	10.29% (8.86%–10.97%)	17.48% (15.32%–19.51%)	20.65% (17.96%–23.57%)	2.75% (2.20%–3.36%)	3.99% (3.37%–4.72%)	33.51% (29.53%–36.65%)	38.43% (33.50%–42.85%)	5.13% (4.13%–6.31%)	6.96% (5.91%–8.07%)
Niger	12.33% (10.82%–13.55%)	13.66% (11.95%–15.15%)	7.09% (6.25%–7.58%)	7.84% (6.81%–8.33%)	9.90% (7.37%–12.06%)	12.12% (10.52%–13.62%)	1.90% (1.40%–2.39%)	2.95% (2.35%–3.52%)	21.45% (17.61%–25.09%)	25.64% (22.54%–28.95%)	3.95% (3.03%–4.87%)	5.75% (4.74%–6.60%)
Nigeria	16.12% (14.17%–17.77%)	16.03% (14.00%–17.60%)	8.46% (7.43%–9.06%)	9.03% (7.90%–9.62%)	15.77% (13.67%–17.51%)	15.34% (13.30%–17.85%)	3.90% (3.36%–4.49%)	4.91% (3.98%–5.76%)	31.28% (27.42%–33.91%)	32.38% (28.67%–35.99%)	7.62% (6.77%–8.43%)	8.81% (7.30%–9.89%)
São Tomé and Príncipe	14.22% (12.46%–15.40%)	14.73% (13.09%–15.97%)	8.64% (7.54%–9.23%)	10.28% (8.98%–10.90%)	12.86% (10.38%–15.38%)	15.10% (12.35%–18.23%)	3.32% (2.60%–4.35%)	5.58% (4.61%–6.56%)	26.87% (22.68%–31.24%)	29.59% (23.86%–35.76%)	6.36% (5.19%–7.84%)	8.63% (7.16%–10.04%)
Senegal	15.14% (13.34%–16.43%)	17.80% (15.77%–19.38%)	8.41% (7.36%–8.95%)	10.23% (8.93%–10.88%)	13.15% (11.56%–14.59%)	18.01% (15.31%–20.38%)	2.87% (2.28%–3.35%)	4.90% (4.20%–5.59%)	28.02% (25.02%–31.24%)	34.21% (29.26%–38.20%)	5.92% (4.94%–6.70%)	8.63% (7.56%–9.60%)
Sierra Leone	16.04% (14.21%–17.39%)	14.30% (12.64%–15.57%)	8.35% (7.30%–8.93%)	8.32% (7.23%–8.95%)	14.94% (11.92%–17.36%)	15.22% (13.41%–17.31%)	2.80% (2.17%–3.40%)	3.85% (3.03%–4.91%)	31.09% (27.01%–34.26%)	31.43% (28.00%–35.36%)	6.03% (4.99%–6.98%)	7.06% (5.71%–8.55%)
Togo	12.79% (11.20%–13.84%)	15.68% (13.95%–17.01%)	7.06% (6.29%–7.58%)	9.01% (7.84%–9.57%)	11.90% (10.61%–13.29%)	16.18% (14.34%–17.90%)	1.69% (1.33%–2.19%)	3.46% (2.88%–4.00%)	26.26% (23.61%–29.04%)	30.51% (27.17%–33.19%)	3.11% (2.51%–3.98%)	5.47% (4.60%–6.39%)

Supplementary Methods

Table of Contents

1. Methodological summary for Estimating Burden of Disease Attributable to Low BMD
    - Flowchart
    - Definition
    - Exposure
    - Relative Risk
    - Attributable Burden
    - References
2. Methodological summary for Estimation of Injuries
    - Flowchart
    - Case Definition
    - Input Data
    - Modeling Strategy
    - References

1. Methodological summary for Estimating Burden of Disease Attributable to Low BMD

Flowchart

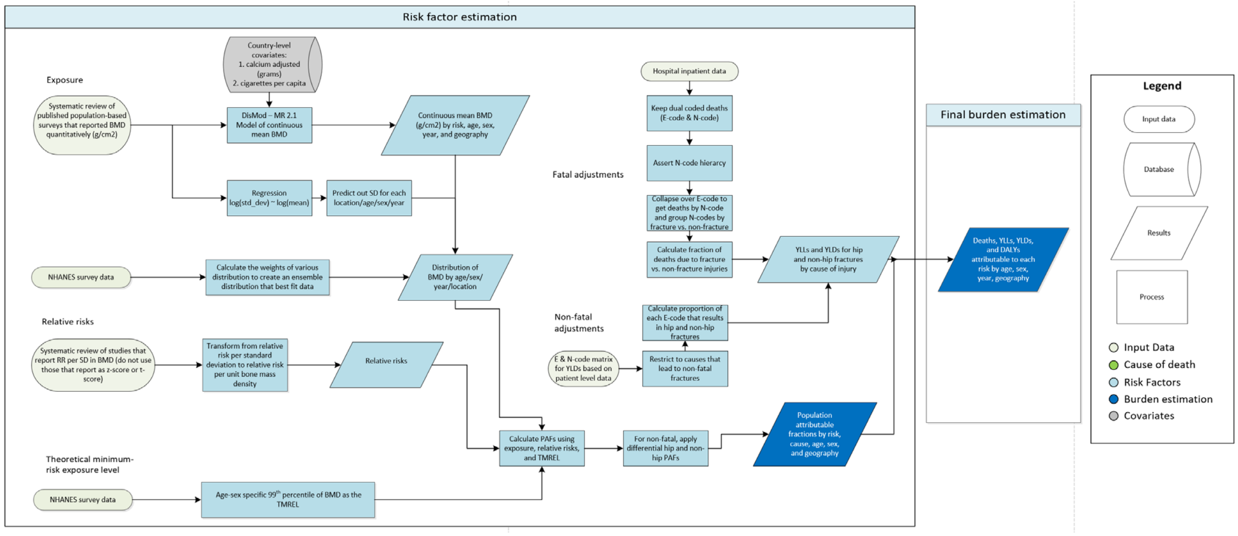

Definition: Low BMD

Bone mineral density (BMD) is a continuous variable measured by dual-X-ray-absorptiometry (DXA) at the femoral neck (FN) and is presented in g/cm^2 after standardizing for the brand of densitometer (sBMD).[1] Low BMD is defined in terms of the difference between mean BMD of a population and the 99th percentile of a reference population at the same age and sex (theoretical minimum-risk exposure level, TMREL).

Exposure

Input data

A systematic review was conducted in GBD 2010 and updated for GBD 2013 and 2015 using the same search string. It was not scheduled for systematic review in GBD 2016, 2017, 2019, or 2021. Details of the search strategy, inclusion and exclusion criteria, risk of bias assessment and data extraction approach utilized in modelling exposure have been described previously.[2,3] In brief, with each successive round of the GBD, new sources suggested by collaborators within the GBD network or identified in the Global Health Data Exchange database by GBD librarians (keywords: bone mineral density or osteoporosis) have been included (GHDX, http://ghdx.healthdata.org/). Inclusion criteria that informed the search are:

- Representative, population-based surveys
- Reporting of quantitative BMD
    - measured by DXA
    - performed at the FN region
    - measured in g/cm^2

Reasons for exclusion included: subsample not representative of the population (e.g., athletes); non-population-based studies (e.g., clinical-based); no prevalence/incidence data; only subtypes of osteoporosis assessed (e.g., steroid-induced osteoporosis); sample number <150; reviews. Mean BMD was occasionally reported in stratified groups, e.g., by fracture status rather than for the total sample. In these cases, the stratified means were aggregated to obtain a total mean BMD at the population level for an age or sex category.

Table 1. Exposure input data

Input data	Exposure
Source count (total)	169
Number of countries with data	48

See **Supplementary Results Appendix, Tables 1a and 1b** for a list of specific countries and regions included in the exposure input data.

Modelling strategy

We modelled mean BMD in DisMod-MR 2.1 as a single "continuous" parameter model by age and sex, and all GBD locations for years 1990–2020. The model had age mesh points at 0 10 20 25 30 40 50 60 70 80 90 & 100, a time window of ten years for fitting data. We made no substantive changes to the modelling strategy from GBD 2017.

The country covariates of total physical activity (MET-min/week), tobacco consumption (cigarettes per capita), mean BMI, and unadjusted calcium intake (g) were included in modelling. Data regarding additional risk factors (e.g., vitamin D supplement intake, sun exposure, etc.) were not available for inclusion in the model.

Table 2. Covariates included in the BMD DisMod-MR meta-regression model

Covariate	Type	Parameter	Exponentiated beta (95% uncertainty interval)
Total-physical activity (MET-min/week), age-standardised	Country-level	Continuous	1.00 (1.00 to 1.00)
Tobacco consumption (cigarettes per capita)	Country-level	Continuous	0.98 (0.96 to 0.99)
Mean BMI	Country-level	Continuous	1.01 (1.00 to 1.01)
Calcium intake (g), unadjusted	Country-level	Continuous	1.00 (1.00 to 1.01)

Theoretical minimum-risk exposure level

The theoretical minimum of risk exposure level, or TMREL, was chosen as the age-sex specific 99th percentile of BMD from five cycles of NHANES study as the reference population, given it is the most broadly accepted standard international reference. Below is a descriptive table of the five NHANES cycles used, inclusive of men and women and all races.

Table 3. TMREL. Summary of NHANES reference population

NHANES cycle	Age range (years)	Number of people tested	BMD range (g/cm^2)
1988	20–90	14,646	0.23–1.84
2005	20–85	3,494	0.40–1.50
2007	20–80	4,726	0.34–1.46

2009	20–80	5,052	0.33–1.63
2013	40–80	3,127	0.39–1.36

Figure 1: Plot of 99th percentile of BMD at femoral neck in each cycle of NHANES

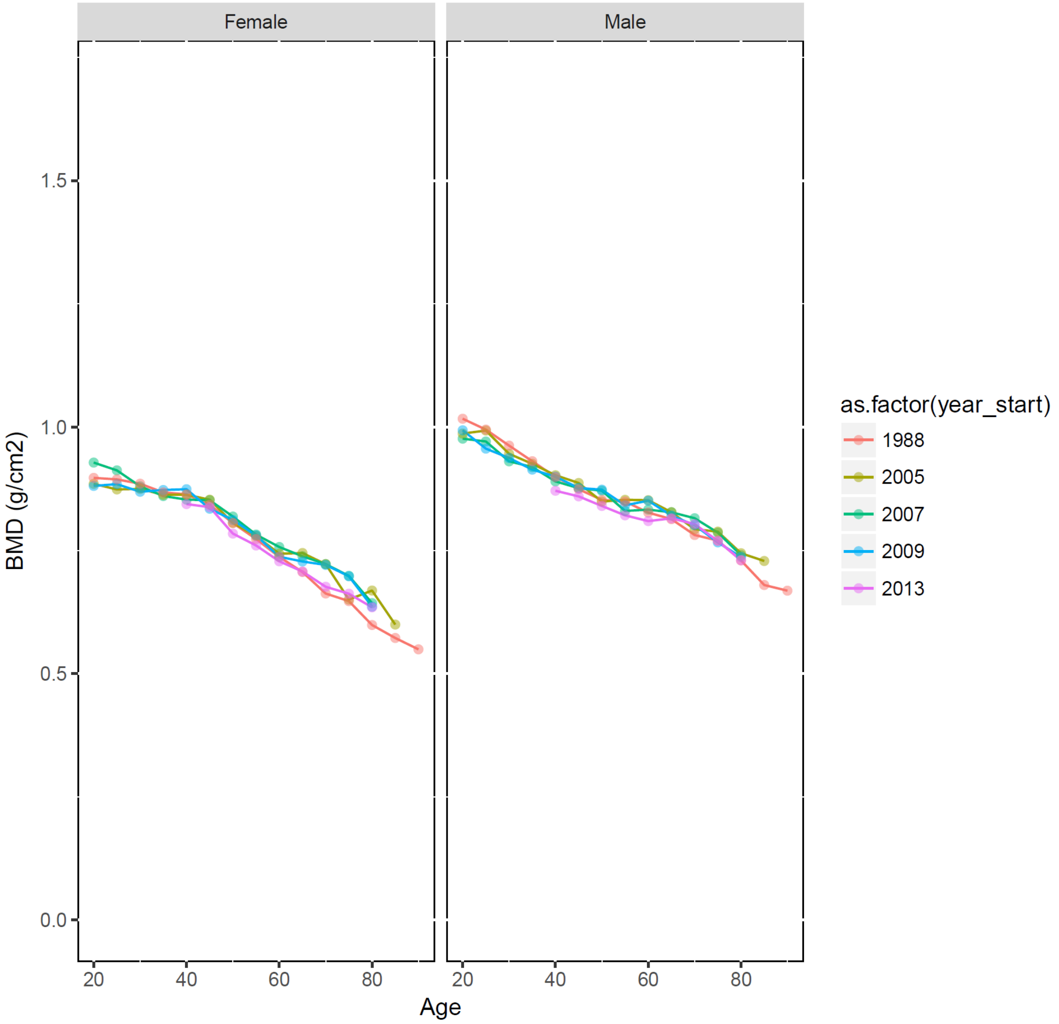

Relative Risk

Input data

To establish the relationship between low BMD and fracture risk, we conducted a systematic review for GBD 2021 to identify studies that reported fracture risk per standard deviation or per unit bone-mass density. We followed the methodologic approach previously described in terms of search strategy, inclusion and exclusion criteria, heterogeneity and risk of bias assessment, data extraction and processing.[2,3] In brief, eligible publication types included peer-reviewed longitudinal cohort studies and meta-analyses published in English that used FNBMD measured by DXA as the exposure variable, and osteoporotic fracture as the outcome of interest. Studies reporting relative risk in an osteoporotic group

versus a non-osteoporotic group were excluded. A review of the literature in PubMed from 2010 to 2020 using the following search string yielded 611 results, six of which ultimately met our inclusion criteria:

(((bone mineral density OR bone mineral densities OR bone density) AND

(mean OR average) AND risk) AND fracture).

These six studies were supplemented with additional studies from a meta-analysis that has been previously used to estimate the relationship between low BMD and fragility fracture[4]. Out of twelve total studies reported in this previous meta-analysis, six met our inclusion criteria; the remaining six were excluded because they either reported cohorts that used a measure of exposure other than BMD measured at the femoral neck, or used mortality as an outcome of interest instead of fracture.

Figure 2: PRISMA diagram of BMD RR systematic review from 2019

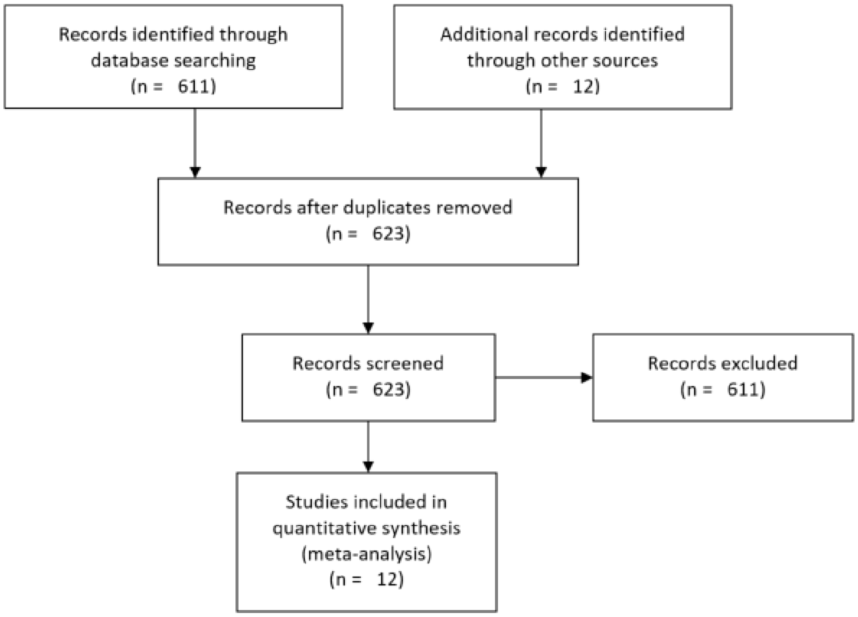

Table 4: Relative risk input data

Input data	Relative risk
Source count (total)	12
Number of countries with data	9

Nine countries were represented among the 12 cohort studies included in the meta-analysis.[5-16]

Modeling strategy

We used study covariates for studies that reported the relative risk of low BMD on hip fracture and non-hip fractures. The mean and standard error for the coefficients were calculated using the MR-BRT crosswalk adjustment method.

We investigated whether adding covariates for the percentage of the cohort that was male (which was always either 0 or 100 percent) or a cubic spline on cohort mean age improved the model fit. An adjustment for percentage male was not included in the final model, as we did not find a significant difference between relative risks for males and females. After testing four iterations with two and three knots placed evenly or by data frequency, it was clear that there was not a reliable relationship between cohort mean age and relative risk. As a result, the age spline was also not included in the final model.

Betas and exponentiated values (which can be interpreted as the relative risks) for the remaining hip and non-hip fracture covariates are shown in the table below:

Table 3. MR-BRT Crosswalk Results for RR of Fracture due to low BMD

Data input	Gamma	Beta coefficient, log (95% CI)	RR per SD unit of BMD
Hip fracture	0.13	0.77 (0.45 to 1.08)	2.18 (1.66 to 2.80)
Non-hip fracture		0.57 (0.30 to 0.84)	1.79 (1.41 to 2.23)

Attributable Burden

Osteoporotic non-hip fractures include fractures of vertebrae, clavicle, scapula, humerus, skull, sternum, face bone, radius or ulna, femur, patella, tibia, fibula, ankle, and pelvis. The attributable burden of fractures due to low BMD for adults 40 years of age and older was calculated by comparing the observed distribution of sFNBMD to the counterfactual distribution for each age, sex, year, and cause according to the following formula:

$$PAF_{oasgt} = \frac{\int_{x=l}^{u} RR_o(x)P_{asgt}(x)dx - RR_o(TMREL_{as})}{\int_{x=l}^{u} RR_o(x)P_{asgt}(x)dx}$$

where PAF_{oasgt} represents the population attributable fraction for outcome o (i.e. hip or non-hip fracture), age group a, sex s, location g, and year t; RR_o (x) is the relative risk at exposure level x for outcome o with the lowest observed exposure as l and the highest as u; P_{asgt}(x) is the exposure at level x for age group a, sex s, location g, and year t; and $TMREL_{as}$ is the TMREL (described above under *Theoretical minimum-risk exposure level*) for age group a and sex s. PAFs were estimated in five-year age groups from 40 to 44 until 95+.

To obtain the attributable burden of the root causes of fractures due to low BMD, two additional processes are required.

First, we calculated the proportion of injury deaths that are due to fractures. We assumed that hip fracture and some non-hip fractures (any fractures apart from those of fingers and toes) are potentially fatal fractures. However, in mortality registration systems, deaths due to injuries are assigned to the

cause of injury (e.g., falls or road injury) rather than to the nature of injury (e.g., fracture or traumatic brain injury). To derive an estimate of the proportion of deaths attributable to the fractures resulting from each of the six categories of injury, inpatient hospital data with detailed diagnostic codes were analysed to estimate the proportion of injury deaths during admission that could be ascribed to fractures. The analysis was restricted to cases that were dual-coded with both the cause of injury ("E-code") and nature of injury ("N-code") that died during the inpatient episode. For those with a code for a fracture, the death was assigned to the fracture in the absence of a more severe injury code that could better explain the death (e.g. moderate to severe head trauma, spinal cord lesion, and intra-abdominal or thoracic organ damage). We then collapsed all deaths over E-code to determine the ratio of deaths attributable to fracture versus non-fracture injuries. This ratio was applied to the PAFs consistently across all countries to obtain the YLLs from each of the six outcomes attributable to low BMD.

To derive an estimate of the proportion of non-fatal burden attributable to the fractures resulting from each of the six categories of injury, we used the E- to N-code matrix generated from dual-coded (E-code/N-code) patient-level data in the GBD injury analyses to determine the proportion of each E-code that results in a certain N-code (See Part 2. Methodologic Summary for Estimation of Injuries). The hip and non-hip fracture PAFs were applied to the appropriate combinations of external cause and fracture estimates of YLD and then summed together to produce a single estimate. This approach was applied consistently across all countries. Details of the methodology are included below.

Below is the list of injuries for which a PAF was calculated. The six categories of injury evaluated in the present analyses are shown in bold:

- Transport injuries
    - **Road injuries**
        - Road injuries
        - Pedestrian road injuries
        - Cyclist road injuries
        - Motorcyclist road injuries
        - Motor vehicle road injuries
        - Other road injuries
    - **Other transport injuries**
- Unintentional injuries
    - **Falls**
    - **Animal contact (non-venomous)**
    - **Exposure to mechanical forces**
        - Other exposure to mechanical forces
- **Interpersonal violence**
    - Assault by other means than firearms

References

1. Lu Y, Fuerst T, Hui S, et al. Standardization of bone mineral density at femoral neck, trochanter and Ward's triangle. Osteoporos Int 2001;12:438–44.

2. Sanchez-Riera L, Carnahan E, Vos T, Veerman L, Norman R, Lim SS, et al. The global burden attributable to low bone mineral density. Ann Rheum Dis. 2014;73(9):1635-45.
3. GBD 2015 Risk Factors Collaborators. Global, regional, and national comparative risk assessment of 79 behavioural, environmental and occupational, and metabolic risks or clusters of risks, 1990-2015: a systematic analysis for the Global Burden of Disease Study 2015. Lancet. 2016;388(10053):1659-724.
4. Johnell O, Kanis JA, Oden A, et al. Predictive value of BMD for hip and other fractures. J Bone Miner Res. 20(7):1185-94
5. Berger C, Langsetmo L, Joseph L, Hanley DA, Davison KS, Josse RG, Prior JC, Kreiger N, Tenenhouse A, Goltzman D, CaMos Research Group. Association between change in BMD and fragility fracture in women and men. J Bone Miner Res. 2009; 24(2): 361-70.
6. Bow CH, Tsang SW, Loong CH, Soong CS, Yeung SC, Kung AW. Bone mineral density enhances use of clinical risk factors in predicting ten-year risk of osteoporotic fractures in Chinese men: the Hong Kong Osteoporosis Study. Osteoporosis Int. 2011; 22(11): 2799-807.
7. Chalhoub D, Orwoll ES, Cawthon PM, Ensrud KE, Boudreau R, Greenspan S, Newman AB, Zmuda J, Bauer D, Cummings S, Cauley JA, Osteoporotic Fractures in Men (MrOS) Study Research Group. Areal and volumetric bone mineral density and risk of multiple types of fracture in older men. Bone. 2016;92:100-106
8. Crandall CJ, Hovey KM, Andrews CA, Cauley JA, Manson JE, Wactawski-Wende J, Wright NC, Li W, Beavers K, Curtis JR, LeBoff MS. Bone mineral density as a predictor of subsequent wrist fractures: Findings from the Women's Health Initiative Study. J Clin Endocrinal Metab. 2015; 100(11): 4315-24.
9. Dargent-Molina P, Favier F, Grandjean H, Baudoin C, Schott AM, Hausherr E, Meunier PJ, Bréart G. Fall-related factors and risk of hip fracture: the EPIDOS prospective study. Lancet. 1996; 348(9021): 145-9.
10. Fujiwara S, Kasagi F, Masunari N, Naito K, Suzuki G, Fukunaga M. Fracture Prediction From Bone Mineral Density in Japanese Men and Women. J Bone Miner Res. 2003; 18(8): 1547-53.
11. Huopio J, Kröger H, Honkanen R, Saarikoski S, Alhava E. Risk factors for perimenopausal fractures: a prospective study. Osteoporos Int. 2000; 11(3): 219-27.
12. Kwok AW, Gong JS, Wang YX, Leung JC, Kwok T, Griffith JF, Leung PC. Prevalence and risk factors of radiographic vertebral fractures in elderly Chinese men and women: Results of Ms. OS (Hong Kong) and Ms. OS (Hong Kong) studies. Osteoporosis Int. 2013; 23(3); 877-85.
13. Melton LJ, Crowson CS, O'Fallon WM, Wahner HW, Riggs BL. Relative contributions of bone density, bone turnover, and clinical risk factors to long-term fracture prediction. J Bone Miner Res. 2003; 18(2): 312–8.
14. Nguyen TV, Eisman JA, Kelly PJ, Sambrook PN. Risk factors for osteoporotic fractures in elderly men. Am J Epidemiol. 1996; 144(3): 255–63.
15. Sheu Y, Cauley JA, Patrick AL, Wheeler VW, Bunker CH, Zmuda JM. Risk factors for fracture in middle-age and older-age men of African descent. J Bone Miner Res. 2014; 29(1): 234-41.
16. Shin CS, Kim MJ, Shim SM, Kim JT, Yu SH, Koo BK, Cho HY, Choi HJ, Cho SW, Kim SW, Kim SY, Yang SO, Cho NH. The prevalence and risk factors of vertebral fractures in Korea. J Bone Miner Metab. 2012; 30(2): 183-192

2. Methodologic Summary for Estimation of Injuries

The methods presented below describe the overall approach to injuries estimation for the GBD study and is not limited to the fracture-related injuries data analyzed for the current manuscript.

This GBD round, the top five causes of injury in terms of the number of fractures they contribute were:

1. Falls
2. Other exposure to mechanical forces
3. Other unintentional injuries
4. Motor vehicle road injuries
5. Physical violence by other means

Flowchart

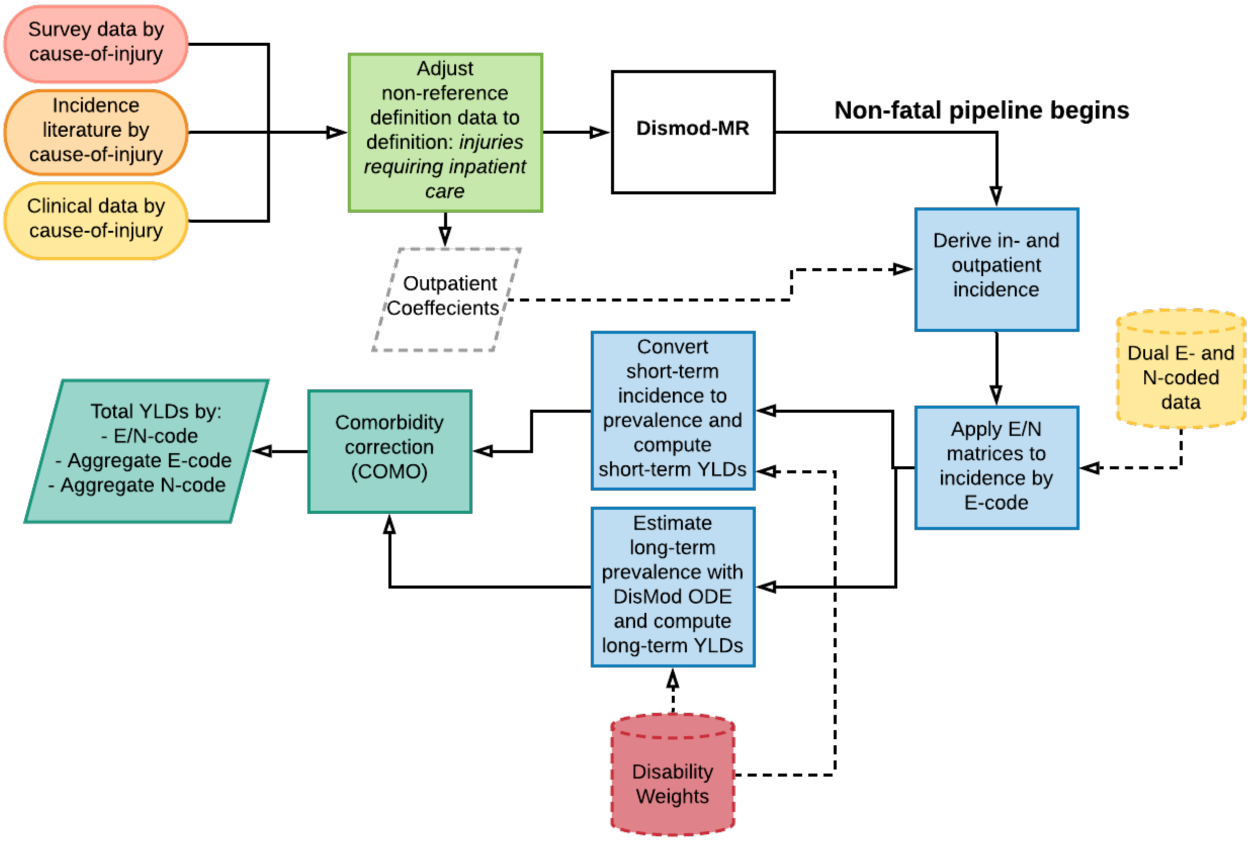

Case definition

For GBD 2021, the Injuries estimation process for non-fatal health outcomes encompasses a range of 29 causes, including transport injuries, falls, drowning, self-harm, interpersonal violence, and animal contact (excluding sexual violence, as described in a separate Appendix section). Injury incidence is defined using ICD-9 codes E000-E999 and ICD-10 chapters V to Y. Chapters S and T in ICD-10 and codes 800-999 in ICD9 are used to estimate morbidity.

Each of these 29 causes of injury can result in a variety of physical injury sequelae (eg, traumatic brain injury), which we call the “nature of injury.” Although the initial models are at the “cause of injury” level (eg, drowning), each cause of injury is distributed into cause-nature pairs to capture the disability that develops from the resulting nature of injury. We report incidence, prevalence, and years lived with disability (YLDs) due to injuries at the cause-nature pair level.

We make additional distinctions between inpatient and outpatient injuries and between short-term and long-term injuries. Inpatient injuries are defined as injuries that lead to overnight hospitalisation, whereas outpatient injuries are defined as ones treated in outpatient settings or emergency care. We define short-term injuries as injuries lasting less than one year and long-term injuries as those lasting longer than one year, at which point we assume lifelong disability.

Input data

Model inputs

To estimate morbidity from injuries, we use data from hospital records, emergency department records, insurance claims, and population-representative surveys to produce YLDs by country, year, sex, age, external cause-of-injury, and nature-of-injury category.

Data searches

GBD 2021 utilised the same data as GBD 2019 [1] with some updates to existing data and additions of new data. For GBD 2021, hospital and emergency department records were supplemented with more recent and available site-years. We incorporated a correction for access to health care facilities to account for individuals who sustain an injury but do not have access to a hospital or health care facility. This correction is based on the Healthcare Access and Quality (HAQ) Index [2].

Table 1 contains information about data coverage for each cause of injury, not including fatal discontinuities: state actor violence, exposure to forces of nature, and conflict and terrorism.

Table 1. Data inputs for injuries incidence modelling

Cause	Total sources	Countries with data
Road injuries	301	77

Pedestrian road injuries	177	22
Cyclist road injuries	186	22
Motorcyclist road injuries	179	22
Motor vehicle road injuries	187	22
Other road injuries	174	18
Other transport injuries	191	20
Falls	234	40
Drowning	204	27
Fire, heat, and hot substances	217	34
Poisonings	214	35
Poisoning by carbon monoxide	163	20
Poisoning by other means	165	21
Exposure to mechanical forces	191	24
Unintentional firearm injuries	187	20
Other exposure to mechanical forces	190	23
Adverse effects of medical treatment	346	49
Animal contact	226	33
Venomous animal contact	189	22
Non-venomous animal contact	190	23
Pulmonary aspiration and foreign body in airway	188	21
Foreign body in eyes	196	20
Foreign body in other body part	201	23
Environmental heat and cold exposure	182	24
Other unintentional injuries	168	20
Self-harm	210	29
Self-harm by firearm	164	18
Self-harm by other specified means	166	21
Interpersonal violence	209	32
Physical violence by firearm	188	21
Physical violence by sharp object	171	25
Physical violence by other means	165	22

Figure 1. Countries with incidence data, across all causes of injury. *Note that specific causes' data availability may vary, as demonstrated via Table 1.*

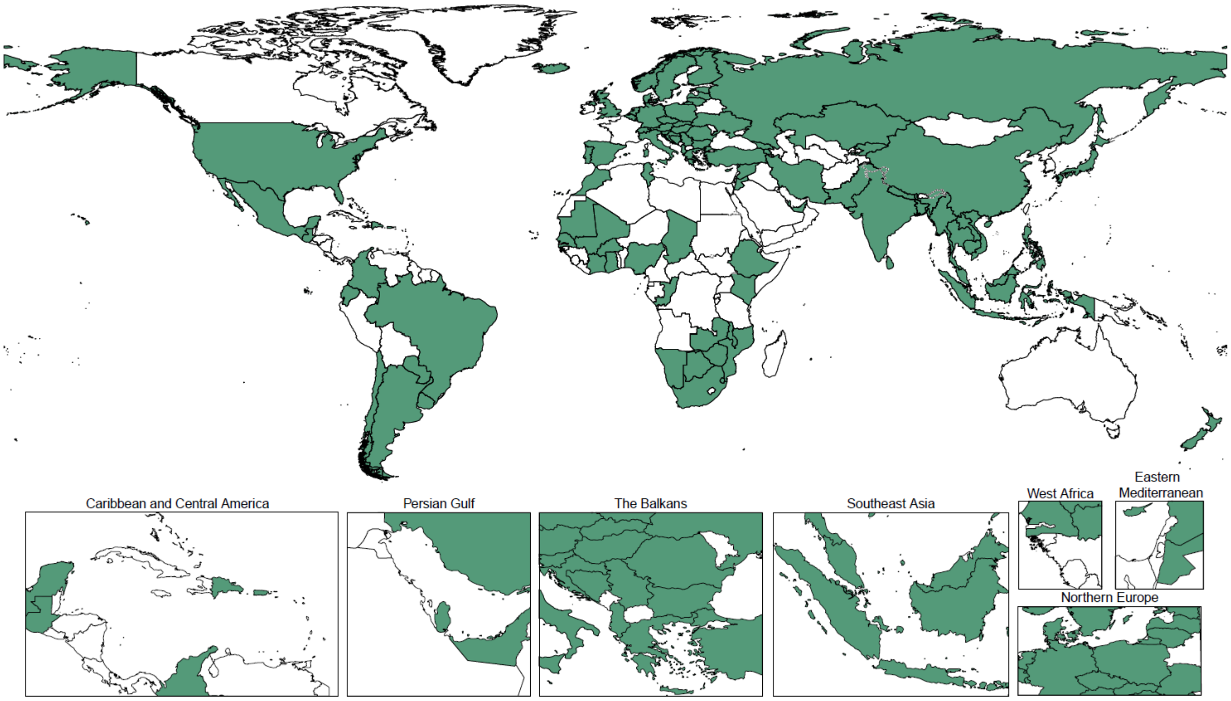

Modelling strategy

As in previous GBD iterations, two categories of injury severity were separately modelled for each injury: injuries warranting inpatient care (inpatient) and injuries warranting other health care (outpatient). Injuries warranting inpatient care refer to injury cases of sufficient severity to require inpatient care if there are no restrictions in access to health care. Injuries warranting other health care refer to injury cases of sufficient severity to require health care attention but not hospitalisation. This category includes emergency department visits.

Cause-of-injury incidence

The list of unique (ie, not counting aggregate categories like road injuries or interpersonal violence) cause-of-injury categories did not change from the 29 unique causes in GBD 2019 [1].

The majority of incidence data exist for external causes of injury. Incidence for cause-of-injury categories was modelled using Bayesian meta-regression method DisMod-MR 2.1 Multiple datasets from hospital and emergency/outpatient departments, insurance claims, and surveys were fed into these incidence models. We separately estimated two categories of injury severity: inpatient and outpatient injuries.

Cause-nature matrices

Because injury disability is linked more to the nature of injury than to the cause of injury, matrices were generated to map the proportion of each cause-of-injury category that results in a particular nature-of-

injury category. These matrices are based on a collection of dual-coded (ie, both cause-of-injury and nature-of-injury coded) hospital and emergency department datasets [3]. The data for this step came from inpatient, outpatient, and emergency room discharge data from Argentina, Brazil, Bulgaria, China, Chile, Colombia, Cyprus, Czech Republic, Denmark, Egypt, Estonia, Georgia, Great Britain, Hungary, Iceland, Iran, Italy, India, Kyrgyzstan, Latvia, Malta, Mauritius, Mexico, Mozambique, Netherlands, New Zealand, Norway, Philippines, Portugal, Slovenia, Spain, Sweden, Macedonia, Uganda, United States, and Zambia. We applied our nature-of-injury severity hierarchy above to assert that every observation had one cause of injury and one nature of injury.

Dirichlet models were used to estimate all the nature-of-injury category proportions for one cause of injury simultaneously. These models allow for consistent borrowing of information across age, sex, inpatient/outpatient, and high/low-income countries and assert that the nature-of-injury proportions within a cause-of-injury category must add up to 1. One cause-nature matrix was created for each combination of injury warranting hospital admission versus injury warranting other health care, high/low-income countries (a binary variable based on GBD super-region), male/female, and age category. Applying these matrices to our cause-of-injury incidence from DisMod-MR 2.1, we produced cases of injury warranting hospital admission and incidence of injury warranting other health care by cause and nature of injury. For causes that are subsets of other causes (child and parent causes), the cause-nature matrix was applied directly to the child causes. Afterward, the incidences of the child cause-nature combinations were scaled to sum to the incidence of the parent cause.

<u>Analysis to inform nature-of-injury category hierarchy and long-term probability of injuries</u>

Similar to GBD 2019, we used follow-up data obtained from a pooled dataset of six follow-up studies from China, the Netherlands, and the US (see Table 2) [1]. These studies followed patients for at least one year after the injury. We also used the Medical Expenditure Panel Survey (MEPS) [4]. MEPS is a large-scale overlapping continuous panel survey of the US non-institutionalised population that collects information on use and cost of health care and SF-12 responses. The follow-up studies used different patient-reported outcome measures to assess health status, namely the SF-36, Version 1 SF-12, and the EQ-5D. To enable comparison across the six datasets, it was necessary to analyse the data in a standardised patient-reported outcome measure. The pooled dataset informed both the nature-of-injury category hierarchy and the long-term probability of injuries, discussed below.

Table 2. Details of injury follow-up surveys used in GBD 2021

Dataset	Year	Type of data collected	Type of patients	Setting	Sample size* and response

Guangdong follow-up survey, China [5]	2006–2007	Follow-up survey among sample of ISS patients	Patients (15+ years) who were hospitalised that had been injured by road traffic injury, fall, blunt or penetrating trauma	Based on three national injury surveillance hospitals in Zhuhai, Guangdong Province in China	998 (response 87%)
LIS follow-up survey, Netherlands[6]	2001–2002	Follow-up survey among stratified sample of ISS patients (oversampling less common, severe injuries)	Patients (15+ years) who visited the Emergency Department of a hospital and were discharged to the home environment and patients who were admitted to hospital	Based on 17 public hospitals in the Netherlands	8,564 (response 37%)
LIS follow-up survey, Netherlands [7]	2007–2008	Follow-up survey among stratified sample of ISS patients (oversampling less common, severe injuries)	Patients (15+ years) who visited the Emergency Department of a hospital and were discharged to the home environment and patients who were admitted to hospital	Based on 15 public hospitals in the Netherlands	8,057 (response 36%)
NSCOT – National study on Costs and Outcomes of Trauma, USA [8]	2001–2002	A prospective cohort study was conducted among a sample of adult trauma patients treated at Level I trauma centres and non-trauma centre hospitals	Patients treated for a moderate to severe injury (as defined by at least one injury of an Abbreviated Injury Scale (AIS) score of 3 or greater	Based on 69 hospitals in 12 states in the US	5,191 (response 61%)
SCTBIFR – South Carolina Traumatic Brain injury Follow-up Registry, USA [9]	1999–2002	A prospective cohort study was conducted among injured in-patients with a traumatic brain injury-related injury	Patients (15+ years) who were admitted to hospitals and met the CDC case definition of TBI – trauma to the head associated with altered consciousness, amnesia, neurological abnormalities, skull fracture, intracranial lesion, or death	Discharged from all nonfederal in-state acute care hospitals	7,613 (response 28%)
Burns outcome study, Netherlands [10]	2003–2006	A multicentre prospective cohort was conducted among adult (severe) burn patients	Injury patients who sustained severe burns	Three public hospitals with specialised burn units.	311 (response 78%)

*number of patients that met the inclusion criteria; response rate = percentage of patients who responded to the follow-up survey (in case of multiple follow-up times the response rate of the first follow-up moment is reported).

Probability of permanent health loss

Disability due to injury was assumed to affect all cases in the short-term with a proportion having long-term (permanent) outcomes. The probability of long-term outcomes was needed to estimate the

incidence and subsequently the prevalence of cases with permanent health loss. In our conceptual model, individuals who suffer from a non-fatal injury will, in the long-term, return to either full or partial health. If one-year post-injury patients return to a health status with more disability than their pre-injury health status, injury patients are assumed to have permanent disability from their injury. The difference between the pre-injury health states and health status one year after injury is assumed to be their permanent level of injury-related disability. We assessed the probability of developing permanent health loss using the pooled dataset of follow-up studies [5-10] and the MEPS [4] that were also used to generate the nature-of-injury hierarchy. We developed estimates of the probability of permanent health loss by nature-of-injury category, injury severity level (injuries warranting inpatient admission and injuries warranting other health care), and age.

Duration of short-term health loss

To determine the duration for treated cases of short-term injury, we analysed patient responses from two Dutch Injury Surveillance System follow-up studies conducted from 2001–2003 and 2007–2009 [11]. These studies collected data at 2.5, 5, 9, and 24 months post-injury to determine whether injury patients were still experiencing problems due to their injury. If not, the patients were asked how many days they had experienced problems. The injury patients that still reported having problems one year after the injury were assumed to be captured in our analysis of permanent disability. The duration for treated cases of short-term injury was estimated for injuries warranting inpatient admission and injuries warranting other health care separately.

Calculation of prevalence from incidence data – short-term injury

For short-term injury outcomes, which were assumed to be less than one year in duration, the prevalence for each cause-of-injury/nature-of-injury/severity-level grouping was approximated by the incidence for that grouping multiplied by the associated nature-of-injury/severity-level-specific duration.

Calculation of prevalence from incidence data – permanent health loss

For permanent health loss, we assumed no remission and thus integrated incidence over time to arrive at prevalence estimates. We used DisMod-MR ODE (ie, the “engine” of DisMod-MR 2.1) to carry out this integration for each combination of cause of injury and nature of injury by country, year, and sex.

Table 3. GBD fracture healthstates and their corresponding disability weights [12]

Healthstate	Healthstate description	Disability weight
**Fracture of clavicle, scapula or humerus**	has a broken shoulder bone, which is painful and swollen. The person cannot use the affected arm and has difficulty with getting dressed.	0.035 (0.021-0.053)

**(short or long term, with or without treatment)**		
**Fracture of face bone (short or long term, with or without treatment)**	has a broken cheek bone or a broken nose or chipped teeth, with swelling and severe pain.	0.067 (0.044-0.097)
**Fracture of foot bones (short term, with or without treatment)**	has a broken foot bone, which causes pain, swelling, and difficulty walking.	0.026 (0.015-0.043)
**Fracture of foot bones (long term, without treatment)**	had a broken foot in the past that did not heal properly. The person now has pain in the foot and has some difficulty walking.	0.026 (0.015-0.042)
**Fracture of hand (short term, with or without treatment)**	has a broken hand, causing pain and swelling.	0.010 (0.005-0.019)
**Fracture of hand (long term, without treatment)**	has stiffness in the hand and a weak grip.	0.014 (0.007-0.025)
**Fracture of neck of femur (short term, with or without treatment)**	has broken a hip and is in pain. The person cannot stand or walk, and needs help washing, dressing, and going to the toilet.	0.258 (0.172-0.356)
**Fracture of neck of femur (long term, with treatment)**	had a broken hip in the past, which was fixed with treatment. The person can only walk short distances, has discomfort when moving around, and has some difficulty in daily activities.	0.058 (0.038-0.084)
**Fracture of neck of femur (long term, without treatment)**	had a broken hip bone in the past, which was never treated and did not heal properly. The person cannot get out of bed and needs help washing and going to the toilet.	0.402 (0.269-0.541)
**Fracture of patella, tibia or fibula or ankle (short term, with or without treatment)**	has a broken shin bone, which causes severe pain, swelling, and difficulty walking.	0.050 (0.032-0.075)
**Fracture of patella, tibia or fibula or ankle (long term, with or without treatment)**	had a broken shin bone in the past that did not heal properly. The person has pain in the knee and ankle, and has difficulty walking.	0.055 (0.036-0.081)
**Fracture of pelvis (short term, with or without treatment)**	has a broken pelvis bone, with swelling and bruising. The person has severe pain, and cannot walk or do daily activities.	0.279 (0.188-0.384)

**Fracture of pelvis (long term, with or without treatment)**	had a broken pelvis in the past and now walks with a limp. There is often pain in the back and groin, and when urinating and sitting for a long time.	0.182 (0.123-0.253)
**Fracture of radius or ulna (short term, with or without treatment)**	has a broken forearm, which causes severe pain, swelling, and limited movement.	0.028 (0.016-0.046)
**Fracture of radius or ulna (long term, without treatment)**	had a broken forearm in the past that did not heal properly, causing some pain and limited movement in the elbow and wrist. The person has difficulty with daily activities such as dressing.	0.043 (0.028-0.064)
**Fracture of skull (short or long term, with or without treatment)**	has a broken skull, but does not have brain damage. The broken area is painful and swollen.	0.071 (0.048-0.100)
**Fracture of sternum and/or fracture of one or two ribs (short or long term, with or without treatment)**	has a broken rib that causes severe pain in the chest, especially when breathing in. The person has difficulty with daily activities such as dressing.	0.103 (0.068-0.145)
**Fracture of vertebral column (short or long term, with or without treatment)**	has broken back bones and is in pain, but still has full use of arms and legs.	0.111 (0.075-0.156)
**Fracture of femur, other than femoral neck (short term, with or without treatment)**	has a broken thigh bone. The person has severe pain and swelling and cannot walk.	0.111 (0.074-0.156)
**Fracture of femur, other than femoral neck (long term, with or without treatment)**	had a broken thigh bone in the past, which was never treated and did not heal properly. The person now has a limp and discomfort when walking.	0.042 (0.027-0.063)

References

1. GBD 2019 Diseases and Injuries Collaborators. Global burden of 369 diseases and injuries in 204 countries and territories, 1990–2019: a systematic analysis for the Global Burden of Disease Study 2019. The Lancet;396(10258): 1204 – 22.

2. GBD 2017 SDG Collaborators. Measuring progress from 1990 to 2017 and projecting attainment to 2030 of the health-related Sustainable Development Goals for 195 countries and territories: a systematic analysis for the Global Burden of Disease Study 2017. The Lancet 2018; 392:2091–138.

3. GBD 2016 Traumatic Brain Injury and Spinal Cord Injury Collaborators. Global, regional, and national burden of traumatic brain injury and spinal cord injury, 1990–2016: a systematic analysis for the Global Burden of Disease Study 2016. The Lancet Neurology. 2019;18(1):56-87.

4. Agency for Healthcare Research and Quality. United States Medical Expenditure Panel Survey. Rockville, United States: Agency for Healthcare Research and Quality. Available at https://meps.ahrq.gov/mepsweb/. Accessed 19 September 2024

5. Chinese Center for Disease Control and Prevention (CCDC). China Zhuhai Study 2006-2007 - China CDC. Available at https://ghdx.healthdata.org/record/china-zhuhai-study-2006-2007-china-cdc. Accessed 19 September 2024

6. Consumer Safety Institute (Netherlands). Netherlands Injury Surveillance System 2002. Available at https://ghdx.healthdata.org/organizations/consumer-safety-institute-netherlands. Accessed 19 September 2024

7. Consumer Safety Institute (Netherlands). Netherlands Injury Surveillance System 2008. Available at https://ghdx.healthdata.org/organizations/consumer-safety-institute-netherlands. Accessed 19 September 2024

8. Mackenzie EJ, Rivara FP, Jurkovich GJ, et al. The National Study on Costs and Outcomes of Trauma. J Trauma 2007; 63: S54-67

9. Centers for Disease Control and Prevention (CDC), Medical University of South Carolina, South Carolina Department of Disabilities and Special Needs, South Carolina Department of Health and Environmental Control. South Carolina Traumatic Brain Injury Follow-up Registry 1999-2013. USA

10. van Loey NE, van Beeck EF, Faber BW, van de Schoot R, Bremer M. Health-related quality of life after burns: a prospective multicentre cohort study with 18 months follow-up. J Trauma. 2011; 72(2): 513-520.

11. Polinder S, van Beeck EF, Essink-Bot ML, Toet H, Looman CW, Mulder S, Meerding WJ. Functional outcome at 2.5, 5, 9, and 24 months after injury in the Netherlands. J Trauma. 2007; 62(1): 133-41

12. Salomon JA, Haagsma JA, Davis A, et al. Disability weights for the Global Burden of Disease 2013 study. The Lancet Global Health. 2015 Nov 1;3(11):e712–23
