# Supplementary material for: The global, regional, and national burden attributable to low bone mineral density, 1990–2020: an analysis of a modifiable risk factor from the Global Burden of Disease Study 2021
Source: Lancet Rheumatol. 2025 Sep 16;7(12):e873–94. doi: 10.1016/S2665-9913(25)00105-5 (PMC12623303; doi:10.1016/S2665-9913(25)00105-5)
Supplement: Supplementary appendix 2 [file mmc2.pdf]

THE LANCET
Rheumatology

Supplementary appendix 2

This appendix formed part of the original submission and has been peer reviewed. We post it as supplied by the authors.

Supplement to: GBD 2021 Low Bone Mineral Density Collaborators. The global, regional, and national burden attributable to low bone mineral density, 1990–2020: an analysis of a modifiable risk factor from the Global Burden of Disease Study 2021. *Lancet Rheumatol* 2025; published online Sept 16. https://doi.org/10.1016/S2665-9913(25)00105-5.

Appendix 2: Authorship appendix to "The global burden attributable to low bone mineral density, 1990 to 2020: An updated and expanded analysis of a modifiable risk factor from the Global Burden of Disease Study 2021"

This appendix provides further authorship detail for "The global burden attributable to low bone mineral density, 1990 to 2020: An updated and expanded analysis of a modifiable risk factor from the Global Burden of Disease Study 2021"

Table of Contents

GBD 2021 Low Bone Mineral Density Collaborators 2

Affiliations 3

Authors' Contributions 10

Providing data or critical feedback on data sources 10

Developing methods or computational machinery 10

Providing critical feedback on methods or results 10

Drafting the work or revising it critically for important intellectual content 11

Managing the estimation or publications process 12

GBD 2021 Low Bone Mineral Density Collaborators

Evelyn Hsieh, Dana Bryazka, Kanyin Liane Ong, Phoebe-Anne Rhinehart, Ewerton Cousin, Hailey Hagins, Cyrus Cooper, Marita Cross, Garland T Culbreth, Karsten E Dreinhoefer, Philippe Halbout, Jacek A Kopec, Sneha Ingle Nicholson, Daniel Prieto-Alhambra, Anthony D Woolf, Theo Vos, Yohannes Habtegiorgis Abate, Sherief Abd-Elsalam, Meriem Abdoun, Mohamed Abouzid, Eman Abu-Gharbieh, Salahdein Aburuz, Abiola Victor Adepoju, Qorinah Estiningtyas Sakilah Adnani, Aqeel Ahmad, Haroon Ahmed, Luai A Ahmed, Syed Mahfuz Al Hasan, Tariq A Alalwan, Rasmieh Mustafa Al-Amer, Hediyeh Alemi, Abid Ali, Yaser Mohammed Al-Worafi, Reza Amani, Abhishek Anil, Jalal Arabloo, Aleksandr Y Aravkin, Demelash Areda, Brhane Berhe Aregawi, Mohammad Asghari-Jafarabadi, Seyyed Shamsadin Athari, Sina Azadnajafabad, Ahmed Y Azzam, Ashish D Badiye, Nasser Bagheri, Sara Bagherieh, Saliu A Balogun, Maciej Banach, Shirin Barati, Pankaj Bhardwaj, Sonu Bhaskar, Gurjit Kaur Bhatti, Yasser Bustanji, Daniela Calina, Vijay Kumar Chattu, Endeshaw Chekol Abebe, Dinh-Toi Chu, Michael H Criqui, Natalia Cruz-Martins, Omid Dadras, Xiaochen Dai, Zhaoli Dai, Reza Darvishi Cheshmeh Soltani, Mohsen Dashti, Tadesse Asmamaw Dejenie, Cristian Del Bo', Edgar Denova-Gutiérrez, Vinoth Gnana Chellaiyan Devanbu, Syed Masudur Rahman Dewan, Vishal R Dhulipala, Michael Ekholuenetale, Mohamed A Elmonem, Farshid Etaee, Adeniyi Francis Fagbamigbe, Ildar Ravisovich Fakhradiyev, Ali Fatehizadeh, Alireza Feizkhah, Ginenus Fekadu, Bikila Regassa Feyisa, Florian Fischer, Abduzhappar Gaipov, Lucia Galluzzo, Mesfin Gebrehiwot, Fataneh Ghadirian, Tiffany K Gill, Kimiya Gohari, Ali Golchin, Bhawna Gupta, Sapna Gupta, Najah R Hadi, Arvin Haj-Mirzaian, Asif Hanif, Netanja I Harlianto, Ikramul Hasan, Md Saquib Hasnain, Amr Hassan, Simon I Hay, Jiawei He, Golnaz Heidari, Kamal Hezam, Yuta Hiraike, Praveen Hoogar, Chengxi Hu, Segun Emmanuel Ibitoye, Arad Iranmehr, Nahlah Elkudssiah Ismail, Masao Iwagami, Ali Jafari-Khounigh, Mihajlo Jakovljevic, Elham Jamshidi, Sathish Kumar Jayapal, Shubha Jayaram, Digisie Mequanint Jemere, Gwang Hun Jeong, Nitin Joseph, Charity Ehimwenma Joshua, Mikk Jürisson, Vidya Kadashetti, Sanjay Kalra, Morteza Abdullatif Khafaie, Himanshu Khajuria, Moien AB Khan, Javad Khanali, Shaghayegh Khanmohammadi, Moawiah Mohammad Khatatbeh, Sorour Khateri, Min Seo Kim, Oleksii Korzh, Kewal Krishan, Mukhtar Kulimbet, Vishnutheertha Kulkarni, Maria Dyah Kurniasari, Chandrakant Lahariya, Tri Laksono, Iván Landires, Kamaluddin Latief, Thao Thi Thu Le, Munjae Lee, Wei-Chen Lee, Erand Llanaj, Kashish Malhotra, Ahmad Azam Malik, Miquel Martorell, Andrea Maugeri, Hadush Negash Meles, Mohsen Merati, Tuomo J Meretoja, Tomislav Mestrovic, Alireza Mirahmadi, Nouh Saad Mohamed, Abdollah Mohammadian-Hafshejani, Ali H Mokdad, Lorenzo Monasta, Yousef Moradi, Negar Morovatdar, Shane Douglas Morrison, Ebrahim Mostafavi, Parsa Mousavi, Sumaira Mubarik, Christopher J L Murray, Sathish Muthu, Mohsen Naghavi, Pirouz Naghavi, Zuhair S Natto, Biswa Prakash Nayak, Mohammad Hadi Nematollahi, Duc Hoang Nguyen, Hien Quang Nguyen, Van Thanh Nguyen, Robina Khan Niazi, Efaq Ali Noman, Dieta Nurrika, Osaretin Christabel Okonji, Michal Ordak, Wael M S Osman, Yasamin Ostadi, Alicia Padron-Monedero, Shahina Pardhan, Pragyan Paramita Parija, Romil R Parikh, Jay Patel, Fanny Emily Petermann-Rocha, Hoang Tran Pham, Elton Junio Sady Prates, Ibrahim Qattea, Mehran Rahimi, Vafa Rahimi-Movaghar, Mosiur Rahman, Masoud Rahmati, Ivano Raimondo, Shakthi Kumaran Ramasamy, Sina Rashedi, Mohammad-Mahdi Rashidi, Salman Rawaf, Elrashdy M Redwan, Nazila Rezaei, Aly M A Saad, Umar Saeed, Amene Saghazadeh, Fatemeh Saheb Sharif-Askari, Amirhossein Sahebkar, Morteza Saki, Joseph W Sakshaug, Mohamed A Saleh, Yoseph Leonardo Samodra, Abdallah M Samy, Francesco Sanmarchi, Muhammad Arif Nadeem Saqib, Art Schuermans, Yashendra Sethi, Allen Seylani, Moyad Jamal Shahwan, Sunder Sham, Mohammed Shannawaz, Sadaf Sharfaei, Manoj Sharma, Seyed Afshin Shorofi, Emmanuel Edwar Siddig, Luís Manuel Lopes Rodrigues Silva, Jasvinder A Singh, Paramdeep Singh, Hamidreza Soleimani, Chandan Kumar

Swain, Shima Tabatabai, Jacques Lukenze Tamuzi, Razieh Tavakoli Oliaee, Seyed Mohammad Tavangar, Masayuki Teramoto, Dufera Rikitu Terefa, Jansje Henny Vera Ticoalu, Asokan Govindaraj Vaithinathan, Tommi Juhani Vasankari, Siavash Vaziri, Fang Wang, Shu Wang, Juan Xia, Naohiro Yonemoto, Chuanhua Yu, Mazyar Zahir, Hanqing Zhao, Magdalena Zielińska, Osama A Zitoun, Lyn M March,* and Lidia Sanchez-Riera.*

*Co-senior authors

Affiliations

Section of Rheumatology, Allergy and Immunology (E Hsieh MD), Department of Internal Medicine (F Etaee MD), Yale School of Medicine, New Haven, CT, USA; Section of Rheumatology (E Hsieh MD), Department of Internal Medicine, VA Connecticut Healthcare System, West Haven, CT, USA; Institute for Health Metrics and Evaluation (D Bryazka MS, K L Ong PhD, P Rhinehart BA, E Cousin PhD, H Hagins MSPH, G T Culbreth PhD, S I Nicholson MSc, Prof T Vos PhD, A Y Aravkin PhD, X Dai PhD, Prof S I Hay FMedSci, J He MSc, T Mestrovic PhD, Prof A H Mokdad PhD, Prof C J L Murray DPhil, Prof M Naghavi PhD), Department of Health Metrics Sciences, School of Medicine (E Cousin PhD, Prof T Vos PhD, A Y Aravkin PhD, X Dai PhD, Prof S I Hay FMedSci, Prof A H Mokdad PhD, Prof C J L Murray DPhil, Prof M Naghavi PhD), Department of Applied Mathematics (A Y Aravkin PhD), University of Washington, Seattle, WA, USA; MRC Lifecourse Epidemiology Unit (Prof C Cooper MD), University of Southampton, Southampton, UK; Institute of Musculoskeletal Sciences (Prof C Cooper MD), University of Oxford, Oxford, UK; Faculty of Medicine and Health (M Cross PhD, Prof L M March PhD), School of Pharmacy and Charles Perkins Centre (Z Dai PhD), School of Chemical & Biomolecular Engineering (E A Noman PhD), Institute of Bone and Joint Research (L Sanchez-Riera PhD), University of Sydney, Sydney, NSW, Australia; Global Alliance for Musculoskeletal Health, Sydney, NSW, Australia (M Cross PhD, Prof K E Dreinhoefer MD); Center of Musculoskeletal Surgery (Prof K E Dreinhoefer MD), Charité Universitätsmedizin Berlin (Charité University Medical Center Berlin), Berlin, Germany; IOF International Osteoporosis Foundation, Nyon, Switzerland (P Halbout PhD); School of Population and Public Health (J A Kopec PhD), University of British Columbia, Vancouver, BC, Canada; Arthritis Research Canada, Richmond, BC, Canada (J A Kopec PhD); Nuffield Department of Orthopaedics, Rheumatology, and Musculoskeletal Sciences (Prof D Prieto-Alhambra PhD), Oxford University, Oxford, UK; Department of Medical Informatics (Prof D Prieto-Alhambra PhD), Erasmus University Medical Center, Rotterdam, Netherlands; Osteoporosis Research Group (Prof A D Woolf FRCP), Lund University, Malmo, Sweden; Global Alliance for Musculoskeletal Health, Truro, UK (Prof A D Woolf FRCP); Department of Clinical Governance and Quality Improvement (Y H Abate MSc), Aleta Wondo General Hospital, Aleta Wondo, Ethiopia; Department of Tropical Medicine and Infectious Diseases (S Abd-Elsalam PhD), Tanta University, Tanta, Egypt; Department of Medicine (Prof M Abdoun PhD), University of Setif Algeria, Sétif, Algeria; Department of Health, Sétif, Algeria (Prof M Abdoun PhD); Department of Physical Pharmacy and Pharmacokinetics (M Abouzid PharmD), Poznan University of Medical Sciences, Poznan, Poland; Department of Clinical Sciences (Prof E Abu-Gharbieh PhD), Department of Basic Biomedical Sciences (Prof Y Bustanji PhD), Sharjah Institute of Medical Sciences (F Saheb Sharif-Askari PhD), College of Medicine (Prof M A Saleh PhD), University of Sharjah, Sharjah, United Arab Emirates; Department of Biopharmaceutics and Clinical Pharmacy (Prof E Abu-Gharbieh PhD), College of Pharmacy (Prof S Aburuz PhD), University of Jordan, Amman, Jordan; Department of Pharmacology and Therapeutics (Prof S

Aburuz PhD), Institute of Public Health (Prof L A Ahmed PhD), Family Medicine Department (M A Khan MSc), United Arab Emirates University, Al Ain, United Arab Emirates; Department of HIV and Infectious Diseases (A V Adepoju MD), Jhpiego, Abuja, Nigeria; Department of Adolescent Research and Care (A V Adepoju MD), Adolescent Friendly Research Initiative and Care, Ado Ekiti, Nigeria; Department of Public Health (Q E S Adnani PhD), Universitas Padjadjaran (Padjadjaran University), Bandung, Indonesia; College of Medicine (A Ahmad PhD), Shaqra University, Shaqra, Saudi Arabia; Department of Biosciences (H Ahmed PhD), COMSATS Institute of Information Technology, Islamabad, Pakistan; Division of Public Health Sciences, Department of Surgery (S M Al Hasan PhD), Department of Surgery (S Azadnajafabad MD), Washington University in St. Louis, St. Louis, MO, USA; Department of Biology (T A Alalwan PhD), College of Health and Sport Sciences (A G Vaithinathan MSc), University of Bahrain, Zallaq, Bahrain; School of Nursing (R M Al-Amer PhD), Department of Basic Medical Sciences (Prof M M Khatatbeh PhD), Yarmouk University, Irbid, Jordan; School of Nursing and Midwifery (R M Al-Amer PhD), Western Sydney University, Sydney, NSW, Australia; Hematology, Oncology and Stem Cell Transplantation Research Center (H Alemi MD), Iranian Research Center for HIV/AIDS (IRCHA) (O Dadras PhD), Department of Neurosurgery (A Iranmehr MD), Non-communicable Diseases Research Center (J Khanali MD, P Mousavi MD, M Rashidi MD, N Rezaei MD), School of Medicine (S Khanmohammadi MD, M Merati MD), Sina Trauma and Surgery Research Center (Prof V Rahimi-Movaghar MD), Research Center for Immunodeficiencies (A Saghazadeh MD), Department of Pathology (Prof S Tavangar MD), Tehran University of Medical Sciences, Tehran, Iran; Department of Zoology (A Ali PhD), Abdul Wali Khan University Mardan, Mardan, Pakistan; Department of Medical Sciences (Prof Y M Al-Worafi PhD), Azal University for Human Development, Sana'a, Yemen; Department of Clinical Sciences (Prof Y M Al-Worafi PhD), University of Science and Technology of Fujairah, Fujairah, United Arab Emirates; Interdisciplinary Graduate Program in Human Toxicology (R Amani DVM), University of Iowa, Iowa City, IA, USA; Holden Comprehensive Cancer Center (R Amani DVM), University of Iowa Hospitals and Clinics, Iowa City, IA, USA; Department of Pharmacology (A Anil MD), Department of Community Medicine and Family Medicine (Prof P Bhardwaj MD), School of Public Health (Prof P Bhardwaj MD), All India Institute of Medical Sciences, Jodhpur, India; All India Institute of Medical Sciences, Bhubaneswar, India (A Anil MD); Health Management and Economics Research Center (J Arabloo PhD), Iran University of Medical Sciences, Tehran, Iran; College of Art and Science (D Areda PhD), Ottawa University, Surprise, AZ, USA; School of Life Sciences (D Areda PhD), Arizona State University, Tempe, AZ, USA; College of Medicine and Health Sciences (B B Aregawi PhD), Department of Medical Laboratory Sciences (H N Meles MSc), Adigrat University, Adigrat, Ethiopia; Cabrini Research (Prof M Asghari-Jafarabadi PhD), Cabrini Health, Malvern, VIC, Australia; School of Public Health and Preventive Medicine (Prof M Asghari-Jafarabadi PhD), Monash University, Melbourne, VIC, Australia; Department of Immunology (S S Athari PhD), Zanjan University of Medical Sciences, Zanjan, Iran; Leeds Institute of Rheumatic and Musculoskeletal Medicine (S Azadnajafabad MD), School of Dentistry (J Patel BSc), University of Leeds, Leeds, UK; ASIDE Healthcare, Lewes, DE, USA (A Azzam MD); Faculty of Medicine (A Azzam MD), October 6 University, 6th of October City, Egypt; Department of Forensic Science (A D Badiye PhD), Government Institute of Forensic Science Nagpur, Nagpur, India; Rashtrasant Tukadoji Maharaj Nagpur University, Nagpur, India (A D Badiye PhD); Health Research Institute (Prof N Bagheri PhD), University of Canberra, Canberra, ACT, Australia; School of Medicine (S Bagherieh BSc), Isfahan University of Medical Sciences, Isfahan, Iran; Goldfields University Department of Rural Health (S A Balogun PhD), Curtin University, Kalgoorlie, WA, Australia; Department of Hypertension (Prof M Banach PhD), Medical University of Lodz, Lodz, Poland; Polish Mothers' Memorial Hospital Research Institute, Lodz, Poland (Prof M Banach PhD); Department of

Anatomy (S Barati PhD), Saveh University of Medical Sciences, Saveh, Iran; Global Health Neurology Lab (S Bhaskar MD), NSW Brain Clot Bank, Sydney, NSW, Australia; Division of Cerebrovascular Medicine and Neurology (S Bhaskar MD), National Cerebral and Cardiovascular Center, Suita, Japan; Department of Medical Lab Technology (Prof G K Bhatti PhD), University Centre for Research and Development (S Kalra DM), Chandigarh University, Mohali, India; School of Pharmacy (Prof Y Bustanji PhD), The University of Jordan, Amman, Jordan; Department of Clinical Pharmacy (Prof D Calina PhD), University of Medicine and Pharmacy of Craiova, Romania, Craiova, Romania; Temerty Faculty of Medicine (V K Chattu MD), University of Toronto, Toronto, ON, Canada; Department of Community Medicine (V K Chattu MD), Datta Meghe Institute of Medical Sciences, Sawangi, India; Department of Medical Biochemistry (E Chekol Abebe MSc), Debre Tabor University, Debre Tabor, Ethiopia; The Interdisciplinary Research Group on Biomedicine and Health (D Chu PhD), Faculty of Applied Sciences (D Chu PhD), VNU International School (VNUIS), Hanoi, Viet Nam; Department of Family Medicine and Public Health (Prof M H Criqui MD), University of California San Diego, La Jolla, CA, USA; Life and Health Sciences Research Institute (ICVS) (Prof N Cruz-Martins PhD), University of Minho, Braga, Portugal; Institute for Research and Innovation in Health (i3S) (Prof N Cruz-Martins PhD), University of Porto, Porto, Portugal; Research Center for Child Psychiatry (O Dadras PhD), University of Turku, Turku, Finland; School of Population Health (Z Dai PhD), University of New South Wales, Sydney, NSW, Australia; Department of Environmental Health (R Darvishi Cheshmeh Soltani PhD), Arak University of Medical Sciences, Arak, Iran; Immunology Research Center (M Dashti MD), Road Traffic Injury Research Center (A Jafari-Khounigh PhD), Cardiovascular Research Center (M Rahimi MD), Tabriz University of Medical Sciences, Tabriz, Iran; Department of Medical Biochemistry (T A Dejenie MSc), University of Gondar, Gondar, Ethiopia; Department of Food, Environmental and Nutritional Sciences (C Del Bo' PhD), Università degli Studi di Milano (University of Milan), Milan, Italy; Center for Nutrition and Health Research (E Denova-Gutiérrez DSc), National Institute of Public Health, Cuernavaca, Mexico; Chettinad Hospital & Research Institute (Prof V G C Devanbu MD), Chettinad Academy of Research and Education, Chennai, India; Department of Pharmacy (S M RDewan PhD), United International University, Dhaka, Bangladesh; Pharmacology Division (S M R Dewan PhD), Center for Life Sciences Research Bangladesh, Dhaka, Bangladesh; University of South Carolina, Columbia, SC, USA (V R Dhulipala MD); Faculty of Science and Health (M Ekholuenetale PhD), University of Portsmouth, Hampshire, UK; Department of Clinical and Chemical Pathology (Prof M A Elmonem PhD), Department of Neurology (Prof A Hassan MD), Cairo University, Cairo, Egypt; Department of Epidemiology and Medical Statistics (A F Fagbamigbe PhD), Department of Health Promotion and Education (S E Ibitoye PhD), University of Ibadan, Ibadan, Nigeria; Research Centre for Healthcare and Community (A F Fagbamigbe PhD), Coventry University, Coventry, UK; Director of the Scientific and Technological Park (I R Fakhradiyev PhD), Research and Publication Activity Division (M Kulimbet MSc), Kazakh National Medical University, Almaty, Kazakhstan; Department of Medicine (I R Fakhradiyev PhD), Korea University, Seoul, South Korea; School of Engineering (A Fatehizadeh PhD), Edith Cowan University, Joondalup, WA, Australia; Department of Social Medicine and Epidemiology (A Feizkhah MD), Guilan University of Medical Sciences, Rasht, Iran; Department of Infectious Diseases and Public Health (G Fekadu PhD), City University of Hong Kong, Hong Kong, China; Department of Pharmacy (G Fekadu PhD), Institute of Health Sciences (B R Feyisa MPH), Department of Public Health (D R Terefa MSc), Wollega University, Nekemte, Ethiopia; Jimma University, Jimma, Ethiopia (B R Feyisa MPH); Institute of Public Health (F Fischer PhD), Charité Universitätsmedizin Berlin (Charité Medical University Berlin), Berlin, Germany; Department of Medicine (A Gaipov PhD), Nazarbayev University, Astana, Kazakhstan; Department of Cardiovascular, Endocrine-metabolic

Diseases, and Aging (L Galluzzo MA), ISS - Italian National Institute of Health, Rome, Italy; Department of Environmental Health (M Gebrehiwot DSc), Wollo University, Dessie, Ethiopia; School of Nursing and Midwifery (F Ghadirian PhD), Obesity Research Center (A Haj-Mirzaian MD), Social Determinants of Health Research Center (J Khanali MD, M Rashidi MD), Department of Orthopedics (A Mirahmadi MD), School of Medicine (Y Ostadi MD), Department of Medical Education (S Tabatabai PhD), Shahid Beheshti University of Medical Sciences, Tehran, Iran; Adelaide Medical School (T K Gill PhD), University of Adelaide, Adelaide, SA, Australia; Department of Biostatistics (K Gohari MS), Tarbiat Modares University, Tehran, Iran; Quantitative Department (K Gohari MS), Department of Epidemiology (S Khanmohammadi MD, S Rashedi MD, H Soleimani MD), Non-Communicable Diseases Research Center (NCDRC), Tehran, Iran; Department of Applied Cell Sciences (A Golchin PhD), Cellular and Molecular Medicine Institute (A Golchin PhD), Urmia University of Medical Sciences, Urmia, Iran; Department of Public Health (B Gupta PhD), Torrens University Australia, Melbourne, VIC, Australia; Department of Toxicology (S Gupta MSc), Shriram Institute for Industrial Research, Delhi, India; Department of Clinical Pharmacology and Medicine (Prof N R Hadi PhD), University of Kufa, Najaf, Iraq; Department of Radiology (A Haj-Mirzaian MD), Massachusetts General Hospital, Boston, MA, USA (M Kim MD); Sakarya University, Turkey, Sakarya, Türkiye (A Hanif PhD); Faculty of Medicine (N I Harlianto MD), Utrecht University, Utrecht, Netherlands; Department of Radiology (N I Harlianto MD), University Medical Center Utrecht, Utrecht, Netherlands; Department of Pharmaceutical Technology (I Hasan MPharm), University of Dhaka, Dhaka, Bangladesh; Department of Pharmacy (Prof M S Hasnain PhD), Marwadi University, Rajkot, India; Independent Consultant, Santa Clara, CA, USA (G Heidari MD); Department of Microbiology (K Hezam PhD), Faculty of Applied Sciences (E A Noman PhD), Taiz University, Taiz, Yemen; School of Medicine (K Hezam PhD), Nankai University, Tianjin, China; Graduate School of Medicine (Y Hiraike PhD), University of Tokyo, Tokyo, Japan; School of Social Sciences (P Hoogar PhD), The Apollo University, Chittoor, India; Department of Psychology (C Hu PhD), Tsinghua University, Beijing, China; Department of Clinical Pharmacy & Pharmacy Practice (Prof N E Ismail PhD), Asian Institute of Medicine, Science and Technology, Bedong, Malaysia; Malaysian Academy of Pharmacy, Puchong, Malaysia (Prof N E Ismail PhD); Department of Health Services Research (M Iwagami PhD), University of Tsukuba, Tsukuba, Japan; Department of Non-Communicable Disease Epidemiology (M Iwagami PhD), London School of Hygiene & Tropical Medicine, London, UK; The World Academy of Sciences UNESCO, Trieste, Italy (Prof M Jakovljevic PhD); Shaanxi University of Technology, Hanzhong, China (Prof M Jakovljevic PhD); Johns Hopkins University, Baltimore, MD, USA (E Jamshidi PharmD); Centre of Studies and Research (S Jayapal PhD), Ministry of Health, Muscat, Oman; Department of Biochemistry (Prof S Jayaram MD), Government Medical College, Mysuru, India; Caring Futures Institute (D M Jemere MBA), Department of Nursing and Health Sciences (S Shorofi PhD), Flinders University, Adelaide, SA, Australia; Geumsan Public Health Center, Geumsan-gun, South Korea (G Jeong MD); College of Medicine (G Jeong MD), Gyeongsang National University, Jinju, South Korea; Department of Community Medicine (N Joseph MD), Manipal Academy of Higher Education, Mangalore, India; Department of Economics (C E Joshua BSc), National Open University, Benin City, Nigeria; Institute of Family Medicine and Public Health (M Jürisson PhD), University of Tartu, Tartu, Estonia; Department of Oral and Maxillofacial Pathology (V Kadashetti MDS), Krishna Vishwa Vidyapeeth (Deemed to be University), Karad, India; Department of Endocrinology (S Kalra DM), Bharti Hospital Karnal, Karnal, India; Department of Public Health (M A Khafaie PhD), Environmental Technologies Research Center, Medical Basic Sciences Research Institute (M A Khafaie PhD), Department of Microbiology (M Saki PhD), Ahvaz Jundishapur University of Medical Sciences, Ahvaz, Iran; Amity Institute of Forensic Sciences (H Khajuria PhD, B P Nayak PhD), Amity Institute of

Public Health (M Shannawaz PhD), Amity University, Noida, India; Primary Care Department (M A Khan MSc), NHS North West London, London, UK; School of Medicine (S Khateri MD), Department of Epidemiology and Biostatistics (Y Moradi PhD), Kurdistan University of Medical Sciences, Sanandaj, Iran; Broad Institute of MIT and Harvard, Cambridge, MA, USA (M Kim MD); Department of General Practice and Family Medicine (Prof O Korzh DSc), Kharkiv National Medical University, Kharkiv, Ukraine; Department of Anthropology (Prof K Krishan PhD), Panjab University, Chandigarh, India; Center of Medicine and Public Health (M Kulimbet MSc), Asfendiyarov Kazakh National Medical University, Almaty, Kazakhstan; Department of Medicine (V Kulkarni MS), Queensland Health, Brisbane, QLD, Australia; Faculty of Medicine and Health Science (M Kurniasari PhD), Universitas Kristen Satya Wacana, Salatiga, Indonesia; School of Nursing (M Kurniasari PhD), Department of Global Health and Health Security (K Latief PhD), Taipei Medical University, Taipei, Taiwan; Division of Evidence Synthesis (C Lahariya MD), Foundation for People-centric Health Systems, New Delhi, India; Division of Lifestyle Medicine (C Lahariya MD), Centre for Health: The Specialty Practice, New Delhi, India; Department of Physiotherapy (T Laksono MS), Universitas Aisyiyah Yogyakarta, Yogyakarta, Indonesia; Institute of Allied Health Sciences (T Laksono MS), National Cheng Kung University, Tainan, Taiwan; Unidad de Genética y Salud Pública (Prof I Landires MD), Instituto de Ciencias Médicas, Las Tablas, Panama; Ministry of Health (Prof I Landires MD), Hospital Joaquín Pablo Franco Sayas, Las Tablas, Panama; Centre for Family Welfare (K Latief PhD), University of Indonesia, Depok, Indonesia; Department of General Medicine (V T Nguyen MD), University of Medicine and Pharmacy at Ho Chi Minh City, Ho Chi Minh City, Viet Nam (T T Le MD); Department of Medical Science (M Lee PhD), Ajou University School of Medicine, Suwon, South Korea; Department of Family Medicine (W Lee PhD), University of Texas Medical Branch, Galveston, TX, USA; Department of Molecular Epidemiology (E Llanaj PhD), German Institute of Human Nutrition Potsdam-Rehbrücke, Potsdam, Germany; German Center for Diabetes Research (DZD), München-Neuherberg, Germany (E Llanaj PhD); Rama Medical College Hospital and Research Centre, Uttar Pradesh, India (K Malhotra MBBS); Institute of Applied Health Research (K Malhotra MBBS), University of Birmingham, Birmingham, UK; Rabigh Faculty of Medicine (Prof A Malik PhD), Department of Dental Public Health (Z S Natto DrPH), King Abdulaziz University, Jeddah, Saudi Arabia; Department of Nutrition and Dietetics (M Martorell PhD), Centre for Healthy Living (M Martorell PhD), University of Concepción, Concepción, Chile; Department of Medical and Surgical Sciences and Advanced Technologies "GF Ingrassia" (A Maugeri PhD), University of Catania, Catania, Italy; Comprehensive Cancer Center (T J Meretoja MD), Helsinki University Hospital, Helsinki, Finland; University of Helsinki, Helsinki, Finland (T J Meretoja MD); University Centre Varazdin (T Mestrovic PhD), University North, Varazdin, Croatia; Molecular Biology Unit (N S Mohamed MSc), Bio-Statistical and Molecular Biology Department (N S Mohamed MSc), Sirius Training and Research Centre, Khartoum, Sudan; Modeling in Health Research Center (A Mohammadian-Hafshejani PhD), Shahrekord University of Medical Sciences, Shahrekord, Iran; Clinical Epidemiology and Public Health Research Unit (L Monasta DSc), Burlo Garofolo Institute for Maternal and Child Health, Trieste, Italy; Clinical Research Development Unit (N Morovatdar MD), Biotechnology Research Center (Prof A Sahebkar PhD), Mashhad University of Medical Sciences, Mashhad, Iran; Division of Plastic and Reconstructive Surgery (S D Morrison MD), University of Washington Medical Center, Seattle, WA, USA; Department of Medicine (E Mostafavi PhD), Stanford Cardiovascular Institute (E Mostafavi PhD), Stanford University, Palo Alto, CA, USA; Unit of Pharmacotherapy, Epidemiology and Economics (Prof S Mubarik PhD), Rijksuniversiteit Groningen (University of Groningen), Groningen, Netherlands; Department of Epidemiology and Biostatistics (Prof S Mubarik PhD, Prof C Yu PhD), Wuhan University, Wuhan, China; Department of Research Methods (S

Muthu PhD), Orthopaedic Research Group, Coimbatore, India; Department of Biotechnology (S Muthu PhD), Karpagam Academy of Higher Education (Deemed to be University), Coimbatore, India; Department of Computer Science (P Naghavi MS), University of Illinois, Urbana, IL, USA; Department of Health Policy and Oral Epidemiology (Z S Natto DrPH), Beth Israel Deaconess Medical Center (S Sharfaei MD), Harvard University, Boston, MA, USA; Applied Cellular and Molecular Research Center (M H Nematollahi PhD), Kerman University of Medical Sciences, Kerman, Iran; Cardiovascular Laboratory (D H Nguyen MD), Cardiovascular Research Department (H Q Nguyen MD), Methodist Hospital, Merrillville, IN, USA; Department of Allergy, Immunology and Dermatology (D H Nguyen MD), Hanoi Medical University, Hanoi, Viet Nam; Tuberculosis Group (V T Nguyen MD), Oxford University Clinical Research Unit, Vietnam, Ho Chi Minh City, Viet Nam; International Islamic University Islamabad, Islamabad, Pakistan (R K Niazi PhD); Department of Public Health (D Nurrika PhD), Banten School of Health Science, South Tangerang, Indonesia; Ministry of Research, Technology and Higher Education (D Nurrika PhD), Higher Education Service Institutions (LL-DIKTI) Region IV, Bandung, Indonesia; School of Pharmacy (O C Okonji MSc), University of the Western Cape, Cape Town, South Africa; Department of Pharmacotherapy and Pharmaceutical Care (M Ordak PhD), Department of Biochemistry and Pharmacogenomics (M Zielińska MPharm), Medical University of Warsaw, Warsaw, Poland; Department of Biology (W M S Osman PhD), Khalifa University, Abu Dhabi, United Arab Emirates; National School of Public Health (A Padron-Monedero PhD), Institute of Health Carlos III, Madrid, Spain; Vision and Eye Research Institute (Prof S Pardhan PhD), Anglia Ruskin University, Cambridge, UK; Department of Community Medicine (P P Parija MD), All India Institute of Medical Sciences, Jammu, India; Division of Health Policy and Management (R R Parikh MD), University of Minnesota, Minneapolis, MN, USA; Global Health Governance Programme (J Patel BSc), University of Edinburgh, Edinburgh, UK; Facultad de Medicina (Faculty of Medicine) (F E Petermann-Rocha PhD), Universidad Diego Portales (Diego Portales University), Santiago, Chile; School of Cardiovascular and Metabolic Health (F E Petermann-Rocha PhD), University of Glasgow, Glasgow, UK; Department of Internal Medicine (H T Pham MD), Weiss Memorial Hospital, Chicago, IL, USA; Department of Maternal-Child Nursing and Public Health (E J S Prates BS), Federal University of Minas Gerais, Belo Horizonte, Brazil; Department of Neonatology (I Qattea MD), Case Western Reserve University, Akron, OH, USA; Department of Population Science and Human Resource Development (Prof M Rahman DrPH), University of Rajshahi, Rajshahi, Bangladesh; Health Service Research and Quality of Life Center (CEReSS) (Prof M Rahmati PhD), Aix-Marseille University, Marseille, France; Department of Medical, Surgical and Experimental Sciences (I Raimondo MD), University of Sassari, Sassari, Italy; Gynecology and Breast Care Center (I Raimondo MD), Mater Olbia Hospital, Olbia, Italy; Department of Radiology (S K Ramasamy MD), Stanford University, Stanford, CA, USA; Brigham and Women's Hospital (S Rashedi MD), Harvard Medical School, Boston, MA, USA; Department of Primary Care and Public Health (Prof S Rawaf MD), Imperial College London, London, UK; Academic Public Health England (Prof S Rawaf MD), Public Health England, London, UK; Department of Biological Sciences (Prof E Redwan PhD), King Abdulaziz University, Jeddah, Egypt; Department of Protein Research (Prof E Redwan PhD), Research and Academic Institution, Alexandria, Egypt; Cardiovascular Department (Prof A M A Saad MD), Zagazig University, Zagazig, Egypt; Operational Research Center in Healthcare (Prof U Saeed PhD), Near East University (NEU), Nicosia Cyprus, Türkiye; International Center of Medical Sciences Research (ICMSR), Islamabad, Pakistan (Prof U Saeed PhD); Center for Global Health Research (Prof A Sahebkar PhD), Saveetha University, Chennai, India; LMU-Munich, Munich, Germany (J W Sakshaug PhD); Institute for Employment Research, Nuremberg, Germany (J W Sakshaug PhD); Faculty of Pharmacy (Prof M A Saleh PhD), Mansoura University,

Mansoura, Egypt; Institute of Epidemiology and Preventive Medicine (Y L Samodra PhD), National Taiwan University, Taipei, Taiwan; Benang Merah Research Center (BMRC), Minahasa Utara, Indonesia (Y L Samodra PhD); Department of Entomology (A M Samy PhD), Medical Ain Shams Research Institute (MASRI) (A M Samy PhD), Ain Shams University, Cairo, Egypt; Department of Biomedical and Neuromotor Sciences (F Sanmarchi MD), University of Bologna, Bologna, Italy; Primary Healthcare Department (F Sanmarchi MD), Azienda USL di Bologna, Bologna, Italy; Research Development Coordination Section (M A N Saqib PhD), Pakistan Health Research Council, Islamabad, Pakistan; School of Sciences (M A N Saqib PhD), University of Management and Technology, Lahore, Pakistan; Faculty of Medicine (A Schuermans BSc), Department of Cardiovascular Sciences (A Schuermans BSc), Katholieke Universiteit Leuven, Leuven, Belgium; Department of Medicine (Y Sethi MD), Swami Vivekanand Subharti University, Meerut, India; National Heart, Lung, and Blood Institute (A Seylani MD), National Institutes of Health, Rockville, MD, USA; Center for Medical and Bio-Allied Health Sciences Research (Prof M J Shahwan PhD), Ajman University, Ajman, United Arab Emirates; Department of Pathology and Laboratory Medicine (S Sham MD), Northwell Health, New York, NY, USA; Department of Safety Services (S Sharfaei MD), Baim Institute for Clinical Research, Boston, MA, USA; Department of Social and Behavioral Health (Prof M Sharma PhD), University of Nevada Las Vegas, Las Vegas, NV, USA; Department of Medical-Surgical Nursing (S Shorofi PhD), Mazandaran University of Medical Sciences, Sari, Iran; Unit of Basic Medical Sciences (E E Siddig MD), University of Khartoum, Khartoum, Sudan; Department of Medical Microbiology and Infectious Diseases (E E Siddig MD), Erasmus University, Rotterdam, Netherlands; Sport Physical Activity and Health Research & Innovation Center (SPRINT) (Prof L M L R Silva PhD), Polytechnic Institute of Guarda, Guarda, Portugal; CICS-UBI Health Sciences Research Center (Prof L M L R Silva PhD), University of Beira Interior, Covilhã, Portugal; School of Medicine (Prof J A Singh MD), Baylor College of Medicine, Houston, TX, USA; Department of Medicine Service (Prof J A Singh MD), US Department of Veterans Affairs (VA), Houston, TX, USA; Department of Radiodiagnosis (P Singh MD), All India Institute of Medical Sciences, Bathinda, India; Department of Analytical and Applied Economics (C Swain MPhil), Utkal University, Bhubaneswar, India; Department of Epidemiology (J L Tamuzi MSc), Stellenbosch University, Cape Town, South Africa; Department of Medicine (J L Tamuzi MSc), Northlands Medical Group, Omuthiya, Namibia; Basic Sciences in Infectious Diseases Research Center (R Tavakoli Oliaee PhD), Shiraz University of Medical Sciences, Shiraz, Iran; Department of Preventive Medicine (M Teramoto MD), Northwestern University, Chicago, IL, USA; Outpatient Department (D R Terefa MSc), Wollega University, Bedele Town, Ethiopia; Faculty of Public Health (J H V Ticoalu MPH), Universitas Sam Ratulangi (Sam Ratulangi University), Manado, Indonesia; UKK Institute, Tampere, Finland (Prof T J Vasankari PhD); Faculty of Medicine and Health Technology (Prof T J Vasankari PhD), Tampere University, Tampere, Finland; Department of Infectious Disease (Prof S Vaziri MD), Kermanshah University of Medical Sciences, Kermanshah, Iran; School of Public Health (F Wang PhD), Xuzhou Medical University, Xuzhou, China; Department of Neurosurgery (S Wang MD), School of Public Health (J Xia PhD), Capital Medical University, Beijing, China; Department of Neurosurgery (S Wang MD), Beijing Tiantan Hospital, Beijing, China; Department of Biostatistics (Prof N Yonemoto PhD), University of Toyama, Toyama, Japan; Department of Public Health (Prof N Yonemoto PhD), Juntendo University, Tokyo, Japan; Norris Comprehensive Cancer Center, Keck School of Medicine (M Zahir MD), University of Southern California, Los Angeles, CA, USA; College of Traditional Chinese Medicine (H Zhao MD), Hebei University, Baoding, China; School of Public Health Sciences (O A Zitoun MD), University of Waterloo, Waterloo, ON, Canada; College of Medicine (O A Zitoun MD), Sulaiman Alrajhi University, Al

Bukairiyah, Saudi Arabia; Department of Rheumatology (Prof L M March PhD), Royal North Shore Hospital, St Leonards, NSW, Australia

Authors' Contributions

Providing data or critical feedback on data sources

Yohannes Habtegiorgis Abate, Qorinah Estiningtyas Sakilah Adnani, Haroon Ahmed, Rasmieh Mustafa Al-amer, Hediyeh Alemi, Abid Ali, Jalal Arabloo, Seyyed Shamsadin Athari, Ahmed Y Azzam, Ashish D Badiye, Sara Bagherieh, Maciej Banach, Sonu Bhaskar, Gurjit Kaur Bhatti, Dana Bryazka, Vijay Kumar Chattu, Dinh-Toi Chu, Michael H Criqui, Natalia Cruz-Martins, Xiaochen Dai, Tadesse Asmamaw Dejenie, Vinoth Gnana Chellaiyan Devanbu, Vishal R Dhulipala, Karsten E Dreinhoefer, Michael Ekholuenetale, Adeniyi Francis Fagbamigbe, Ildar Ravisovich Fakhradiyev, Ali Fatehizadeh, Alireza Feizkhah, Ginenus Fekadu, Ali Golchin, Sapna Gupta, Najah R Hadi, Arvin Haj-Mirzaian, Philippe Halbout, Golnaz Heidari, Praveen Hoogar, Evelyn Hsieh, Segun Emmanuel Ibitoye, Nahlah Elkudssiah Ismail, Mihajlo Jakovljevic, Sathish Kumar Jayapal, Shubha Jayaram, Digisie Mequanint Jemere, Charity Ehimwenma Joshua, Vidya Kadashetti, Morteza Abdullatif Khafaie, Himanshu Khajuria, Moien AB Khan, Shaghayegh Khanmohammadi, Sorour Khateri, Min Seo Kim, Oleksii Korzh, Kewal Krishan, Maria Dyah Kurniasari, Chandrakant Lahariya, Kamaluddin Latief, Thao Thi Thu Le, Munjae Lee, Wei-Chen Lee, Erand Llanaj, Kashish Malhotra, Lyn M March, Nouh Saad Mohamed, Abdollah Mohammadian-Hafshejani, Ali H Mokdad, Lorenzo Monasta, Yousef Moradi, Sumaira Mubarik, Christopher J L Murray, Mohsen Naghavi, Zuhair S Natto, Biswa Prakash Nayak, Hien Quang Nguyen, Van Thanh Nguyen, Robina Khan Niazi, Dieta Nurrika, Kanyin Liane Ong, Romil R Parikh, Hoang Tran Pham, Elton Junio Sady Prates, Daniel Prieto-Alhambra, Ibrahim Qattea, Mehran Rahimi, Vafa Rahimi-Movaghar, Masoud Rahmati, Ivano Raimondo, Shakthi Kumaran Ramasamy, Sina Rashedi, Salman Rawaf, Phoebe-Anne Rhinehart, Aly M A Saad, Umar Saeed, Morteza Saki, Abdallah M Samy, Lidia Sanchez-Riera, Yashendra Sethi, Allen Seylani, Sunder Sham, Mohammed Shannawaz, Luís Manuel Lopes Rodrigues Silva, Jasvinder A Singh, Paramdeep Singh, Chandan Kumar Swain, Shima Tabatabai, Dufera Rikitu Terefa, Tommi Juhani Vasankari, Theo Vos, Shu Wang, Anthony D Woolf, Juan Xia, Naohiro Yonemoto, Chuanhua Yu, and Magdalena Zielińska.

Developing methods or computational machinery

Aleksandr Y Aravkin, Garland T Culbreth, Xiaochen Dai, Simon I Hay, Jiawei He, Ali H Mokdad, Christopher J L Murray, Mohsen Naghavi, Kanyin Liane Ong, and Theo Vos.

Providing critical feedback on methods or results

Yohannes Habtegiorgis Abate, Sherief Abd-Elsalam, Meriem Abdoun, Mohamed Abouzid, Eman Abu-Gharbieh, Salahdein Aburuz, Abiola Victor Adepoju, Qorinah Estiningtyas Sakilah Adnani, Aqeel Ahmad, Haroon Ahmed, Luai A Ahmed, Syed Mahfuz Al Hasan, Rasmieh Mustafa Al-amer, Hediyeh Alemi, Abid Ali, Yaser Mohammed Al-Worafi, Jalal Arabloo, Demelash Areda, Mohammad Asghari-Jafarabadi, Seyyed Shamsadin Athari, Sina Azadnajafabad, Ahmed Y Azzam, Ashish D Badiye, Nasser Bagheri, Sara Bagherieh, Saliu A Balogun, Maciej Banach, Pankaj Bhardwaj, Sonu Bhaskar, Gurjit Kaur Bhatti, Dana Bryazka, Yasser Bustanji, Vijay Kumar Chattu, Endeshaw Chekol Abebe, Dinh-Toi Chu, Michael H Criqui, Marita Cross, Natalia Cruz-Martins, Omid Dadras, Xiaochen Dai, Zhaoli Dai, Reza Darvishi Cheshmeh Soltani, Mohsen Dashti, Tadesse Asmamaw Dejenie, Cristian Del Bo', Vinoth Gnana Chellaiyan Devanbu, Vishal R Dhulipala, Karsten E Dreinhoefer, Michael Ekholuenetale, Mohamed A Elmonem, Farshid Etaee,

Adeniyi Francis Fagbamigbe, Ildar Ravisovich Fakhradiyev, Ali Fatehizadeh, Alireza Feizkhah, Ginenus Fekadu, Bikila Regassa Feyisa, Florian Fischer, Abduzhappar Gaipov, Lucia Galluzzo, Mesfin Gebrehiwot, Tiffany K Gill, Kimiya Gohari, Ali Golchin, Bhawna Gupta, Sapna Gupta, Najah R Hadi, Arvin Haj-Mirzaian, Philippe Halbout, Asif Hanif, Netanja I Harlianto, Ikramul Hasan, Md Saquib Hasnain, Simon I Hay, Golnaz Heidari, Kamal Hezam, Yuta Hiraike, Praveen Hoogar, Evelyn Hsieh, Chengxi Hu, Segun Emmanuel Ibitoye, Arad Iranmehr, Nahlah Elkudssiah Ismail, Masao Iwagami, Ali Jafari-Khounigh, Mihajlo Jakovljevic, Elham Jamshidi, Sathish Kumar Jayapal, Shubha Jayaram, Digisie Mequanint Jemere, Gwang Hun Jeong, Nitin Joseph, Charity Ehimwenma Joshua, Mikk Jürisson, Vidya Kadashetti, Sanjay Kalra, Morteza Abdullatif Khafaie, Himanshu Khajuria, Moien AB Khan, Javad Khanali, Shaghayegh Khanmohammadi, Moawiah Mohammad Khatatbeh, Sorour Khateri, Jacek A Kopec, Oleksii Korzh, Kewal Krishan, Maria Dyah Kurniasari, Chandrakant Lahariya, Tri Laksono, Iván Landires, Kamaluddin Latief, Thao Thi Thu Le, Munjae Lee, Wei-Chen Lee, Erand Llanaj, Kashish Malhotra, Ahmad Azam Malik, Lyn M March, Miquel Martorell, Andrea Maugeri, Hadush Negash Meles, Mohsen Merati, Tomislav Mestrovic, Alireza Mirahmadi, Nouh Saad Mohamed, Abdollah Mohammadian-Hafshejani, Ali H Mokdad, Yousef Moradi, Negar Morovatdar, Ebrahim Mostafavi, Sumaira Mubarik, Christopher J L Murray, Sathish Muthu, Mohsen Naghavi, Pirouz Naghavi, Zuhair S Natto, Biswa Prakash Nayak, Duc Hoang Nguyen, Hien Quang Nguyen, Van Thanh Nguyen, Robina Khan Niazi, Efaq Ali Noman, Dieta Nurrika, Osaretin Christabel Okonji, Kanyin Liane Ong, Michal Ordak, Wael M S Osman, Yasamin Ostadi, Shahina Pardhan, Pragyan Paramita Parija, Romil R Parikh, Jay Patel, Fanny Emily Petermann-Rocha, Hoang Tran Pham, Elton Junio Sady Prates, Daniel Prieto-Alhambra, Mehran Rahimi, Vafa Rahimi-Movaghar, Mosiur Rahman, Masoud Rahmati, Shakthi Kumaran Ramasamy, Sina Rashedi, Mohammad-Mahdi Rashidi, Salman Rawaf, Elrashdy M Redwan, Nazila Rezaei, Phoebe-Anne Rhinehart, Aly M A Saad, Umar Saeed, Amene Saghazadeh, Fatemeh Saheb Sharif-Askari, Morteza Saki, Joseph W Sakshaug, Mohamed A Saleh, Yoseph Leonardo Samodra, Abdallah M Samy, Lidia Sanchez-Riera, Muhammad Arif Nadeem Saqib, Art Schuermans, Yashendra Sethi, Mohammed Shannawaz, Sadaf Sharfaei, Seyed Afshin Shorofi, Emmanuel Edwar Siddig, Luís Manuel Lopes Rodrigues Silva, Jasvinder A Singh, Paramdeep Singh, Hamidreza Soleimani, Chandan Kumar Swain, Shima Tabatabai, Jacques Lukenze Tamuzi, Razieh Tavakoli Oliaee, Seyed Mohammad Tavangar, Masayuki Teramoto, Dufera Rikitu Terefa, Jansje Henny Vera Ticoalu, Siavash Vaziri, Theo Vos, Fang Wang, Shu Wang, Anthony D Woolf, Juan Xia, Naohiro Yonemoto, Chuanhua Yu, Mazyar Zahir, Hanqing Zhao, Magdalena Zielińska, and Osama A Zitoun.

Drafting the work or revising it critically for important intellectual content

Yohannes Habtegiorgis Abate, Sherief Abd-Elsalam, Mohamed Abouzid, Eman Abu-Gharbieh, Salahdein Aburuz, Qorinah Estiningtyas Sakilah Adnani, Haroon Ahmed, Luai A Ahmed, Syed Mahfuz Al Hasan, Tariq A Alalwan, Rasmieh Mustafa Al-amer, Hediyeh Alemi, Abid Ali, Yaser Mohammed Al-Worafi, Reza Amani, Abhishek Anil, Jalal Arabloo, Brhane Berhe Aregawi, Seyyed Shamsadin Athari, Sina Azadnajafabad, Ahmed Y Azzam, Ashish D Badiye, Sara Bagherieh, Saliu A Balogun, Maciej Banach, Shirin Barati, Sonu Bhaskar, Gurjit Kaur Bhatti, Dana Bryazka, Yasser Bustanji, Daniela Calina, Vijay Kumar Chattu, Endeshaw Chekol Abebe, Cyrus Cooper, Ewerton Cousin, Marita Cross, Natalia Cruz-Martins, Garland T Culbreth, Zhaoli Dai, Cristian Del Bo', Edgar Denova-Gutiérrez, Syed Masudur Rahman Dewan, Vishal R Dhulipala, Karsten E Dreinhoefer, Michael Ekholuenetale, Mohamed A Elmonem, Adeniyi Francis Fagbamigbe, Ali Fatehizadeh, Florian Fischer, Lucia Galluzzo, Fataneh Ghadirian, Tiffany K Gill, Ali Golchin, Bhawna Gupta, Sapna Gupta, Najah R Hadi, Arvin Haj-Mirzaian, Philippe Halbout, Netanja I Harlianto, Md Saquib Hasnain, Amr Hassan, Simon I Hay, Golnaz Heidari, Kamal Hezam, Yuta Hiraike, Evelyn Hsieh, Chengxi Hu, Segun Emmanuel Ibitoye, Arad Iranmehr, Nahlah Elkudssiah Ismail, Mihajlo

Jakovljevic, Sathish Kumar Jayapal, Shubha Jayaram, Digisie Mequanint Jemere, Gwang Hun Jeong, Nitin Joseph, Charity Ehimwenma Joshua, Mikk Jürisson, Vidya Kadashetti, Sanjay Kalra, Himanshu Khajuria, Moien AB Khan, Javad Khanali, Moawiah Mohammad Khatatbeh, Min Seo Kim, Oleksii Korzh, Kewal Krishan, Mukhtar Kulimbet, Vishnutheertha Kulkarni, Maria Dyah Kurniasari, Chandrakant Lahariya, Iván Landires, Kamaluddin Latief, Thao Thi Thu Le, Wei-Chen Lee, Erand Llanaj, Kashish Malhotra, Ahmad Azam Malik, Lyn M March, Miquel Martorell, Andrea Maugeri, Hadush Negash Meles, Mohsen Merati, Tuomo J Meretoja, Tomislav Mestrovic, Alireza Mirahmadi, Nouh Saad Mohamed, Abdollah Mohammadian-Hafshejani, Ali H Mokdad, Lorenzo Monasta, Shane Douglas Morrison, Ebrahim Mostafavi, Parsa Mousavi, Christopher J L Murray, Sathish Muthu, Mohsen Naghavi, Zuhair S Natto, Biswa Prakash Nayak, Mohammad Hadi Nematollahi, Duc Hoang Nguyen, Hien Quang Nguyen, Van Thanh Nguyen, Robina Khan Niazi, Sneha Ingle Nicholson, Osaretin Christabel Okonji, Kanyin Liane Ong, Michal Ordak, Wael M S Osman, Yasamin Ostadi, Alicia Padron-Monedero, Shahina Pardhan, Romil R Parikh, Jay Patel, Fanny Emily Petermann-Rocha, Hoang Tran Pham, Elton Junio Sady Prates, Daniel Prieto-Alhambra, Ibrahim Qattea, Vafa Rahimi-Movaghar, Masoud Rahmati, Ivano Raimondo, Shakthi Kumaran Ramasamy, Salman Rawaf, Elrashdy M Redwan, Phoebe-Anne Rhinehart, Aly M A Saad, Umar Saeed, Fatemeh Saheb Sharif-Askari, Amirhossein Sahebkar, Morteza Saki, Abdallah M Samy, Lidia Sanchez-Riera, Francesco Sanmarchi, Art Schuermans, Yashendra Sethi, Allen Seylani, Moyad Jamal Shahwan, Mohammed Shannawaz, Manoj Sharma, Seyed Afshin Shorofi, Emmanuel Edwar Siddig, Luís Manuel Lopes Rodrigues Silva, Jasvinder A Singh, Paramdeep Singh, Hamidreza Soleimani, Chandan Kumar Swain, Shima Tabatabai, Jacques Lukenze Tamuzi, Razieh Tavakoli Oliaee, Seyed Mohammad Tavangar, Dufera Rikitu Terefa, Asokan Govindaraj Vaithinathan, Tommi Juhani Vasankari, Theo Vos, Fang Wang, Shu Wang, Anthony D Woolf, Juan Xia, Naohiro Yonemoto, Mazyar Zahir, Magdalena Zielińska, and Osama A Zitoun.

Managing the estimation or publications process

Hailey Hagins, Simon I Hay, Evelyn Hsieh, Lyn M March, Ali H Mokdad, Christopher J L Murray, Mohsen Naghavi, Sneha Ingle Nicholson, and Kanyin Liane Ong.
